# A systematic review of the effectiveness of strategies to improve health care provider performance in low- and middle-income countries: Methods and descriptive results

**DOI:** 10.1371/journal.pone.0217617

**Published:** 2019-05-31

**Authors:** Samantha Y. Rowe, David H. Peters, Kathleen A. Holloway, John Chalker, Dennis Ross-Degnan, Alexander K. Rowe

**Affiliations:** 1 Malaria Branch, Division of Parasitic Diseases and Malaria, Center for Global Health, Centers for Disease Control and Prevention, Atlanta, Georgia, United States of America; 2 CDC Foundation, Atlanta, Georgia, United States of America; 3 Department of International Health, Johns Hopkins Bloomberg School of Public Health, Baltimore, Maryland, United States of America; 4 World Health Organization, Southeast Asia Regional Office, New Delhi, India; 5 International Institute of Health Management Research, Jaipur, India; 6 Pharmaceuticals & Health Technologies Group, Management Sciences for Health, Arlington, Virginia, United States of America; 7 Harvard Medical School, Boston, Massachusetts, United States of America; 8 Harvard Pilgrim Health Care Institute, Boston, Massachusetts, United States of America; Ruprecht Karls University Heidelberg, GERMANY

## Abstract

**Background:**

Health care provider (HCP) performance in low- and middle-income countries (LMICs) is often inadequate. The Health Care Provider Performance Review (HCPPR) is a comprehensive systematic review of the effectiveness and cost of strategies to improve HCP performance in LMICs. We present the HCPPR’s methods, describe methodological and contextual attributes of included studies, and examine time trends of study attributes.

**Methods:**

The HCPPR includes studies from LMICs that quantitatively evaluated any strategy to improve HCP performance for any health condition, with no language restrictions. Eligible study designs were controlled trials and interrupted time series. In 2006, we searched 15 databases for published studies; in 2008 and 2010, we completed searches of 30 document inventories for unpublished studies. Data from eligible reports were double-abstracted and entered into a database, which is publicly available. The primary outcome measure was the strategy’s effect size. We assessed time trends with logistic, Poisson, and negative binomial regression modeling. We were unable to register with PROSPERO (International Prospective Register of Systematic Reviews) because the protocol was developed prior to the PROSPERO launch.

**Results:**

We screened 105,299 citations and included 824 reports from 499 studies of 161 intervention strategies. Most strategies had multiple components and were tested by only one study each. Studies were from 79 countries and had diverse methodologies, geographic settings, HCP types, work environments, and health conditions. Training, supervision, and patient and community supports were the most commonly evaluated strategy components. Only 33.6% of studies had a low or moderate risk of bias. From 1958–2003, the number of studies per year and study quality increased significantly over time, as did the proportion of studies from low-income countries. Only 36.3% of studies reported information on strategy cost or cost-effectiveness.

**Conclusions:**

Studies have reported on the efficacy of many strategies to improve HCP performance in LMICs. However, most studies have important methodological limitations. The HCPPR is a publicly accessible resource for decision-makers, researchers, and others interested in improving HCP performance.

## Introduction

Each year in low- and middle-income countries (LMICs), millions of children and adults die prematurely [1,2]; although many interventions exist that can prevent such deaths [3–6]. Low coverage of these interventions has been identified as a critical public health problem [3,6] and a major obstacle to achieving Millennium Development Goals [4] and the Sustainable Development Goals [7].

A key part of almost any strategy for increasing the effective coverage of health interventions involves health care providers (HCPs), including health workers in hospitals, clinics, pharmacies, drug shops, and communities. However, HCP performance in LMICs is often inadequate, as documented in studies of child health [8,9], sexually transmitted diseases [10], obstetrics [11,12], mental disorders [13], injuries [14], diabetes [15], malaria [16, 17], medicine use [18], and illnesses managed in hospitals [19] and by private sector health workers [18,20]. The global burden of unsafe medical care in LMICs is high, conservatively estimated at more than 33 million disability-adjusted life years lost annually [21,22]. Notably, inadequate care occurs despite substantial efforts by governments, non-governmental organizations, and donors.

Improving HCP performance is essential, as it involves preventing errors of omission (e.g., patients not receiving needed medicines), as well as avoiding harmful practices (e.g., giving sedatives to children with pneumonia [9]) and improving the patient’s experience [23]. Some research suggests that improving performance might increase utilization of health services [24].

Numerous studies in LMICs have evaluated a wide variety of strategies to improve HCP performance. Systematic reviews that distill the evidence on effectiveness and cost can be valuable for guiding policy to reduce medical errors, focusing programmatic efforts on strategies with relatively greater effectiveness, and avoiding strategies that are relatively ineffective.

Many existing systematic reviews have focused on specific strategies, such as training [25–30], computer-based training [31], distance learning [32], essential drug programs [33], integration of services [34], job aids [35,36], lay health workers [37], self-assessment [38], supervision [39,40], incentives [41], and telemedicine [42]. Some of these reviews focus exclusively on LMICs, while others include studies from LMICs and high-income countries. However, a key limitation of single-strategy reviews is that they only partly address the fundamental programmatic question: what are the most effective and affordable ways to improve HCP performance? To answer this broader question for the LMIC context, all strategies tested in LMICs must be examined and compared.

Several systematic reviews have included multiple, but not all, strategies. The largest of these reviews [43] had few studies from LMICs. Four reviews presented only descriptive or semi-quantitative summaries [44–47]. One review, which was updated several times, focused on strategies to improve medicine use in LMICs [18,48–50]. At least four reviews of systematic reviews of single strategies have been completed [51–54].

Existing reviews often have other important limitations. First, they rarely summarize economic data on strategy cost or cost-effectiveness [29]. Second, some reviews do not use methods that have become standard in the field of systematic reviews [44–46]. Third, results of strategy-versus-strategy (i.e., head-to-head) comparisons are often not integrated with results of strategy-versus-control comparisons, which underutilizes a large portion of the evidence base [25,40,46,49]. Fourth, the databases on which the reviews are based are either not publicly available or only available as a static table, which limits their usability [25,43,44,46]. Additionally, existing reviews use such heterogeneous methods that it is difficult to synthesize their results. For example, measures of strategy effectiveness have included risk differences, adjusted risk differences, relative risks, adjusted relative risks, and non-quantitative categories.

An updated quantitative systematic review of multiple strategies is needed that includes all strategies, all facets of HCP performance, economic data, head-to-head studies, a publicly available database in a dynamic format, the use of a single analytic framework, and state-of-the-art methods for systematic reviews. The Health Care Provider Performance Review (HCPPR) is a systematic review designed to help fill this gap. The primary objective is to assess individually the effectiveness and cost of all strategies to improve HCP performance outcomes in LMICs (effectively, a series of parallel systematic reviews), including both strategy-versus-control comparisons and head-to-head comparisons, from controlled and interrupted time series (ITS) studies. Specific objectives of the review include the following:

1. Produce a publicly available database of studies on improving HCP performance for program managers and other decision-makers, policy analysts, donors, technical agencies, and researchers;
2. Conduct analyses to estimate the effectiveness of a wide variety of strategies, including combinations of strategies, to improve HCP performance, and comparisons to identify more and less effective strategies;
3. Conduct in-depth analyses of strategies involving training and supervision to identify attributes associated with greater effectiveness;
4. Develop evidence-based guidance on how to improve HCP performance in LMICs; and
5. Contribute to a research agenda to fill critical knowledge gaps on how to improve HCP performance.

Now is a particularly important time to conduct systematic reviews, such as the HCPPR, on improving HCP performance. The large growth in donor funding in the past decade [55] provides an enormous opportunity to improve health in LMICs, and strengthening HCP performance has the potential to increase the effectiveness and efficiency of programs supported by such funding. Improving HCP performance will also be essential for meeting a target of the Sustainable Development Goals that calls for achieving universal health coverage, which requires “access to quality essential health-care services” [56]. More generally, research on improving HCP performance fits within the larger public health priorities of conducting research to strengthen human resources for health [57,58] and health systems [59,60].

## Materials and methods

The methods and results of our systematic review are presented in a series of articles that, taken together, include all elements recommended by the Preferred Reporting Items for Systematic Reviews and Meta-analyses (PRISMA) guidelines [61]. This article presents the review’s methodology and contextual attributes of included studies, and examines time trends of some of these attributes. Articles in preparation will present results on strategy effectiveness, training and supervision strategies, and a network meta-analysis of results. The PRISMA checklist (S1 File) and study protocol (S2 File) are available as on-line Supporting Information files. We attempted to register our protocol with PROSPERO (International prospective register of systematic reviews). However, the protocol for this review was developed and the review was underway prior to the launch of PROSPERO and as such, it was ineligible to be registered. We were unable to identify another site to register the protocol.

### Eligibility criteria

The eligibility criteria were adapted from Grimshaw et al. [43]. We included published and unpublished studies conducted in LMICs that quantitatively evaluated a strategy to improve HCP performance. Eligible strategies had to include at least one component that plausibly could affect HCP performance either directly (e.g., training, supervision, or HCP incentives) or indirectly, by changing the physical, economic, or policy environment in which HCPs work (e.g., providing essential medicines, changing user fees, or implementing new health regulations). We excluded studies of strategies without any component directly or indirectly targeting HCPs (e.g., only community education by radio broadcasts). HCPs were broadly defined as hospital-, other health facility-, or community-based health workers; pharmacists; and shopkeepers and informal vendors who sell medicines. We excluded studies of traditional healers who were not part of a well-defined program to implement standards of care based on “Western” or allopathic/osteopathic medical principles. LMICs were countries with a low, lower-middle, or upper-middle income economy, as defined by the World Bank in 2006 (the year we began the literature search) [62]. Studies from both the public and private sector were eligible. We included studies on any health condition, written in any language. We included results only for the primary study outcomes defined by the study authors, or if authors did not designate any outcomes as primary, we defined primary outcomes based on the study objectives (which sometimes meant including all outcomes). There were no restrictions on types of study outcomes (e.g., health facility characteristics; HCP knowledge, attitudes, and practices; patient behaviors and health outcomes; and cost). However, we excluded outcomes with trends that were difficult to interpret in the context of a given study (e.g., “percent of time spent performing curative care” when the strategy did not specifically aim to increase time spent on curative care).

Eligible study designs included pre- versus post-intervention studies with a randomized or non-randomized comparison group, post-intervention only studies with a randomized comparison group, and ITS with at least three data points before and after the intervention. Studies needed at least one primary outcome based on an eligible study design. For example, a pre- versus post-intervention study with a non-randomized comparison group would be excluded if the primary outcomes were only measured at follow-up. We excluded outcomes if HCP performance was “perfect” for the outcome at both baseline and follow-up in the intervention group (e.g., baseline and follow-up values of 100% for “percent of patients correctly treated”). Similarly, for outcomes on HCP practices expressed as a percentage, we excluded effect sizes if the baseline value was 95% or greater, as there was so little room for improvement that effect sizes would be constrained to be small. For outcomes expressed as a percentage, we only included data points based on at least 20 observations per study group and time point. We excluded outcome measures that were not taken at comparable follow-up times between study groups. For ITS, we excluded study outcomes for which the baseline time series was highly unstable and thus could not be reliably modeled, and we excluded outlier outcome measures that probably did not represent the true trend in HCP performance (e.g., an unusually high baseline measure just before a strategy was implemented that was likely due to HCPs’ anticipation of the strategy).

### Literature search

The literature search strategy had six components. First, we searched 15 electronic databases: Campbell Collaboration, Cumulative Index to Nursing & Allied Health Literature (CINAHL), Cochrane Library (which includes the Database of Abstracts of Review of Effects [DARE] and the Cochrane Central Register of Controlled Trials [CENTRAL]), Dissertation Abstracts (for theses and dissertations), EconLit, Eldis, EMBASE, the Effective Practice and Organisation of Care (EPOC) specialized register, Education Resources Information Center (ERIC), Global Health, The Healthcare Management Information Consortium (HMIC), MEDLINE, Science Citation Index (SCI), Sociological Abstracts, and Social Sciences Citation Index (SSCI). The search strategy was based on that used by the International Network for the Rational Use of Drugs (INRUD) [50,63]. These databases were searched in groups in May 2006, September 2006, and May 2007 and went back in time as far as the databases allowed. Second, we searched our personal libraries and asked eleven colleagues for references and unpublished studies. Third, we searched document inventories and websites of 30 organizations involved with HCP performance (Box 1). This component of the search was done primarily between January 2006 and October 2008, and one website was searched in April 2010. Fourth, we performed a hand search of bibliographies from 510 previous reviews and other articles. Fifth, after being contacted to answer questions concerning their studies, 17 authors of studies that were included in the review sent additional, new reports related to their studies. Sixth, after reading an included report that lacked many basic details (e.g., a short presentation at a scientific conference), data abstractors searched the Internet for supplemental articles that could be abstracted along with that report. Details of the literature search strategy are provided in the study protocol (S2 File).

**Box 1. Thirty organizations whose document inventories and websites were searched.**

Basic Support for Institutionalizing Child Survival (BASICS); Capacity Project; U.S. Centers for Disease Control and Prevention; Center for Global Development; CORE group; Danish International Development Agency; U.K. Department for International Development; EngenderHealth; Global Alliance for Vaccines and Immunization; Global Fund to Fight AIDS, Tuberculosis, and Malaria; HealthNet TPO; Human Resources for Health Resource Center; International Conference on Social Health Insurance in Developing Countries (Berlin, December 2005); International Conference on Improving Use of Medicines (ICIUM) 1997 and 2004 conference proceedings; Institute for Healthcare Improvement; WHO/INRUD database [50]; JHPIEGO; Management Sciences for Health; Pan American Health Organization; Partners in Health; PHRPlus; Population Council; PRIME II Project; Partnership for Social Science in Malaria Control; Quality Assurance Project; Safe Injection Global Network; United Nations Children’s Fund (UNICEF); U.S. Agency for International Development (USAID DEC); World Bank; and WHO.

### Screening search results and data abstraction

**Fig 1:**
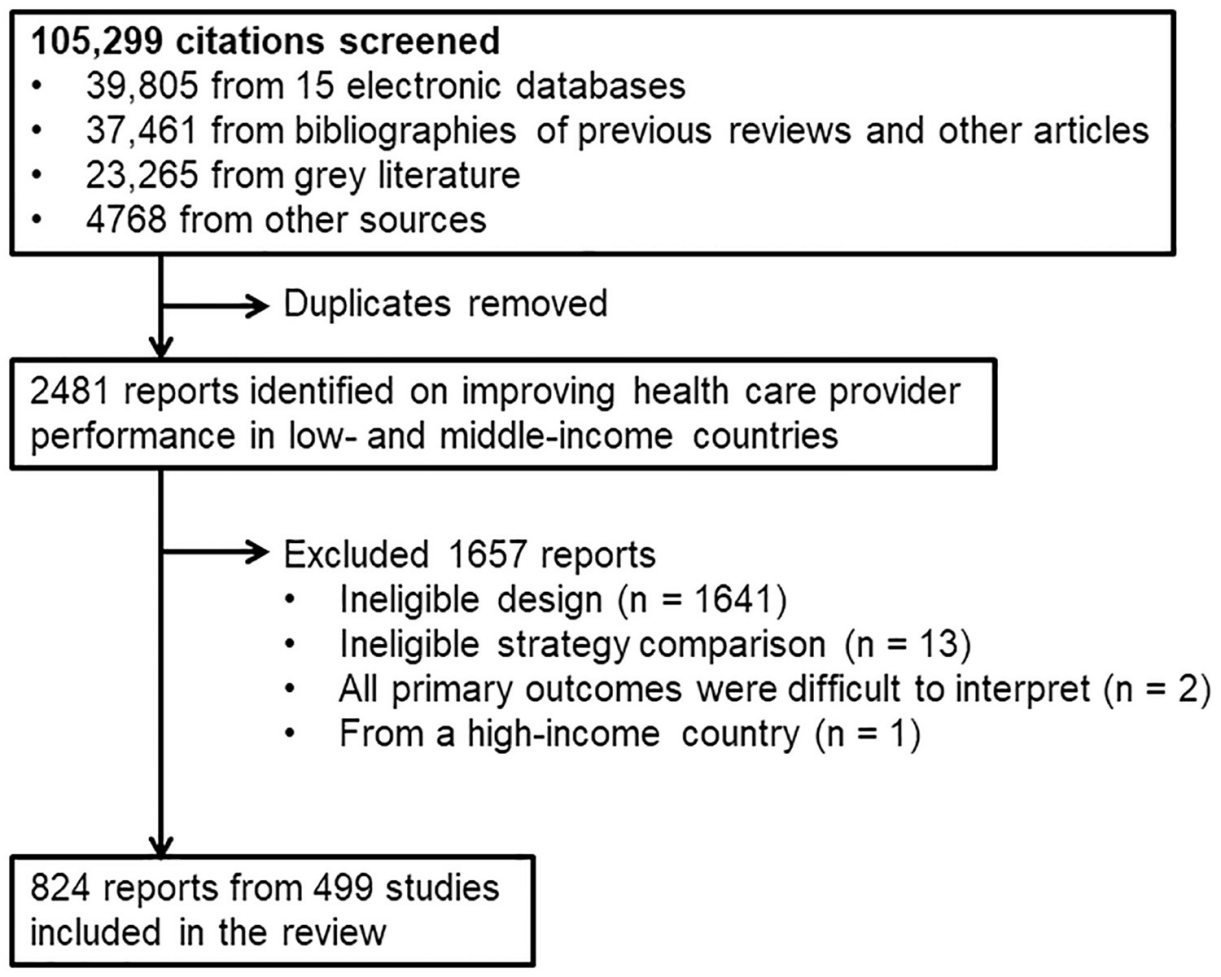
Summary flowchart of the literature search.

Search results were screened and data were abstracted by a team of investigators and trained research assistants. Before beginning, concordance testing was conducted against a “gold standard” list of reports until at least 80% could be identified by each team member. Titles and abstracts from the literature search were reviewed to identify potentially eligible reports. If the title or abstract was insufficient, a full text version was obtained. Full texts of potentially eligible reports were reviewed to identify those that met the inclusion criteria. An investigator (SYR) double-checked all decisions made by the research assistants about which reports would be included. During data abstraction, 16 reports were found to be ineligible and subsequently excluded (last three bulleted items in Fig 1).

Before beginning data abstraction, concordance testing of all team members was conducted until the percent agreement between individual abstractors and a gold standard set of abstracted data (based on consensus by several investigators) was >80%. We also assessed concordance for “paired abstraction” in which two reviewers independently abstracted data and then discussed and resolved discrepancies (concordance = percent agreement between paired abstraction and the gold standard); the mean concordance was 90.8%.

Data were abstracted independently by two team members using a standardized form (Annex 3 of S2 File). The form was an expanded version of that used by INRUD [50,63]. Discrepancies were resolved through discussion and, if needed, consultation with a third data abstractor. Members of the data abstraction team met about once a month for ongoing refresher training and to discuss and resolve data abstraction difficulties. Data were entered into a computer database (Microsoft Access, Microsoft, Inc., Redmond, Washington), and both abstractors for any given study had to confirm that data were entered accurately. Data elements included details on study location and timing, setting where services were delivered, HCP type, strategies to improve performance, study design, sample size, outcomes, effect sizes, risk of bias domains, and cost or economic evaluations. Risk of bias domains were adapted from Grimshaw et al. [43]. When a study report did not include a needed data element or when information was unclear, we made at least four attempts to contact study authors.

For crossover trials, although the analysis typically includes post-intervention data from before and after the crossover of strategies, we only considered post-intervention data before the crossover. We reasoned that post-crossover data were likely to be biased due to exposure to the strategies implemented before the crossover.

We split a small number of studies into “sub-studies” such that the effect sizes in each sub-study corresponded to a different strategy (Box 2).

**Box 2. Scenarios in which studies were split into sub-studies.**

1. When distinct strategy components in a single study group were implemented with observations between components’ implementation (e.g., one sub-study examines the effect of training only, and another sub-study examines the combined effect of training and supervision)
2. When two intervention groups had a different timing of strategies and have observations between components’ implementation: one sub-study that evaluates the impact of a strategy compared to a non-intervention control (i.e., before a strategy is introduced to an intervention group, it serves as a non-intervention control for the other intervention group), and a second sub-study that evaluates the marginal impact of one strategy over another (a head-to-head comparison). For example, one sub-study examines the effect of training only, and another sub-study examines the marginal effect of adding supervision to training.
3. When a strategy involved health facility- and community-level components that were implemented and evaluated separately over time (e.g., facility components implemented and evaluated in study years 1–2 and community components implemented in study years 3–5 and evaluated during all study years [81]), with separate outcomes measured at the facility and community levels: one sub-study that evaluates the effect of facility-level components on facility-level outcomes, and a second sub-study that evaluates the effect of both facility- and community-level components on community-level outcomes.

### Assessment of risk of bias

Our method was based on guidance from the Cochrane EPOC Group [64]. Risk of bias at the study level was categorized as low, moderate, high, or very high (S3 File). Randomized studies, ITS, and non-randomized studies were initially categorized as low, moderate, and high risk of bias, respectively. We then assessed the following domains: number of clusters per study arm, dataset completeness, balance in baseline outcome measurements, balance in baseline characteristics, outcome reliability, adequacy of concealment of allocation, intervention likelihood of affecting data collection, intervention independence from other changes, and number of data points before and after the intervention. Some domains only applied to certain study designs. A study’s risk of bias category was reduced by one level for every applicable domain that was “not done” and for every two applicable domains that were “unclear”. Once a study’s category was “very high”, additional domains that were not done or unclear did not change the category (i.e., there was no category below very high risk of bias). Separate analyses were conducted for all studies and for studies with a low or moderate risk of bias.

### Estimating effect sizes

The primary outcome measure was the effect size, which was defined as an absolute percentage-point difference and calculated such that positive values indicate improvement (S3 File). For study outcomes designed to decrease (e.g., percent of patients receiving unnecessary treatments), we multiplied effect sizes by –1.

For non-ITS studies, effect sizes were based on the baseline value closest in time to the beginning of the strategy and the follow-up value furthest in time from the beginning of the strategy. In non-ITS studies, for outcomes that were dichotomous, percentages, or a bounded continuous outcome that could be logically converted to a percentage (e.g., a performance score ranging from 0–12), the effect size was calculated with Eq 1.

$$effect\;size={\left(followup-baseline\right)}_{intervention}-{\left(followup-baseline\right)}_{control}$$ (1)

In non-ITS studies, for unbounded continuous outcomes, so that the scale of the effect size is a percentage-point change, the effect size was calculated with Eq 2.

$$effect\;size=100\%\left[{\left(\frac{followup-baseline}{baseline}\right)}_{intervention}-{\left(\frac{followup-baseline}{baseline}\right)}_{control}\right]$$ (2)

Separate analyses were performed for the small number of continuous outcomes with a baseline value of zero, which caused the effect size to be undefined.

For ITS studies, segmented linear regression modeling [65] was performed to estimate a summary effect size that incorporated both the level and trend effects. The summary effect size was the outcome level at the mid-point of the follow-up period as predicted by the regression model minus a predicted counterfactual value that equaled the outcome level based on the pre-intervention trend extended to the mid-point of the follow-up period (S3 File). This summary effect size was used because it allowed the results of ITS studies to be combined with those of non-ITS studies.

### Analysis overview

To achieve the HCPPR’s objective of developing evidence-based guidance on improving HCP performance, three analytic steps were required.

1. Define a series of mutually exclusive strategy groups and categorize each strategy into one strategy group.
2. Determine which studies and which results can be meaningfully compared, and to which settings the results can be generalized.
3. Within the groups of results that can be compared: estimate the effectiveness of the strategy groups, assess the quality of the evidence on effectiveness, and make comparisons among strategies in a way that accounts for or reduces bias from outliers, small numbers of studies per strategy, unequal sample sizes, methodological and contextual differences among the studies, and comparison type (intervention versus control, and head-to-head).

#### Step 1: Defining strategy groups

To define a series of mutually exclusive strategy groups and categorize each strategy into one strategy group, we first coded the presence of 194 detailed strategy components for each study arm exposed to an improvement strategy. Next, we grouped the detailed strategy components into 10 component categories (Box 3 and S4 File). We defined a “unique strategy group” as any unique combination of the 10 component categories. The 10 component categories were not specified *a priori* in the review’s protocol. However, the definitions were developed based on conceptual considerations (i.e., which strategy components seemed similar in terms of method, target population, mechanism of action, and in the case of training, the intensity of the training) and not based on effect sizes. The 10 component categories can be disaggregated for future analyses at a more granular level.

**Box 3. Definitions of strategy components categories.^a^**

1. Patient and community support. E.g., community health education, social marketing of health services, and cash transfers to community members.
2. Printed or electronic information (including job aids) for HCPs that is not an integral part of another component. Other strategy components (especially training) often include printed information for HCPs; and in these cases, the printed information was not considered a separate component. As the name suggests, this category includes printed or electronic information for HCPs when it is not an integral part of another component. E.g., a strategy that only consists of distributing pamphlets to HCPs.
3. High-intensity training. Defined as training with a duration greater than 5 days (or ongoing training) and at least one interactive educational method (i.e., clinical practice, role play, or interactive sessions). This category includes academic detailing (i.e., one-on-one training by an opinion leader).
4. Low-intensity training. Any training that was not categorized as high-intensity training (above). This category includes the informal education of HCPs by their peers.
5. Supervision. E.g., improving routine supervision, benchmarking, audit with feedback, peer review, and HCP seeking instructions or second opinions from higher-level HCPs.
6. Group problem solving. E.g., continuous quality improvement, improvement collaboratives, and group problem solving with or without formal teams.
7. Other management techniques that do not include group problem solving and supervision (which are separate component categories). For example, HCP group process that is neither training nor group problem solving, group meetings of HCPs and community members, HCP self-assessment, and changes in processes of care to improve utilization of health services.
8. Strengthening infrastructure. E.g., a new information system, repairing health facilities, improved medicine logistics, and provision of drugs or equipment. Rarely (in five studies), a piece of equipment was not counted as a separate “strengthening infrastructure” component when it was an integral part of another strategy and would not be expected to have an independent effect. For example, in a strategy that included community education (coded as a “Patient and community support” component), the provision of microphones and loudspeakers for use with the community education campaign was not considered a separate component (IDNUM 192100001).
9. Financing and incentives. E.g., changing user fees, revolving drug funds, insurance system, contracting-in or contracting out services, and financial or non-financial incentives.
10. Regulation and governance. E.g., standard drug quality requirements, licensing and accreditation schemes, and resource control given to local government or civil society organizations.

^a^ See S4 File for details and Table A7-6 in S7 File for data on the distribution of combinations of these strategy component categories in included studies.

Placebo strategy components were coded as placebos in the review’s database, but were ignored in the analysis. For example, control groups that were exposed to a placebo strategy (e.g., training on herbal medicine) were analyzed together with control groups that received no new intervention. Note that we describe control groups as receiving “no new intervention” because all HCPs are constantly exposed to pre-existing or “business as usual” interventions (e.g., routine supervision and provision of medical supplies).

#### Step 2: Determining which results can be compared

To determine which results can be compared, four attributes were used: study type, outcome type, outcome scale, and HCP cadre. We first distinguished between non-inferiority studies (i.e., studies that test if a novel strategy is not less effective than an alternative strategy that is typically known to be effective) with gold standard HCPs in the control group (e.g., a study to determine if trained nurses in the intervention group could perform vasectomies as well as physicians in the control group) and all other studies (e.g., a study of in-service training, with a control group of HCPs without the training). These study types were analyzed separately because a successful result of the first study type is an effect size close to zero, while a successful result of the second study type is typically non-zero. For each study type, we categorized effect sizes into 24 subgroups (Table 1), according to six outcome categories (e.g., processes of care, health outcomes, etc.), two outcome scales (percentages and other continuous outcomes), and two HCP cadres (facility-based HCPs and lay health workers). Comparisons are only made within these subgroups and not between them (i.e., between any two cells in Table 1). The outcome categories and HCP cadres can be disaggregated in future analyses to obtain results at a more granular level.

**Table 1:** Number of studies, comparisons, and effect sizes for each outcome category^a^.

General outcome category	Percentage outcomes		Continuous outcomes		Total
	Predominantly health facility-based HCPs^b^	Predominantly lay health workers^c^	Predominantly health facility-based HCPs^b^	Predominantly lay health workers^c^	
1. Elements that facilitate correct HCP performance: availability of supplies and equipment; supervision; and HCP knowledge, attitudes, and satisfaction	52 S	7 S	12 S	2 S	70 S
	77 C	7 C	16 C	2 C	99 C
	318 ES	74 ES	27 ES^d^	4 ES	423 ES
2. Processes of care (or HCP practice outcomes): patient assessment, diagnosis, treatment, chemoprophylaxis, vaccination, counseling and communication, referral, consultation time, and HCP documentation	182 S	10 S	69 S	3 S	220 S
	255 C	15 C	95 C	8 C	307 C
	1208 ES^e^	43 ES	153 ES^f^	8 ES	1412 ES
3. Patient health outcomes: morbidity and mortality	47 S	20 S	60 S	24 S	128 S
	58 C	26 C	78 C	30 C	163 C
	130 ES^g^	64 ES	185 ES	76 ES	455 ES
4. Patient care-seeking/utilization of health services	53 S	17 S	62 S	5 S	120 S
	75 C	19 C	88 C	7 C	165 C
	249 ES	68 ES	166 ES^h^	12 ES	495 ES
5. Patient non-health outcomes: patient or caretaker knowledge, healthy behaviors, patient and community attitudes, patient satisfaction	95 S	38 S	25 S	12 S	147 S
	144 C	45 C	42 C	14 C	212 C
	647 ES	287 ES	58 ES	29 ES	1021 ES
6. Cost	7 S	1 S	51 S	2 S	56 S
	16 C	3 C	63 C	2 C	77 C
	18 ES	3 ES	114 ES	2 ES	137 ES
Total (all general categories combined)	306 S	69 S	219 S	41 S	499 S
	444 C	85 C	303 C	56 C	687 C
	2570 ES	539 ES	703 ES	131 ES	3943 ES^i^

C = Comparison, ES = effect size, HCP = health care provider, S = study.

^a^ See Table A7-7 in S7 File for more detail on the number effect sizes, comparisons, and studies stratified by comparison type (i.e., strategy-versus-control and strategy-versus-strategy).

^b^ Studies of physicians, nurses, midwives, and other HCPs that typically work in a health facility. Studies in this group could include lay health workers, but other HCPs are also exposed to improvement strategies.

^c^ Studies for which improving lay health worker performance is the primary focus. The context might include other HCPs (e.g., village lay health workers might refer seriously ill patients to nurses), but improving the performance of these other HCPs is not the study focus.

^d^ Three effect sizes from one comparison in one study had a divide-by-zero issue (i.e., a baseline value of zero that caused the relative effect size to be undefined, which required an alternative effect size calculation of the intervention follow-up value minus the control follow-up value, and which required analyzing these effect sizes separately from the large majority of effect sizes with no divide-by-zero issue). The remaining 24 effect sizes from 16 comparisons in 12 studies had no divide-by-zero issue.

^e^ One effect size in one comparison came from a non-inferiority study. This effect size must be analyzed separately from the other 1207 effect sizes (from 254 comparisons in 181 studies) that came from studies designed to identify a difference between study arms.

^f^ Two effect sizes from one comparison in one study had a divide-by-zero issue (see footnote c, above). The remaining 151 effect sizes from 95 comparisons in 69 studies had no divide-by-zero issue.

^g^ Two effect sizes in two comparisons came from two non-inferiority studies. These effect sizes must be analyzed separately from the other 128 effect sizes (from 56 comparisons in 45 studies) that came from studies designed to identify a difference between study arms.

^h^ One effect size from one comparison in one study had a divide-by-zero issue (see footnote c, above). The remaining 165 effect sizes from 88 comparisons in 62 studies had no divide-by-zero issue.

^i^ The median number of effect sizes per comparison = 3 (range: 1–102, interquartile range: 1–7).

#### Step 3: Estimate strategy effectiveness, assess evidence quality, and compare strategies

To estimate strategy effectiveness from a single study comparison (e.g., a comparison of two study arms), the effect size was defined as the median of all effect sizes in the comparison for outcomes in the same outcome group (i.e., in the same cell in Table 1). Median effect sizes, which have been used in other systematic reviews [18,66], simplify the analysis (i.e., one effect size per comparison) and reduce the influence of outliers.

Several methods were used to estimate strategy effectiveness from multiple studies and make comparisons in ways that account for or reduce bias from outliers, small numbers of studies per strategy, unequal sample sizes, methodological and contextual differences among the studies, and comparison type (intervention versus control, and head-to-head). These methods, which include comparisons of medians, meta-analysis, and network meta-analysis [67], are described in other reports in preparation.

To assess the quality of the evidence on the effectiveness of each strategy, the Grading of Recommendations Assessment, Development, and Evaluation (GRADE) system was used [68]. To identify publication bias, we examined results for studies of all strategies in a particular outcome group with at least 10 comparisons per strategy. We inspected funnel plots and used Egger’s test of asymmetry (significance of p < 0.1) [69]. We used I^2^ as a measure of consistency for each meta-analysis.

We performed four pre-specified sensitivity analyses. First, we analyzed only studies with a low or moderate risk of bias. Second, we analyzed strategies that included training or supervision to identify factors associated with greater effectiveness. Third, for strategies with large effect sizes, we examined whether the large effect sizes could be due to limited contextual variability. This analysis involved broadening the definition of unique strategy groups to include strategies with the same core components but with other components allowed. Fourth, to better characterize the contexts in which a strategy might be more or less effective, we stratified results according to the level of resources and development where the study was conducted.

### Time trends

To assess time trends in study attributes, we defined the time for a given study as the mid-point year between when data collection began and ended. We used this measure of time rather than publication year because results were often presented in multiple reports or with varying length of delay in publication. Time trends in the odds of studies having a particular attribute per year were assessed using logistic regression. Time trends in the number of studies having a particular attribute per year were assessed with a Poisson regression model or with a negative binomial regression model if over-dispersion was present. Goodness-of-fit was assessed with a chi-squared test of deviance, with a p-value > 0.05 indicating adequate model fit. For analyses of the number of studies per year, studies with a data collection mid-point after 2003 were excluded because such studies were unlikely to be representative of all research done after that time due to publication lag. Unless otherwise specified, analyses were performed with SAS version 9.3 (SAS Institute Inc., Cary, North Carolina). Hypothesis testing was done with an alpha level of 0.05.

## Results

### Literature search

Altogether, we screened 105,299 citations. The search of 15 electronic databases in May 2006 yielded 39,805 citations (Fig 1 and S5 File). An evaluation of the search strategy revealed that of 84 “gold standard” studies that were previously identified as meeting the inclusion criteria, 68 were identified by the literature search (sensitivity = 68/84, or 81.0%). The search of grey literature, which was conducted from January 2006 to October 2008, yielded 23,265 titles. The search of bibliographies of the 510 previous reviews and other articles yielded 37,461 citations. The remaining search methods identified 4768 titles. After removing duplicate citations, screening of the titles and abstracts of the 105,299 citations yielded 2481 potentially eligible reports. Screening of the full text of these reports identified 824 eligible reports for data abstraction. Of the 2481 potentially eligible reports, 1657 were excluded due to: ineligible study design (n = 1641), ineligible study comparison such as a community-only intervention versus a control group (n = 13), all primary outcomes were difficult to interpret (n = 2), or the study was from a high-income country (n = 1). The final database included 824 reports, which contained data from 499 studies.

Of the 499 studies included, we had 456 “non-split” studies, 13 studies that were split into two sub-studies each, one that was split into three sub-studies (IDNUM 135890101–135890103), and one that was split into 14 sub-studies (IDNUM 246490101–246490602). Of the 824 reports, 540 (65.5%) were published in scientific journals. Data abstraction involved personal communications from authors in 53.3% (266/499) of studies. Thus, the HCPPR database contains more information than what is in the original reports, although the database in no way replaces the reports.

### Study attributes

The 499 studies in the review represent a wide diversity of methodologies, geographic settings, HCP types, work environments, and health conditions (see S6 File for study details and http://www.hcpperformancereview.org/download-databases for the database). Altogether, there were 687 comparisons among 996 study arms (Table 2). Two-thirds (453/687, or 65.9%) of the comparisons evaluated a strategy versus a true control group (i.e., no new intervention), one-third (225/687, or 32.8%) were head-to-head comparisons, and a few (9/687, or 1.3%) were strategy versus placebo control group comparisons. There were 3943 effect sizes, with a median of 3 effect sizes per comparison (range: 1–102). Among all 499 studies, 173 (34.7%) were pre- versus post-intervention studies with a non-randomized comparison group, 140 (28.1%) were pre- versus post-intervention studies with a randomized comparison group, 122 (24.4%) were ITS, and 64 (12.8%) were post-intervention only studies with a randomized comparison group. Altogether, 42.3% (211/499) of studies had a randomized design.

**Table 2:** General study attributes.

Study attribute	All studies (N = 499)
Number of study arms	
1	95 (19.0%)
2	332 (66.5%)
3	52 (10.4%)
4	19 (3.8%)
5	1 (0.2%)
Total number of study arms across all studies	996
Total number of comparisons across all studies	687
Strategy versus true (no new intervention) control group	453 (65.9%)
Strategy versus placebo control group	9 (1.3%)
Strategy versus strategy (head-to-head comparison)	225 (32.8%)
Total number of effect sizes across all studies	3943
Median number of effect sizes per study (range)	4 (1–171)
Median number of effect sizes per comparison (range)	3 (1–102)
Study designs	
Pre-post study with non-randomized controls	173 (34.7%)
Pre-post study with randomized controls	140 (28.1%)
Interrupted time series with no controls	101 (20.2%)
Post-only study with randomized controls	64 (12.8%)
Interrupted time series with non-randomized controls	14 (2.8%)
Interrupted time series with randomized controls	7 (1.4%)
Country income classification (World Bank)	
Low income	260 (52.1%)
Lower-middle income	164 (32.9%)
Upper-middle income	66 (13.2%)
Mixture of lower-middle and upper-middle income	6 (1.2%)
Mixture of low and lower-middle income	3 (0.6%)
Risk of bias category^a^	
Low	66 (13.2%)
Moderate	102 (20.4%)
High	158 (31.7%)
Very high	173 (34.7%)
WHO region where study was conducted^b^	
Africa	186 (37.3%)
Southeast Asia	139 (27.9%)
America	79 (15.8%)
Western Pacific	49 (9.8%)
Eastern Mediterranean	31 (6.2%)
Europe	20 (4.0%)
Multiple	5 (1.0%)
Year of publication^c^ (or date of document for unpublished reports), by decade	
2010 or later (latest year was 2011)^d^	16 (3.2%)
2000–2009	273 (54.7%)
1990–1999	169 (33.9%)
1980–1989	26 (5.2%)
Before 1980 (earliest year was 1963)	13 (2.6%)
Unclear or not stated	2 (0.4%)
Data collection methods (multiple responses allowed per study)	
Record or chart review	314 (62.9%)
Interview with patient or patient’s caretaker	227 (45.5%)
Physical exam of patient	65 (13.0%)
Interview with HCP	45 (9.0%)
Observation of HCP-patient interaction	44 (8.8%)
Questionnaire for HCP (any administration method)	42 (8.4%)
Simulated client	24 (4.8%)
Observation of facility	18 (3.6%)
Questionnaire for patient or patient’s caretaker	14 (2.8%)
Observation of patient’s behaviors	9 (1.8%)
Case scenario	8 (1.6%)
Examination for HCP	6 (1.2%)
Observation of HCP practices not involving real patients	6 (1.2%)
Interview with administrator	4 (0.8%)
Self-assessment	3 (0.6%)
Observation of patient’s home	3 (0.6%)
Questionnaire for administrator	2 (0.4%)
Urban versus rural study setting	
Rural areas only	163 (32.7%)
Urban areas with or without peri-urban areas	144 (28.9%)
Mix of urban and rural areas	101 (20.2%)
Town with or without rural areas	29 (5.8%)
Peri-urban areas only	20 (4.0%)
Unclear or not stated	42 (8.4%)

^a^ See S3 File for details on the definitions of the risk of bias categories.

^b^ The number of countries sum to more than 499 because the five multi-region studies are both shown as a separate category and combined in the region-specific counts. For example, for the Africa region, 183 studies were conducted only in Africa, and three additional studies were each conducted in multiple regions (e.g., one of the three additional studies was conducted in both the Africa and Southeast Asia regions); thus the total for the Africa region is 186.

^c^ For studies represented by multiple reports, year of publication is the year of publication for the report that was first identified in our review.

^d^ These were studies that were originally identified as unpublished, but were published by the time of the data abstraction.

The proportion of studies categorized as having a low, moderate, high, and very high risk of bias were 13.2%, 20.4%, 31.7%, and 34.7%, respectively (Table 2). Results for individual risk-of-bias domains are presented in S7 File (Table A7-2). For the 326 studies that used a randomized or ITS design (with an initial risk-of-bias classification of low or moderate, respectively), the main deficiencies in risk-of-bias domains that caused a drop in the final risk of bias classification were: imbalance in baseline outcome measurements or contextual characteristics between study arms, and having a small number of clusters (three or less) per study arm (for randomized studies); and intervention not being independent of other changes, and fewer than six measures before or after the intervention (for ITS studies) (S7 File, Table A7-5). We found no association between study quality (in terms of risk of bias) and whether a study was published in a scientific journal (p = 0.27) (Table 3).

**Table 3:** Number of studies stratified by publication status and risk of bias category^a^.

Risk of bias category	Publication status		Total^b^
	At least one study report published in scientific journal No. (column %)	No study reports published in scientific journal No. (column %)	
Low	58 (14.1)	8 (9.1)	66
Moderate	86 (20.9)	16 (18.2)	102
High	125 (30.4)	33 (37.5)	158
Very high	142 (34.5)	31 (35.2)	173
Total	411 (100)	88 (100)	499

^a^ The Cochran–Mantel–Haenszel test of whether the mean risk of bias score differs by publication status yields a p-value of 0.27. If the low and moderate categories are combined, and the high and very high categories are combined, the Cochran–Mantel–Haenszel row mean test between publication status and the 2-level risk of bias category yields a p-value of 0.16.

^b^ The percentages of low, moderate, high, and very high risk of bias studies with at least one report published in a scientific journal were 87.9% (58/66), 84.3% (86/102), 79.1% (125/158), and 82.1% (142/173), respectively.

The 499 studies were conducted in 79 different LMICs, and about half (260/499, or 52.1%) were from low-income countries (Table 2). About one-third of studies (186/499, or 37.3%) were conducted in the Africa WHO region, 37.7% in Asia (Southeast Asia and Western Pacific regions), 15.8% in the Americas, and 10.2% in other regions. One-third of studies (163/499, or 32.7%) were conducted only in rural areas, 32.9% (164/499) were only from urban or peri-urban areas, and 26.0% (130/499) were from mixed settings. Numerous data collection methods were used, with the most common being record review (62.9% of studies) and patient interviews (45.5%).

The most common places where services were delivered were outpatient health facilities, in 52.7% (263/499) of studies; community settings, including HCPs’ own homes (35.7%); hospital outpatient departments (32.5%); and hospital and health facility inpatient wards (23.4%) (Table 4). Notably, 40 studies involved pharmacies, and 21 were in other drug shops. Studies often mentioned multiple service delivery locations. Ownership of the places where services were delivered was most often the government, in 62.7% (313/499) of studies, and the private sector (23.2%).

**Table 4:** Study setting: Places where services were delivered, who owns or operates the service delivery points, and types of health care providers.

Study attribute	All studies (N = 499)
Places where services were delivered (multiple responses allowed)	
Outpatient health facility	263 (52.7%)
Household or community setting	178 (35.7%)
Hospital outpatient department	162 (32.5%)
Hospital inpatient wards^a^	114 (22.9%)
School	42 (8.4%)
Pharmacy	40 (8.0%)
Drug shop	21 (4.2%)
Other outpatient setting	10 (2.0%)
Laboratory	6 (1.2%)
Non-hospital health facility inpatient ward^a^	6 (1.2%)
Brothel or sex establishment	3 (0.6%)
In transit (e.g., in a vehicle) to hospital or health facility	1 (0.2%)
Not reported	1 (0.2%)
Who owns or operates the place where services were delivered (multiple responses allowed per study)	
Public or government	313 (62.7%)
Private, for profit^b^	53 (10.6%)
Private, not for profit^b^	41 (8.2%)
Private, profit status unknown or not reported^b^	37 (7.4%)
Public-private partnership^b^	8 (1.6%)
Other	25 (5.0%)
Unclear or not reported	39 (7.8%)
Type of health care providers (multiple responses allowed per study)	
Physician	236 (47.3%)
Nurse	195 (39.1%)
Lay health worker	188 (37.7%)
Nursing aide	109 (21.8%)
Midwife	78 (15.6%)
Paramedic or unspecified non-physician	57 (11.4%)
Health educator or information officer	36 (7.2%)
Pharmacist	32 (6.4%)
Clinical officer	24 (4.8%)
Laboratorian	14 (2.8%)
Student	15 (3.0%)
Health care provider, type unspecified	84 (16.8%)
Lay health worker was the predominant type of health care provider in at least one study comparison^c^	90 (18.0%)

^a^ Altogether, 23.4% (117/499) of studies were in any inpatient ward (hospital or non-hospital).

^b^ Altogether, 23.2% (116/499) of studies involved a private sector setting (all types).

^c^ Lay health workers were the predominant provider for all study comparisons in 87 studies and were the predominant provider for at least one (but not all) study comparisons in three studies (in the review’s database, these three studies were classified as non-LHW-predominant.

The review captured studies on a wide array of HCP types, including physicians (in 47.3% of studies), nurses (39.1%), midwives (15.6%), lay health workers (including traditional birth attendants) (37.7%), and pharmacists (6.4%) (Table 4 and S7 File). Lay health workers were the predominant type of HCP in 90 studies. The review also included studies on numerous health conditions, including infectious diseases, non-communicable diseases, pregnancy, and family planning (Table 5). Many studies involved multiple health conditions.

**Table 5:** Health conditions addressed by studies in the review.

Health condition(multiple responses allowed per study)	No. of studies with at least one effect size related to the health condition (N = 499 studies)
Multiple (or all) health conditions	179 (35.9%)
Pregnancy	81 (16.2%)
Reproductive health (not pregnancy related)	67 (13.4%)
Malnutrition	49 (9.8%)
Acute respiratory infections	43 (8.6%)
Diarrhea	43 (8.6%)
HIV/AIDS +\- other sexually transmitted diseases	43 (8.6%)
Malaria	36 (7.2%)
Newborn health conditions	33 (6.6%)
Vaccine-preventable illnesses	30 (6.0%)
Non-communicable diseases (not covered by other categories, such as asthma)	23 (4.6%)
Tuberculosis	17 (3.4%)
Heart disease	14 (2.8%)
Sexually transmitted diseases (HIV/AIDS not specifically included)	12 (2.4%)
Hypertension	12 (2.4%)
Parasitic diseases, excluding malaria	10 (2.0%)
Other infectious diseases (not covered by other categories, such as appendicitis)	9 (1.8%)
Child health (not covered by other categories, such was well-baby checks)	8 (1.6%)
Mental health	6 (1.2%)
Injuries and trauma	4 (0.8%)

**Fig 2:**
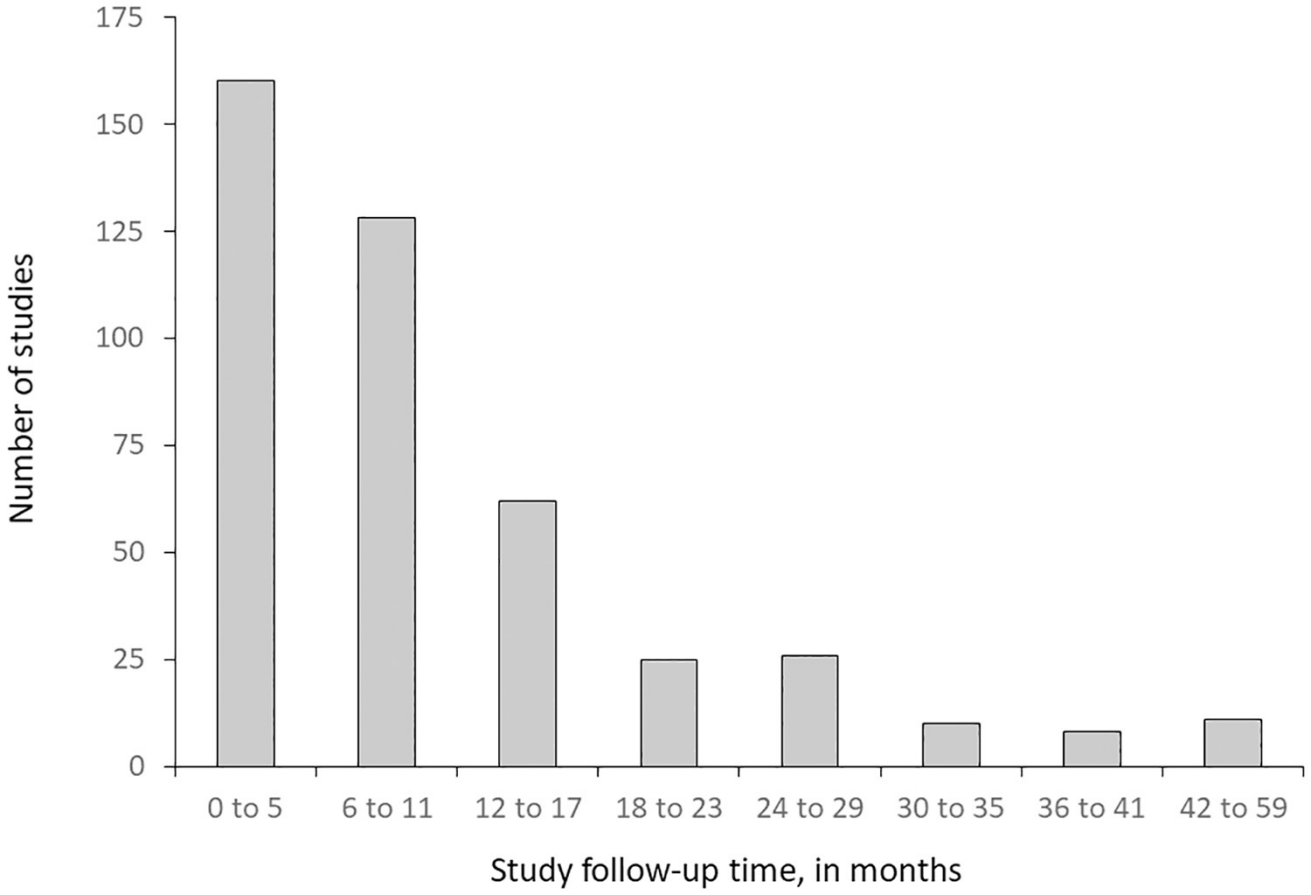
Study follow-up times^a^ for 432 studies that reported study duration. ^a^ Follow-up time for a given study is the median follow-up time for measurement of all primary outcomes in the study.

Among the 432 studies that reported follow-up time, the follow-up duration of many studies was relatively short: less than 6 months for 37.0% of studies, 6–11 months for 29.6% of studies, and 12–59 months for 33.3% (Fig 2). Sixty-seven studies did not report duration.

### Strategies tested

Training, supervision, and patient and community supports were the most commonly evaluated components (Table 6). Altogether, 161 unique strategy groups were tested by studies included in the review (S7 File, Table A7-6). Most (101, or 62.7%) of these strategy groups were tested by only one or two studies each. We identified 490 unique combinations of the 194 detailed strategy components, with 87.1% (427/490) of these combinations tested by only one study each.

**Table 6:** Distribution of strategy components across all intervention arms.

Strategy component	No. (%) of intervention arms that included the strategy component (N = 687 arms)
Low-intensity training (i.e., any training not categorized as high-intensity training); includes informal education of HCPs by their peers	340 (49.5)
Patient or community support (e.g., community health education)	320 (46.6)
Supervision (e.g., improving routine supervision)	286 (41.6)
Strengthening infrastructure (e.g., provision of drugs)	233 (33.9)
Management techniques, excluding group problem solving and supervision (e.g., changing processes of care to improve utilization of health services)	184 (26.8)
High-intensity training (i.e., duration > 5 days or ongoing training or academic detailing; and at least one interactive education method, such as clinical practice, role play, or interactive sessions)	164 (23.9)
Financing and incentives (e.g., changing user fees)	161 (23.4)
Governance or regulation (e.g., accreditation schemes)	121 (17.6)
Group problem solving (e.g., continuous quality improvement)	76 (11.1)
Printed or electronic information or job aid for HCPs that is not an integral part of another component^a^	46 (6.7)

^a^ Other strategy components (especially training) often include printed information for HCPs; and in these cases, the printed information was not considered a separate component. This category includes printed or electronic information for HCPs when the information is not an integral part of another component. For example, a strategy that only consists of distributing pamphlet to HCPs.

### Outcome categories

Many different outcomes were used by studies in the review; a key task was to create a manageable number of outcome categories with enough within-category homogeneity to allow for a meaningful analysis. We first created 23 topic categories, most of which could have outcomes on a percentage or continuous scale (Table 7). Next, we grouped outcomes into six general categories and two outcome scales (Table 1). Individual studies could belong to more than one of the 12 outcome sub-types. Studies were also classified into those targeting primarily health facility-based HCPs, such as physicians and nurses, and those predominantly focused on lay health workers (Tables 1 and 4).

**Table 7:** Categories of all 3943 effect sizes from all 499 included studies.

Outcome	Outcome scale		Total (percentage and continuous combined)
	Percentage	Continuous	
General category 1 (elements that facilitate HCP performance)			
Availability of supplies and equipment	50	9	59
HCP attitudes	77	8	85
HCP knowledge	235	14	249
HCP satisfaction	14	0	14
Supervision	16	0	16
General category 2 (processes of care)			
Assessment	169	5	174
Case management^a^	126	7	133
Chemoprophylaxis	11	1	12
Consultation time	2	13	15
Counseling and communication	258	18	276
Diagnosis	21	17	38
HCP documentation	35	0	35
Referral	37	9	46
Treatment	586	91	677
Vaccination	6	0	6
General category 3 (health outcomes)			
Morbidity	173	153	326
Mortality	21	108	129
General category 4 (care-seeking)			
Patient care-seeking	317	178	495
General category 5 (effects on patients that are neither health outcomes nor care-seeking)			
Patient behavior that is not care-seeking (e.g., compliance with treatment instructions)	514	69	583
Patient or caregiver knowledge	232	8	240
Patient or community attitudes	147	10	157
Patient satisfaction	41	0	41
General category 6 (cost outcomes)			
Cost	21	116	137

^a^ Outcomes that include multiple steps of the case-management pathway (e.g., correct diagnosis and treatment).

### Cost and cost-effectiveness

Of all 499 studies, only 181 (36.3%) reported any information on strategy costs or other economic evaluations. Studies infrequently (108/499, or 21.6%) reported the cost of even one strategy component. Almost one-third of studies (157/499, or 31.5%) compared the strategy costs of two or more study groups, which includes an assumed zero cost for no-intervention control groups. Only 124 studies (24.8%) compared strategy costs of two or more study groups in terms of a cost ratio (e.g., cost per service provided). For studies that did include economic information, many different methods and types of cost or cost-effectiveness data were reported.

### Time trends

**Fig 3:**
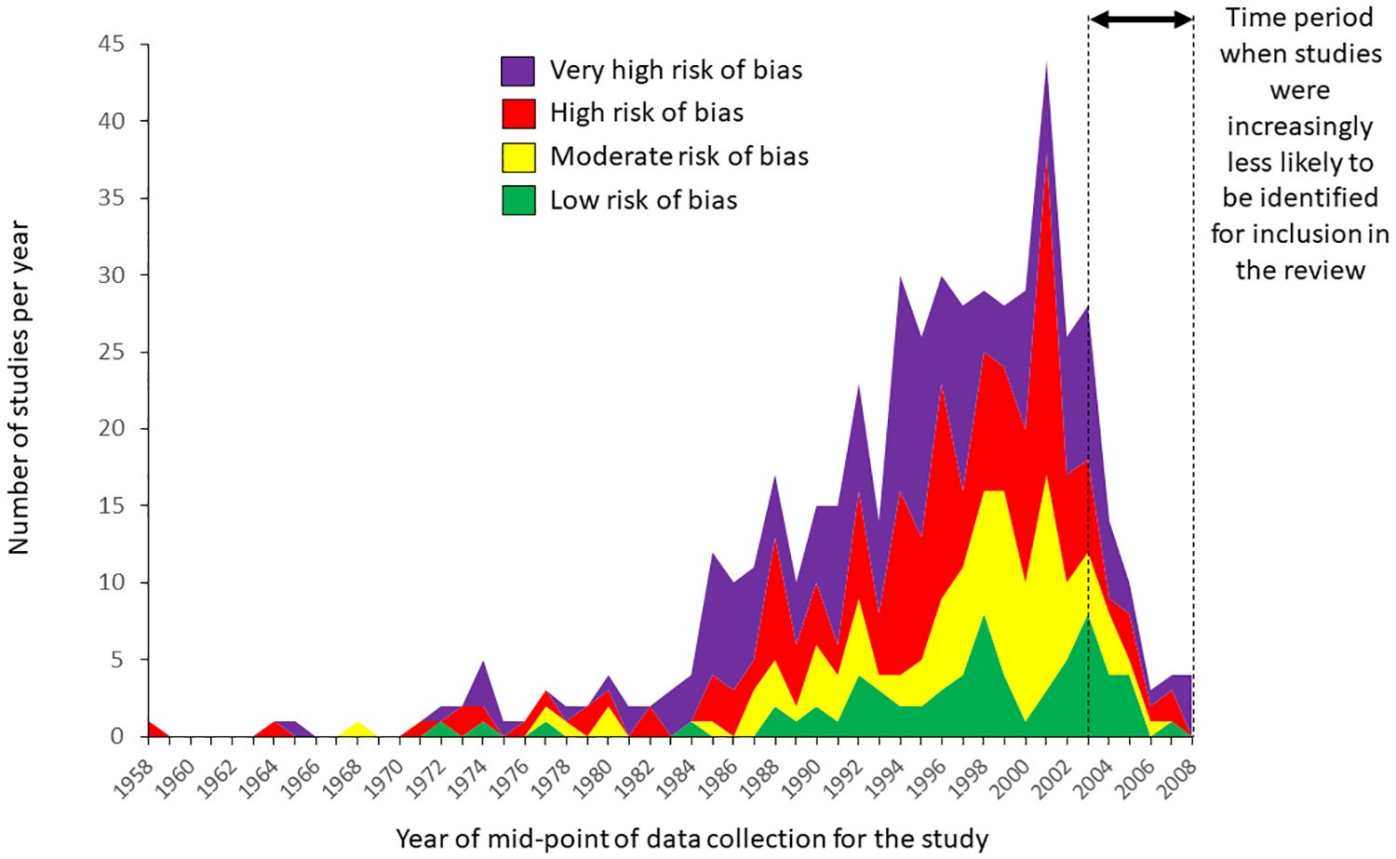
Number and risk of bias of studies with acceptable research designs^a^ over time. ^a^ Study designs eligible for the review included pre- versus post-intervention studies with a randomized or non-randomized comparison group, post-intervention only studies with a randomized comparison group, and interrupted time series with at least three data points before and after the intervention.

The number and quality of studies improved significantly over the time covered by the review, from the late 1950s to the 2000s (Table 8 and Fig 3). The growth in research was so dramatic that the number of studies per year significantly increased for every category of study we examined. Additionally, over time, studies were significantly more likely to be conducted in low-income countries, in Africa, and in private sector settings. Over time, studies were significantly less likely to be conducted in community settings and to be published in a scientific journal. Although the number of studies per year that reported cost or economic data has significantly increased over time, the proportion of studies reporting this information has essentially remained unchanged.

**Table 8:** Time trends in study attributes.

Study attribute	Year of the mid-point of data collection				Percent annual change	P-value of annual change
	1958 to 1979	1980s	1990s	2000s^a^		
*All studies*						
No. of studies	23	75	239	162		
Mean no. of studies per year	1.0	7.5	23.9	31.7	12.2^b^	<0.0001
*Risk of bias*						
Low risk of bias						
No. of studies (% of all studies)	3 (13.0)	4 (5.3)	33 (13.8)	26 (16.1)	4.6^c^	0.029
Mean no. of studies per year	0.1	0.4	3.3	4.2	13.6	<0.0001
Low or moderate risk of bias						
No. of studies (% of all studies)	6 (26.1)	14 (18.7)	84 (35.2)	64 (39.5)	4.7	0.0012
Mean no. of studies per year	0.3	1.4	8.4	12.3	14.2	<0.0001
*Country income classification*, *as defined by the World Bank (2006)*						
Low income						
No. of studies (% of all studies)	8 (34.8)	37 (49.3)	114 (47.7)	104 (64.2)	4.8	0.0002
Mean no. of studies per year	0.4	3.7	11.4	18.3	12.2	<0.0001
Lower-middle income						
No. of studies (% of all studies)	8 (34.8)	30 (40.0)	87 (36.4)	48 (29.6)	–2.6	0.03
Mean no. of studies per year	0.4	3.0	8.7	11.8	11.7	<0.0001
Upper-middle income						
No. of studies (% of all studies)	7 (30.4)	9 (12.0)	42 (17.6)	14 (8.6)	–3.1	0.039
Mean no. of studies per year	0.3	0.9	4.2	2.5	8.9	<0.0001
*Geographic region*, *as defined by the World Health Organization*						
Africa						
No. of studies (% of all studies)	4 (17.4)	24 (32.0)	89 (37.2)	69 (42.6)	4.6	0.0008
Mean no. of studies per year	0.2	2.4	8.9	10.5	12.2	<0.0001
America						
No. of studies (% of all studies)	3 (13.0)	15 (20.0)	46 (19.3)	15 (9.3)	–3.3	0.024
Mean no. of studies per year	0.1	1.5	4.6	3.3	10.7	<0.0001
Eastern Mediterranean						
No. of studies (% of all studies)	2 (8.7)	6 (8.0)	9 (3.8)	16 (9.9)	1.3	0.60
Mean no. of studies per year	0.1	0.6	0.9	3.3	11.8	<0.0001
Europe						
No. of studies (% of all studies)	3 (13.0)	3 (4.0)	13 (5.4)	1 (0.6)	–5.7	0.014
Mean no. of studies per year	0.1	0.3	1.3	0.3	7.0	0.0003
Southeast Asia						
No. of studies (% of all studies)	9 (39.1)	22 (29.3)	56 (23.4)	52 (32.1)	–1.0	0.43
Mean no. of studies per year	0.4	2.2	5.6	12.0	11.0	<0.0001
Western Pacific						
No. of studies (% of all studies)	2 (8.7)	7 (9.3)	28 (11.7)	12 (7.4)	0.1	0.97
Mean no. of studies per year	0.09	0.7	2.8	2.8	11.9	<0.0001
*Ownership of the place where services were delivered*						
Public						
No. of studies (% of all studies)	12 (52.2)	44 (58.7)	142 (59.4)	115 (71.0)	2.4	0.054
Mean no. of studies per year	0.5	4.4	14.2	22.0	12.5	<0.0001
Private^d^						
No. of studies (% of all studies)	1 (4.4)	16 (21.3)	59 (24.7)	40 (24.7)	3.3	0.035
Mean no. of studies per year	0.05	1.6	5.9	7.3	15.2	<0.0001
Community setting						
No. of studies (% of all studies)	18 (78.3)	44 (58.7)	79 (33.1)	49 (30.3)	–6.6	<0.0001
Mean no. of studies per year	0.8	4.4	7.9	10.3	8.3	<0.0001
*Publication in a scientific journal*^e^						
No. of studies (% of all studies)	21 (91.3)	71 (94.7)	194 (81.2)	125 (77.2)	–4.7	0.009
Mean no. of studies per year	1.0	7.1	19.4	24.0	11.3	<0.0001
*Study reported any data on strategy cost or other economic evaluation*						
No. of studies (% of all studies)	6 (26.1)	28 (37.3)	89 (37.2)	58 (35.8)	0.5	0.68
Mean no. of studies per year	0.3	2.8	8.9	11.8	12.0	<0.0001

^a^ For analyses of number of studies per year, this category includes 2000–2003. For analyses of the proportion of studies with a particular attribute, this category includes 2000–2008.

^b^ Annual change in the number of studies per year. For example, between 1958 and 2003, the number of studies per year increased by 12.2% per year.

^c^ Annual change in the odds that a study will have the attribute. For example, between 1958 and 2003, the odds of a study having a low risk of bias increased by 4.6% per year.

^d^ Includes private for profit, private non-profit, private with profit status unknown, and public-private partnership.

^e^ For studies with results in multiple reports, a study was considered published if at least report was published in a scientific journal.

## Discussion

The HCPPR identified an unexpectedly large number of studies that evaluated strategies to improve HCP performance in LMICs. About two-thirds of study reports described studies with study designs that did not meet the criteria for inclusion in the review. There remained a remarkable 499 studies with stronger designs (i.e., controlled studies and ITS), which were included. These studies represent evaluations of a great diversity of strategies to improve HCP performance for numerous health conditions, tested in a wide variety of settings.

While the richness of the evidence base presents a substantial opportunity to understand how best to improve HCP performance in a variety of contexts for many types of quality problems, some key challenges exist. First, risk of bias in the included studies remains a major concern: about two-thirds of studies had a high or very high risk of bias. Second, synthesizing study results was complicated by lack of standardization and missing details on strategy description, outcomes, measurement methods, analysis, and contextual description. Additionally, only about one-third of studies reported any information on strategy cost or cost-effectiveness. Finally, the evidence supporting most strategies is rather thin. Most strategies were evaluated by only one or two comparisons, and one cannot make broad generalizations about such strategies with so little evidence.

### Strengths and limitations

Our review had several notable strengths. It is the largest and most comprehensive systematic review on the topic of HCP performance in LMICs and the first to use network meta-analysis to quantitatively incorporate head-to-head comparisons into analyses of strategy effectiveness. Another strength is the high level of detail collected on strategies, methods, and context, which was used to reduce the bias of strategy-to-strategy comparisons. The availability of the HCPPR database containing all of the detailed data, systematically extracted for the review, allows other researchers to conduct additional studies tailored to their needs, for example, comparing strategies that have been reported from similar geographic or health system contexts, or analyses targeting specific types of health providers or outcomes. Making the review’s database publicly available adheres to a new standard on data sharing in health research [70].

Nonetheless, our review also had several important limitations. First, the included studies themselves often had limitations: missing data elements (e.g., study dates and sample sizes); incomplete descriptions of the strategy, methods, and setting; difficulty in assessing study precision (often because of a failure to adjust clustered data for correlation); and little detail on cost and cost-effectiveness. Fortunately, the authors of 266 studies responded to our queries, and the resulting information was enormously helpful in filling data gaps.

The second main limitation was the challenge of defining strategy groups. As there is no universally recognized taxonomy for strategies to improve HCP performance [71–75], we took a pragmatic approach. We created strategy groups that we thought would be generally understood by program and research audiences, and we tried to balance the requirement of homogeneity within strategy groups with the need of having strategy groups with enough studies to allow for a meaningful analysis. By publicly sharing the HCPPR database, users will not be restricted to using our categorization method. Additionally, despite our aim of creating strategy groups that each included a reasonable number of studies, most strategy groups were only evaluated by one or two studies, which ultimately complicated the analysis and limited our ability to make robust generalizations. These results highlight the importance of developing an agreed-upon taxonomy of strategies, as well as the need for more replication studies of promising strategies (a need seen in other areas of health science [76]).

The third main limitation was the relatively simple approach we took in dealing with the considerable heterogeneity among studies in terms of settings, methods (especially outcomes), and other attributes. How heterogeneity is addressed is critical because it defines which results can be compared and to which settings and HCP types can the results be generalized. Fourth, due to the large number of statistical tests conducted and the retrospective nature of the review, results of statistical testing should be viewed as hypothesis screening, not true hypothesis testing. Fifth, by excluding studies of strategies that only targeted communities, we unintentionally excluded strategies such as direct-to-consumer advertising [77,78] and community education as a stand-alone strategy [79]. Finally, the review is out of date. Novel strategies, such as sending clinical reminders to HCPs via their mobile phone [80], are not represented. However, we are currently updating the review.

## Conclusions

The HCPPR addresses an important gap in our knowledge about the effectiveness and cost of strategies to improve HCP performance in LMICs. Analyses of the studies included in the review’s database that are described in this report will allow program managers, policy analysts, donors, technical agencies, and researchers to identify effective approaches to improve HCP performance tested in a variety of settings, and to choose components that will strengthen future improvement strategies.

## Supporting information

S1 File The Preferred Reporting Items for Systematic Reviews and Meta-Analyses (PRISMA) checklist. (PDF)

S2 File The study protocol. (PDF)

S3 File Detailed methods for assessing risk of bias, calculating effect sizes, coding, and analysis. (PDF)

S4 File Definition of 10 strategy component categories. (PDF)

S5 File Detailed flowchart of the literature search, as recommended by Preferred Reporting Items for Systematic Reviews and Meta-Analyses (PRISMA) guidelines. (PDF)

S6 File Details of the 499 studies included in the review. (XLSX)

S7 File Additional results. (PDF)
