# Supplementary material for: A systematic review of the effectiveness of strategies to improve health care provider performance in low- and middle-income countries: Methods and descriptive results
Source: PLoS One. 2019 May 31;14(5):e0217617. doi: 10.1371/journal.pone.0217617 (PMC6544255; doi:10.1371/journal.pone.0217617)
Supplement: S2 File — (PDF) [file pone.0217617.s002.pdf]

Appendix 2. The protocol of the Health Care Provider Performance Review

Protocol for a systematic review of the effectiveness and costs of interventions to improve health care provider performance and related health outcomes in low- and middle-income countries

Final

I. PROJECT TITLE

A systematic review of the effectiveness and costs of interventions to improve health care provider performance and related health outcomes in low- and middle-income countries

II. ABBREVIATIONS

BASICS	Basic Support for Institutionalizing Child Survival
CDC	Centers for Disease Control and Prevention
DANIDA	Danish International Development Agency
DFID	U.K. Department for International Development
EPOC	Effective Practice and Organization of care
GAVI	Global Alliance for Vaccines and Immunization
HCP	Health care provider
JHPIEGO	John Hopkins International Education for Reproductive Health
LMICs	Low- and middle-income countries
MSH	Management Sciences for Health
OECD	Organization for Economic Cooperation and Development
PSSMC	Partnership for Social Science in Malaria Control
QAP	Quality Assurance Project
QUORUM	Quality of Reporting of Meta-analyses
UNICEF	United Nations Children's Fund
USAID	U.S. Agency for International Development
WHO	World Health Organization

III. BACKGROUND AND JUSTIFICATION

Each year in low- and middle-income countries (LMICs) millions of children and adults die prematurely [Black, 2003; WHO, 2003] when many interventions exist that can prevent such deaths [Jones, 2003; Travis, 2004]. Low coverage of these interventions has been identified as a critical public health problem [Jones, 2003] and a major obstacle to achieving Millennium Development Goals [Travis, 2004].

Health care providers (HCPs)[1] are a key part of almost any strategy for increasing coverage of health interventions, however performance[1] is often inadequate, as documented in studies of child health [Bryce, 2003; Naimoli, 2001; Rowe, 2001], sexually transmitted diseases [Bitera, 2002], family planning, obstetrics [Goldman, 2003], mental disorders [Abas, 2003], injuries [Bickler, 2002], diabetes [Whiting, 2002], malaria [Ofori-Adjei, 1996; Rowe, 2000; Rowe, 2003; Zurovac, 2004], illnesses managed by private sector health workers [Brugha, 1998; Mills, 2002], and medicine use [Hogerzeil, 1993; Ross-Degnan, 1997; WHO, 2001]. Notably, inadequate care occurs despite substantial efforts by governments, donors, and other partners.

[1] See Annex 1 for definitions.

Improving performance is not only important to prevent errors of omission (e.g., a patient needing a medicine does not receive it), but also to avoid harmful practices (e.g., giving sedatives to children with pneumonia [Rowe, 2001]). Furthermore, some research suggests that improving performance may increase utilization of health services [Arifeen, 2004].

Hundreds of studies in LMICs have evaluated a wide variety of strategies to improve HCP performance; and a systematic review that distills the evidence on the effectiveness and costs can be invaluable for guiding policy to reduce medical errors, focusing programmatic efforts on strategies that are effective, and avoiding those that are not. Perhaps the largest published systematic review [Grimshaw, 2004], which did not intend to exclude studies from LMICs, identified only 3 studies from LMICs—although other studies from LMICs exist. Two systematic reviews focused exclusively on interventions to improve medicine use in LMICs [Ross-Degnan, 1997; WHO, 2001]. Two systematic reviews were descriptive, non-quantitative summaries [Siddiqi, 2005; Amaral, 2008]. Several others focused on specific strategies, such as job aids [Knebel, 2000a; Grace, 2008], self-assessment [Bose, 2001], computer-based training [Knebel, 2000b], distance learning [Knebel, 2001], integration of services [Briggs, 2001], telemedicine [Wootton, 2001], and essential drug programs [Ratanawijitrasin, 2001]; however, some of these reviews included a mix of studies from LMICs and industrialized countries. Furthermore, a key limitation of single-strategy reviews is that most studies have evaluated strategies with multiple components (e.g., training and supervision and job aids); and in such studies, one cannot generally tease apart the individual effectiveness of just one of the components.

Although these reviews represent important contributions, an updated review is needed that includes new research and incorporates methodological advances (e.g., quantitative methods for identifying intervention and study characteristics associated with effectiveness [e.g., to answer questions such as: Which types of training interventions are more successful?]).

On a closely related topic, a need exists for a model or theory that describes (and ideally predicts) HCP practices. Several experts have referred to behavioral theories that might apply to HCP practices [Grol, 1997; Grol & Grimshaw, 2003; Ross-Degnan, 1997; Woodward, 2000]; however, this multiplicity of theories leads to a rather fragmented picture, as no single theory seems to describe HCP practices very comprehensively. Furthermore, these theories have not been validated with empirical evidence from studies of HCPs. The justification for developing a theory to describe HCP practices is that it could be used to develop (or refine) interventions that are more likely to be effective in promoting desirable practices. Stated the opposite way, without a reasonably good model, the only way effective interventions will be developed is via intuition or luck. Even a rough "first generation" theory might be useful for stimulating further research and consequent improvement.

Increased funding for disease-specific initiatives (e.g., via the Global Fund to Fight AIDS, Tuberculosis, and Malaria) provides an enormous opportunity to improve health in LMICs, and strengthening HCP performance has the potential to increase the efficiency and effectiveness of such initiatives. More generally, research on improving HCP performance fits within the larger public health priorities of conducting research to strengthen human resources

for health [Chen, 2004; Narasimhan, 2004] and health systems [Alliance for Health Policy and Systems Research, 2004; Task Force on Health Systems Research, 2004].

IV. OBJECTIVES

Conduct a systematic review of the effectiveness and costs of interventions to improve HCP performance and related health outcomes in LMICs, and produce the following.

A. A database of studies on improving HCP performance, which could be used by policy-makers and other researchers
B. A detailed report with fully transparent methods, input data, and results
C. A list of evidence-based policy recommendations (i.e., guidance on how to implement clinical guidelines in LMICs)
D. A summary article for publication in a peer-reviewed journal
E. A research agenda (or a refinement of an existing research agenda) to identify and fill critical knowledge gaps on how to implement clinical guidelines in LMICs
F. A theory describing HCP practices, which combines existing theories (where appropriate) and emphasizes the empirical evidence that supports the theory (where available)

V. INVESTIGATORS AND CONTRIBUTORS

A. Names (in alphabetical order)
    1. Charity Akpala, Johns Hopkins Univ. [abstractor]
    2. Sushama Acharya, CDC [abstractor]
    3. Dinorah Calles, CDC [abstractor]
    4. Tashana Carty, CDC [title screener]
    5. John Chalker, Management Sciences for Health (MSH)
    6. Adrijana Corluka, Johns Hopkins Univ. [abstractor]
    7. Di (Didi) Cross, CDC [abstractor]
    8. Bhavya Doshi, CDC [title screener]
    9. Margaret (Meg) Griffith, CDC [abstractor]
    10. Atsumi Hirose, CDC [abstractor]
    11. Kathy Holloway, WHO [co-investigator]
    12. Qing Li, CDC [abstractor]
    13. Connie Liu, Case Western Reserve Univ. [abstractor]
    14. Earl Long, CDC [title screener]
    15. Jason McKnight, CDC [abstractor]
    16. Dawn Osterholt, Cincinnati Children's Hospital [abstractor]
    17. Amy Patterson, Emory Univ. [abstractor]
    18. David Peters, World Bank [co-investigator]
    19. Gabriel Ponce-de-Léon, CDC [title screener]
    20. SM Atiqur (Atiq) Rahman, CDC [abstractor]
    21. Dennis Ross-Degnan, Harvard University [co-investigator]
    22. Alexander Rowe, CDC [primary investigator]
    23. Samantha Rowe, CDC [co-investigator]

24. Nirali Shah, Johns Hopkins Univ. [abstractor]
25. Banafsheh Siadat, World Bank [administrative coordinator]
26. Savitha Subramanian, World Bank [administrative coordinator and title screener]
27. Anilkrishna (Anil) Thota, CDC [abstractor]
28. Jeff Willis, Johns Hopkins Univ. [abstractor]
29. Shannon Wood, CDC [title screener]
30. Karen Wosje, Cincinnati Children's Hospital [abstractor]
31. Chunying Xie, CDC [title screener]

B. Publication and authorship
   1. Decisions on who will be an author will be based on Harvard University guidelines (Annex 5). To be a co-author, the individual needs to have: 1) made a substantial intellectual contribution to the conception, design, analysis, and/or interpretation of data; and 2) participated in writing a manuscript. Individuals who have only assisted with data management or data abstraction and individuals who have only provided informal input (e.g., providing comments on a protocol or manuscript) will be a contributor; all such contributors will be named in the Acknowledgment section.
   2. There are several potential publications from this project (e.g., summary of the entire review, policy implications, framework/theory of HCP practices, reviews of specific interventions)

C. Responsibilities
   1. Drs. Chalker, Holloway, Peters, Ross-Degnan, A. Rowe, and S. Rowe will all help plan, review, and edit the protocol, reports, and manuscripts. Other individuals will assist with these tasks, according to their time and interest.
   2. Dr. A. Rowe will document meetings; ensure that group decisions are reflected in the protocol; oversee the literature search, data abstraction, data management, and analysis; and write initial drafts of a report or manuscript. Dr. A. Rowe will be the first author of the main review paper. Other individuals will assist with these tasks, according to their time and interest.

VI. METHODS

A. <u>General description.</u> This project will be a systematic review of published and unpublished studies meeting minimum study design criteria. Where appropriate, sensitivity analyses will be conducted in which studies that satisfy a slightly less rigorous set of inclusion criteria are added to the main group of included studies. Results will be stratified by a variety of factors (e.g., intervention type, study design characteristics, adequacy of statistical analysis, and study outcome). The methods are based on those used by Ross-Degnan and colleagues [Ross-Degnan, 1997], WHO [WHO, 2001], and Grimshaw and colleagues [Grimshaw, 2004]. The review will adhere to the Quality of Reporting of Meta-analyses (QUORUM) guidelines [Moher, 1999] and guidelines for reporting economic studies [Drummond & Jefferson, 1996]. Development of the theory to explain HCP practices will involve drafting an initial theoretical framework based on existing theories, searching for evidence while conducting the systematic review that

supports or refutes aspects of the initial framework, and refining the initial framework based on evidence gathered during the review.

B. Scope of the review/inclusion & exclusion criteria for studies
   1. Timing. For studies identified through the search of electronic databases, studies were included if they were published by May 31, 2006. For studies identified through other methods (personal libraries and search of gray literature), studies were included if they were found by September 15, 2010.
   2. Setting. LMICs were defined as either a "low-income" country, "lower-middle-income" country, or an "upper-middle-income" country, according to the World Bank [World Bank website, http://web.worldbank.org/WBSITE/EXTERNAL/DATASTATISTICS/0,,contentMDK:20421402~menuPK:64133156~pagePK:64133150~piPK:64133175~theSitePK:239419,00.html accessed October 14, 2005]. See Annex 2 for search strategy.
   3. Type of health condition. No exclusions. Performance related to any health condition is acceptable.
   4. Type of HCP. Any health facility-based health worker, community health workers (any person who is part of a recognized community health worker program), pharmacists, and shopkeepers who sell medicines. Private sector health workers with at least some medical or para-medical training will also be included. Household-based providers (e.g., a patient's family or neighbors) are excluded.
   5. Type of intervention. Any intervention with at least 1 component that aims to influence HCP performance.
      a) Note. There are rare situations in which a study should be split into different records (i.e., sub-studies), such that the effect sizes in each record perfectly correspond with the intervention components coded in the record. The Data Coordinator will be consulted to confirm whether and how a study should be split. The situations in which a study should be split into different sub-studies are:
         1. Distinct intervention components in a single study group are implemented far apart in time, not intended to be implemented as a package, with observations between components' implementation (e.g., 1 "sub-study" [record 1] examines the effect of training only, and another sub-study [record 2] examines the combined effect of training and supervision)
         2. Two intervention groups have different timing of strategies and with observations between components' implementation, allowing the opportunity for both medium and high priority effect sizes (e.g., one record examines the effect of training only, and another record examines the marginal effect of supervision over training)
         3. An intervention involves facility- and community-level components, with outcomes measured at facility and community levels (e.g., one record examines the effect of facility-level components on facility-level outcomes, and another record examines the effect of both facility- and community-level components on community-level outcomes)

6. Study design.
   a) Studies with at least one "primary" outcome and with one of the following study designs are considered to be of "adequate" design and will be included in the primary analysis:
      1. Randomized controlled before-and-after trials
      2. Non-randomized controlled before-and-after trials
      3. Randomized controlled post-only trials
      4. Interrupted time series designs with at least 3 data points before and after the intervention
      5. Replicated trials that individually lack an "adequate" design in which replications show consistent results. That is, the same strategies are implemented in 2 or more different places, and each of these replications is evaluated with a before-and-after trial without controls, or a non-randomized post-only trial with controls. A potential example is the study by Chalker [Chalker, 2001]. Results from all replications will be pooled (weighted by the sample size of the replication) to derive a single effect size.
   b) The following will be collected, but analyzed separately, as part of a sensitivity analysis:
      1. Interrupted time series designs with <3 data points before and after the intervention (this design includes before-and-after trials without controls—e.g., simple program evaluations). Such studies might have greater validity if there is compelling evidence that the intervention was the only major influence on performance. For example, if the pre- and post-intervention performance measurements are close together in time, there might not be enough time for potentially confounding factors to change (thus, they cannot confound intervention-outcome relationships). We acknowledge that it might be difficult to judge whether there is "compelling evidence that the intervention was the only major influence on performance."
      2. Non-randomized post-only trials with controls. Such studies might have greater validity if there is compelling evidence that the control group was similar to the intervention group. We acknowledge that it might be difficult to judge whether this "compelling" evidence exists.
7. Type of outcomes.
   a) Objective or "direct" measures of HCP behavior, including tasks related to patient assessment, diagnosis and diagnostic testing, medicine use for specific conditions (medicines prescribed; medicines dispensed), general medicine use (medicines prescribed; medicines dispensed), injection practices, counseling (content of counseling; interpersonal quality of communication), referral (recommended referrals; completed referrals), and prevention activities
   b) Patient outcomes, including health outcomes (morbidity, mortality), patient knowledge, patient adherence to therapy, patient satisfaction, and

health care utilization (e.g., number of outpatient, emergency department, or inpatient visits). Note that there needs to be some evidence that HCPs were in the chain of events leading to the health outcome.

c) Economic outcomes, including cost of medicines, and costs of outpatient, emergency department, or inpatient visits
d) "Intermediate" outcomes, including HCP knowledge, HCP skills, and drug supply
e) Note 1. Results from studies that only report on health outcomes (e.g., community health worker studies that only report the impact on child mortality) might be analyzed separately from studies that include process indicators of HCP performance.
f) Note 2. Results from studies that only report on performance indicators that are not based on a specific guideline (e.g., "% of all patients receiving an antibiotic" when the study did not assess whether or not patients needed an antibiotic) might be analyzed separately from studies in which performance outcomes are based on a specific guideline.
g) Note 3. Results from studies that only report on "intermediate" outcomes (e.g., HCP knowledge) might be analyzed separately from studies that include more direct indicators of HCP performance.
h) Note 4. Outcomes that are excluded: 1) outcomes that are not compared between $\geq$2 study groups or are not compared over time in one study group (e.g., outcomes that are reported as the combined result of all study groups and thus no effect size can be calculated); 2) outcomes that involve <20 observations per study group and time point in all comparisons; 3) outcomes that are similar to another outcome that was already abstracted (e.g., exclude the complement or subgroup-specific results of an already-abstracted outcome)[2]; 4) outcomes whose results were extrapolated from a model (e.g., simulation study) and not actually observed data; and 5) outcomes with methodological problems such that their measures are not interpretable[3].

8. Outcome measures: Generally, all outcome measures are abstracted. However, when the abstraction of all measures seems onerous (e.g., a randomized controlled post-only trial that involves >5 measures per outcome), the Data Coordinator will be consulted to consider whether fewer measures per outcome might be abstracted (e.g., only abstract first, median, and final post-intervention measures). Note that for interrupted time series studies, all outcome measures are abstracted.
9. Sample size. No exclusion criteria, but sample size of studies will be examined at the time of analysis to determine if any need to be excluded. Note that all

---

[2] If the subgroup-specific indicators are well-accepted indicators of impact or are indicators that reflect the different standard treatment guidelines for each subgroup, these subgroup-specific outcomes should be included. For example, in a study of febrile patients, "% of patients aged <5 years treated correctly" and "% of patients aged 5+ years treated correctly" should be included because fever treatment guidelines differ for children and older patients.

[3] For example, in a study in which pregnant women are recommended to receive both antenatal and postnatal care, exclude "% of women who had only antenatal care visits" because a decrease in this outcome could be due to an increase in good behavior (% of women who had both antenatal and postnatal care visits) or an increase in bad behavior (% of women who had neither antenatal nor postnatal visits).

comparisons (e.g., study group 1 vs. study group 2) must have ≥20 observations per study group and time point.

10. Adequacy of statistical analysis. No exclusion criteria, but results of studies with inadequate analyses (especially "unit of analysis error," e.g., ignoring correlation when there are multiple consultations per HCP) might be: displayed differently from studies with adequate analyses, adjusted (e.g., given a lower relative weight), or removed in a sensitivity analysis.
11. Language. No restriction on language of the studies.

C. Search strategy[4]

1. Search 15 electronic databases, including: Campbell Collaboration, CINAHL (Cumulative Index to Nursing & Allied Health Literature), Cochrane Library (which includes Database of Abstracts of Review of Effects (DARE) and the Cochrane Central Register of Controlled Trials (CENTRAL)), Dissertation Abstracts (for theses and dissertations), EconLit, Eldis, EMBASE, the Effective Practice and Organization of Care (EPOC) specialized register, ERIC (Education Resources Information Center), Global Health, HMIC (The Healthcare Management Information Consortium), MEDLINE, SCI (Science Citation Index), Sociological Abstracts, and SSCI (Social Sciences Citation Index). Use an iterative approach (see Annex 2 for details). [Note: we intentionally did not search Healthline (because it is a consumer health website and we did not expect to find many relevant results), Healthstar (by the time of our search, its data had been merged into MEDLINE, which we were already searching), and SIGLE (because its primarily contained gray literature on studies from high-income countries)]
    a) Perform initial electronic search based on the INRUD strategy (after modification to expand outcomes beyond drug use)
    b) Compare results against a list of known studies that meet the inclusion criteria
    c) Revise and implement the search strategy to increase sensitivity
    d) Add additional search terms from World Bank staff and re-ran the search
2. Bibliographies of previous systematic reviews and other related papers. See Annex 2 for details.
3. Hand search of the INRUD bibliography (as of February 12, 2007) and Kathy Holloway's database (as of June 27, 2007).
4. Searches of document inventories and websites of organizations involved in implementing interventions to improve HCP performance. See Annex 2 for details.
5. Contact colleagues and ask for references and unpublished studies (Annex 2).

[4] In addition to the five methods described in this section, the investigators had planned to perform a hand-search of 30 key journals (i.e., the 15 journals that are most and the 15 journals that are least commonly represented among studies that are known to meet the inclusion criteria). However, as this method would have required searching 158,000 titles (for journal issues published from 1980 to 2008) and staff time was limited, the investigators decided to drop this method.

D. Data abstraction (see Annex 3 for details)
   1. Basic descriptive aspects of studies
   2. "Contextual" factors (see Annex 4)
   3. Quality criteria (see Annex 3)
   4. Details on analysis: estimate of the effect in each group, standard error of the estimate of effect, significance level, appropriateness of analysis method (correct or incorrect), description of any analysis problem
   5. Data from each study will be abstracted twice (by 2 different investigators) onto forms. Data abstracted by the 2 investigators will be compared and discrepancies will be reconciled (with consultation with a third investigator, if necessary). Data from the final version of the completed abstraction form will be stored as a Microsoft Access file (Microsoft Inc. Redmond, Washington).

E. Data collection from study investigators
   1. <u>Contextual factors.</u> See bullet VI.D.2. Contextual factors may not be in published articles or unpublished reports, and an attempt will be made to obtain this information from study investigators.
   2. <u>Qualitative data collection on factors that influenced intervention effectiveness.</u> Study investigators may have well-informed and reasonably valid opinions about the factors that influenced the effectiveness of the intervention they examined, and such factors may not have been included (or were only briefly mentioned) in a published article or unpublished report. An attempt will be made to obtain this information from study investigators. (See Annex 4 for details.)

F. Analysis
   1. Descriptive analysis
      a) Definition of effect size for dichotomous outcomes
         1. Effect size = $(\%POST - \%PRE)_{intervention} - (\%POST - \%PRE)_{reference}$
         2. If there is no reference study group, but there is a pre- and post-intervention measurement, effect size = $(\%POST - \%PRE)_{intervention}$
         3. If there are no baseline measurements, but there is a reference group, effect size = $(\%POST)_{intervention} - (\%POST)_{reference}$
      b) Definition of effect size for outcomes that are a percentage for each patient or subject
         1. Summarize multiple observations as a mean percentage. Effect size = $(\%POST - \%PRE)_{intervention} - (\%POST - \%PRE)_{reference}$
         2. If there is no reference study group, but there is a pre- and post-intervention measurement, effect size = $(\%POST - \%PRE)_{intervention}$
         3. If there are no baseline measurements, but there is a reference group, effect size = $(\%POST)_{intervention} - (\%POST)_{reference}$
      c) Definition of effect size for outcomes that are continuous, but <u>obviously</u> bounded (e.g., knowledge score from 0 to 50) and summarized as a mean score

1. Convert the mean score to a percentage by dividing the mean score by the maximum possible score. Effect size = $(\%POST - \%PRE)_{intervention} - (\%POST - \%PRE)_{reference}$
2. If there is no reference study group, but there is a pre- and post-intervention measurement, then convert the mean score to a percentage by dividing the mean score by the maximum possible score. Effect size = $(\%POST - \%PRE)_{intervention}$
3. If there are no baseline measurements, but there is a reference group, then convert the mean score to a percentage by dividing the mean score by the maximum possible score. Effect size = $(\%POST)_{intervention} - (\%POST)_{reference}$
4. Note 1. The maximum possible score must be the same for all subjects.
5. Note 2. If minimum possible score is not zero, then shift mean scores so minimum = zero (e.g., if score ranges from -10 to +10, then add 10 to all results, so the range is shifted to 0 to +20).

d) Definition of effect size for outcomes that are continuous, but not obviously bounded (e.g., mortality rate)
   1. Effect size = $100\% * \{([POST - PRE] / PRE)_{intervention} - ([POST - PRE] / PRE)_{reference}\}$
   2. If there is no reference study group, but there is a pre- and post-intervention measurement, effect size = $100\% * ([POST - PRE] / PRE)_{intervention}$
   3. If there are no baseline measurements, but there is a reference group, effect size = $100\% * (POST_{intervention} - POST_{reference}) / POST_{reference}$

e) For the analysis of interrupted time series studies, see Annex 6

f) Other possible methods, as appropriate
   1. Relative differences
   2. $[(POST - PRE)_{intervention} - (POST - PRE)_{reference}]$ / standard deviations of pre-intervention measurements (i.e., average of intervention and control groups)
   3. Standardized mean difference (SMD), from Grimshaw et al. (page 75) where SMD = $(POST_{intervention} - POST_{reference})$ / standard deviation of $PRE_{reference}$

g) <u>Note 1.</u> Studies often have multiple outcomes and multiple potential effect sizes. This review will analyze only one "best" effect size per study. To identify a study's "best" effect size, outcomes will be categorized as "primary" or other; and effect sizes will be categorized as high, medium, or low.
   1. Categorizing outcomes. Analyses will be conducted on the "primary" outcome of the study (i.e., as identified by investigators, or as clearly indicated by the subject of the study). If more than one primary outcome is described, then a median of primary outcomes will be calculated. If no primary outcomes can be

identified, then median will be calculated of all relevant outcomes that are reported.

2. Categorizing effect sizes. Effect sizes will be categorized as high, medium, or low priority. For each study, the highest category effect size (or median of effects sizes, if more than one) will be included in the final analysis.
    a. High priority means the effect size compares an intervention and non-intervention control group and is from an adequate design study and the post-intervention measurement taken furthest from the time of intervention.
    b. Medium priority means the effect size compares 2 intervention groups (rather than between an intervention group and control group) and is from an adequate design study and the post-intervention measurement taken furthest from the time of intervention.
    c. Low priority means all other types of effect sizes.

h) Note 2. Regarding sample size, all comparisons (e.g., study group 1 vs. study group 2) must have $\geq$20 observations per study group and time point.

i) Note 3. The effect size expression (arithmetic difference of differences—see section VI.F.1.a) can lead to problematic results when the outcome worsens in the control group. Here are two examples.
    1. Effect sizes can have values ranging from –200 percentage points (if $PRE_{control}$ = 0%, $POST_{control}$ = 100%, $PRE_{intervention}$ = 100%, and $POST_{intervention}$ = 0%) to +200 percentage points (if $PRE_{control}$ = 100%, $POST_{control}$ = 0%, $PRE_{intervention}$ = 0%, and $POST_{intervention}$ = 100%). Effect sizes that are greater than 100 percentage points or substantially less than zero can be difficult to interpret. Such results generally require that the outcome worsened in the control group over time. In these instances, the plausibility of such worsening should be carefully explored.
    2. Positive effect sizes of a “reasonable” magnitude (e.g., +25 percentage points) could result from no change in the intervention group (e.g., $PRE_{intervention}$ = 55% and $POST_{intervention}$ = 55%), but a worsening in the control group (e.g., $PRE_{control}$ = 60% and $POST_{control}$ = 35%). As above, in these instances, the plausibility of such worsening should be carefully explored.

j) Note 4. Some studies report results separately for replications of the same basic study (i.e., same strategies implemented in the same setting). For example, Weinberg et al. (2001) studies the effect of a continuous quality improvement strategy in two similar hospitals in Bogotá, Colombia. Reporting results separately can be useful for showing how the same strategy can have different effects in different facilities (or for different HCPs). However, for this review, an effort will be made to combine such results. The justification is that results for larger groups of health facilities or HCPs are more generalizable than results for an

individual health facility or HCP. One could argue that the effect of a strategy will always vary at least somewhat among facilities or HCPs, and thus results should be presented separately. However, it is because results will vary that combining results can improve generalizability.

k) Note 5: If the baseline measurement of a continuous, unbounded outcome (e.g., number of IUDs inserted per month) for a study group is zero, the effect size is $[POST - PRE]_{intervention} - [POST - PRE]_{reference}$, rather than $100\%*\{([POST - PRE] / PRE)_{intervention} - ([POST - PRE] / PRE)_{reference}\}$. For example, if $PRE_{intervention} = 0$, $POST_{intervention} = 21$, $PRE_{reference} = 1$, and $POST_{reference} = 10$; then effect size = (21–0) – (10–1) = 12. Note that these effect sizes will be analyzed separately from others because their units will be different.

l) Note 6: For continuous, unbounded outcomes in which only the before-and-after differences in each study group are available (i.e., individual baseline and follow-up measures are not available), the effect size is $100\% *([POST - PRE]_{intervention} - [POST - PRE]_{reference})/([POST - PRE]_{reference})$. For example, if $[POST - PRE]_{intervention} = 20$ and $[POST - PRE]_{reference} = 10$, then effect size = 100%*(20–10)/10 = 100 %-points. In essence, the before-and-after difference is the "outcome".

m) Note 7: Since May 17, 2010, only high- or medium-priority effect sizes are abstracted. However, the database will include all outcome measures, even those that contribute to low-priority effect sizes. Thus, database users will have the data needed to calculate the low-priority effect sizes.

2. Other analyses
    a) Analyze strength of study design and adequacy of statistical analyses over time (e.g., studies from 1980s vs. 1990s vs. 2000s)
    b) Meta-analysis, if studies are combinable
    c) Identification of study and intervention characteristics (including contextual factors) associated with cost or effectiveness
    d) Analyses of "positive deviance" case studies—i.e., studies that managed to increase outcomes to close to 100% (e.g., Chalker, 2001; and Weinberg et al., 2001) or studies with effect sizes >50 percentage points.

VII. REFERENCES

Amaral JJ, Victora CG. The effect of training in Integrated Management of Childhood Illness (IMCI) on the performance and healthcare quality of pediatric healthcare workers: a systematic review. Revista Brasileira de Saúde Materno Infantil 2008;8(2):151–61.

Grace C, James J, Hadi Y. Selective review of work aids for alternative health care providers in developing countries. Report prepared for the Bill and Melinda Gates Foundation, June 2008.

Siddiqi K, Newell J, Robinson M. Getting evidence into practice: what works in developing countries? Int J Qual Health Care. 2005 Oct;17(5):447-54.

Abas M, Baingana F, Broadhead J, Iacoponi E, Vanderpyl J. Common mental disorders and primary health care: current practice in low-income countries. *Harvard Review of Psychiatry* 2003; **11**: 166–73.

Alliance for Health Policy and Systems Research. Strengthening health systems: the role and promise of policy and systems research. Geneva: Alliance for Health Policy and Systems Research, 2004.

Arifeen SE, Blum LS, Hoque DME, Chowdury EK, Khan R, Black RE, et al. Integrated Management of Childhood Illness (IMCI) in Bangladesh: early findings from a cluster-randomized study. *Lancet* 2004; **364:** 1595–1602.

Bickler SW, Rode H. Surgical services for children in developing countries. *Bull World Health Organ* 2002; **80**: 829–835.

Bitera R, Alary M, Masse B, et al. [Quality of disease management of sexually transmitted diseases: investigation of care in six countries in West Africa]. *Sante* 2002; **12**: 233–9.

Bose S, Oliveras E, Edson WN. 2001. How can self-assessment improve the quality of healthcare? Operations Research Issue Paper 2(4). Published for the U.S. Agency for International Development by the Quality Assurance Project, Bethesda, MD, and JHPIEGO Corporation, Baltimore, MD. Available on the Internet at address: http://www.qaproject.org/pubs/PDFs/selfassess402.pdf.

Briggs CJ, Capdegelle P, Garner P. Strategies for integrating primary health services in middle- and low-income countries: effects on performance, costs and patient outcomes. *Cochrane Database of Systematic Reviews* (4):CD003318, 2001.

Brugha R, Zwi A. Improving the quality of private sector delivery of public health services: challenges and strategies. *Health Policy and Planning* 1998; **13**: 107–120.

Bryce J, el Arifeen S, Pariyo G, Lanata CF, Gwatkin D, Habicht JP, et al. Reducing child mortality: can public health deliver? *Lancet* 2003; **362**: 159–164.

Chalker J. Improving antibiotic prescribing in Hai Phong Province, Viet Nam: the "antibiotic-dose" indicator. *Bull World Health Organ* 2001; **79**: 313–320.

Chen L, Evans T, Anand S, Boufford JI, Brown H, Chowdhury M, et al. Human resources for health: overcoming the crisis. *Lancet* 2004; **364:** 1984–90.

Drummond MF, Jefferson TO, for the BMJ Working Party on guidelines for authors and peer-reviewers of economic submissions to the *British Medical Journal*. Guidelines for authors and peer-reviewers of economic submissions to the *British Medical Journal*. *BMJ* 1996;313:275–83.

Goldman N, Glei DA. Evaluation of midwifery care: results from a survey in rural Guatemala. *Soc Sci Med* 2003; **56**: 685–700.

Grimshaw JM, Thomas RE, MacLennan G, Fraser C, Ramsay C, Vale L et al. Effectiveness and efficiency of guideline dissemination and implementation strategies. *Health Technol Assess* 2004: **8**(6).

Grol R. Beliefs and evidence in changing clinical practice. *BMJ* 1997; **315**: 418–421.

Grol R, Grimshaw J. From best evidence to best practice: effective implementation of change in patient care. *Lancet* 2003; **362**: 1225–30.

Hogerzeil HV, Bimo, Ross-Degnan D, et al. Field tests for rational drug use in twelve developing countries. *Lancet* 1993; **342**: 1408–10.

Jones G, Steketee RW, Black RE, Bhutta ZA. Morris SS. Bellagio Child Survival Study Group. How many child deaths can we prevent this year? *Lancet* 2003; **362**: 65–71.

Knebel E. 2000a. The use of manual job aids by health care providers: What do we know? Operations Research Issue Paper 1(1). Bethesda, MD: Published for the U.S. Agency for International Development by the Quality Assurance Project, Center for Human Services, University Research Co., LLC. Bethesda, MD. Available on the Internet at address: http://www.qaproject.org/pubs/PDFs/ISSUESJA.PDF.

Knebel E. 2000b. The use and effect of computer-based training: What do we know? Operations Research Issues Paper 1(2). Published for the U.S. Agency for International Development by the Quality Assurance Project, Center for Human Services, University Research Co., LLC. Bethesda, MD. Available on the Internet at address: http://www.qaproject.org/pubs/PDFs/researchcbtx.pdf.

Knebel E. 2001. The use and effect of distant education in healthcare: What do we know? Operations Research Issue Paper 2(2). Bethesda, MD: Published for the U.S. Agency for International Development by the Quality Assurance Project, Center for Human Services, University Research Co., LLC. Bethesda, MD. Available on the Internet at address: http://www.qaproject.org/pubs/PDFs/distlrnissue.pdf.

Mills A, Brugha R, Hanson K, McPake B. What can be done about the private health sector in low-income countries? *Bull World Health Organ* 2002; **80**: 325–30.

Moher D, Cook DJ, Eastwood S, Olkin I, Rennie D, Stroup DF, for the QUOROM Group. Improving the quality of reports of meta-analyses of randomised controlled trials: the QUOROM statement. *Lancet* 1999; **354**:1896–1900.

Naimoli J. Theoretical and Empirical Advances in Research on the Implementation of an Integrated Approach to Managing Childhood Illness in Outpatient Facilities in Developing Countries [doctoral dissertation]. Cambridge, MA: Harvard School of Public Health, 2001.

Narasimhan V, Brown H, Pablos-Mendez A, Adams O, Dussault G, Elzinga G, Nordstrom A, Habte D, Jacobs M, Solimano G, Sewankambo N, Wibulpolprasert S, Evans T, Chen L. Responding to the global human resources crisis. *Lancet* 2004; **363**: 1469–72.

Ratanawijitrasin S, Soumerai SB, Weerasuriya K. Do national medicinal drug policies and essential drug programs improve drug use?: a review of experiences in developing countries. *Soc Sci Med* 2001; **53**: 831–844.

Ross-Degnan D, Laing R, Santoso B, Ofori-Adjei, D, Lamoureux C, Hogerzeil H. Improving pharmaceutical use in primary care in developing counties: a critical review of experience and lack of experience. Presented at the International Conference on Improving Use of Medicines, Chiang Mai, Thailand, April 1997.

Rowe AK, Hamel MJ, Flanders WD, Doutizanga R, Ndoya J, Deming MS. Predictors of correct treatment of children with fever seen at outpatient health facilities in the Central African Republic. *Am J Epidemiol* 2000; **151**: 1029–1035.

Rowe AK, Onikpo F, Lama M, Cokou, F, Deming MS. Management of childhood illness at health facilities in Benin: problems and their causes. *Am J Public Health* 2001; **91**: 1625–1635.

Rowe AK, Lama M, Onikpo F, Deming MS. Risk and protective factors for two types of error in the treatment of children with fever at outpatient health facilities in Benin. *Int J Epidemiol* 2003; **32**: 296–303.

Task Force on Health Systems Research. Informed choices for attaining the Millennium Development Goals: towards an international cooperative agenda for health-systems research. *Lancet* 2004; **364:** 997–1003.

Travis P, Bennett S, Haines A, Pang T, Bhutta Z, Hyder AA, Pielemeier NR, Mills A, Evans T. Overcoming health-systems constraints to achieve the Millennium Development Goals. *Lancet* 2004; **364:** 900–6.

Weinberg M, Fuentes JM, Ruiz AI, et al. Reducing infections among women undergoing cesarean section in Colombia by means of continuous quality improvement methods. *Archives of Internal Medicine* 2001; **161:** 2357–65.

Woodward CA. Issues in health services delivery Discussion paper No. 1: Improving provider skills: strategies for assisting health workers to modify and improve skills: developing quality health care—a process of change. Department of Organization of Health Services Delivery, World Health Organization, Geneva. 2000. Document number: WHO/EIP/OSD/00-1.

Wootton R. Telemedicine and developing countries--successful implementation will require a shared approach. *Journal of Telemedicine and Telecare* 2001; **7** (suppl 1) :1–6.

World Bank. The World Development Report 2004: Making services work for poor people. New York: Oxford University Press. 2004.

World Health Organization. Interventions and strategies to improve the use of antimicrobials in developing countries. Drug Management Program, World Health Organization, Geneva. 2001. Document number: WHO/CDS/CSR/DRS/2001.9. (Plus a personal communication from J. Chalker on May 17, 2004, which included revised results tables.)

Zurovac, Rowe AK, Ochola SA, Noor AM, Midia B, English M, et al. Predictors of the quality of health worker treatment practices for uncomplicated malaria at government health facilities in Kenya. *Int J Epidemiol* 2004; **33:** 1080–91.

ANNEX 1. Definitions

Determinants of HCP performance include interventions designed to improve performance (e.g., training) and any other factor that could influence performance (e.g., HCP's age, cadre, or severity of a patient's illness).

Health care providers (HCPs) include any health facility-based health worker, community health workers (any person who is part of a recognized community health worker program), pharmacists, and shopkeepers and informal vendors who sell medicines. Private sector health workers with at least some medical or para-medical training will also be included.

Health facility means any building in which health services are delivered, from small health posts to large, tertiary care hospitals.

Managers are all health facility staff and their supervisors (at any level of the health system, and including professional staff, non-professional staff, and administrators) who perform managerial or supervisory functions for health workers, regardless of whether or not the functions are part of the person's official duties.

Medical errors are deviations from an accepted standard of practice, recognizing that some errors are clearly more serious than others (e.g., major versus minor treatment errors [Rowe, 2003]).

Performance includes both clinical competence (e.g., adherence to a policy, standard, or clinical practice guideline) and interpersonal skills (i.e., HCPs treat patients and clients with kindness and respect, and HCPs motivate patients and clients to follow the HCP's instructions). For the purposes of this review, performance includes both adherence to a policy or guideline as well as more general performance indicators that are not necessary linked to a specific policy or guideline (e.g., "% of all patients receiving an antibiotic" when the study did not assess whether or not patients needed an antibiotic).

Private health workers or *workers in the private health sector* are "all providers who exist outside the public sector, whether their aim is philanthropic or commercial, and whose aim is to treat illness or prevent disease." [Mills, 2002]

ANNEX 2. Details of the search strategy

We developed a strategy to maximize our ability to identify articles that were relevant to our review. This appendix describes the development of this search strategy.

A.1. Development of a gold standard list of studies

First, we created a list of “gold standard” studies, studies we considered to meet the inclusion criteria for this review. Our gold standard list comprised of studies on health care provider behavior change strategies cited in the bibliographies of the following 4 reviews:

Loevinsohn B, Harding A. Buying results? Contracting for health service delivery in developing countries. Lancet 2005; 366: 676–681.

Ross-Degnan D, Laing R, Santoso B, Ofori-Adjei D, Lamoureux C, Hogerzeil H. Improving pharmaceutical use in primary care in developing counties: a critical review of experience and lack of experience. Presented at the International Conference on Improving Use of Medicines, Chiang Mai, Thailand, April 1997.

Siddiqi K, Newell J, Robinson M. Getting evidence into practice: what works in developing countries? International Journal for Quality in Health Care 2005; 17(5): 447–453.

WHO. Interventions and strategies to improve the use of antimicrobials in developing countries: a review. World Health Organization 2001, WHO/CDS/CSR/DRS/2001.9.

Our gold standard set contained 118 references, 84 of which were contained in electronic databases (the remaining gold standard articles were book chapters or unpublished reports).

A.2. Development of an electronic search strategy

Next, we developed an electronic search strategy to maximize the search’s ability to retrieve gold standard articles that were contained in electronic databases.

We conducted electronic searches of 3 databases:

1) Medical Literature Analysis and Retrieval System Online (MEDLINE [1951 – November 4, 2005])
2) Excerpta Medica Database (EMBASE [1980 – November 4, 2005])
3) Cumulative Index to Nursing and Allied Health Literature (CINAHL [1982 – November 4, 2005])

To link with and search the MEDLINE database, we used PubMed. To link with and search the EMBASE and CINAHL databases, we used OVID.

As a baseline test, we used the strategy developed by Ross-Degnan et al. to search MEDLINE, EMBASE, and CINAHL. This test identified only 12% of the gold standard articles contained in

the electronic databases. To improve the search's sensitivity, we modified the strategy by Ross-Degnan et al. by: 1) eliminating the steps that searched specifically for drug studies and certain types of common illnesses, and 2) revising the terms to identify developing countries. We also reviewed the Medical Subject Heading (MeSH) terms and keywords of the gold standard articles that had not been retrieved to identify words to be added to the search term list. Several iterations of electronic searching, reviewing MeSH terms and keywords, and revising the search terms resulted in a strategy that identified 63 of the 84 gold standard articles contained in electronic databases, giving a **sensitivity of 75%**. This strategy retrieved a total of **19,648 articles**.

We used this strategy as a basis for the strategies for the Cochrane Library/EPOC, Dissertation Abstracts, EconLit, Eldis, and SCI/Web of Science databases. The strategies for searching CINAHL, Cochrane Library/EPOC, Dissertation Abstracts, EconLit, Eldis, ERIC, EMBASE, MEDLINE (via PubMed), and SCI/Web of Science without additional World Bank terms are listed below.

CINAHL and EMBASE (via OVID)

(Community Care/ or Health Care Personnel/ or Health Care Facility/ or health worker(s).ti)
AND
(Continuing Education or Medical Education or Paramedical Education or Medical Audit or Practice Guidelines or intervention.ti or audit.ti or training.ti)

Cochrane Library and EPOC

(community health services / health personnel / health services research / pharmaceutical services / pharmacy services, hospital)
AND
(education, continuing / health care costs / health education / health knowledge, attitudes, practice / inservice training / quality assurance, health care / audit / impact / training / intervention)

Dissertation Abstracts

(Health care or Health education)
AND
(education or treatment guidelines or training or educational or standard treatment or treatment practices or audit or compliance or continuing education or evaluation or program evaluation or quality assurance or health care quality)

EconLit

(health facilities or community health or medical student(s) or health workers or public services or public physicians or primary medical care or family physicians or public health facilities or rural health or primary health care or medical auxiliaries or health staff or medical assistants or health systems or pharmacists or hospital or health aides or health services or family practice or health personnel or physician(s) or health centres or private practice or voluntary workers or

health care providers or health post(s))
AND
(treatment or guidelines or training or educational or workshops or standard management or structured care or seminar or assessment or audit or compliance or evaluation or program evaluation or quality assurance or quality health care)
AND
(Asia or West Indies or Polynesia or Micronesia or Middle East or Africa or Latin America or Central America or South America or Caribbean or West Indies region or Hispañola or Southeast Asia or Sub-Saharan Africa or Eastern Europe or the Balkans)

<u>Eldis</u>

(hospital / aides / health services / practice / personnel / facilities / workers / physicians)
AND
(intervention / education / training / implementation / standard care / standard treatment / guidelines)

<u>ERIC</u>

(hospital(s) or health or family practice or personnel or physician(s) or medical student(s) or medical or medicine)
AND
(intervention or education or training or implementation or guidelines or educational Practices or workshop(s) or seminar(s))

<u>MEDLINE (via PubMed)</u>

1. (children's hospital[ti] or community health services[majr:noexp] or doctor(s)[tiab] or health personnel[mesh] or health posts[tiab] or health services research[mesh:noexp] or health workers[tiab] or hospital(s)[tiab] or medical students[ti] or obstetric care[ti] or pharmaceutical services[majr:noexp] or pharmacy service, hospital[majr:noexp] or physicians[tiab] or primary health care[tiab] or primary health centres[tiab] or providers[ti])

2. (audit[ti] or education, continuing[mesh] or health care costs[mesh] or health education[mesh] or health knowledge, attitudes, practice[mesh] or impact[ti] or inservice training[mesh] or intervention[tiab] or quality assurance, health care[mesh] or training[ti])

3. ((Afghanistan or Bangladesh or Benin or Bhutan or Burkina Faso or Burundi or Cambodia or Cameroon or Central African Republic or Chad or Comoros or Congo or the Democratic Republic of Congo or Cote d'Ivoire or Eritrea or Ethiopia or The Gambia or Ghana or Guinea or Guinea-Bissau or Haiti or India or Kenya or Democratic Republic of Korea or Kyrgyszstan Republic or Lao PDR or Lesotho or Liberia or Madagascar or Malawi or Mali or Mauritania or Moldova or Mongolia or Mozambique or Myanmar or Nepal or Nicaragua or Niger or Nigeria or North Korea or Pakistan or Papua New Guinea or Rwanda or Sao Tome and Principe or Senegal or Sierra Leone or Solomon Islands or Somalia or Sudan or Tajikistan or Tanzania or Timor-Leste or Togo or Uganda or Uzbekistan or Vietnam or Yemen or Republic of Yemen or Zaire or

Zambia or Zimbabwe) or (Albania or Algeria or Angola or Armenia or Azerbaijan or Belarus or Bolivia or Bosnia and Herzegovina or Brazil or Bulgaria or Cape Verde or China or Colombia or Cuba or Djibouti or Dominican Republic or Ecuador or Egypt or Arab Republic of Egypt or El Salvador or Fiji or Georgia or Guatemala or Guyana or Honduras or Indonesia or Iran or Islamic Republic of Iran or Iraq or Jamaica or Jordan or Kazakhstan or Kiribati or Macedonia or FYR of Macedonia or Former Yugoslav Republic of Macedonia or Maldives or Marshall Islands or Micronesia or Federated States of Micronesia or Morocco or Namibia or Paraguay or Peru or Philippines or Romania or Samoa or Serbia and Montenegro or Sri Lanka or Suriname or Swaziland or Syrian Arab Republic or Syria or Thailand or Tonga or Tunisia or Turkmenistan or Ukraine or Vanuatu or West Bank and Gaza) or (American Samoa or Antigua and Barbuda or Argentina or Barbados or Belize or Botswana or Chile or Costa Rica or Croatia or Czech Republic or Dominica or Equatorial Guinea or Estonia or Gabon or Grenada or Hungary or Latvia or Lebanon or Libya or Lithuania or Malaysia or Mauritius or Mayotte or Mexico or Northern Mariana Islands or Oman or Palau or Panama or Poland or Russian Federation or Seychelles or Slovak Republic or South Africa or St. Kitts and Nevis or St. Lucia or St. Vincent and the Grenadines or Trinidad and Tobago or Turkey or Uruguay or Venezuela or Bolivarian Republic of Venezuela))

4. (developing countries or less developed countries or third-world countries or under-developed countries or poor countries or less developed countries or under developed countries or less developed nations or third world nations or under developed nations or developing nations or poor nations or poor economies or third world economies or developing economies or under developed economies or less developed economies)

5. (Burma or Czechoslovakia or Democratic Republic of Congo or French Guiana or East Timor or Laos or North Korea or Ivory Coast or Republic of Georgia or Republic of Yemen or Republic of Zaire or Slovakia or Soviet Union or Surinam or USSR or West Samoa or Yugoslavia or Zaire)

6. (Asia or West Indies or Polynesia or Micronesia or Middle East or Africa or Latin America or Central America or South America or Caribbean or West Indies region or Hispañola or Southeast Asia or Sub-Saharan Africa or Eastern Europe or the Balkans)

7. #3 or #4 or #5 or #6

8. #1 and #2 and #7

<u>SCI/Web of Science</u>

(community health or community-based or family physicians or family practice or health aides or health centers or health centres or health facilities or health personnel or health posts or health provider(s) or health services or health staff or health systems or health workers or hospital or medical assistants or medical auxiliaries or medical student(s) or pharmacists or physician(s) or primary health care or primary medical care or private practitioner or public health facilities or public physicians or public services or regional hospitals or rural health or teaching hospitals or voluntary workers)

AND
(health care quality or health education or impact or implementation or intervention or participatory action or practice guidelines or program evaluation or quality assurance or seminar or standard management or standard treatment or standards or structured care or training or treatment guidelines or treatment practices or utilization or workshops)
AND
((Afghanistan or Bangladesh or Benin or Bhutan or Burkina Faso or Burundi or Cambodia or Cameroon or Central African Republic or Chad or Comoros or Congo or the Democratic Republic of Congo or Cote d'Ivoire or Eritrea or Ethiopia or The Gambia or Ghana or Guinea or Guinea-Bissau or Haiti or India or Kenya or Democratic Republic of Korea or Kyrgyszstan Republic or Lao PDR or Lesotho or Liberia or Madagascar or Malawi or Mali or Mauritania or Moldova or Mongolia or Mozambique or Myanmar or Nepal or Nicaragua or Niger or Nigeria or North Korea or Pakistan or Papua New Guinea or Rwanda or Sao Tome and Principe or Senegal or Sierra Leone or Solomon Islands or Somalia or Sudan or Tajikistan or Tanzania or Timor-Leste or Togo or Uganda or Uzbekistan or Vietnam or Yemen or Republic of Yemen or Zaire or Zambia or Zimbabwe) or (Albania or Algeria or Angola or Armenia or Azerbaijan or Belarus or Bolivia or Bosnia and Herzegovina or Brazil or Bulgaria or Cape Verde or China or Colombia or Cuba or Djibouti or Dominican Republic or Ecuador or Egypt or Arab Republic of Egypt or El Salvador or Fiji or Georgia or Guatemala or Guyana or Honduras or Indonesia or Iran or Islamic Republic of Iran or Iraq or Jamaica or Jordan or Kazakhstan or Kiribati or Macedonia or FYR of Macedonia or Former Yugoslav Republic of Macedonia or Maldives or Marshall Islands or Micronesia or Federated States of Micronesia or Morocco or Namibia or Paraguay or Peru or Philippines or Romania or Samoa or Serbia and Montenegro or Sri Lanka or Suriname or Swaziland or Syrian Arab Republic or Syria or Thailand or Tonga or Tunisia or Turkmenistan or Ukraine or Vanuatu or West Bank and Gaza) or (American Samoa or Antigua and Barbuda or Argentina or Barbados or Belize or Botswana or Chile or Costa Rica or Croatia or Czech Republic or Dominica or Equatorial Guinea or Estonia or Gabon or Grenada or Hungary or Latvia or Lebanon or Libya or Lithuania or Malaysia or Mauritius or Mayotte or Mexico or Northern Mariana Islands or Oman or Palau or Panama or Poland or Russian Federation or Seychelles or Slovak Republic or South Africa or St. Kitts and Nevis or St. Lucia or St. Vincent and the Grenadines or Trinidad and Tobago or Turkey or Uruguay or Venezuela or Bolivarian Republic of Venezuela))

We added search terms recommended by World Bank colleagues and re-ran the electronic search. The strategy retrieved 68 of the gold standard articles, resulting in a final sensitivity of 81% (68/84).

With the World Bank terms added, the strategies for searching Campbell Collaboration, CINAHL, Cochrane Library, Dissertation Abstracts, EconLit, Eldis, EMBASE, ERIC, HMIC, MEDLINE (via PubMed), Science Citation Index/Web of Science/SSCI/Global Health, and Sociological Abstracts are listed below:

AFGHANISTAN OR BANGLADESH OR BENIN OR BHUTAN OR BURKINAFASO OR BURUNDI OR CAMBODIA OR CAMEROON OR CENTRAL AFRICAN REPUBLIC OR CHAD OR COMOROS OR CONGO OR COTE D'IVOIRE OR ERITREA OR COTE DIVOIRE OR ETHIOPIA OR GAMBIA OR GHANA OR GUINEA OR HAITI OR INDIA OR KENYA OR KOREA OR KYRGYSZSTAN OR LAO OR LAOS OR LESOTHO OR LIBERIA OR MADAGASCAR OR MALAWI OR MALI OR MAURITANIA OR MOLDOVA OR MONGOLIA OR MOZAMBIQUE OR MYANMAR OR NEPAL OR NICARAGUA OR NIGER OR NIGERIA OR PAKISTAN OR RWANDA OR SAO TOME OR SENEGAL OR SIERRA LEONE OR SOLOMON ISLANDS OR SOMALIA OR SUDAN OR TAJIKISTAN OR TANZANIA OR TIMOR OR TOGO OR UGANDA OR UZBEKISTAN OR VIETNAM OR YEMEN OR ZAIRE OR ZAMBIA OR ZIMBABWE OR ALBANIA OR ALGERIA OR ANGOLA OR ARMENIA OR AZERBAIJAN OR BELARUS OR BOLIVIA OR BOSNIA OR HERZEGOVINA OR BRAZIL OR BULGARIA OR CAPE VERDE OR CHINA OR COLOMBIA OR CUBA OR DJIBOUTI OR DOMINICAN REPUBLIC OR ECUADOR OR EGYPT OR EL SALVADOR OR FIJI OR GEORGIA OR GUATEMALA OR GUYANA OR HONDURAS OR INDONESIA OR IRAN OR IRAQ OR JAMAICA OR JORDAN OR KAZAKHSTAN OR KIRIBATI OR MACEDONIA OR MALDIVES OR MARSHALL ISLANDS OR MICRONESIA OR MOROCCO OR NAMIBIA OR PARAGUAY OR PERU OR PHILIPPINES OR ROMANIA OR SAMOA OR SERBIA OR MONTENEGRO OR SRI LANKA OR SURINAME OR SWAZILAND OR SYRIA OR THAILAND OR TONGA OR TUNISIA OR TURKMENISTAN OR UKRAINE OR VANUATU OR WEST BANK OR GAZA OR ANTIGUA OR BARBUDA OR ARGENTINA OR BARBADOS OR BELIZE OR BOTSWANA OR CHILE OR COSTA RICA OR CROATIA OR CZECH REPUBLIC OR DOMINICA OR EQUATORIAL GUINEA OR ESTONIA OR GABON OR GRENADA OR HUNGARY OR LATVIA OR LEBANON OR LIBYA OR LITHUANIA OR MALAYSIA OR MAURITIUS OR MAYOTTE OR MEXICO OR MARIANA ISLANDS OR OMAN OR PALAU OR PANAMA OR POLAND OR RUSSIAN FEDERATION OR SEYCHELLES OR SLOVAK REPUBLIC OR SOUTH AFRICA OR ST. KITTS OR ST KITTS OR NEVIS OR ST. LUCIA OR ST LUCIA OR ST. VINCENT OR ST VINCENT OR GRENADINES OR TRINIDAD OR TOBAGO OR TURKEY OR URUGUAY OR VENEZUELA OR ASIA OR WEST INDIES OR POLYNESIA OR MICRONESIA OR MIDDLE EAST OR AFRICA OR LATIN AMERICA OR CENTRAL AMERICA OR SOUTH AMERICA OR CARIBBEAN OR WEST INDIES REGION OR HISPANOLA OR SOUTHEAST ASIA OR SUBSAHARAN AFRICA OR EASTERN EUROPE OR HISPANIOLA OR BURMA OR EAST TIMOR OR CONGO OR GUIANA OR CZECHOSLOVAKIA OR LAOS OR NORTH KOREA OR IVORY COAST OR REPUBLICOF GEORGIA OR YEMEN OR ZAIRE OR SLOVAKIA OR SOVIET UNION OR SURINAM OR USSR OR SAMOA OR YUGOSLAVIA OR DEVELOPING NATIONS OR DEVELOPING COUNTRIES OR LESS DEVELOPED NATIONS OR LESS DEVELOPED COUNTRIES OR THIRD WORLD NATIONS OR THIRD WORLD COUNTRIES OR UNDERDEVELOPED NATIONS UNDERDEVELOPED COUNTRIES OR POOR NATIONS OR POOR COUNTRIES OR POOR ECONOMIES

AND

DOCTOR OR DOCTORS OR PERSONNEL OR WORKER OR WORKERS OR STUDENT OR STUDENTS OR PHYSICIAN OR PHYSICIANS OR PROVIDER OR PROVIDERS OR PARAMEDIC* OR MIDWIFE OR MIDWIVES OR PROFESSIONAL OR NURSE OR NURSES OR PRACTITIONER OR PRACTITIONERS OR THERAPIST OR THERAPISTS OR ATTENDANT OR ATTENDANTS OR HOSPITAL OR HOSPITALS OR POST OR POSTS OR
CENTER OR FACILITIES OR FACILITY OR SERVICE OR COMMUNITY

AND

EDUCATION OR INSERVICE OR TRAINING OR AUDIT OR PRACTICE OR
INTERVENTION OR ASSURANCE OR KNOWLEDGE OR ATTITUDE OR
COVERAGE OR OCCUPATION OR OCCUPATIONAL OR PLANNING OR VOCATION OR VOCATIONAL OR CAREER OR EMPLOYEE OR
JOB SATISFACTION OR MENTORING OR WORK OR WORKING OR WAGE
OR WAGES OR BENEFIT OR SAFETY OR POPULATION OR DEMOGRAPHY
OR INCENTIVES OR HUMAN ADAPTIBILITY OR PREVENTION OR
IMMUNIZATIONS OR IMMUNIZE OR VACCINATIONS OR VACCINATE
OR VACCINE OR DISEASE OR TREATMENT OR THERAPY OR PREVENTIVE
OR SANITATION OR IMPROVEMENT OR ECONOMIC OR INTEGRATION OR
INITIATIVES OR STRATEGIC PLANNING OR LEADERSHIP OR SECTOR
WIDE OR DECENTRALIZATION OR DECONCENTRATION
OR AUTONOMIZATION OR AUTHORITY OR FEE OR FEES OR PAYMENT OR
RISK OR INSURANCE OR FINANCING OR COPAYMENT OR CASH OR VOUCHERS
OR COST OR EFFICIENT OR EFFICIENCY OR SAVINGS ACCOUNT OR
MOBILIZATION OR EMPOWERMENT OR ENGAGEMENT
OR COALITION OR ORGANIZATION OR CONSUMER OR PATIENT OR
REPORT CARD OR SCORECARDS OR PATIENT SATISFACTION OR
MARKETING OR FRANCHISE OR DEMAND
CONTRACT OR CONTRACTING OR REGULATION OR ACCREDITATION OR
LICENSING OR SERVICE AGREEMENT OR PARTNERSHIP OR
STEWARDSHIP OR PERFORMANCE OR QUALITY OR BENCHMARK OR BENCHMARKING OR
OR REORGANIZATION OR REDESIGN OR REENGINEERING OR TEAM OR
TEAMS OR GUIDELINE OR SUPPLYCHAIN OR CERTIFICATE OF NEED OR ASSESSMENT OR MOTIVATION OR COMPETENCE OR STAFF OR
STAFFING OR SKILL OR PRODUCTIVITY

CINAHL

CINAHL - Cumulative Index to Nursing & Allied Health Literature

1982 to September Week 3 2006

Change Database | CDC Information Center Reference staff | Help | LOGOFF

#	Search History	Results	Display
1	(afghanistan or bangladesh or benin or burkina faso or burundi or cambodia or central african republic or chad or comoros or congo or cote d'ivoire or eritrea or ethiopia or ivory coast or gambia or ghana or guinea or haiti or india or kenya or korea or kyrgyszstan or lao or laos or lesotho or liberia or madagascar or malawi or mali or mauritania or modova or mongoli or mozambique or mayanmar or nepal or nicaragua or niger or nigeria or pakistan or rwanda or sao tome or senegal or sierra leone or solomon islands or somalia or sudan or tajikistan or tanzania or timor or togo or uganda or uzbekistan or vietnam or yemen or zaire or zambia or zimbabwe or albania or algeria or angola or armenia or azerbaijan or belarus or bolivia or bosnia or herzegovina or brazil or bulgaria or cape verde or china or colombia or cuba or djibouti or dominican republic or ecuador or egypt or el salvador or fiji or georgia or guatemala or guyana or honduras or indonesia or iran or iraq or jamaica or jordan or kazakhstan or kiribati or macedonia or maldives or marshall islands or micronesia or morocco or namibia or paraguay or peru or philippines or romania or samoa or serbia or montenegro or sri lanka or suriname or swaziland or syria).mp.	25776	DISPLAY
2	(thailand or tonga or tunisia or turkmenistan or ukraine or vanuatu or west bank or gaza or antigua or barbuda or argentina or barbados or belize or botswana or chile or costa rica or croatia or czech republic or dominica or equatorial guinea or estonia or gabon or grenada or hungary or latvia or lebanon or libya or lithuania or libya or malaysia or mauritius or mayotte or mexico).mp.	6419	DISPLAY
3	(mariana islands or oman or palau or panama or poland or russian federation or seychelles or slovak republic or south africa or st kitts or nevis or st lucia or st vincent or grenadines or trinidad or tobago or turkey or uruguay or venezuela or asia or west indies or polynesia or micronesia or middle east or africa or latin	11526	DISPLAY

CONTRACT

	america or central america or south america or caribbean).mp.		
4	(((((developing adj (nation$1 or country or countries)) or less developed) adj (country or counties)) or third world) adj (nation$1 or country or countries or economies)).mp.	57	DISPLAY
5	(hispanola or southeast asia or saharan africa or eastern europe or hispaniola or burma or east timor or congo or guiana or czechoslovakia or laos or north korea or yemen or republic of georgia or zaire or slovakia or soviet union or surinam or ussr or yugoslavia).mp.	1522	DISPLAY
6	(center$1 or facilities or facility or service$1 or community or education or inservice or training or audit or practice or intervention$1 or assurance or knowledge or attitude$1 or coverage or occupation$1 or occupational or planning or vocation or vocational or career or employee or job satisfaction or mentoring or work or working or wage or wages or benefit$1 or safety or population or demography or incentives or human adaptibility or prevention or immunizations or immunize or vaccinations or vaccinate or vaccine$1 or disease or treatment or therapy or preventive or sanitation or improvement or economic$1 or integration or initiatives or strategic planning or leadership or sector wide or decentralization or deconcentration or autonomization or authority or fee or fees or payment or risk or insurance or financing or copayment or cash or vouchers or cost or efficient or efficiency or savings account$1 or mobilzation or empowerment or engagement or coalition$1 or organization$1 or consumer or patient or report card$1 or scorecards or patient satisfaction or marketing or franchise or demand or contract or contracting or regulation or accreditation or licensing or service agreement$1 or partnership$1 or stewardship or performance or quality or benchmark or benchmarking or regorganization or redesign or re-engineering or team or teams or guideline$1 or supply chain or certificate of need or assessment or motivation or competence or staff or staffing or skill or productivity).mp.	887056	DISPLAY
7	(doctor$1 or personnel or worker$1 or student$1 or physician$1 or provider$1 or paramedical or midwife or midwives or professional$1 or nurse$1 or practitioner$1 or therapist$1 or attendant$1 or hospital$1 or	439530	DISPLAY

	post$1).mp.		
8	1 or 2 or 3 or 4 or 5	41726	DISPLAY
9	8 and "9".mp. [mp=title, subject heading word, abstract, instrumentation]	955	DISPLAY
10	6 and 9	857	DISPLAY
11	limit 10 to yr="2005 - 2006"	201	DISPLAY
12	limit 10 to yr="2006"	52	DISPLAY
13	limit 10 to yr="2005"	149	DISPLAY
14	limit 10 to yr="2004"	142	DISPLAY
15	limit 10 to yr="2003"	147	DISPLAY
16	limit 10 to yr="2002"	81	DISPLAY
17	from 12 keep 1-52	52	DISPLAY
18	from 13 keep 1-149	149	DISPLAY
19	from 14 keep 1-142	142	DISPLAY
20	from 15 keep 1-147	147	DISPLAY
21	from 16 keep 1-81	81	DISPLAY
22	8 and 7 and 6	15389	DISPLAY
23	((doctor$1 or personnel or worker$1 or student$1 or physician$1 or provider$1 or paramedical or midwife or midwives or professional$1 or nurse$1 or practitioner$1 or therapist$1 or attendant$1 or hospital$1 or post$1) and (center$1 or facilities or facility or service$1 or community or education or inservice or training or audit or practice or intervention$1 or assurance or knowledge or attitude$1 or coverage or occupation$1 or occupational or planning or vocation or vocational or career or employee or job satisfaction or mentoring or work or working or wage or wages or benefit$1 or safety or population or demography or incentives or human adaptibility or prevention or immunizations or immunize or vaccinations or vaccinate or vaccine$1 or disease or treatment or therapy or preventive or sanitation or improvement or economic$1 or integration or initiatives or strategic planning or leadership or sector wide or decentralization or deconcentration or autonomization or authority or fee or fees or payment or risk or insurance or financing or copayment or cash or vouchers or cost or efficient or efficiency or savings	789	DISPLAY

	account$1 or mobilzation or empowerment or engagement or coalition$1 or organization$1 or consumer or patient or report card$1 or scorecards or patient satisfaction or marketing or franchise or demand or contract or contracting or regulation or accreditation or licensing or service agreement$1 or partnership$1 or stewardship or performance or quality or benchmark or benchmarking or regorganization or redesign or re-engineering or team or teams or guideline$1 or supply chain or certificate of need or assessment or motivation or competence or staff or staffing or skill or productivity) and (afghanistan or bangladesh or benin or burkina faso or burundi or cambodia or central african republic or chad or comoros or congo or cote d'ivoire or eritrea or ethiopia or ivory coast or gambia or ghana or guinea or haiti or india or kenya or korea or kyrgyszstan or lao or laos or lesotho or liberia or madagascar or malawi or mali or mauritania or modova or mongoli or mozambique or mayanmar or nepal or nicaragua or niger or nigeria or pakistan or rwanda or sao tome or senegal or sierra leone or solomon islands or somalia or sudan or tajikistan or tanzania or timor or togo or uganda or uzbekistan or vietnam or yemen or zaire or zambia or zimbabwe or albania or algeria or angola or armenia or azerbaijan or belarus or bolivia or bosnia or herzegovina or brazil or bulgaria or cape verde or china or colombia or cuba or djibouti or dominican republic or ecuador or egypt or el salvador or fiji or georgia or guatemala or guyana or honduras or indonesia or iran or iraq or jamaica or jordan or kazakhstan or kiribati or macedonia or maldives or marshall islands or micronesia or morocco or namibia or paraguay or peru or philippines or romania or samoa or serbia or montenegro or sri lanka or suriname or swaziland or syria or (thailand or tonga or tunisia or turkmenistan or ukraine or vanuatu or west bank or gaza or antigua or barbuda or argentina or barbados or belize or botswana or chile or costa rica or croatia or czech republic or dominica or equatorial guinea or estonia or gabon or grenada or hungary or latvia or lebanon or libya or lithuania or libya or malaysia or mauritius or mayotte or mexico) or (mariana islands or oman or palau or panama or poland or russian federation or seychelles or slovak republic or south africa or st kitts or nevis or st lucia or st vincent or grenadines or trinidad or tobago or		

	turkey or uruguay or venezuela or asia or west indies or polynesia or micronesia or middle east or africa or latin america or central america or south america or caribbean) or (((((developing adj (nation$1 or country or countries)) or less developed) adj (country or counties)) or third world) adj (nation$1 or country or countries or economies)) or (hispanola or southeast asia or saharan africa or eastern europe or hispaniola or burma or east timor or congo or guiana or czechoslovakia or laos or north korea or yemen or republic of georgia or zaire or slovakia or soviet union or surinam or ussr or yugoslavia))).ti.		
24	limit 23 to yr="2006 - 2007"	39	DISPLAY
25	limit 23 to yr="2004 - 2005"	177	DISPLAY
26	limit 23 to yr="2002 - 2003"	158	DISPLAY
27	limit 23 to yr="2000 - 2001"	140	DISPLAY
28	limit 23 to yr="1996 - 1999"	117	DISPLAY
29	limit 23 to yr="1990 - 1995"	99	DISPLAY
30	limit 23 to yr="1980 - 1989"	56	DISPLAY
31	limit 23 to yr="1900 - 1979"	1	DISPLAY
32	24 or 25 or 26 or 27 or 28 or 29 or 30 or 31	787	DISPLAY
33	from 24 keep 1-39	39	DISPLAY
34	from 25 keep 1-177	177	DISPLAY
35	from 26 keep 1-158	158	DISPLAY
36	from 27 keep 1-140	140	DISPLAY
37	from 28 keep 1-117	117	DISPLAY
38	29 or 30 or 31	156	DISPLAY
**39**	**from 38 keep 1-156**	**156**	**DISPLAY**

Combine Searches | Delete Searches | Save Search/Alert

Advanced Search | Basic Search | Find Citation | More Fields | Search Tools

Keyword

Enter **Keyword** or phrase (use "$" for truncation):

☑ Map Term to Subject Heading

(afghanistan or bangladesh or benin or burkina faso or burundi or cambodia or central african republic or chad or comoros or congo or cote d'ivoire or eritrea or ethiopia or ivory coast or gambia or ghana or guinea or haiti or india or kenya or korea or kyrgyszstan or lao or laos or lesotho or liberia or madagascar or malawi or mali or mauritania or modova or mongoli or mozambique or mayanmar or nepal or nicaragua or niger or nigeria or pakistan or rwanda or sao tome or senegal or sierra leone or solomon islands or somalia or sudan or tajikistan or tanzania or timor or togo or uganda or uzbekistan or vietnam or yemen or zaire or zambia or zimbabwe or albania or algeria or angola or armenia or azerbaijan or belarus or bolivia or bosnia or herzegovina or brazil or bulgaria or cape verde or china or colombia or cuba or djibouti or dominican republic or ecuador or egypt or el salvador or fiji or georgia or guatemala or guyana or honduras or indonesia or iran or iraq or jamaica or jordan or kazakhstan or kiribati or macedonia or maldives or marshall islands or micronesia or morocco or namibia or paraguay or peru or philippines or romania or samoa or serbia or montenegro or sri lanka or suriname or swaziland or syria or thailand or tonga or tunisia or turkmenistan or ukraine or vanuatu or west bank or gaza or antigua or barbuda or argentina or barbados or belize or botswana or chile or costa rica or croatia or czech republic or dominica or equatorial guinea or estonia or gabon or grenada or hungary or latvia or lebanon or libya or lithuania or libya or malaysia or mauritius or mayotte or mexico or mariana islands or oman or palau or panama or poland or russian federation or seychelles or slovak republic or south africa or st kitts or nevis or st lucia or st vincent or grenadines or trinidad or tobago or turkey or uruguay or venezuela or asia or west indies or polynesia or micronesia or middle east or africa or latin america or central america or south america or caribbean or developing nations or developing countries or developing nation or developing country or less developed countries or less developed country or third world or hispanola or southeast asia or saharan africa or eastern europe or hispaniola or burma or east timor or congo or guiana or czechoslovakia or laos or north korea or yemen or republic of georgia or zaire or slovakia or soviet union or surinam or ussr or yugoslavia)
43674 edit delete

#4 (doctor* or personnel or worker* or student* or physician* or provider* or paramedical or midwife or midwives or professional* or nurse* or practitioner* or therapist* or attendant* or hospital* or post* or center* or facilities or facility or service* or community or education or inservice or training or audit or practice or intervention* or assurance or knowledge or attitude* or coverage or occupation* or occupational or planning or vocation or vocational or career or employee or job satisfaction or mentoring or work or working or wage or wages or benefit* or safety or population or demography or incentives or human adaptibility or prevention or immunizations or immunize or vaccinations or vaccinate or vaccine* or disease or treatment or therapy or preventive or sanitation or improvement or economic* or integration or initiatives or strategic planning or leadership or sector wide or decentralization or deconcentration or autonomization or authority or fee or fees or payment or risk or insurance or financing or copayment or cash or vouchers or cost or efficient or efficiency or savings account* or mobilzation or empowerment or engagement or coalition* or organization* or consumer or patient or report card* or scorecards or patient satisfaction or marketing or franchise or demand or contract or contracting or regulation or accreditation or licensing or service agreement* or partnership* or stewardship or performance or quality or benchmark or benchmarking or regorganization or redesign or re-engineering or team or teams or guideline* or supply chain or certificate of need or assessment or motivation or competence or staff or staffing or skill or productivity):ti,ab,kw 433159 edit delete

#5 (#3 AND #4), from 1980 to 2006 39852 edit delete

#6 (doctor* or personnel or worker* or student* or physician* or provider* or paramedical or midwife or midwives or professional* or nurse* or practitioner* or therapist* or attendant* or hospital* or post* or center* or facilities or facility or service* or community or education or inservice or training or audit or

practice or intervention* or assurance or knowledge or attitude* or coverage or occupation* or occupational or planning or vocation or vocational or career or employee or job satisfaction or mentoring or work or working or wage or wages or benefit* or safety or population or demography or incentives or human adaptibility or prevention or immunizations or immunize or vaccinations or vaccinate or vaccine* or disease or treatment or therapy or preventive or sanitation or improvement or economic* or integration or initiatives or strategic planning or leadership or sector wide or decentralization or deconcentration or autonomization or authority or fee or fees or payment or risk or insurance or financing or copayment or cash or vouchers or cost or efficient or efficiency or savings account* or mobilzation or empowerment or engagement or coalition* or organization* or consumer or patient or report card* or scorecards or patient satisfaction or marketing or franchise or demand or contract or contracting or regulation or accreditation or licensing or service agreement* or partnership* or stewardship or performance or quality or benchmark or benchmarking or regorganization or redesign or re-engineering or team or teams or guideline* or supply chain or certificate of need or assessment or motivation or competence or staff or staffing or skill or productivity):ti 260718 edit delete

#7 (afghanistan or bangladesh or benin or burkina faso or burundi or cambodia or central african republic or chad or comoros or congo or cote d'ivoire or eritrea or ethiopia or ivory coast or gambia or ghana or guinea or haiti or india or kenya or korea or kyrgyszstan or lao or laos or lesotho or liberia or madagascar or malawi or mali or mauritania or modova or mongoli or mozambique or mayanmar or nepal or nicaragua or niger or nigeria or pakistan or rwanda or sao tome or senegal or sierra leone or solomon islands or somalia or sudan or tajikistan or tanzania or timor or togo or uganda or uzbekistan or vietnam or yemen or zaire or zambia or zimbabwe or albania or algeria or angola or armenia or azerbaijan or belarus or bolivia or bosnia or herzegovina or brazil or bulgaria or cape verde or china or colombia or cuba or djibouti or dominican republic or ecuador or egypt or el salvador or fiji or georgia or guatemala or guyana or honduras or indonesia or iran or iraq or jamaica or jordan or kazakhstan or kiribati or macedonia or maldives or marshall islands or micronesia or morocco or namibia or paraguay or peru or philippines or romania or samoa or serbia or montenegro or sri lanka or suriname or swaziland or syria or thailand or tonga or tunisia or turkmenistan or ukraine or vanuatu or west bank or gaza or antigua or barbuda or argentina or barbados or belize or botswana or chile or costa rica or croatia or czech republic or dominica or equatorial guinea or estonia or gabon or grenada or hungary or latvia or lebanon or libya or lithuania or libya or malaysia or mauritius or mayotte or mexico or mariana islands or oman or palau or panama or poland or russian federation or seychelles or slovak republic or south africa or st kitts or nevis or st lucia or st vincent or grenadines or trinidad or tobago or turkey or uruguay or venezuela or asia or west indies or polynesia or micronesia or middle east or africa or latin america or central america or south america or caribbean or developing nations or developing countries or developing nation or developing country or less developed countries or less developed country or third world or hispanola or southeast asia or saharan africa or eastern europe or hispaniola or burma or east timor or congo or guiana or czechoslovakia or laos or north korea or yemen or republic of georgia or zaire or slovakia or soviet union or surinam or ussr or yugoslavia):ti 3615 edit delete

#8 (#6 AND #7), from 1980 to 2006 2115 edit delete

#9 MeSH descriptor Health Personnel explode all trees with qualifier: ED 536 edit delete

#10 (#7 AND #9), from 1980 to 2006 22 edit delete

#11 (TRAINING):ti,ab,kw 14650 edit delete

#12 (#11 AND #7) 158 edit delete

#13 (#12 AND NOT #10) 141 ed

Dissertation Abstracts

Set Items Description
S1 2115 AFGHANISTAN OR BANGLADESH OR BENIN OR BHUTAN OR BURKINA()FASO OR BURUNDI OR CAMBODIA OR CAMEROON
S2 20062 CENTRAL()AFRICAN()REPUBLIC OR CHAD OR COMOROS OR CONGO OR - COTE()D'IVOIRE OR ERITREA OR COTE()D()IVOIRE OR ETHIOPIA OR G- AMBIA OR GHANA OR GUINEA OR HAITI OR INDIA OR KENYA OR KOREA - OR KYRGYSZSTAN OR LAO OR LAOS OR LESOTHO OR LIBERIA
S3 9456 MADAGASCAR OR MALAWI OR MALI OR MAURITANIA OR MOLDOVA OR MONGOLIA OR MOZAMBIQUE OR MYANMAR OR NEPAL OR NICARAGUA OR NIGER OR NIGERIA OR PAKISTAN OR RWANDA OR SAO()TOME OR SENEGAL OR SIERRA()LEONE OR SOLOMON()ISLANDS OR SOMALIA OR SUDAN
S4 11614 TAJIKISTAN OR TANZANIA OR TIMOR OR TOGO OR UGANDA OR UZBEKISTAN OR VIETNAM OR YEMEN OR ZAIRE OR ZAMBIA OR ZIMBABWE OR A- LBANIA OR ALGERIA OR ANGOLA OR ARMENIA OR AZERBAIJAN OR BELA- RUS OR BOLIVIA OR BOSNIA OR HERZEGOVINA OR BRAZIL
S5 31690 BULGARIA OR CAPE()VERDE OR CHINA OR COLOMBIA OR CUBA OR DJIBOUTI OR DOMINICAN()REPUBLIC OR ECUADOR OR EGYPT OR EL()SALVADOR OR FIJI OR GEORGIA OR GUATEMALA OR GUYANA OR HONDURAS OR INDONESIA OR IRAN OR IRAQ OR JAMAICA OR JORDAN
S6 7505 KAZAKHSTAN OR KIRIBATI OR MACEDONIA OR MALDIVES OR MARSHALL()ISLANDS OR MICRONESIA OR MOROCCO OR NAMIBIA OR PARAGUAY OR PERU OR PHILIPPINES OR ROMANIA OR SAMOA OR SERBIA OR MONTENEGRO OR SRI()LANKA OR SURINAME OR SWAZILAND OR SYRIA
S7 4166 THAILAND OR TONGA OR TUNISIA OR TURKMENISTAN OR UKRAINE OR VANUATU OR WEST()BANK OR GAZA
S8 8826 ANTIGUA OR BARBUDA OR ARGENTINA OR BARBADOS OR BELIZE OR BOTSWANA OR CHILE OR COSTA()RICA OR CROATIA OR CZECH()REPUBLIC OR DOMINICA OR EQUATORIAL()GUINEA OR ESTONIA OR GABON OR GRENADA OR HUNGARY OR LATVIA OR LEBANON OR LIBYA OR LITHUANIA
S9 19519 MALAYSIA OR MAURITIUS OR MAYOTTE OR MEXICO OR MARIANA()ISLANDS OR OMAN OR PALAU OR PANAMA OR POLAND OR RUSSIAN()FEDERATION OR SEYCHELLES OR SLOVAK()REPUBLIC OR SOUTH()AFRICA OR ST()KITTS OR ST(1W)KITTS OR NEVIS OR ST()LUCIA OR ST(1W)LUCIA
S10 4592 ST()VINCENT OR GRENADINES OR TRINIDAD OR TOBAGO OR TURKEY - OR URUGUAY OR VENEZUELA
S11 23358 ASIA OR WEST()INDIES OR POLYNESIA OR MICRONESIA OR MIDDLE EAST OR AFRICA OR LATIN AMERICA OR CENTRAL AMERICA OR SOUTH A- MERICA OR CARIBBEAN OR WEST INDIES REGION OR HISPA?OLA OR SOUTHEAST ASIA OR SUB()SAHARAN()AFRICA OR EASTERN()EUROPE
S12 67 HISPANIOLA
S13 6235 BURMA OR EAST()TIMOR OR CONGO OR GUIANA OR CZECHOSLOVAKIA -OR LAOS OR NORTH()KOREA OR IVORY()COAST OR REPUBLIC(1W)GEORGIA OR YEMEN OR ZAIRE OR SLOVAKIA OR SOVIET()UNION OR SURINAM - OR USSR OR SAMOA OR YUGOSLAVIA
S14 6743 DEVELOPING()(NATION? ? OR COUNTRIES) OR LESS()DEVELOPED()(-

NATION? ? OR COUNTRIES) OR THIRD()WORLD()(NATION? ? OR COUNTR-
IES) OR UNDER()DEVELOPED()(NATION? ? OR COUNTRIES) OR POOR()-
(NATION? ? OR COUNTRIES OR ECONOMIES)

S15 118262 S1 OR S2 OR S3 OR S4 OR S5 OR S6 OR S7 OR S8 OR S9 OR S10 -
OR S11 OR S12 OR S13 OR S14

S16 333882 DOCTOR? ? OR PERSONNEL OR WORKER? ? OR STUDENT? ? OR
PHYSICIAN? ? OR PROVIDER? ? OR PARAMEDICAL OR MIDWIFE OR MIDWIVES -
OR PROFESSIONAL? ? OR NURSE? ? OR PRACTITIONER? ? OR THERAPIS-
T? ? OR ATTENDANT? ? OR HOSPITAL? ? OR POST? ?

S17 210491 CENTER? ? OR FACILITIES OR FACILITY OR SERVICE? ? OR COMMUNITY

S18 464780 S16 OR S17

S19 40393 S18 AND S15

S20 23358 ASIA OR WEST()INDIES OR POLYNESIA OR MICRONESIA OR MIDDLE
EAST OR AFRICA OR LATIN AMERICA OR CENTRAL AMERICA OR SOUTH A-
MERICA OR CARIBBEAN OR WEST INDIES REGION OR HISPA?OLA OR SOUTHEAST
ASIA OR SUB()SAHARAN()AFRICA OR EASTERN()EUROPE

S21 67 HISPANIOLA

S22 6235 BURMA OR EAST()TIMOR OR CONGO OR GUIANA OR CZECHOSLOVAKIA -
OR LAOS OR NORTH()KOREA OR IVORY()COAST OR REPUBLIC(1W)GEORGIA OR
YEMEN OR ZAIRE OR SLOVAKIA OR SOVIET()UNION OR SURINAM -
OR USSR OR SAMOA OR YUGOSLAVIA

S23 6743 DEVELOPING()(NATION? ? OR COUNTRIES) OR LESS()DEVELOPED()(-
NATION? ? OR COUNTRIES) OR THIRD()WORLD()(NATION? ? OR COUNTR-
IES) OR UNDER()DEVELOPED()(NATION? ? OR COUNTRIES) OR POOR()-
(NATION? ? OR COUNTRIES OR ECONOMIES)

S24 555815 EDUCATION OR INSERVICE OR TRAINING OR AUDIT OR PRACTICE OR
INTERVENTION? ? OR ASSURANCE OR KNOWLEDGE OR ATTITUDE? ?OR CO-
VERAGE OR OCCUPATION? ? OR OCCUPATIONAL OR PLANNING OR VOCAT-
ION OR VOCATIONAL OR CAREER OR EMPLOYEE

S25 398063 JOB()SATISFACTION OR MENTORING OR WORK OR WORKING OR WAGE -
OR WAGES OR BENEFIT? ? OR SAFETY OR POPULATION OR DEMOGRAPHY -
OR INCENTIVES OR HUMAN()ADAPTIBILITY OR PREVENTION OR IMMUNIZ-
ATIONS OR IMMUNIZE OR VACCINATIONS OR VACCINATE

S26 376259 VACCINE? ? OR DISEASE OR TREATMENT OR THERAPY OR PREVENTIVE
OR SANITATION OR IMPROVEMENT OR ECONOMIC? ? OR INTEGRATION OR
INITIATIVES OR STRATEGIC()PLANNING OR LEADERSHIP OR SECTOR()-
WIDE OR DECENTRALIZATION OR DECONCENTRATION

S27 202821 AUTONOMIZATION OR AUTHORITY OR FEE OR FEES OR PAYMENT OR RISK
OR INSURANCE OR FINANCING OR COPAYMENT OR CASH OR VOUCHERS
OR COST OR EFFICIENT OR EFFICIENCY OR SAVINGS()ACCOUNT? ? OR
MOBILIZATION OR EMPOWERMENT OR ENGAGEMENT

S28 138203 COALITION? ? OR ORGANIZATION? ? OR CONSUMER OR PATIENT OR -
REPORT()CARD? ? OR SCORECARDS OR PATIENT()SATISFACTION OR MAR-
KETING OR FRANCHISE OR DEMAND

S29 278104 CONTRACT OR CONTRACTING OR REGULATION OR ACCREDITATION OR -
LICENSING OR SERVICE()AGREEMENT ? ? OR PARTNERSHIP? ? OR STEWARDSHIP OR
PERFORMANCE OR QUALITY OR BENCHMARK OR BENCHMARKING

OR REORGANIZATION OR REDESIGN OR RE()ENGINEERING OR TEAM
S30 151156 TEAMS OR GUIDELINE? ? OR SUPPLY()CHAIN OR CERTIFICATE(1W)NEED OR ASSESSMENT OR MOTIVATION OR COMPETENCE OR STAFF OR STAFFING OR SKILL OR PRODUCTIVITY
S31 333882 DOCTOR? ? OR PERSONNEL OR WORKER? ? OR STUDENT? ? OR PHYSICIAN? ? OR PROVIDER? ? OR PARAMEDICAL OR MIDWIFE OR MIDWIVES - OR PROFESSIONAL? ? OR NURSE? ? OR PRACTITIONER? ? OR THERAPIS- T? ? OR ATTENDANT? ? OR HOSPITAL? ? OR POST? ?
S32 938790 STUDY OR STUDIES
S33 210491 CENTER? ? OR FACILITIES OR FACILITY OR SERVICE? ? OR COMMUNITY
S34 21471 CHILDREN(2W) HOSPITAL? ?/TI OR COMMUNITY()HEALTH()SERVICES- /DE OR DOCTOR/TI,AB OR DOCTORS/TI,AB OR HEALTH PERSONNEL! OR HEALTH POSTS/TI,AB OR HEALTH()SERVICES()RESEARCH/DE OR HEALTH- ()WORKERS/TI,AB OR HOSPITAL/TI,AB OR HOSPITALS/TI,AB
S35 11 HEALTH()POSTS/TI,AB
S36 12050 MEDICAL()STUDENTS/TI OR OBSTETRIC()CARE/TI OR PHARMACEUTICAL()SERVICES/DE OR PHARMACY()SERVICE()HOSPITAL/DE OR PHYSICIANS/TI,AB OR PRIMARY()HEALTH()CARE/TI,AB OR PRIMARY()HEALTH()C- ENTERS/TI,AB OR PROVIDERS/TI,AB
S37 70608 AUDIT/TI OR EDUCATION, CONTINUING! OR HEALTH CARE COSTS! OR HEALTH EDUCATION! OR HEALTH KNOWLEDGE, ATTITUDES, PRACTICE! - OR IMPACT/TI OR INSERVICE, TRAINING! OR INTERVENTION/TI,AB OR QUALITY ASSURANCE, HEALTHCARE! OR TRAINING/TI
******

S55 118262 S15 OR S20 OR S21 OR S22 OR S23
S56 584189 S30 OR S29 OR S28 OR S27
S57 970648 S37 OR S25 OR S24 OR S26 OR S24
S58 498295 S17 OR S16 OR S33 OR S34 OR S31 OR S35 OR S36 OR S37
S59 18937 S58 AND S57 AND S56 AND S55
S60 474 S58/TI AND S57/TI AND S56/TI AND S55/TI

EconLit

Set Items Description
S1 2855 AFGHANISTAN OR BANGLADESH OR BENIN OR BHUTAN OR BURKINA()F- ASO OR BURUNDI OR CAMBODIA OR CAMEROON
S2 21013 CENTRAL()AFRICAN()REPUBLIC OR CHAD OR COMOROS OR CONGO OR - COTE()D'IVOIRE OR ERITREA OR COTE()D()IVOIRE OR ETHIOPIA OR G- AMBIA OR GHANA OR GUINEA OR HAITI OR INDIA OR KENYA OR KOREA - OR KYRGYSZSTAN OR LAO OR LAOS OR LESOTHO OR LIBERIA
S3 7500 MADAGASCAR OR MALAWI OR MALI OR MAURITANIA OR MOLDOVA OR M- ONGOLIA OR MOZAMBIQUE OR MYANMAR OR NEPAL OR NICARAGUA OR NIG- ER OR NIGERIA OR PAKISTAN OR RWANDA OR SAO()TOME OR SENEGAL OR SIERRA()LEONE OR SOLOMON()ISLANDS OR SOMALIA OR SUDAN

S4 10485 TAJIKISTAN OR TANZANIA OR TIMOR OR TOGO OR UGANDA OR UZBEK-
ISTAN OR VIETNAM OR YEMEN OR ZAIRE OR ZAMBIA OR ZIMBABWE OR A-
LBANIA OR ALGERIA OR ANGOLA OR ARMENIA OR AZERBAIJAN OR BELA-
RUS OR BOLIVIA OR BOSNIA OR HERZEGOVINA OR BRAZIL

S5 23931 BULGARIA OR CAPE()VERDE OR CHINA OR COLOMBIA OR CUBA OR DJ-
IBOUTI OR DOMINICAN()REPUBLIC OR ECUADOR OR EGYPT OR EL()SALV-
ADOR OR FIJI OR GEORGIA OR GUATEMALA OR GUYANA OR HONDURAS OR
INDONESIA OR IRAN OR IRAQ OR JAMAICA OR JORDAN

S6 6424 KAZAKHSTAN OR KIRIBATI OR MACEDONIA OR MALDIVES OR MARSHAL-
L()ISLANDS OR MICRONESIA OR MOROCCO OR NAMIBIA OR PARAGUAY OR
PERU OR PHILIPPINES OR ROMANIA OR SAMOA OR SERBIA OR MONTENEG-
RO OR SRI()LANKA OR SURINAME OR SWAZILAND OR SYRIA

S7 3738 THAILAND OR TONGA OR TUNISIA OR TURKMENISTAN OR UKRAINE OR
VANUATU OR WEST()BANK OR GAZA

S8 13543 ANTIGUA OR BARBUDA OR ARGENTINA OR BARBADOS OR BELIZE OR B-
OTSWANA OR CHILE OR COSTA()RICA OR CROATIA OR CZECH()REPUBLIC
OR DOMINICA OR EQUATORIAL()GUINEA OR ESTONIA OR GABON OR GREN-
ADA OR HUNGARY OR LATVIA OR LEBANON OR LIBYA OR LITHUANIA

S9 16129 MALAYSIA OR MAURITIUS OR MAYOTTE OR MEXICO OR MARIANA()ISL-
ANDS OR OMAN OR PALAU OR PANAMA OR POLAND OR RUSSIAN()FEDERAT-
ION OR SEYCHELLES OR SLOVAK()REPUBLIC OR SOUTH()AFRICA OR ST(-
)KITTS OR ST(1W)KITTS OR NEVIS OR ST()LUCIA OR ST(1W)LUCIA

S10 4776 ST()VINCENT OR GRENADINES OR TRINIDAD OR TOBAGO OR TURKEY -
OR URUGUAY OR VENEZUELA

S11 41705 ASIA OR WEST()INDIES OR POLYNESIA OR MICRONESIA OR MIDDLE -
EAST OR AFRICA OR LATIN AMERICA OR CENTRAL AMERICA OR SOUTH A-
MERICA OR CARIBBEAN OR WEST INDIES REGION OR HISPA?OLA OR SOU-
THEAST ASIA OR SUB()SAHARAN()AFRICA OR EASTERN()EUROPE

S12 1 HISPANIOLA

S13 6628 BURMA OR EAST()TIMOR OR CONGO OR GUIANA OR CZECHOSLOVAKIA -
OR LAOS OR NORTH()KOREA OR IVORY()COAST OR REPUBLIC(1W)GEORG-
IA OR YEMEN OR ZAIRE OR SLOVAKIA OR SOVIET()UNION OR SURINAM -
OR USSR OR SAMOA OR YUGOSLAVIA

S14 22992 DEVELOPING()(NATION? ? OR COUNTRIES) OR LESS()DEVELOPED()(-
NATION? ? OR COUNTRIES) OR THIRD()WORLD()(NATION? ? OR COUNTR-
IES) OR UNDER()DEVELOPED()(NATION? ? OR COUNTRIES) OR POOR()-
(NATION? ? OR COUNTRIES OR ECONOMIES)

S15 132743 S1 OR S2 OR S3 OR S4 OR S5 OR S6 OR S7 OR S8 OR S9 OR S10 -
OR S11 OR S12 OR S13 OR S14

S16 55530 DOCTOR? ? OR PERSONNEL OR WORKER? ? OR STUDENT? ? OR PHYSI-
CIAN? ? OR PROVIDER? ? OR PARAMEDICAL OR MIDWIFE OR MIDWIVES -
OR PROFESSIONAL? ? OR NURSE? ? OR PRACTITIONER? ? OR THERAPIS-
T? ? OR ATTENDANT? ? OR HOSPITAL? ? OR POST? ?

S17 71658 CENTER? ? OR FACILITIES OR FACILITY OR SERVICE? ? OR COMMU-
NITY

S18 128687 EDUCATION OR INSERVICE OR TRAINING OR AUDIT OR PRACTICE OR
INTERVENTION? ? OR ASSURANCE OR KNOWLEDGE OR ATTITUDE? ?OR CO-

VERAGE OR OCCUPATION? ? OR OCCUPATIONAL OR PLANNING OR VOCAT-
ION OR VOCATIONAL OR CAREER OR EMPLOYEE

S19 127511 JOB()SATISFACTION OR MENTORING OR WORK OR WORKING OR WAGE -
OR WAGES OR BENEFIT? ? OR SAFETY OR POPULATION OR DEMOGRAPHY -
OR INCENTIVES OR HUMAN()ADAPTIBILITY OR PREVENTION OR IMMUNIZ-
ATIONS OR IMMUNIZE OR VACCINATIONS OR VACCINATE

S20 415737 VACCINE? ? OR DISEASE OR TREATMENT OR THERAPY OR PREVENTIVE
OR SANITATION OR IMPROVEMENT OR ECONOMIC? ? OR INTEGRATION OR
INITIATIVES OR STRATEGIC()PLANNING OR LEADERSHIP OR SECTOR()-
WIDE OR DECENTRALIZATION OR DECONCENTRATION

S21 126039 AUTONOMIZATION OR AUTHORITY OR FEE OR FEES OR PAYMENT OR R-
ISK OR INSURANCE OR FINANCING OR COPAYMENT OR CASH OR VOUCHERS
OR COST OR EFFICIENT OR EFFICIENCY OR SAVINGS()ACCOUNT? ? OR
MOBILIZATION OR EMPOWERMENT OR ENGAGEMENT

S22 158534 COALITION? ? OR ORGANIZATION? ? OR CONSUMER OR PATIENT OR -
REPORT()CARD? ? OR SCORECARDS OR PATIENT()SATISFACTION OR MAR-
KETING OR FRANCHISE OR DEMAND

S23 123731 CONTRACT OR CONTRACTING OR REGULATION OR ACCREDITATION OR -
LICENSING OR SERVICE()AGREEMENT ? ? OR PARTNERSHIP? ? OR STEW-
ARDSHIP OR PERFORMANCE OR QUALITY OR BENCHMARK OR BENCHMARKING
OR REORGANIZATION OR REDESIGN OR RE()ENGINEERING OR TEAM

S24 59087 TEAMS OR GUIDELINE? ? OR SUPPLY()CHAIN OR CERTIFICATE(1W)N-
EED OR ASSESSMENT OR MOTIVATION OR COMPETENCE OR STAFF OR STA-
FFING OR SKILL OR PRODUCTIVITY

S25 55530 DOCTOR? ? OR PERSONNEL OR WORKER? ? OR STUDENT? ? OR PHYSI-
CIAN? ? OR PROVIDER? ? OR PARAMEDICAL OR MIDWIFE OR MIDWIVES -
OR PROFESSIONAL? ? OR NURSE? ? OR PRACTITIONER? ? OR THERAPIS-
T? ? OR ATTENDANT? ? OR HOSPITAL? ? OR POST? ?

S26 242893 STUDY OR STUDIES

S27 71658 CENTER? ? OR FACILITIES OR FACILITY OR SERVICE? ? OR COMMU-
NITY

S28 2825 CHILDREN(2W) HOSPITAL? ?/TI OR COMMUNITY()HEALTH()SERVICES-
/DE OR DOCTOR/TI,AB OR DOCTORS/TI,AB OR HEALTH PERSONNEL! OR
HEALTH POSTS/TI,AB OR HEALTH()SERVICES()RESEARCH/DE OR HEALTH-
()WORKERS/TI,AB OR HOSPITAL/TI,AB OR HOSPITALS/TI,AB

S29 2 HEALTH()POSTS/TI,AB

S30 1980 MEDICAL()STUDENTS/TI OR OBSTETRIC()CARE/TI OR PHARMACEUTIC-
AL()SERVICES/DE OR PHARMACY()SERVICE()HOSPITAL/DE OR PHYSICIA-
NS/TI,AB OR PRIMARY()HEALTH()CARE/TI,AB OR PRIMARY()HEALTH()C-
ENTERS/TI,AB OR PROVIDERS/TI,AB

S31 19801 AUDIT/TI OR EDUCATION, CONTINUING! OR HEALTH CARE COSTS! OR
HEALTH EDUCATION! OR HEALTH KNOWLEDGE, ATTITUDES, PRACTICE! -
OR IMPACT/TI OR INSERVICE, TRAINING! OR INTERVENTION/TI,AB OR
QUALITY ASSURANCE, HEALTHCARE! OR TRAINING/TI

S32 0 QUALITY ASSURANCE, HEALTH CARE!

S33 0 HEALTH KNOWLEDGE, ATTITUDES, PRACTICE!

S34 0 EPIDEMIOLOGIC METHODS!

S35 0 S59 AND (HEALTH? OR DOCTOR? OR PHYSICIAN? ? OR PHARMAC? OR MIDWIF? OR MIDWIVES OR DENTIST? ? OR NURSE OR NURSES OR CLIN-IC? ? OR HOSPITAL? ?)
S36 0 S58 OR S37
S37 0 S63 AND S57 AND S56 AND S55
S38 0 S63/TI AND S57/TI AND S56/TI AND S55/TI
S39 0 S64 AND (HEALTH? OR DOCTOR? OR PHYSICIAN? ? OR PHARMAC? OR MIDWIF? OR MIDWIVES OR DENTIST? ? OR NURSE OR NURSES OR CLI-NIC? ? OR HOSPITAL? ?)
S40 0 S65 AND (HEALTH? OR DOCTOR? OR PHYSICIAN? ? OR PHARMAC? OR MIDWIF? OR MIDWIVES OR DENTIST? ? OR NURSE OR NURSES OR CLIN-IC? ? OR HOSPITAL? ?)
S41 906 15 AND (S16 OR S17) AND (S18 OR S19 OR S20 OR S21 OR S22 OR S23 OR S24 OR S27 OR S28 OR S25 OR S29 OR S30 OR S31)
S42 905 S41/1980:2006
S43 204 S42 AND (HEALTH? OR DOCTOR? OR PHYSICIAN? ? OR PHARMAC? OR MIDWIF? OR MIDWIVES OR DENTIST? ? OR NURSE OR NURSES OR CLI-NIC? ? OR HOSPITAL? ?)

Eldis

ELDIS search strategy:
"(physician* or nurse* or doctor* or midwi* or dentist* or paramedic* or worker* or volunteer*) and (train* or education) AND Health policy AND Africa"
Your search found 127 out of 30736 records

EMBASE

(EDUCATION OR INSERVICE OR TRAINING OR AUDIT OR PRACTICE OR INTERVENTION OR ASSURANCE OR KNOWLEDGE OR ATTITUDE OR COVERAGE OR OCCUPATION OR OCCUPATIONAL OR PLANNING OR VOCATION OR VOCATIONAL OR CAREER OR EMPLOYEE or employees or 'jOB SATISFACTION' OR MENTORING OR WORK OR WORKING OR WAGE or WAGES OR BENEFIT OR SAFETY OR POPULATION OR dEMOGRAPHY OR INCENTIVES OR 'HUMAN ADAPTIBILITY' OR PREVENTION OR IMMUNIZATIONS OR IMMUNIZE OR VACCINATIONS OR VACCINATE OR VACCINE OR DISEASE OR TREATMENT OR THERAPY OR PREVENTIVE OR SANITATION OR IMPROVEMENT OR ECONOMIC OR INTEGRATION OR iNITIATIVES OR 'sTRATEGIC PLANNING' OR LEADERSHIP OR 'SECTOR-WIDE' OR dECENTRALIZATION OR DECONCENTRATION or AUTONOMIZATION OR AUTHORITY OR FEE OR FEES OR PAYMENT OR RISK OR INSURANCE OR FINANCING OR COPAYMENT OR CASH OR VOUCHERS OR COST or costs OR EFFICIENT OR EFFICIENCY OR 'SAVINGS ACCOUNT' OR MOBILIZATION OR EMPOWERMENT OR ENGAGEMENT or COALITION OR ORGANIZATION OR CONSUMER or consumers or PATIENT OR 'REPORT CARD' OR SCORECARDS OR 'pATIENT SATISFACTION' OR MARKETING OR FRANCHISING OR DEMAND or CONTRACT OR CONTRACTING OR REGULATION OR ACCREDITATION OR lICENSING OR 'SERVICE AGREEMENT' OR PARTNERSHIP or partnerships OR STEWARDSHIP OR PERFORMANCE OR QUALITY OR BENCHMARK OR BENCHMARKING or REORGANIZATION OR REDESIGN OR 'RE-ENGINEERING' OR TEAM or TEAMS OR GUIDELINE OR 'SUPPLY CHAIN' OR 'CERTIFICATE of NEED'

OR ASSESSMENT OR MOTIVATION OR COMPETENCE OR STAFF OR STAFFING OR SKILL OR PRODUCTIVITY) and (AFGHANISTAN OR BANGLADESH OR BENIN OR BHUTAN OR 'BURKINA FASO' OR BURUNDI OR CAMBODIA OR CAMEROON or 'cENTRAL AFRICAN REPUBLIC' OR CHAD OR COMOROS OR CONGO OR 'COTE D IVOIRE' OR ERITREA OR ETHIOPIA OR GAMBIA OR GHANA OR GUINEA OR HAITI OR INDIA OR KENYA OR KOREA OR KYRGYSZSTAN OR LAO OR LAOS OR LESOTHO OR LIBERIA or MADAGASCAR OR MALAWI OR MALI OR MAURITANIA OR MOLDOVA OR MONGOLIA OR MOZAMBIQUE OR MYANMAR OR NEPAL OR NICARAGUA OR NIGER OR NIGERIA OR PAKISTAN OR RWANDA OR 'SAO TOME' OR SENEGAL OR 'SIERRA LEONE' OR 'sOLOMON ISLANDS' OR SOMALIA OR SUDAN or TAJIKISTAN OR TANZANIA OR TIMOR OR TOGO OR UGANDA OR UZBEKISTAN OR VIETNAM OR YEMEN OR ZAIRE OR ZAMBIA OR ZIMBABWE OR ALBANIA OR ALGERIA OR ANGOLA OR ARMENIA OR AZERBAIJAN OR BELARUS OR BOLIVIA OR BOSNIA OR HERZEGOVINA OR BRAZIL or BULGARIA OR 'CAPE VERDE' OR CHINA OR COLOMBIA OR CUBA OR DJIBOUTI OR 'DOMINICAN REPUBLIC' OR ECUADOR OR EGYPT OR 'EL SALVADOR' OR FIJI OR GEORGIA OR GUATEMALA OR GUYANA OR HONDURAS OR INDONESIA OR IRAN OR IRAQ OR JAMAICA OR JORDAN or KAZAKHSTAN OR KIRIBATI OR MACEDONIA OR MALDIVES OR 'MARSHALL ISLANDS' OR MICRONESIA OR MOROCCO OR NAMIBIA OR PARAGUAY OR PERU OR PHILIPPINES OR ROMANIA OR SAMOA OR SERBIA OR MONTENEGRO OR 'SRI LANKA' OR SURINAME OR SWAZILAND OR SYRIA or THAILAND OR TONGA OR TUNISIA OR TURKMENISTAN OR UKRAINE OR VANUATU OR 'WEST BANK' OR GAZA or ANTIGUA OR BARBUDA OR ARGENTINA OR BARBADOS OR BELIZE OR BOTSWANA OR CHILE OR 'COSTA RICA' OR CROATIA OR 'CZECH REPUBLIC' OR DOMINICA OR 'EQUATORIAL GUINEA' OR ESTONIA OR GABON OR GRENADA OR HUNGARY OR LATVIA OR LEBANON OR LIBYA OR LITHUANIA or MALAYSIA OR MAURITIUS OR MAYOTTE OR MEXICO OR 'MARIANA ISLANDS' OR OMAN OR PALAU OR PANAMA OR POLAND OR 'RUSSIAN FEDERATion' or SEYCHELLES OR 'SLOVAK REPUBLIC' OR 'SOUTH AFRICA' OR 'ST. KITTS' OR NEVIS OR 'ST. LUCIA' OR 'ST. VINCENT' OR GRENADINES OR TRINIDAD OR TOBAGO OR TURKEY OR URUGUAY OR VENEZUELA or ASIA OR 'WEST INDIES' OR POLYNESIA OR MICRONESIA OR 'MIDDLE eAST' OR AFRICA OR 'LATIN AMERICA' OR 'CENTRAL AMERICA' OR 'SOUTH AMERICA' OR CARIBBEAN OR hispaniola OR 'SOUTHEAST ASIA' OR 'SUB SAHARAN AFRICA' OR 'EASTERN EUROPE' or BURMA OR 'eAST TIMOR' OR CONGO OR GUIANA OR CZECHOSLOVAKIA OR LAOS OR 'NORTH KOREA' OR 'IVORY COAST' OR 'REPUBLIC of GEORGIA' OR YEMEN OR ZAIRE OR SLOVAKIA OR 'SOVIET UNION' OR SURINAM OR USSR OR SAMOA OR YUGOSLAVIA or 'DEVELOPING nATIONs' OR 'developing COUNTRIES' OR 'LESS DEVELOPEd NATIONs' or 'less developed COUNTRIES' OR 'THIRD WORLD NATIONs' OR 'third world COUNTRIES' OR 'UNDER DEVELOPED NATIONs' or 'underdeveloped countries' OR 'POOR NATIONs' OR 'poor COUNTRIES' OR 'poor ECONOMIES') and (DOCTOR or doctors OR PERSONNEL OR WORKER or workers OR STUDENT or students OR PHYSICIAN OR PROVIDER or providers OR PARAMEDICAL OR MIDWIFE OR MIDWIVES OR PROFESSIONAL or professionals OR NURSE or nurses OR PRACTITIONER OR THERAPIST OR ATTENDANT or attendants OR HOSPITAL or hospitals OR POST or posts or CENTER or centers OR FACILITIES OR FACILITY OR SERVICE OR COMMUnITY)

<u>ERIC</u>

Set Items Description
S1 1180 AFGHANISTAN OR BANGLADESH OR BENIN OR BHUTAN OR BURKINA()F-
ASO OR BURUNDI OR CAMBODIA OR CAMEROON
S2 8262 CENTRAL()AFRICAN()REPUBLIC OR CHAD OR COMOROS OR CONGO OR -

COTE()D'IVOIRE OR ERITREA OR COTE()D()IVOIRE OR ETHIOPIA OR G-
AMBIA OR GHANA OR GUINEA OR HAITI OR INDIA OR KENYA OR KOREA -
OR KYRGYSZSTAN OR LAO OR LAOS OR LESOTHO OR LIBERIA

S3 4411 MADAGASCAR OR MALAWI OR MALI OR MAURITANIA OR MOLDOVA OR M-
ONGOLIA OR MOZAMBIQUE OR MYANMAR OR NEPAL OR NICARAGUA OR NIG-
ER OR NIGERIA OR PAKISTAN OR RWANDA OR SAO()TOME OR SENEGAL OR
SIERRA()LEONE OR SOLOMON()ISLANDS OR SOMALIA OR SUDAN

S4 5211 TAJIKISTAN OR TANZANIA OR TIMOR OR TOGO OR UGANDA OR UZBEK-
ISTAN OR VIETNAM OR YEMEN OR ZAIRE OR ZAMBIA OR ZIMBABWE OR A-
LBANIA OR ALGERIA OR ANGOLA OR ARMENIA OR AZERBAIJAN OR BELA-
RUS OR BOLIVIA OR BOSNIA OR HERZEGOVINA OR BRAZIL

S5 17169 BULGARIA OR CAPE()VERDE OR CHINA OR COLOMBIA OR CUBA OR DJ-
IBOUTI OR DOMINICAN()REPUBLIC OR ECUADOR OR EGYPT OR EL()SALV-
ADOR OR FIJI OR GEORGIA OR GUATEMALA OR GUYANA OR HONDURAS OR
INDONESIA OR IRAN OR IRAQ OR JAMAICA OR JORDAN

S6 3941 KAZAKHSTAN OR KIRIBATI OR MACEDONIA OR MALDIVES OR MARSHAL-
L()ISLANDS OR MICRONESIA OR MOROCCO OR NAMIBIA OR PARAGUAY OR
PERU OR PHILIPPINES OR ROMANIA OR SAMOA OR SERBIA OR MONTENEG-
RO OR SRI()LANKA OR SURINAME OR SWAZILAND OR SYRIA

S7 1971 THAILAND OR TONGA OR TUNISIA OR TURKMENISTAN OR UKRAINE OR
VANUATU OR WEST()BANK OR GAZA

S8 3843 ANTIGUA OR BARBUDA OR ARGENTINA OR BARBADOS OR BELIZE OR B-
OTSWANA OR CHILE OR COSTA()RICA OR CROATIA OR CZECH()REPUBLIC
OR DOMINICA OR EQUATORIAL()GUINEA OR ESTONIA OR GABON OR GREN-
ADA OR HUNGARY OR LATVIA OR LEBANON OR LIBYA OR LITHUANIA

S9 11663 MALAYSIA OR MAURITIUS OR MAYOTTE OR MEXICO OR MARIANA()ISL-
ANDS OR OMAN OR PALAU OR PANAMA OR POLAND OR RUSSIAN()FEDERAT-
ION OR SEYCHELLES OR SLOVAK()REPUBLIC OR SOUTH()AFRICA OR ST(-
)KITTS OR ST(1W)KITTS OR NEVIS OR ST()LUCIA OR ST(1W)LUCIA

S10 1602 ST()VINCENT OR GRENADINES OR TRINIDAD OR TOBAGO OR TURKEY -
OR URUGUAY OR VENEZUELA

S11 12241 ASIA OR WEST()INDIES OR POLYNESIA OR MICRONESIA OR MIDDLE -
EAST OR AFRICA OR LATIN AMERICA OR CENTRAL AMERICA OR SOUTH A-
MERICA OR CARIBBEAN OR WEST INDIES REGION OR HISPA?OLA OR SOU-
THEAST ASIA OR SUB()SAHARAN()AFRICA OR EASTERN()EUROPE

S12 2 HISPANIOLA

S13 5165 BURMA OR EAST()TIMOR OR CONGO OR GUIANA OR CZECHOSLOVAKIA -
OR LAOS OR NORTH()KOREA OR IVORY()COAST OR REPUBLIC(1W)GEORG-
IA OR YEMEN OR ZAIRE OR SLOVAKIA OR SOVIET()UNION OR SURINAM -
OR USSR OR SAMOA OR YUGOSLAVIA

S14 13162 DEVELOPING()(NATION? ? OR COUNTRIES) OR LESS()DEVELOPED()(-
NATION? ? OR COUNTRIES) OR THIRD()WORLD()(NATION? ? OR COUNTR-
IES) OR UNDER()DEVELOPED()(NATION? ? OR COUNTRIES) OR POOR()-
(NATION? ? OR COUNTRIES OR ECONOMIES)

S15 60499 S1 OR S2 OR S3 OR S4 OR S5 OR S6 OR S7 OR S8 OR S9 OR S10 -
OR S11 OR S12 OR S13 OR S14

S16 615656 DOCTOR? ? OR PERSONNEL OR WORKER? ? OR STUDENT? ? OR PHYSI-

CIAN? ? OR PROVIDER? ? OR PARAMEDICAL OR MIDWIFE OR MIDWIVES - OR PROFESSIONAL? ? OR NURSE? ? OR PRACTITIONER? ? OR THERAPIS- T? ? OR ATTENDANT? ? OR HOSPITAL? ? OR POST? ?

S17 293304 CENTER? ? OR FACILITIES OR FACILITY OR SERVICE? ? OR COMMU- NITY

S18 917637 EDUCATION OR INSERVICE OR TRAINING OR AUDIT OR PRACTICE OR INTERVENTION? ? OR ASSURANCE OR KNOWLEDGE OR ATTITUDE? ?OR CO- VERAGE OR OCCUPATION? ? OR OCCUPATIONAL OR PLANNING OR VOCAT- ION OR VOCATIONAL OR CAREER OR EMPLOYEE

S19 251013 JOB()SATISFACTION OR MENTORING OR WORK OR WORKING OR WAGE - OR WAGES OR BENEFIT? ? OR SAFETY OR POPULATION OR DEMOGRAPHY - OR INCENTIVES OR HUMAN()ADAPTIBILITY OR PREVENTION OR IMMUNIZ- ATIONS OR IMMUNIZE OR VACCINATIONS OR VACCINATE

S20 223412 VACCINE? ? OR DISEASE OR TREATMENT OR THERAPY OR PREVENTIVE OR SANITATION OR IMPROVEMENT OR ECONOMIC? ? OR INTEGRATION OR INITIATIVES OR STRATEGIC()PLANNING OR LEADERSHIP OR SECTOR()- WIDE OR DECENTRALIZATION OR DECONCENTRATION

S21 98031 AUTONOMIZATION OR AUTHORITY OR FEE OR FEES OR PAYMENT OR R- ISK OR INSURANCE OR FINANCING OR COPAYMENT OR CASH OR VOUCHERS OR COST OR EFFICIENT OR EFFICIENCY OR SAVINGS()ACCOUNT? ? OR MOBILIZATION OR EMPOWERMENT OR ENGAGEMENT

S22 116862 COALITION? ? OR ORGANIZATION? ? OR CONSUMER OR PATIENT OR - REPORT()CARD? ? OR SCORECARDS OR PATIENT()SATISFACTION OR MAR- KETING OR FRANCHISE OR DEMAND

S23 189900 CONTRACT OR CONTRACTING OR REGULATION OR ACCREDITATION OR - LICENSING OR SERVICE()AGREEMENT ? ? OR PARTNERSHIP? ? OR STEW- ARDSHIP OR PERFORMANCE OR QUALITY OR BENCHMARK OR BENCHMARKING OR REORGANIZATION OR REDESIGN OR RE()ENGINEERING OR TEAM

S24 263302 TEAMS OR GUIDELINE? ? OR SUPPLY()CHAIN OR CERTIFICATE(1W)N- EED OR ASSESSMENT OR MOTIVATION OR COMPETENCE OR STAFF OR STA- FFING OR SKILL OR PRODUCTIVITY

S25 615656 DOCTOR? ? OR PERSONNEL OR WORKER? ? OR STUDENT? ? OR PHYSI- CIAN? ? OR PROVIDER? ? OR PARAMEDICAL OR MIDWIFE OR MIDWIVES - OR PROFESSIONAL? ? OR NURSE? ? OR PRACTITIONER? ? OR THERAPIS- T? ? OR ATTENDANT? ? OR HOSPITAL? ? OR POST? ?

S26 365428 STUDY OR STUDIES

S27 293304 CENTER? ? OR FACILITIES OR FACILITY OR SERVICE? ? OR COMMU- NITY

S28 15944 CHILDREN(2W) HOSPITAL? ?/TI OR COMMUNITY()HEALTH()SERVICES- /DE OR DOCTOR/TI,AB OR DOCTORS/TI,AB OR HEALTH PERSONNEL! OR HEALTH POSTS/TI,AB OR HEALTH()SERVICES()RESEARCH/DE OR HEALTH- ()WORKERS/TI,AB OR HOSPITAL/TI,AB OR HOSPITALS/TI,AB

S29 1 HEALTH()POSTS/TI,AB

S30 9913 MEDICAL()STUDENTS/TI OR OBSTETRIC()CARE/TI OR PHARMACEUTIC- AL()SERVICES/DE OR PHARMACY()SERVICE()HOSPITAL/DE OR PHYSICIA- NS/TI,AB OR PRIMARY()HEALTH()CARE/TI,AB OR PRIMARY()HEALTH()C- ENTERS/TI,AB OR PROVIDERS/TI,AB

S31	68368	AUDIT/TI OR EDUCATION, CONTINUING! OR HEALTH CARE COSTS! OR HEALTH EDUCATION! OR HEALTH KNOWLEDGE, ATTITUDES, PRACTICE! - OR IMPACT/TI OR INSERVICE, TRAINING! OR INTERVENTION/TI,AB OR QUALITY ASSURANCE, HEALTHCARE! OR TRAINING/TI
S32	0	QUALITY ASSURANCE, HEALTH CARE!
S33	0	HEALTH KNOWLEDGE, ATTITUDES, PRACTICE!
S34	0	EPIDEMIOLOGIC METHODS!
S35	0	S59 AND (HEALTH? OR DOCTOR? OR PHYSICIAN? ? OR PHARMAC? OR MIDWIF? OR MIDWIVES OR DENTIST? ? OR NURSE OR NURSES OR CLIN-IC? ? OR HOSPITAL? ?)
S36	0	S58 OR S37
S37	0	S63 AND S57 AND S56 AND S55
S38	0	S63/TI AND S57/TI AND S56/TI AND S55/TI
S39	0	S64 AND (HEALTH? OR DOCTOR? OR PHYSICIAN? ? OR PHARMAC? OR MIDWIF? OR MIDWIVES OR DENTIST? ? OR NURSE OR NURSES OR CLI-NIC? ? OR HOSPITAL? ?)
S40	0	S65 AND (HEALTH? OR DOCTOR? OR PHYSICIAN? ? OR PHARMAC? OR MIDWIF? OR MIDWIVES OR DENTIST? ? OR NURSE OR NURSES OR CLIN-IC? ? OR HOSPITAL? ?)
S41	57767	S15 AND (S16 OR S17 OR S18 OR S19 OR S20 OR S21 OR S22 OR - S23 OR S24 OR S25 OR S26 OR S27 OR S28 OR S29 OR S30 OR S31)
S42	4920	S41 AND (HEALTH? OR DOCTOR? OR PHYSICIAN? ? OR PHARMAC? OR MIDWIF? OR MIDWIVES OR DENTIST? ? OR NURSE OR NURSES OR CLIN-IC? ? OR HOSPITAL? ?)
S43	3403	S42/TI
S44	2401	S43/1980:2006
S45	89844	HEALTH? OR DOCTOR? OR PHYSICIAN? ? OR PHARMAC? OR MIDWIF? - OR MIDWIVES OR DENTIST? ? OR NURSE OR NURSES OR CLINIC? ? OR - HOSPTIAL? ?
S46	21589	S45/TI
S47	870	S15 AND S46
S48	633	S47/1980:2006
S49	621	S41 AND S48
S50	462	S41/TI AND S49
S62	289	
?		

HMIC

HMIC Health Management Information Consortium
September 2006

Change Database Help LOGOFF

Personal Account Name: **samrowe**

#	Search History	Results	Display
1	(afghanistan or bangladesh or benin or burkina faso or burundi or cambodia or central african republic or chad or comoros or congo or cote d'ivoire or eritrea or ethiopia or ivory coast or gambia or ghana or guinea or haiti or india or kenya or korea or kyrgyszstan or lao or laos or lesotho or liberia or madagascar or malawi or mali or mauritania or modova or mongoli or mozambique or mayanmar or nepal or nicaragua or niger or nigeria or pakistan or rwanda or sao tome or senegal or sierra leone or solomon islands or somalia or sudan or tajikistan or tanzania or timor or togo or uganda or uzbekistan or vietnam or yemen or zaire or zambia or zimbabwe or albania or algeria or angola or armenia or azerbaijan or belarus or bolivia or bosnia or herzegovina or brazil or bulgaria or cape verde or china or colombia or cuba or djibouti or dominican republic or ecuador or egypt or el salvador or fiji or georgia or guatemala or guyana or honduras or indonesia or iran or iraq or jamaica or jordan or kazakhstan or kiribati or macedonia or maldives or marshall islands or micronesia or morocco or namibia or paraguay or peru or philippines or romania or samoa or serbia or montenegro or sri lanka or suriname or swaziland or syria).mp. [mp=title, other title, abstract, heading words]	1565	DISPLAY
2	(doctor$1 or personnel or worker$1 or student$1 or physician$1 or provider$1 or paramedical or midwife or midwives or professional$1 or nurse$1 or practitioner$1 or therapist$1 or attendant$1 or hospital$1 or post$1).mp. [mp=title, other title, abstract, heading words]	98048	DISPLAY
3	1 and 2	530	DISPLAY
4	(center$1 or facilities or facility or service$1 or community).mp. [mp=title, other title, abstract, heading words]	111790	DISPLAY
5	(education or inservice or training or audit or practice or intervention$1 or assurance or knowledge or attitude$1 or coverage or	96729	DISPLAY

CONTRACT

	occupation$1 or occupational or planning or vocation or vocational or career or employee).mp. [mp=title, other title, abstract, heading words]		
6	(job satisfaction or mentoring or work or working or wage or wages or benefit$1 or safety or population or demography or incentives or human adaptibility or prevention or immunizations or immunize or vaccinations or vaccinate).mp. [mp=title, other title, abstract, heading words]	70702	DISPLAY
7	(vaccine$1 or disease or treatment or therapy or preventive or sanitation or improvement or economic$1 or integration or initiatives or strategic planning or leadership or sector wide or decentralization or deconcentration or autonomization or authority or fee or fees or payment or risk or insurance or financing or copayment or cash or vouchers or cost or efficient or efficiency or savings account$1 or mobilzation or empowerment or engagement).mp. [mp=title, other title, abstract, heading words]	86083	DISPLAY
8	(coalition$1 or organization$1 or consumer or patient or report card$1 or scorecards or patient satisfaction or marketing or franchise or demand).mp. [mp=title, other title, abstract, heading words]	45752	DISPLAY
9	(contract or contracting or regulation or accreditation or licensing or service agreement$1 or partnership$1 or stewardship or performance or quality or benchmark or benchmarking or regorganization or redesign or re-engineering or team or teams or guideline$1 or supply chain or certificate of need or assessment or motivation or competence or staff or staffing or skill or productivity).mp. [mp=title, other title, abstract, heading words]	86537	DISPLAY
10	4 or 5 or 6 or 7 or 8 or 9	226801	DISPLAY
11	3 and 10	481	DISPLAY
12	(thailand or tonga or tunisia or turkmenistan or ukraine or vanuatu or west bank or gaza or antigua or barbuda or argentina or barbados or belize or botswana or chile or costa rica or croatia or czech republic or dominica or equatorial guinea or estonia or gabon or grenada or hungary or latvia or lebanon or libya or lithuania or libya or malaysia or mauritius or mayotte or mexico).mp. [mp=title, other title,	655	DISPLAY

	abstract, heading words]		
13	(mariana islands or oman or palau or panama or poland or russian federation or seychelles or slovak republic or south africa or st kitts or nevis or st lucia or st vincent or grenadines or trinidad or tobago or turkey or uruguay or venezuela or asia or west indies or polynesia or micronesia or middle east or africa or latin america or central america or south america or caribbean).mp. [mp=title, other title, abstract, heading words]	1465	DISPLAY
14	(((((developing adj (nation$1 or country or countries)) or less developed) adj (country or counties)) or third world) adj (nation$1 or country or countries or economies)).mp. [mp=title, other title, abstract, heading words]	24	DISPLAY
15	(hispanola or southeast asia or saharan africa or eastern europe or hispaniola or burma or east timor or congo or guiana or czechoslovakia or laos or north korea or yemen or republic of georgia or zaire or slovakia or soviet union or surinam or ussr or yugoslavia).mp. [mp=title, other title, abstract, heading words]	690	DISPLAY
16	12 or 13 or 14 or 15	2389	DISPLAY
17	3 and 16	122	DISPLAY
18	11 or 17	493	DISPLAY
19	limit 18 to yr="2000 - 2006"	184	DISPLAY
20	from 19 keep 1-184	184	DISPLAY
21	limit 18 to yr="1980 - 1999"	307	DISPLAY
22	limit 18 to yr="1990 - 1999"	159	DISPLAY
23	from 22 keep 1-159	159	DISPLAY
24	limit 18 to yr="1950 - 1989"	150	DISPLAY
**25**	**from 24 keep 1-150**	**150**	**DISPLAY**

Combine Searches Delete Searches Save Search/Alert

Advanced Search Basic Search Find Citation More Fields Search Tools

Keyword

Enter **Keyword** or phrase (use "$" for truncation):

☑ Map Term to Subject Heading

MEDLINE (via PubMed) (In Dialog)

```
Set   Items   Description
S1  1543769   EDUCATION OR INSERVICE OR TRAINING OR AUDIT OR PRACTICE OR
              INTERVENTION? ? OR ASSURANCE OR KNOWLEDGE OR ATTITUDE? ? OR C-
              OVERAGE OR OCCUPATION? ? OR OCCUPATIONAL OR PLANNING OR VOCAT-
              ION OR VOCATIONAL OR CAREER OR EMPLOYEE
S2  1825485   JOB()SATISFACTION OR MENTORING OR WORK OR WORKING OR WAGE -
              OR WAGES OR BENEFIT? ? OR SAFETY OR POPULATION OR DEMOGRAPHY -
              OR INCENTIVES OR HUMAN()ADAPTIBILITY OR PREVENTION OR IMMUNIZ-
              ATIONS OR IMMUNIZE OR VACCINATIONS OR VACCINATE
S3  4957568   VACCINE? ? OR DISEASE OR TREATMENT OR THERAPY OR PREVENTIVE
               OR SANITATION OR IMPROVEMENT OR ECONOMIC? ? OR INTEGRATION OR
               INITIATIVES OR STRATEGIC()PLANNING OR LEADERSHIP OR SECTOR()-
              WIDE OR DECENTRALIZATION OR DECONCENTRATION
S4  1259660   AUTONOMIZATION OR AUTHORITY OR FEE OR FEES OR PAYMENT OR R-
              ISK OR INSURANCE OR FINANCING OR COPAYMENT OR CASH OR VOUCHERS
               OR COST OR EFFICIENT OR EFFICIENCY OR SAVINGS()ACCOUNT? ? OR
              MOBILIZATION OR EMPOWERMENT OR ENGAGEMENT
S5  1548180   COALITION? ? OR ORGANIZATION? ? OR CONSUMER OR PATIENT OR -
              REPORT()CARD? ? OR SCORECARDS OR PATIENT()SATISFACTION OR MAR-
              KETING OR FRANCHISING OR DEMAND
S6  7341918   S1 OR S2 OR S3 OR S4 OR S5
S7  1203296   CONTRACT OR CONTRACTING OR REGULATION OR ACCREDITATION OR -
              LICENSING OR SERVICE()AGREEMENT? ? OR PARTNERSHIP? ? OR STEWA-
              RDSHIP OR PERFORMANCE OR QUALITY OR BENCHMARK OR BENCHMARKING
              OR REORGANIZATION OR REDESIGN OR RE()ENGINEERING OR TEAM
S8   731744   TEAMS OR GUIDELINE? ? OR SUPPLY()CHAIN OR CERTIFICATE(1W)N-
              EED OR ASSESSMENT  OR MOTIVATION OR COMPETENCE OR STAFF OR ST-
              AFFING OR SKILL OR PRODUCTIVITY
S9  7986723   S6 OR S7 OR S8
S10   13335   AFGHANISTAN OR BANGLADESH OR BENIN OR BHUTAN OR BURKINA()F-
              ASO OR BURUNDI OR CAMBODIA OR CAMEROON
S11  233582   CENTRAL()AFRICAN()REPUBLIC OR CHAD OR COMOROS OR CONGO OR -
              COTE()D'IVOIRE OR ERITREA OR COTE()D()IVOIRE OR ETHIOPIA OR G-
              AMBIA OR GHANA OR GUINEA OR HAITI OR INDIA OR KENYA OR KOREA -
              OR KYRGYSZSTAN OR LAO OR LAOS OR LESOTHO OR LIBERIA
S12   49258   MADAGASCAR OR MALAWI OR MALI OR MAURITANIA OR MOLDOVA OR M-
              ONGOLIA OR MOZAMBIQUE OR MYANMAR OR NEPAL OR NICARAGUA OR NIG-
              ER OR NIGERIA OR PAKISTAN OR RWANDA OR SAO()TOME OR SENEGAL OR
               SIERRA()LEONE OR SOLOMON()ISLANDS OR SOMALIA OR SUDAN
S13   65091   TAJIKISTAN OR TANZANIA OR TIMOR OR TOGO OR UGANDA OR UZBEK-
              ISTAN OR VIETNAM OR YEMEN OR ZAIRE OR ZAMBIA OR ZIMBABWE OR A-
              LBANIA  OR ALGERIA OR ANGOLA OR ARMENIA OR AZERBAIJAN OR BELA-
              RUS OR BOLIVIA OR BOSNIA OR HERZEGOVINA OR BRAZIL
S14   93907   BULGARIA OR CAPE()VERDE OR CHINA OR COLOMBIA OR CUBA OR DJ-
              IBOUTI OR DOMINICAN()REPUBLIC OR ECUADOR OR EGYPT OR EL()SALV-
```

ADOR OR FIJI OR GEORGIA OR GUATEMALA OR GUYANA OR HONDURAS OR INDONESIA OR IRAN OR IRAQ OR JAMAICA OR JORDAN

S15 28785 KAZAKHSTAN OR KIRIBATI OR MACEDONIA OR MALDIVES OR MARSHAL-L()ISLANDS OR MICRONESIA OR MOROCCO OR NAMIBIA OR PARAGUAY OR PERU OR PHILIPPINES OR ROMANIA OR SAMOA OR SERBIA OR MONTENEG-RO OR SRI()LANKA OR SURINAME OR SWAZILAND OR SYRIA

S16 32076 THAILAND OR TONGA OR TUNISIA OR TURKMENISTAN OR UKRAINE OR VANUATU OR WEST()BANK OR GAZA

S17 46899 ANTIGUA OR BARBUDA OR ARGENTINA OR BARBADOS OR BELIZE OR B-OTSWANA OR CHILE OR COSTA()RICA OR CROATIA OR CZECH()REPUBLIC OR DOMINICA OR EQUATORIAL()GUINEA OR ESTONIA OR GABON OR GREN-ADA OR HUNGARY OR LATVIA OR LEBANON OR LIBYA OR LITHUANIA

S18 88268 MALAYSIA OR MAURITIUS OR MAYOTTE OR MEXICO OR MARIANA()ISL-ANDS OR OMAN OR PALAU OR PANAMA OR POLAND OR RUSSIAN()FEDERAT-ION OR SEYCHELLES OR SLOVAK()REPUBLIC OR SOUTH()AFRICA OR ST(-)KITTS OR ST(1W)KITTS OR NEVIS OR ST()LUCIA OR ST(1W)LUCIA

S19 23371 ST()VINCENT OR GRENADINES OR TRINIDAD OR TOBAGO OR TURKEY - OR URUGUAY OR VENEZUELA

S20 113013 ASIA OR WEST()INDIES OR POLYNESIA OR MICRONESIA OR MIDDLE - EAST OR AFRICA OR LATIN AMERICA OR CENTRAL AMERICA OR SOUTH A-MERICA OR CARIBBEAN OR WEST INDIES REGION OR HISPA?OLA OR SOU-THEAST ASIA OR SUB()SAHARAN()AFRICA OR EASTERN()EUROPE

S21 44 HISPANIOLA

S22 84172 BURMA OR EAST()TIMOR OR CONGO OR GUIANA OR CZECHOSLOVAKIA - OR LAOS OR NORTH()KOREA OR IVORY()COAST OR REPUBLIC(1W)GEORG-IA OR YEMEN OR ZAIRE OR SLOVAKIA OR SOVIET()UNION OR SURINAM - OR USSR OR SAMOA OR YUGOSLAVIA

S23 65946 DEVELOPING()(NATION? ? OR COUNTRIES) OR LESS()DEVELOPED()(-NATION? ? OR COUNTRIES) OR THIRD()WORLD()(NATION? ? OR COUNTR-IES) OR UNDER()DEVELOPED()(NATION? ? OR COUNTRIES) OR POOR()-(NATION? ? OR COUNTRIES OR ECONOMIES)

S24 761381 S10 OR S11 OR S12 OR S13 OR S14 OR S15 OR S16 OR S17 OR S18 OR S19 OR S20 OR S21 OR S22 OR S23

S25 1624852 DOCTOR? ? OR PERSONNEL OR WORKER? ? OR STUDENT? ? OR PHYSI-CIAN? ? OR PROVIDER? ? OR PARAMEDICAL OR MIDWIFE OR MIDWIVES - OR PROFESSIONAL? ? OR NURSE? ? OR PRACTITIONER? ? OR THERAPIS-T? ? OR ATTENDANT? ? OR HOSPITAL? ? OR POST? ?

S26 825622 CENTER? ? OR FACILITIES OR FACILITY OR SERVICE? ? OR COMMU-NITY

S27 2087266 S25 OR S26

S28 188544 S27 AND S24

S29 154907 S9 AND S28

S30 73085 S29 AND (STUDY OR STUDIES)

S31 69232 S30/AB

S32 5865106 S9/TI,AB

S33 63989 S32 AND S31

S34 44508 S33/ENG,HUMAN

S35 91295 S24/TI,AB AND S27/TI,AB
S36 30610 S35 AND S34
S37 30610 S24/TI,AB AND S36
S38 2640315 S6/TI OR S7/TI OR S8/TI
S39 10541 S38 AND S37
S40 604079 S26/TI OR S27/TI
S41 2816 S39 AND S40
S42 2640315 S1/TI OR S2/TI OR S3/TI OR S4/TI OR S5/TI OR S7/TI OR S8/TI
S43 15518 S42 AND S34
S44 7555 S10/AB
S45 6014 S10/TI
S46 136210 S11/TI,AB
S47 33979 S12/TI,AB
S48 41149 S13/TI,AB
S49 53060 S14/TI,AB
S50 53060 S14/TI,AB
S51 15223 S15/TI,AB
S52 10079 S10/TI,AB
S53 13621 S16/TI,AB
S54 25662 S17/TI,AB
S55 39308 S18/TI,AB
S56 16152 S19/TI,AB
S57 48666 S20/TI,AB
S58 44 S21/TI,AB
S59 23652 S22/TI,AB
S60 20797 S23/TI,AB
S61 771237 60 OR S59 OR S58 OR S57 OR S56 OR S55 OR S54 OR S53 OR S52 OR S51 OR S50 OR S48 OR S47 OR S46
S62 406325 S60 OR S59 OR S58 OR S57 OR S56 OR S55 OR S54 OR S53 OR S52 OR S51 OR S50 OR S48 OR S47 OR S46
S63 72102 S62 AND S42
S64 11352 S34 AND S63
S65 10541 S64 AND (S25/TI,AB OR S26/TI,AB)
S66 721008 CHILDREN(2W)HOSPITAL? ?/TI OR COMMUNITY()HEALTH()SERVICES/-DE OR DOCTOR/TI,AB OR DOCTORS/TI,AB OR HEALTH PERSONNEL! OR H-EALTH()POSTS/TI,AB OR HEALTH()SERVICES()RESEARCH/DE OR HEALTH-()WORKERS/TI,AB OR HOSPITAL/TI,AB OR HOSPITALS/TI,AB
S67 153675 MEDICAL()STUDENTS/TI OR OBSTETRIC()CARE/TI OR PHARMACEUTIC-AL()SERVICES/DE OR PHARMACY()SERVICE()HOSPITAL/DE OR PHYSICIA-NS/TI,AB OR PRIMARY()HEALTH()CARE/TI,AB OR PRIMARY()HEALTH()C-ENTRES/TI,AB OR PROVIDERS/TI,AB
S68 818451 S66 OR S67
S69 413517 AUDIT/TI OR EDUCATION, CONTINUING! OR HEALTH CARE COSTS! OR HEALTH EDUCATION! OR HEALTH KNOWLEDGE, ATTITUDES, PRACTICE! -OR IMPACT/TI OR INSERVICE TRAINING! OR INTERVENTION/TI,AB OR -QUALITY ASSURANCE, HEALTHCARE! OR TRAINING/TI
S71 149104 QUALITY ASSURANCE, HEALTH CARE!

S72	539997	S71 OR S69
S73	11369	S72 AND S68 AND S24
S74	8890	S65 NOT S73
S75	6723	S74 AND EPIDEMIOLOGIC METHODS!
S76	6723	S75/ENG, HUMAN, AB
S77	6716	S76/1970:2006
S78	6705	S76/1975:2006
S79	6657	S76/1980:2006

SCI Web Of Science, SSCI, and Global Health (In Dialog)

ss (Health(2N)services OR health(2N)workers OR health(2N)clinics OR health()care OR birth()attendants OR health(2N)providers OR health(2N)education)/ti AND

Ss (Asia OR Africa OR developing()countries OR Middle()East OR Latin()America OR south()America OR eastern()europe)/de,ti AND

(PERFORMANCE OR QUALITY OR BENCHMARK? OR Effective? Or efficien? OR appraisal?
OR Training OR education OR knowledge OR competen? OR evaluation OR cost()recovery)/de,ti,ab OR
Ss (analysis OR SKILL OR PRODUCTIVITY OR IMPACT OR INTERVENTION OR initiative?
OR rating OR REPORT()CARD? OR SCORECARD? OR PATIENT()SATISFACTION OR quality)/de,ti,ab

Restrict to 1984-2006

Sociological Abstracts

((kw=nurse or nurses or doctor or doctors or physician
or physicians or therapist or therapists or nurse or nurses or midwife or
midwives or dentist or dentists) and ((kw=afghanistan or bangladesh or
benin or burkina faso or burundi or cambodia or central african republic
or chad or comoros or congo or cote d'ivoire or eritrea or ethiopia or
ivory coast or gambia or ghana or guinea or haiti or india or kenya or
korea or kyrgyszstan or lao or laos or lesotho or liberia or madagascar
or malawi or mali or mauritania or modova or mongoli or mozambique or
mayanmar or nepal or nicaragua or niger or nigeria or pakistan or rwanda
or sao tome or senegal or sierra leone or solomon islands or somalia or
sudan or tajikistan or tanzania or timor or togo or uganda or uzbekistan
or vietnam or yemen or zaire or zambia or zimbabwe or albania or algeria
or angola or armenia or azerbaijan or belarus or bolivia or bosnia or
herzegovina or brazil or bulgaria or cape verde or china or colombia or
cuba or djibouti or dominican republic or ecuador or egypt or el salvador
or fiji or georgia or guatemala or guyana or honduras or indonesia or iran
or iraq or jamaica or jordan or kazakhstan or kiribati or macedonia or
maldives or marshall islands or micronesia or morocco or namibia or
paraguay or peru or philippines or romania or samoa or serbia or
montenegro or sri lanka or suriname or swaziland or syria) and (KW=doctor

or doctors or personnel or worker or workers or student or students or physician or physicians or provider or providers or paramedical or midwife or midwives or professional or nurse or practitioner or therapist or attendant or hospital or post or center or facilities or facility or service or community or education or inservice or training or audit or practice or intervention or assurance or knowledge or attitude or coverage or occupation or occupational or planning or vocation or vocational or career or employee or job satisfaction or mentoring or work or working or wage or wages or benefit or safety or population or demography or incentives or human adaptibility or prevention or immunizations or immunize or vaccinations or vaccinate or vaccine or disease or treatment or therapy or preventive or sanitation or improvement or economic or integration or initiatives or strategic planning or leadership or sector wide or decentralization or deconcentration or autonomization or authority or fee or fees or payment or risk or insurance or financing or copayment or cash or vouchers or cost or efficient or efficiency or savings account or mobilzation or empowerment or engagement or coalition or organization or consumer or patient or report card or scorecards or patient satisfaction or marketing or franchise or demand or contract or contracting or regulation or accreditation or licensing or service agreement or partnership or stewardship or performance or quality or benchmark or benchmarking or regorganization or redesign or re-engineering or team or teams or guideline or supply chain or certificate of need or assessment or motivation or competence or staff or staffing or skill or productivity)))
and (program or programs or intervention or interventions)

We also performed a special electronic search of MEDLINE (1950-2006) via Ovid to identify IMCI studies. The search strategy for this special search is below:

1 Integrated Management of Childhood Illness.af. (136)
2 IMCI.af. (233)
3 1 or 2 (271)
4 limit 3 to yr="1950 - 2006" (264)
5 remove duplicates from 4 (262)
6 from 5 keep 1-262 (262)

A.3. Non-electronic search strategy

We also conducted the following non-electronic searches to identify articles for this review:

1) We reviewed bibliographies of 510 previous systematic reviews and other related papers:

1. Abas M, Baingana F, Broadhead J, Iacoponi E, Vanderpyl Abas M, Baingana F, Broadhead J, Iacoponi E, Vanderpyl J. Common mental disorders and primary health care: Current practice in low-income countries. Harvard Review of Psychiatry, 2003;11(3):166-73.

2. Abbatt, F. Scaling up health and education workers: community health workers. Literature Review. DFID Health Systems Resource Centre. March 2005.
3. Adams O, Hicks V. Round Table Discussion: Pay and Non-Pay Incentives, Performance and Motivation. Prepared for WHO's December 2000 Global Health Workforce Strategy Group, Geneva. Human Resources Development e-Journal, 2000;4(3). Available at: http://www.who.int/hrh/en/HRDJ_4_3_02.pdf, accessed Dec 24, 2008
4. Aiga H, Banta JE. Emerging crisis of continuing professional education: becoming a dead letter between external pressure and personal incentive? Int J Health Plann Mgmt 2003; 18: 267–271.
5. Akinbami LJ, Cheng TL, Kornfeld D. A review of teen-tot programs: comprehensive clinical care for young parents and their children. Adolescence, 2001;36:381-393.
6. Alderman H, Lavy V. Household responses to public health services: cost and quality tradeoffs. The World Bank Research Observer, 1996;11(1):3-22
7. Aljunid S. The role of private medical practitioners and their interactions with public health services in Asian countries. Health Policy and Planning, 1995;10(4):333-349.
8. Allard P, Maunsell E, Labbé J, Dorval M. Educational interventions to improve cancer pain control: a systematic review. J Palliat Med. 2001;4(2):191-203.
9. Allen LH, Gillespie SR. What Works? A Review of the Efficacy and Effectiveness of Nutrition Interventions. United Nations Administrative Committee on Coordination Sub-Committee on Nutrition (ACC/SCN), Asian Development Bank (ADB). 2001.
10. Alliance for Health Policy and Systems Research. Strengthening health systems: the role and promise of policy and systems research. Global Forum for Health Research, Geneva: 2004. ISBN 2-940286-25-6. available at: http://www.who.int/alliance-hpsr/resources/Strengthening_complet.pdf, accessed May 20, 2010.
11. Amaral JJF, Victora CG. The effect of training in Integrated Management of Childhood Illness (IMCI) of the performance and healthcare quality of pediatric healthcare workers: a systematic review. Revista Brasileira de Saúde Materno Infantil, 2008;8(2):151–61.
12. Anderson LA, Sharpe PA. Improving patient and provider communication: a synthesis and review of communication interventions. Patient Education and Counseling, 1991;17:99-134.
13. Anonymous. Africa child survival initiative-Combatting childhood communicable diseases (ACSI-CCCD) Project Report. USAID, CDC. Atlanta, GA: 1994.
14. Anonymous. Community-based education of health personnel. Report of a WHO Study Group. World Health Organization Technical Report Series 746. Geneva: 1987. available at: http://whqlibdoc.who.int/trs/WHO_TRS_746.pdf, accessed April 2, 2010
15. Anonymous. Controlling malaria in Africa: progress and priorities. Africa child survival initiative-Combatting childhood communicable diseases (ACSI-CCCD) Project Report. USAID, CDC. Atlanta, GA: 1994.
16. Anonymous. MotherCare 1989-1993 : country project descriptions. John Snow, Inc., USAID. September 1993. USAID Award/Agreement Number: DPE-5966-Z-00-8083-00. available at: http://pdf.usaid.gov/pdf_docs/PNABR392.pdf, accessed May 20, 2010.
17. Anonymous. MotherCare Matters: Five Years of Learning and Action 1993-1998. A Compendium of Quarterly Newsletters and Literature Reviews on Maternal and Neonatal Health and Nutrition. Arlington, VA: John Snow, Inc., October 1998. available at: http://pdf.usaid.gov/pdf_docs/PNADN978.pdf, accessed January 21, 2010.

18. Anonymous. Strengthening the performance of community health workers in primary health care. Report of a WHO Study Group. WHO Technical Report Series 780. WHO: Geneva, 1989.
19. Anonymous. Strengthening the performance of community health workers in primary health care. Report of a WHO Study Group. World Health Organ Tech Rep Ser. 1989;780:1-46. available at: http://whqlibdoc.who.int/trs/WHO_TRS_780.pdf, accessed March 29, 2010
20. Anonymous. Training and utilization of auxiliary personnel for rural health teams in developing countries. Report of a WHO Expert Committee. World Health Organization Technical Report Series, Volume 633. World Health Organization: Geneva, 1979.
21. Apisarnthanarak A, Fraser VJ. Bloodborne pathogens and health care workers in developing countries: risk assessments and preventive strategies. Journal of Infectious Diseases and Antimicrobial Agents, 2002;19(2):65-79.
22. Arhin-Tenkorang, D. Mobilizing Resources for Health: The Case for User Fees Revisited. Draft C, 3 November 2000. Available at: http://www.cid.harvard.edu/cidwp/pdf/081.pdf, accessed Dec 9, 2008.
23. Arnold SR, Straus SE. Interventions to improve antibiotic prescribing practices in ambulatory care. Cochrane Database Syst Rev. 2005 Oct 19;(4):CD003539.
24. Ashworth NL, Chad KE, Harrison EL, Reeder BA, Marshall SC. Home versus center based physical activity programs in older adults. Cochrane Database Syst Rev. 2005;(1):CD004017.
25. Askew I, Berer M. The contribution of sexual and reproductive health services to the fight against HIV/AIDS: A review. Reprod Health Matters, 2003;11(22):51-73.
26. Awases G, Gbary A, Nyoni J, Chatora R. Migration of Health Professionals in Six Countries: A Synthesis Report. World Health Organization Regional Office for Africa: Brazzaville. 2004. 68 pages. Available at: http://www.hrhresourcecenter.org/node/61, accessed May 18, 2010.
27. Baguley D, Killeen T, Wright J. International health links: an evaluation of partnerships between health-care organizations in the UK and developing countries. Trop Doct. 2006;36(3):149-54.
28. Bamako Initiative Management Unit. Economic crisis, adjustment, and the Bamako Initiative: health care financing in the economic context of sub-saharan Africa. Background document for the Pan-African Conference on Community Financing in PHC, Kinshasa, Zaire, 25-27 June 1990. UNICEF, New York: June 1990.
29. Batt K, Fox-Rushby JA, Castillo-Riquelme M. The costs, effects and cost-effectiveness of strategies to increase coverage of routine immunizations in low- and middle-income countries: systematic review of the grey literature. Bull World Health Organ. 2004 Sep;82(9):689-96.
30. Bearden DT, Allen GP. Impact of antimicrobial control programs on patient outcomes. Pharmacy perspective. Dis Manage Health Outcomes, 2003;11(11):723-736.
31. Beekmann SE, Henderson DK. Protection of healthcare workers from bloodborne pathogens. Curr Opin Infect Dis. 2005;18(4):331-6.
32. Beith A, Eichler R, Weil D. Performance-Based Incentives for Health: A Way to Improve Tuberculosis Detection and Treatment Completion? Center for Global Development. CGD Working Paper #122 April 2007.

33. Beith A, Eichler R, Weil D. Worldwide: Incentives for Tuberculosis Diagnosis and Treatment. p. 237-256. In: Eichler R, Levine R, and the Performance-Based Incentives Working Group. Performance incentives for global health: potential and pitfalls. Versa Press: East Peoria, Illinois. 2009. available at: http://www.cgdev.org/doc/books/PBI/06_CGD_Eichler_Levine-Ch12.pdf; accessed July 7, 2009.
34. Benbassat J, Taragin M. What is adequate health care and how can quality of care be improved? Int J Health Care Qual Assur Inc Leadersh Health Serv. 1998;11(2-3):58-64.
35. Bennett JW, Glasziou PP. Computerised reminders and feedback in medication management: a systematic review of randomised controlled trials. Med J Aust. 2003;178(5):217-22.
36. Bennett S, Dakpallah G, Garner P, Gilson L, Nittayaramphong S, Zurita B, Zwi A. Carrot and stick: state mechanisms to influence private provider behavior. Health Policy Plan. 1994 Mar;9(1):1-13.
37. Bennett S, Franco LM. Public sector health worker motivation and health sector reform: a conceptual framework. January 1999. Major Applied Research 5, Technical Paper No. 1. Abt Associates, Inc: Bethesda, 1999.
38. Bennett S, Gzirishvili D. An In-depth Analysis of the Determinants and Consequences of Worker Motivation in Two Hospitals in Tbilisi, Georgia. December 2000. Major Applied Research 5. Working Paper 9. Abt Associates, Inc: Bethesda, 2000.
39. Bennett S, McPake B, Mills A. (editors) Private Health Providers in Developing Countries: Serving the Public Interest. 318 pages, St. Martin's Press, October 1997. ISBN-10: 1856494969, ISBN-13: 978-1856494960
40. Bennett S, Mills A. Government capacity to contract: health sector experience and lessons. Public Administration and Development, 1998;18(4):307-326.
41. Bennett S. Promoting the private sector: a review of developing country trends. Health Policy and Planning, 1992;7(2):97-110
42. Berer M. Integration of sexual and reproductive health services: a health sector priority. Reprod Health Matters. 2003;11(21):6-15.
43. Bero LA, Grilli R, Grimshaw JM, Harvey E, Oxman AD, Thomson MA. Closing the gap between research and practice: an overview of systematic reviews of interventions to promote the implementation of research findings. The Cochrane Effective Practice and Organization of Care Review Group. BMJ. 1998 Aug 15;317(7156):465-8.
44. Berwick DM. Lessons from developing nations on improving health care. BMJ. 2004 May 8;328(7448):1124-9. Erratum in: BMJ. 2004 May 29;328(7451):1301
45. Bhattacharyya K, Winch P, LeBan K, Tien M. Community health worker incentives and disincentives: how they affect motivation, retention, and sustainability. Published by the Basic Support for Institutionalizing Child Survival Project (BASICS II) for the United States Agency for International Development. Arlington, VA. October 2001.
46. Bhutta ZA, Ahmed T, Black RE, Cousens S, Dewey K, Giugliani E, Haider BA, Kirkwood B, Morris SS, Sachdev HP, Shekar M; Maternal and Child Undernutrition Study Group. What works? Interventions for maternal and child undernutrition and survival. Lancet. 2008 Feb 2;371(9610):417-40
47. Bhutta ZA, Darmstadt GL, Hasan BS, Haws RA. Community-based interventions for improving perinatal and neonatal health outcomes in developing countries: a review of the evidence. Pediatrics, 2005;115:519-617.

48. Bhutta ZA, Gupta I, de'Silva H, Manandhar D, Awasthi S, Hossain SM, Salam MA. Maternal and child health: is South Asia ready for change? BMJ. 2004 Apr 3;328(7443):816-9.
49. Bitran R, Yip WC. A review of health care provider payment reform in selected countries in Asia and Latin America. Major Applied Research 2, Working Paper 1. Bethesda, MD: Partnerships for Health Reform Project. Abt Associates, Inc. August 1998.
50. Bolam A, Tillen D, Costello A. Does health education improve newborn care? In: Costello A, Manandhar D. Improving newborn infant health in developing countries. Imperial College Press: London. 2000; 393-408
51. Borghi J, Ensor T, Somanathan A, Lissner C, Mills A; Lancet Maternal Survival Series steering group. Mobilising financial resources for maternal health. Lancet. 2006 Oct 21;368(9545):1457-65.
52. Bosch-Capblanch, Garner P. Primary health care supervision in developing countries. Tropical Medicine and International Health, 2008;13(3):369-383.
53. Bose S, Oliveras E, Edson WN. 2001. How can self-assessment improve the quality of healthcare? Operations Research Issue Paper 2(4). Published for the U.S. Agency for International Development (USAID) by the Quality Assurance (QA) Project, Bethesda, MD and JHPIEGO Corporation, Baltimore, MD.
54. Bossert T, Beauvais J, Bowser D. Decentralization of health systems: preliminary review of four country case studies. Major Applied Research 6, Technical Report No. 1. Bethesda, MD: Partnerships for Health Reform Project. Abt Associates, Inc. January 2000.
55. Bossert TJ, Beauvais JC. Decentralization of health systems in Ghana, Zambia, Uganda and the Philippines: a comparative analysis of decision space. Health Policy Plan. 2002 Mar;17(1):14-31.
56. Bower P, Garralda E, Kramer T, Harrington R, Sibbald B. The treatment of child and adolescent mental health problems in primary care: a systematic review. Fam Pract. 2001;18(4):373-82.
57. Bradley JE, Mayfield MV, Mehta MP, Rukonge A. Participatory evaluation of reproductive health care quality in developing countries. Soc Sci Med. 2002 Jul;55(2):269-82.
58. Bradley P, Lindsay B. Care delivery and self-management strategies for adults with epilepsy. CochraneDatabase of Systematic Reviews 2008, Issue 1. Art. No.: CD006244. DOI: 10.1002/14651858.CD006244.pub2. Update of: Bradley P, Lindsay B. Specialist epilepsy nurses for treating epilepsy. Cochrane Database Syst Rev. 2001;(4):CD001907
59. Brady BK, McGahan L, Skidmore B. Systematic review of economic evidence on stroke rehabilitation services. Int J Technol Assess Health Care. 2005;21(1):15-21.
60. Brieger W, Unwin A, Greer G, Meek S. Interventions to improve the role of medicine sellers in malaria case management for children in Africa. London, UK and Arlington, VA, USA: the Malaria Consortium and BASICS for the United States Agency for International Development. 2005. Prepared for The Sub-group for Communication and Training, The Malaria Case Management Working Group, Roll Back Malaria. Presented at Consultative Meeting On Interventions to Improve the Role of Medicine Sellers in Case Management of Malaria (Fever) in Children Under 5. Accra, Ghana, 26-27 May 2004.

61. Brieger WR, Adeniyi JD, Parker KA, Oladepo O. Health education in Africa: 1975-2000. Health Educ Res. 2000 Aug;15(4):383-91.
62. Briggs CJ, Garner P. Strategies for integrating primary health services in middle- and low-income countries at the point of delivery. Cochrane Database Syst Rev. 2006 Apr 19;(2):CD003318.
63. Brooker C, Repper JM, Booth A. The effectiveness of community mental health nursing: a review. British Journal of Clinical Governance, 1996; 1(2):44 - 50.
64. Broomberg J. Managing the health care market in developing countries: prospects and problems. Health Policy Plan. 1994 Sep;9(3):237-51.
65. Brown L, Breese K. Nepal Case Study Program: Evolution and Lessons Learned. Annotated Bibliography of Reports and Materials on Child Survival Programs. October 29, 2003. available at: http://www.phishare.org/files/1768_Nepal_Bibliography.pdf, accessed Jan 5, 2009
66. Brown L, Macintyre K, Trujillo L. Interventions to reduce HIV/AIDS stigma: what have we learned? AIDS Educ Prev. 2003;15(1):49-69.
67. Brugha R, Chandramohan D, Zwi A. Viewpoint: management of malaria--working with the private sector. Trop Med Int Health. 1999 May;4(5):402-6.
68. Brugha R, Zwi A. Improving the quality of private sector delivery of public health services: challenges and strategies. Health Policy Plan. 1998 Jun;13(2):107-20
69. Brugha R, Zwi AB. Sexually transmitted disease control in developing countries: the challenge of involving the private sector. Sex Transm Infect. 1999 Oct;75(5):283-5.
70. Bryce J, Victora CG, Habicht JP, Vaughan JP, Black RE. The multi-country evaluation of the integrated management of childhood illness strategy: lessons for the evaluation of public health interventions. Am J Public Health. 2004 Mar;94(3):406-15.
71. Buchan, J. Scaling up health and education workers: increasing the performance and productivity of an existing stock of health workers. Literature Review. DFID Health Systems Resource Centre. March 2005.
72. Buekens P, Keusch G, Belizan J, Bhutta ZA. Evidence-based global health. JAMA. 2004;291(21):2639-41.
73. Burkhalter B and Graham V, eds. Presented Papers: High Impact PVO Child Survival Programs, Volume 2. Proceedings of an Expert Consultation, Gaulladet University, Washington, DC, June 21-24, 1998. Published for the PVO community and the U.S. Agency for International Development by the Child Survival Collaboration and Resources (CORE) Group, Washington, DC, and Basic Support for Institutionalizing Child Survival Project (BASICS II), Arlington, VA, 1999.
74. Burkhalter BR, Bashir N, editors. Innovative approaches to child survival: summaries of evaluation studies by La Leche League, Project HOPE, Project Concern, and World Relief. Published for the US Agency for International Development by the Basic Support for Institutionalizing Child Survival (BASICS) Project, Arlington, VA. 1998.
75. Bustreo F, Harding A, Axelsson H. Can developing countries achieve adequate improvements in child health outcomes without engaging the private sector? Bulletin of the World Health Organization, 2003;81(12):886-95.
76. Calderon, TA. Nutrition education training of health workers and other field staff to support chronically deprived communities. Public Health Nutrition, 2001;4(6A): 1421-1424.

77. Campbell OM, Graham WJ; Lancet Maternal Survival Series steering group. Strategies for reducing maternal mortality: getting on with what works. Lancet. 2006;368(9543):1284-99.
78. Carr-Hill RA. Efficiency and equity implications of the health care reforms. Soc Sci Med. 1994;39(9):1189-201.
79. Carrin G, Waelkens MP, Criel B. Community-based health insurance in developing countries: a study of its contribution to the performance of health financing systems. Trop Med Int Health. 2005;10(8):799-811.
80. Carrin G. Community financing of drugs in Sub-Saharan Africa. Int J Health Plann Manage. 1987;2(2):125-45.
81. Carroli G, Villar J, Piaggio G, Khan-Neelofur D, Gülmezoglu M, Mugford M, Lumbiganon P, Farnot U, Bersgjø P; WHO Antenatal Care Trial Research Group. WHO systematic review of randomised controlled trials of routine antenatal care. Lancet. 2001;357(9268):1565-70.
82. Catsambas TT, LM Franco, M Gutmann, E Knebel, P Hill, and Y-S Lin. 2008. Evaluating Health Care Collaboratives: The Experience of the Quality Assurance Project. Collaborative Evaluation Series. Published by the USAID Health Care Improvement Project. Bethesda, MD: University Research Co., LLC (URC). available at: http://www.hciproject.org/Pubs/PDFs/EvaluatingHCCollaboratives.pdf, accessed May 4, 2009.
83. Chaix-Couturier C, Durand-Zaleski I, Jolly D, Durieux P. Effects of financial incentives on medical practice: results from a systematic review of the literature and methodological issues. Int J Qual Health Care. 2000 Apr;12(2):133-42.
84. Chalker, JC. Interventions for improved prescribing and dispensing of medicines in Nepal, Thailand and Vietnam. PhD Dissertation. Division of International Health, Department of Public Health Sciences, Karolinska Institutet, Stockholm, Sweden. 2003. available at: http://diss.kib.ki.se/2003/91-7349-690-1/, accessed May 21, 2010.
85. Chandler CI, Jones C, Boniface G, Juma K, Reyburn H, Whitty CJ. Guidelines and mindlines: why do clinical staff over-diagnose malaria in Tanzania? A qualitative study. Malar J. 2008;7:53.
86. Chase E, Carr-Hill R. The dangers of managerial perversion: quality assurance in primary health care. Health Policy Plan. 1994;9(3):267-72; discussion 272-8.
87. Chaudhury RR, Parameswar R, Gupta U, Sharma S, Tekur U, Bapna JS. Quality medicines for the poor: experience of the Delhi programme on rational use of drugs. HEALTH POLICY AND PLANNING, 2005;20(2): 124–136.
88. Cheah TS. The impact of clinical guidelines and clinical pathways on medical practice: effectiveness and medico-legal aspects. Ann Acad Med Singapore. 1998;27(4):533-9.
89. Chee, G. Working with Private Providers to Improve the Delivery of Priority Services. PHR Primer. 2004.
90. Chinnock P, Siegfried N, Clarke M. Is evidence-based medicine relevant to the developing world? PLoS Med. 2005;2(5):e107.
91. Christakis DA, Garrison MM, Ebel BE, Wiehe SE, Rivara FP. Pediatric smoking prevention interventions delivered by care providers: a systematic review. Am J Prev Med. 2003;25(4):358-62.
92. Christianson J, Leatherman S, Sutherland K. Financial incentives, healthcare providers and quality improvements. A review of the evidence. Quest for Quality and Improved

Performance (QQUIP), The Health Foundation, 2007. available at: http://www.hrhresourcecenter.org/node/1941, accessed May 21, 2010.
93. Christianson J, Leatherman S, Sutherland K. Financial incentives, healthcare providers and quality improvements. A review of the evidence. The Health Foundation: London, 2007.
94. Colclough, C. Marketing Education and Health in Developing Countries. Miracle or Mirage? Clarendon Press, Oxford: 1997.
95. Collins PY, Holman AR, Freeman MC, Patel V. What is the relevance of mental health to HIV/AIDS care and treatment programs in developing countries? A systematic review. Aids, 2006;20(12):1571-1582.
96. Crandall LA, Coggan JM. Impact of new information technologies on training and continuing education for rural health professionals. J Rural Health. 1994;10(3):208-15.
97. Creese A, Floyd K, Alban A, Guinness L. Cost-effectiveness of HIV/AIDS interventions in Africa: a systematic review of the evidence. Lancet. 2002 May 11;359(9318):1635-43.
98. Creese AL. User charges for health care: a review of recent experience. Health Policy Plan. 1991 Dec;6(4):309-19.
99. Croghan TW, Beatty A, Ron A. Routes to Better Health for Children in Four Developing Countries. The Milbank Quarterly, 2006; 84(2): 333–358
100. Cuca R, Pierce CS. Experiments in family planning: lessons from the developing world. The Johns Hopkins University Press, Baltimore: 1978, 276 pages.
101. Cutts F, Olive J-M. Vaccination Programs in Developing Countries. In: Plotkin SA and Orenstein WA, editors. Vaccines. Philadelphia: W.B Saunders, 1999, pp. 1047-1073.
102. Daniels N, Flores W, Pannarunothai S, Ndumbe PN, Bryant JH, Ngulube TJ, Wang YK. An evidence-based approach to benchmarking the fairness of health-sector reform in developing countries. Bull World Health Organ, 2005;83(7):534-540.
103. Darmstadt GL, Bhutta ZA, Cousens S, Adam T, Walker N, de Bernis L; Lancet Neonatal Survival Steering Team. Evidence-based, cost-effective interventions: how many newborn babies can we save? Lancet. 2005 Mar 12-18;365(9463):977-88.
104. Darras C. Local health services: some lessons from their evolution in Bolivia. Trop Med Int Health. 1997;2(4):356-62.
105. Davey P, Brown E, Fenelon L, Finch R, Gould I, Hartman G, Holmes A, Ramsay C, Taylor E, Wilcox M, Wiffen P. Interventions to improve antibiotic prescribing practices for hospital inpatients. Cochrane Database Syst Rev. 2005 Oct 19;(4):CD003543.
106. Davis DA, Thomson MA, Oxman AD, Haynes RB. Changing physician performance. A systematic review of the effect of continuing medical education strategies. JAMA. 1995;274(9):700-5.
107. De Brouwere V, Van Leberghe W (editors). Safe Motherhood Strategies: A Review of the Evidence. Studies in Health Services Organisation & Policy, 17, 2001. ITG Press, Antwerp. available at: http://www.jsieurope.org/safem/cgi-bin/library.fcgi, accessed Jan 20, 2009 [hand-searched the list of publications in the 'Articles' section on the website]
108. De Geyndt W. Improving the quality of health care in Latin America. Int J Qual Health Care, 2001;13:85-7.
109. De Geyndt, W. Managing the quality of health care in developing countries. World Bank Technical Paper Series, Number 258. The World Bank, 1995.

110.Dehne K, Snow R. Integrating STI management into family planning services: what are the benefits? World Health Organization, Geneva: 1999. available at: http://whqlibdoc.who.int/hq/1999/WHO_RHR_99.10_eng.pdf, accessed April 1, 2010.
111.Dehne KL, Snow R, O'Reilly KR. Integration of prevention and care of sexually transmitted infections with family planning services: what is the evidence for public health benefits? Bull World Health Organ. 2000;78(5):628-39.
112.Delnoij DM, Klazinga NS, Van der Velden K. Building integrated health systems in central and eastern Europe - An analysis of WHO and World Bank views and their relevance to health systems in transition. Eur J Public Health, 2003;13:240-5.
113.Demedts V, Schoonbaert D, Roelants G. Organization of health care in developing countries, selected publications (1980-2001). April 2001. The Library, Prince Leopold Institute of Tropical Medicine, Antwerp.
114.Demedts V, Schoonbaert D. Organization of health care in developing countries, selected publications (1990-2004). June 2004. The Library, Prince Leopold Institute of Tropical Medicine, Antwerp.
115.Devereaux PJ, Choi PT, Lacchetti C, Weaver B, Schünemann HJ, Haines T, Lavis JN, Grant BJ, Haslam DR, Bhandari M, Sullivan T, Cook DJ, Walter SD, Meade M, Khan H, Bhatnagar N, Guyatt GH. A systematic review and meta-analysis of studies comparing mortality rates of private for-profit and private not-for-profit hospitals. CMAJ. 2002;166(11):1399-406.
116.Devereaux PJ, Schünemann HJ, Ravindran N, Bhandari M, Garg AX, Choi PT, Grant BJ, Haines T, Lacchetti C, Weaver B, Lavis JN, Cook DJ, Haslam DR, Sullivan T, Guyatt GH. Comparison of mortality between private for-profit and private not-for-profit hemodialysis centers: a systematic review and meta-analysis. JAMA. 2002 Nov 20;288(19):2449-57. Review. Erratum in: JAMA. 2004 Jan 14;291(2):186.
117.Doggett C, Burrett S, Osborn DA. Home visits during pregnancy and after birth for women with an alcohol or drug problem. Cochrane Database Syst Rev. 2005;(4):CD004456.
118.Dolea C, Adams O. Motivation of health care workers - review of theories and empirical evidence. Cahiers de sociologie et de démographie médicales, 2005;45(1):135-162.
119.Doumit G, Gattellari M, Grimshaw J, O'Brien MA. Local opinion leaders: effects on professional practice and health care outcomes. Cochrane Database Syst Rev. 2007;(1):CD000125. Update of: Thomson O'Brien MA, Oxman AD, Haynes RB, Davis DA, Freemantle N, Harvey EL. Local opinion leaders: effects on professional practice and health care outcomes. Cochrane Database Syst Rev. 2000;(2):CD000125.
120.Dovlo, D. Using mid-level cadres as substitutes for internationally mobile health professionals in Africa. A desk review. Human Resources for Health, 2004;2(1):7.
121.Dreschler D, Jutting JP. Private health insurance in low- and middle-income countries. Scope, limitations, and policy responses. OECD Development Centre, March 2005.
122.Duke T, Tamburlini G, Silimperi D; Paediatric Quality Care Group. Improving the quality of paediatric care in peripheral hospitals in developing countries. Arch Dis Child. 2003 Jul;88(7):563-5.
123.Duke, T. Randomised trials in child health in developing countries. 3rd Edition, July 2004 - June 2005. Centre for International Child Health, University Department of Paediatrics, Royal Children's Hospital, Victoria, Australia. October 18, 2005. available at:

http://www.ichrc.org/RCT%20child%20health/RCTs%20in%20child%20health%20in%20developing%20countries%202004-2005.pdf, accessed March 16, 2010.
124. Durieux P, Trinquart L, Colombet I, Niès J, Walton R, Rajeswaran A, Rège Walther M, Harvey E, Burnand B. Computerized advice on drug dosage to improve prescribing practice. Cochrane Database Syst Rev. 2008;(3):CD002894.
125. Egger D, Lipson D, Adams O. Achieving the right balance: The role of policy-making processes in managing human resources for health problems. Issues in health services delivery, Discussion paper No. 2. WHO/EIP/OSD/00.2. Evidence and Information for Policy, Department of Organization of Health Services Delivery, World Health Organization: Geneva, 2000.
126. Eggleston K, Ling L, Qingyue M, Lindelow M, Wagstaff A. Health service delivery in China: a literature review. Health Econ. 2008 Feb;17(2):149-65.
127. Eggleston K, Ling L, Qingyue M, Lindelow M, Wagstaff A. Health service delivery in China: a literature review. World Bank Policy Research Working Paper 3978, August 2006.
128. Egwu IN. Selective interventions in primary health care: planning and practical implications in Nigeria. International Journal of Health Planning and Management, 1987;2:201-211.
129. Eichler, R. Can "Pay for Performance" increase utilization by the poor and improve the quality of health services? Discussion paper for the first meeting of the Pay for Performance Working Group, Center for Global Development. February 7, 2006. available at: http://www.cgdev.org/doc/ghprn/PBI%20Background%20Paper.pdf, accessed May 21, 2010.
130. Ekman B. Community-based health insurance in low-income countries: a systematic review of the evidence. Health Policy Plan. 2004 Sep;19(5):249-70.
131. El Ansari W, Phillips CJ, Hammick M. Collaboration and partnerships: developing the evidence base. Health Soc Care Community. 2001;9(4):215-27.
132. Elkan R, Kendrick D, Hewitt M, Robinson JJ, Tolley K, Blair M, Dewey M, Williams D, Brummell K. The effectiveness of domiciliary health visiting: a systematic review of international studies and a selective review of the British literature. Health Technol Assess. 2000;4(13):i-v, 1-339.
133. England, R. Experiences of contracting with the private sector. A selective review. March 2004. DFID Health Systems Resource Centre.
134. Ensor T, Cooper S. Overcoming Barriers to Health Service Access and Influencing the Demand Side Through Purchasing. HNP Discussion Paper. September 2004.
135. Ensor T, Cooper S. Overcoming barriers to health service access: influencing the demand side. Health Policy and Planning, 2004;19(2): 69–79.
136. Ensor T. Consumer-led Demand Side Financing for Health and Education: An International Review. The Assessment of Health Care Financing Options for the Poor. July 2003. FINAL DRAFT. WHO/BAN/DSF/03.1
137. Ensor T. Consumer-led demand side financing in health and education and its relevance for low and middle income countries. Int J Health Plann Manage. 2004 Jul-Sep;19(3):267-85.
138. Espallargues M, Valderas JM, Alonso J. Provision of feedback on perceived health status to health care professionals: a systematic review of its impact. Med Care. 2000;38(2):175-86.

139. Evans DB, Edejer TT, Adam T, Lim SS. Methods to assess the costs and health effects of interventions for improving health in developing countries. BMJ. 2005;331(7525):1137-40.
140. Evans, DB. Cost-effectiveness analysis of interventions at the community level. Expert Committee on Malaria, Geneva, 19 to 27 October 1998, Draft Agenda Item 8. World Health Organization, Geneva: 1998. MAL/EC20/98.26.
141. Fairbank L, O'Meara S, Renfrew MJ, Woolridge M, Sowden AJ, Lister-Sharp D. A systematic review to evaluate the effectiveness of interventions to promote the initiation of breastfeeding. Health Technol Assess. 2000;4(25):1-171. available at: http://www.hta.ac.uk/fullmono/mon425.pdf, accessed March 16, 2010.
142. Farquhar CM, Stryer D, Slutsky J. Translating research into practice: the future ahead. Int J Qual Health Care. 2002;14(3):233-49.
143. Feachem RG, Hogan RC, Merson MH. Diarrhoeal disease control: reviews of potential interventions. Bull World Health Organ. 1983;61(4):637-40.
144. Feachem RG, Koblinsky MA. Interventions for the control of diarrhoeal diseases among young children: measles immunization. Bull World Health Organ. 1983;61(4):641-52.
145. Feachem RG, Koblinsky MA. Interventions for the control of diarrhoeal diseases among young children: promotion of breast-feeding. Bull World Health Organ. 1984;62(2):271-91.
146. Feachem RG. Interventions for the control of diarrhoeal diseases among young children: promotion of personal and domestic hygiene. Bull World Health Organ. 1984;62(3):467-76.
147. Feachem RG. Interventions for the control of diarrhoeal diseases among young children: supplementary feeding programmes. Bull World Health Organ. 1983;61(6):967-79.
148. Fielding D, Duff A. Compliance with treatment protocols: interventions for children with chronic illness. Arch Dis Child. 1999;80(2):196-200.
149. Figley MM, Margulis AR. The impact of new imaging technology on health care, research, and teaching: an international symposium. AJR Am J Roentgenol. 1987;149(6):1111-26.
150. Fixsen DL, Naoom SF, Blase KA, Friedman RM, Wallace F. Implementation Research: A Synthesis of the Literature. Tampa, FL: University of South Florida, Louis de la Parte Florida Mental Health Institute, The National Implementation Research Network (FMHI Publication #231). 2005.
151. Flynn MA, McNeil DA, Maloff B, Mutasingwa D, Wu M, Ford C, Tough SC. Reducing obesity and related chronic disease risk in children and youth: a synthesis of evidence with 'best practice' recommendations. Obes Rev. 2006;7 Suppl 1:7-66.
152. Forsetlund L, Bjørndal A, Rashidian A, Jamtvedt G, O'Brien MA, Wolf F, Davis D, Odgaard-Jensen J, Oxman AD. Continuing education meetings and workshops: effects on professional practice and health care outcomes. Cochrane Database Syst Rev. 2009 Apr 15;(2):CD003030. Update of: O'Brien MA, Freemantle N, Oxman AD, Wolf F, Davis DA, Herrin J. Cochrane Database Syst Rev. 2001;(2):CD003030.
153. Foster P, Bezmalinovic B, Curtis C, Farrell B, Maitra K, Trayfors W. Evaluation of the Training in Reproductive Health (TRH II) Project. LTG Associates, Inc.; TvT Global Health and Development Strategies. POPTECH Publication Number 2002–069–011. Population Technical Assistance Project, Washington DC: January 2003.

154. Foster SO. Thirteen lessons learned -- 1981-1993. Africa Child Survival Initiative (ACSI), Combatting Childhood Communicable Diseases (CCCD). Implementation experience, 13 African countries: Burundi, CAR, Congo, Cote d'Ivoire, Guinea, Lesotho, Liberia, Malawi, Nigeria, Rwanda, Swaziland, Togo, Zaire. United States Agency for International Development (USAID) and Centers for Disease Control and Prevention (CDC), in collaboration with World Health Organization (WHO), United Nations Children's Fund (UNICEF), Rotary International. 1993. 35 pages
155. Franco LM, Bennett S, Kanfer R. Health sector reform and public sector health worker motivation: a conceptual framework. Soc Sci Med. 2002;54(8):1255-66.
156. Freemantle N, Bloor K. Lessons from international experience in controlling pharmaceutical expenditure. I: Influencing patients. BMJ. 1996;312(7044):1469-71.
157. Fritzen, SA. Strategic management of the health workforce in developing countries: what have we learned? Human Resources for Health, 2007;5:4.
158. Fullerton J, Piet-Pelon NJ, Trayfors W, Wilcox S. EVALUATION OF PRIME II. October 2003. LTG Associates, Inc.
159. García-Moreno C. Dilemmas and opportunities for an appropriate health-service response to violence against women. Lancet. 2002;359(9316):1509-14.
160. Garcia-Prado A. Sweetening the Carrot. Motivating public physicians for better performance. World Bank Policy Research Working Paper 3772, November 2005. Washington DC: World Bank.
161. Garg AX, Adhikari NK, McDonald H, Rosas-Arellano MP, Devereaux PJ, Beyene J, Sam J, Haynes RB. Effects of computerized clinical decision support systems on practitioner performance and patient outcomes: a systematic review. JAMA. 2005;293(10):1223-38.
162. Gauri, V. Are incentives everything? Payment mechanisms for health care providers in developing countries. World Bank Policy Research Working Paper 2624, 2001.
163. Gilroy K, Winch P. Management of sick children by community health workers. Intervention models and programme examples. WHO, UNICEF: Geneva, 2006. available at: http://whqlibdoc.who.int/publications/2006/9789280639858_eng.pdf, accessed March 17, 2010.
164. Gilson L, McIntyre D. Removing user fees for primary care in Africa: the need for careful action. BMJ. 2005;331(7519):762-5.
165. Gilson L, Mills A. Health sector reforms in sub-Saharan Africa: lessons of the last 10 years. Health Policy. 1995;32(1-3):215-43.
166. Glassman A, Todd J, Gaarder M. Chapter 6: Latin America: Cash Transfers to Support Better Household Decisions. p. 89-121. In: Eichler R, Levine R, and the Performance-Based Incentives Working Group. Performance incentives for global health: potential and pitfalls. Versa Press: East Peoria, Illinois. 2009. available at: http://www.cgdev.org/doc/books/PBI/06_CGD_Eichler_Levine-Ch6.pdf; accessed July 7, 2009.
167. Glassman A, Todd JE, Gaarder M. Performance-Based Incentives for Health: Conditional Cash Transfer Programs in Latin America and the Caribbean (April 23, 2007). Center for Global Development Working Paper No. 120. Available at SSRN: http://ssrn.com/abstract=1003250. accessed June 12, 2009

168. Gnecco G, Lucero S, Bassi A, Loncomilla R, Brown LD. Making a Commitment to Quality: Development of a National Quality Assurance Program in Chile 1991–1998. Quality Assurance Project, Center for Human Services, Bethesda: 1999.
169. Gomes M, Pang L. (editors) Interventions to improve antimalarial use. WHO Drug Information, 1998; 76(Suppl 1).
170. Gomes, M. Improving the compliance of antimalarials. WHO Drug Information, 1997; 11(4): 236-239.
171. González M, Colindres H, Urroz O, Orlich J, Veldhuyzen van Zanten T. Health Care Sector Reform and Quality Assurance in Costa Rica. Results of Process Improvement Teams Working in Health Facilities Managed by the Costa Rican Social Security Institute. Latin America and Caribbean Health and Nutrition Sustainability, Quality Assurance Project, Center for Human Services, Bethesda, Maryland, USA.
172. Goodman C, Brieger W, Unwin A, Mills A, Meek S, Greer G. Medicine sellers and malaria treatment in sub-Saharan Africa: what do they do and how can their practice be improved? Am J Trop Med Hyg. 2007;77(6 Suppl):203-18.
173. Goodman CA, Mills AJ. The evidence base on the cost-effectiveness of malaria control measures in Africa. Health Policy Plan. 1999;14(4):301-12.
174. Goodman CA, Mutemi WM, Baya EK, Willetts A, Marsh V. The cost-effectiveness of improving malaria home management: shopkeeper training in rural Kenya. Health Policy Plan. 2006;21(4):275-88.
175. Gorter A, et al. Competitive Voucher Schemes for Health. Background Paper. August 2003. Instituto Centro Americano de la Salud (ICAS). available at: http://www.icas.net/new-icasweb/docs/Background%20Paper%20Competitive%20vouchers.doc, accessed Dec 10, 2008
176. Gottret P, Schieber G. Health Financing Revisited: A Practioner's Guide. World Bank, 2006. Available at: http://siteresources.worldbank.org/INTHSD/Resources/topics/Health-Financing/HFRFull.pdf, accessed Sept 7, 2009.
177. Grace C, James J, Hadi Y. Selective Review of Work Aids For Alternative Health Care Providers In Developing Countries, June 2008. Health and Life Sciences Partnership (HLSP).
178. Grace C. Developing new technologies to address neglected diseases: The role of Product Development Partnerships and Advanced Market Commitments, June 2006. London: DFID Health Resource Centre.
179. Graham W, Wagaarachchi P, Penney G, McCaw-Binns A, Antwi KY, Hall MH. Criteria for clinical audit of the quality of hospital-based obstetric care in developing countries. Bull World Health Organ. 2000;78(5):614-20.
180. Graham WJ, Bell JS, Bullough CH. Can skilled attendance at delivery reduce maternal mortality in developing countries? In: Safe motherhood strategies: a review of the evidence, edited by Vincent De Brouwere, Wim Van Lerberghe. Antwerp, Belgium, ITGPress, 2001. :97-129. (Studies in Health Services Organisation and Policy No. 17)
181. Green, CP. Mother Support Groups. A Review of Experience in Developing Countries. Published for the US Agency for International Development (USAID) by the Basic Support for Institutionalizing Child Survival (BASICS) Project, Arlington, VA. 1998.
182. Green, CP. Strategic Management of Family Planning Programs. Policy Research Working Paper Series No. 976. The World Bank, Washington DC, September 1992.

183. Greenhalgh J, Meadows K. The effectiveness of the use of patient-based measures of health in routine practice in improving the process and outcomes of patient care: a literature review. J Eval Clin Pract. 1999;5(4):401-16.
184. Grilli R, Freemantle N, Minozzi S, Domenighetti G, Finer D. [Impact of mass media on the use of health services. A systematic review of the literature] Epidemiol Prev. 1998;22(2):103-10.
185. Grimshaw JM, Russell IT. Effect of clinical guidelines on medical practice: a systematic review of rigorous evaluations. Lancet. 1993;342(8883):1317-22.
186. Grimshaw JM, Shirran L, Thomas R, Mowatt G, Fraser C, Bero L, Grilli R, Harvey E, Oxman A, O'Brien MA. Changing provider behavior: an overview of systematic reviews of interventions. Med Care. 2001;39(8 Suppl 2):II2-45.
187. Grimshaw JM, Thomas RE, MacLennan G, Fraser C, Ramsay CR, Vale L, Whitty P, Eccles MP, Matowe L, Shirran L, Wensing M, Dijkstra R, Donaldson C. Effectiveness and efficiency of guideline dissemination and implementation strategies. Health Technol Assess. 2004;8(6):iii-iv, 1-72.
188. Grindrod KA, Patel P, Martin JE. What interventions should pharmacists employ to impact health practitioners' prescribing practices? Ann Pharmacother. 2006;40(9):1546-57.
189. Grol R, Grimshaw J. From best evidence to best practice: effective implementation of change in patients' care. Lancet. 2003;362(9391):1225-30.
190. Grol R. Improving the quality of medical care: building bridges among professional pride, payer profit, and patient satisfaction. JAMA. 2001;286(20):2578-85.
191. Gross PA, Pujat D. Implementing practice guidelines for appropriate antimicrobial usage: a systematic review. Med Care. 2001;39(8 Suppl 2):II55-69.
192. Gruen RL, Weeramanthri TS, Knight SE, Bailie RS. Specialist outreach clinics in primary care and rural hospital settings. Cochrane Database Syst Rev 2004: CD003798.
193. Gulliford MC, Picou DI. Health services research in the English-speaking Caribbean 1984-93: a quantitative review. Health Policy Plan. 1995;10(2):191-7.
194. Gulliford MC. Controlling non-insulin-dependent diabetes-mellitus in developing countries. Int J Epidemiol, 1995;24:S53-S59.
195. Gwatkin DR, Wagstaff A, Yazbeck AS. Reaching the Poor with Health, Nutrition, and Population Services. What Works, What Doesn't, and Why. The World Bank, Washington, DC. 2005.
196. Gwatkin, DR. Reducing Health Inequities in Developing Countries. Pages 1791-1810. In: Detels R, McEwen J, Beaglehole R, Tanaka H (editors). Oxford Textbook of Public Health. Oxford University Press: New York, 2002.
197. Gwynne G, Zschock DK. Special supplement. Health care financing in Latin America and the Caribbean J Health Adm Educ. 1990;8(4 Pt 2):741-930.
198. Gyapong M, Garshong B, 2007. Lessons Learned in Home Management of Malaria: Implementation Research in Four African Countries. Geneva: WHO.available at: http://www.who.int/tdr/publications/training-guideline-publications/lessons-home-management-malaria/pdf/lessons_hmm.pdf. Accessed April 30, 2009.
199. Gysels M, Richardson A, Higginson IJ. Communication training for health professionals who care for patients with cancer: a systematic review of training methods. Support Care Cancer. 2005;13(6):356-66.

200. Haak H, Hogerzeil HV. Essential drugs for ration kits in developing countries. Health Policy Plan. 1995;10(1):40-9.
201. Haberland N, Measham DM. (Editors) Responding to Cairo: Case Studies of Changing Practice in Reproductive Health and Family Planning. Population Council, March 2002, 462 pages. ISBN-10: 0878341064, ISBN-13: 978-0878341061
202. Habibula S, Sethi D, Maree-Kelly A. Advanced trauma life support training for hospital staff. Cochrane Database of Systematic Reviews, 2004: CD004173.
203. Hadley M, Maher D. Community involvement in tuberculosis control: lessons from other health care programmes. Int J Tuberc Lung Dis, 2000; 4(5):401–408.
204. Hahn RA, Bilukha OO, Crosby A, Fullilove MT, Liberman A, Moscicki EK, Snyder S, Tuma F, Schofield A, Corso PS, Briss P; Task Force on Community Preventive Services. First reports evaluating the effectiveness of strategies for preventing violence: early childhood home visitation. Findings from the Task Force on Community Preventive Services. MMWR Recomm Rep. 2003 Oct 3;52(RR-14):1-9.
205. Haines A, Kuruvilla S, Borchert M. Bridging the implementation gap between knowledge and action for health. Bull World Health Organ. 2004;82(10):724-31
206. Haines A, Sanders D, Lehmann U, Rowe AK, Lawn JE, Jan S, Walker DG, Bhutta Z. Achieving child survival goals: potential contribution of community health workers. Lancet. 2007;369(9579):2121-31.
207. Hales, DJ. Promoting breastfeeding: strategies for changing hospital policy. Studies in Family Planning, 1981;12(4):167-172.
208. Halm EA, Lee C, Chassin MR. Is volume related to outcome in health care? A systematic review and methodologic critique of the literature. Ann Intern Med. 2002;137(6):511-20.
209. Hampson SE, Skinner TC, Hart J, Storey L, Gage H, Foxcroft D, Kimber A, Shaw K, Walker J. Effects of educational and psychosocial interventions for adolescents with diabetes mellitus: a systematic review. Health Technol Assess. 2001;5(10):1-79.
210. Hargraves T-L, Bennett AA, Brien JE. Evaluating outpatient pharmacy services: a literature review of specialist heart failure services. International Journal of Pharmacy Practice, 2006;14(1):3-9.
211. Harling G, Wood R, Beck EJ. Efficiency of Interventions in HIV Infection, 1994–2004. Dis Manage Health Outcomes 2005;13(6):371-394.
212. Harrington J, Noble LM, Newman SP. Improving patients' communication with doctors: a systematic review of intervention studies. Patient Educ Couns. 2004;52(1):7-16.
213. Harvey EL, Glenny A, Kirk SF, Summerbell CD. Improving health professionals' management and the organisation of care for overweight and obese people. Cochrane Database Syst Rev. 2001;(2):CD000984.
214. Harvey SA, Jennings L, Chinyama M, Masaninga F, Mulholland K, Bell DR. Improving community health worker use of malaria rapid diagnostic tests in Zambia: package instructions, job aid and job aid-plus-training. Malar J. 2008;7:160.
215. Haws R, Winch PJ, Castillo J. Innovative community-based interventions to improve newborn health in Latin America and the Caribbean. The CORE Group, Johns Hopkins University, August 2004.
216. Haws RA, Darmstadt GL. Integrated, evidence-based approaches to save newborn lives in developing countries. Arch Pediatr Adolesc Med. 2007;161(1):99-102.
217. Haws RA, Thomas AL, Bhutta ZA, Darmstadt GL. Impact of packaged interventions on neonatal health: a review of the evidence. Health Policy Plan. 2007;22(4):193-215.

218.Heath JA. The financing and provisioning of education and health services in developing countries. Review article. Economics of Education Review, 1998;17:359-62.
219.Hecht R, Batson A, Brenzel L. Making Health Care Accountable. Finance & Development; Mar 2004; 41, 1; ABI/INFORM Research. pg. 16-19.
220.Heerey M, Merritt AP, Kols AJ. Improving the quality of care. Quality improvement projects from The Johns Hopkins University, Bloomberg School of Public Health, Center for Communication Programs, Center Publication No. 101. February 2003.
221.Heneghan AM, Horwitz SM, Leventhal JM. Evaluating intensive family preservation programs: a methodological review. Pediatrics. 1996;97(4):535-42.
222.Hersh WR, Helfand M, Wallace J, Kraemer D, Patterson P, Shapiro S, Greenlick M. Clinical outcomes resulting from telemedicine interventions: a systematic review. BMC Med Inform Decis Mak. 2001;1:5. Epub 2001 Nov 26.
223.Hicks V, Adams O. The effects of economic and policy incentives on provider practice. Issues in health services delivery. Discussion paper No. 5. Evidence and Information for Policy, Department of Organization of Health Services Delivery, World Health Organization: Geneva, 2000.
224.Higginson IJ, Finlay I, Goodwin DM, Cook AM, Hood K, Edwards AG, Douglas HR, Norman CE. Do hospital-based palliative teams improve care for patients or families at the end of life? J Pain Symptom Manage. 2002;23(2):96-106.
225.Hinkin J. Hand decontamination: what interventions improve compliance? EDTNA ERCA J. 2002;28(3):134-7.
226.Hodnett ED. WITHDRAWN: Continuity of caregivers for care during pregnancy and childbirth. Cochrane Database Syst Rev. 2008;(4):CD000062.
227.Hogan DR, Baltussen R, Hayashi C, Lauer JA, Salomon JA. Cost effectiveness analysis of strategies to combat HIV/AIDS in developing countries. BMJ 2005;331;1431-1437.
228.Hogerzeil HV, et al. Field tests for rational drug use in twelve developing countries. Lancet. 1993 Dec 4;342(8884):1408-10.
229.Homedes N, Ugalde A, Chaumont C. Scientific evaluations of interventions to improve the adequate use of pharmaceuticals in Third World countries. Public Health Rev. 2001;29(2-4):207-30.
230.Homedes N, Ugalde A. Improving the use of pharmaceuticals through patient and community level interventions. Soc Sci Med. 2001;52(1):99-134.
231.Hopkins H, Talisuna A, Whitty CJM, Staedke SG. Impact of home-based management of malaria on health outcomes in Africa: a systematic review of the evidence. Malaria Journal 2007, 6:134. doi:10.1186/1475-2875-6-134
232.Horton S, Sanghvi T, Phillips M, Fiedler J, Perez-Escamilla R, Lutter C, Rivera A, Segall-Correa AM. Breastfeeding promotion and priority setting in health. Health Policy Plan. 1996;11(2):156-68.
233.Hubley J. Patient education in the developing world--a discipline comes of age. Patient Educ Couns. 2006 Apr;61(1):161-4.
234.Huddart J, Picazo OF, Duale S. The Health Sector Human Resource Crisis in Africa: An Issues Paper. Support for Analysis and Research in Africa (SARA) Project, Academy for Educational Development, Washington, DC: February 2003.
235.Hulsman RL, Ros WJ, Winnubst JA, Bensing JM. Teaching clinically experienced physicians communication skills. A review of evaluation studies. Med Educ. 1999;33(9):655-68.

236. Hunt DL, Haynes RB, Hanna SE, Smith K. Effects of computer-based clinical decision support systems on physician performance and patient outcomes: a systematic review. JAMA. 1998;280(15):1339-46.
237. International Council of Nurses, International Hospital Federation, International Pharmaceutical Federation, World Confederation for Physical Therapy, World Dental Federation, World Medical Association.Guidelines: incentives for health professionals. 2008. available at: http://www.who.int/workforcealliance/documents/Incentives_Guidelines%20EN.pdf, accessed May 13, 2009
238. Ioannidis JP, Lau J. Evidence on interventions to reduce medical errors: an overview and recommendations for future research. J Gen Intern Med. 2001;16(5):325-34.
239. Ipas, IHCAR. Deciding women's lives are worth saving: Expanding the role of midlevel providers in safe abortion care. Issues in Abortion Care 7. April 15, 2002. available at: http://www.ipas.org/Publications/asset_upload_file460_2494.pdf, accessed January 21, 2010.
240. James C, Morris SS, Keith R, Taylor A. Impact on child mortality of removing user fees: simulation model. BMJ 2005;331;747-749
241. Jamtvedt G, Young JM, Kristoffersen DT, O'Brien MA, Oxman AD. Audit and feedback: effects on professional practice and health care outcomes. Cochrane Database Syst Rev. 2006;(2):CD000259.
242. Jan S, Bian Y, Jumpa M, Meng Q, Nyazema N, Prakongsai P, Mills A. Dual job holding by public sector health professionals in highly resource-constrained settings: problem or solution? Bull World Health Organ. 2005;83(10):771-6.
243. Janovsky K, Peters D. Improving health services and strengthening health systems: adopting and implementing innovative strategies. An exploratory review in twelve countries. Making Health Systems Work: Working Paper No. 5. WHO/EIP/healthsystems/2006.2.
244. Jarrett SW, Ofosu-Amaah S. Strengthening health services for MCH in Africa: the first four years of the 'Bamako Initiative'. Health Policy and Planning, 1992;7(2):164-176.
245. Jayaraman S, Sethi D. Advanced trauma life support training for ambulance crews. Cochrane Database Syst Rev. 2010;(1):CD003109. Update of: Cochrane Database Syst Rev. 2001;(2):CD003109.
246. Johns B, Torres TT; WHO-CHOICE. Costs of scaling up health interventions: a systematic review. Health Policy Plan. 2005;20(1):1-13.
247. Johnson A, Sandford J, Tyndall J. Written and verbal information versus verbal information only for patients being discharged from acute hospital settings to home. Cochrane Database Syst Rev. 2003;(4):CD003716.
248. Johnston G, Crombie IK, Davies HT, Alder EM, Millard A. Reviewing audit: barriers and facilitating factors for effective clinical audit. Qual Health Care. 2000;9(1):23-36.
249. Johnston ME, Langton KB, Haynes RB, Mathieu A. Effects of computer-based clinical decision support systems on clinician performance and patient outcome. A critical appraisal of research. Ann Intern Med. 1994;120(2):135-42.
250. Jonas, E. Improving provider performance - an exploration of the literature. MotherCare Matters, 2000;9(2):1-23.
251. Kadaï A, Sall FL, Andriantsara G, Perrot J. The benefits of setting the ground rules and regulating contracting practices. Bull World Health Organ. 2006;84(11):897-902.

252.Kafle KK, Shrestha N, Karkee SB, Prasad RR, Bhuju GB, Das PL. Intervention studies on rational use of drugs in public and private sector in Nepal. Nepal Med Coll J, 2005;7:47-50
253.Kakai R, Wamola IA. Minimising antibiotic resistance to Staphylococcus aureus in developing countries. East Afr Med J. 2002;79(11):574-9.
254.Kaplan WA. Can the ubiquitous power of mobile phones be used to improve health outcomes in developing countries? Global Health. 2006;2:9.
255.Kattwinkel J, Cook LJ, Nowacek G, Bailey C, Crosby WM, Hurt H, Shorte J. Regionalized perinatal education. Seminars in Neonatology, 2004;9(2):155-165.
256.Kaushal R, Shojania KG, Bates DW. Effects of computerized physician order entry and clinical decision support systems on medication safety: a systematic review. Arch Intern Med. 2003;163(12):1409-16.
257.Kawamoto K, Houlihan CA, Balas EA, Lobach DF. Improving clinical practice using clinical decision support systems: a systematic review of trials to identify features critical to success. BMJ. 2005;330(7494):765. Epub 2005 Mar 14.
258.Kazanjian A, Green C, Wong J, Reid R. Effect of the hospital nursing environment on patient mortality: a systematic review. J Health Serv Res Policy. 2005;10(2):111-7.
259.Keller DS, Dermatis H. Current Status of Professional Training in the Addictions. Subst Abus. 1999;20(3):123-140.
260.Kelley LM, Black RE, editors. Research to support household and community IMCI. Report of a meeting, 22-24 January 2001, Baltimore, Maryland, USA. J Health Popul Nutr. 2001;19(2):S111-48.
261.Kerber KJ, de Graft-Johnson JE, Bhutta ZA, Okong P, Starrs A, Lawn JE. Continuum of care for maternal, newborn, and child health: from slogan to service delivery. Lancet. 2007;370(9595):1358-69.
262.Khaleghian P, Gupta MD. Public management and essential public health functions. World Bank Policy Research Working Paper WPS 3220, February 2004.
263.Kilminster SM, Jolly BC. Effective supervision in clinical practice settings: a literature review. Med Educ. 2000;34(10):827-40.
264.Knapp M, Funk M, Curran C, Prince M, Grigg M, McDaid D. Economic barriers to better mental health practice and policy. Health Policy Plan, 2006;21(3):157-70.
265.Knebel, E. The use and effect of computer-based training in health care: what do we know? QAP Operations Research Issue Paper. April 2000; Volume 2.
266.Knebel, E. The use and effect of distance education in healthcare: what do we know? QAP Operations Research Issue Paper. September 2001; Volume 1, Issue 2.
267.Knebel, E. The use of manual job aids by health care providers: what do we know? QAP Operations Research Issue Paper. February 2000; Volume 1.
268.Knippenberg R, Lawn JE, Darmstadt GL, Begkoyian G, Fogstad H, Walelign N, Paul VK; Lancet Neonatal Survival Steering Team. Systematic scaling up of neonatal care in countries. Lancet. 2005;365(9464):1087-98.
269.Koblinsky MA, Campbell O, Heichelheim J. Organizing delivery care: what works for safe motherhood? Bull World Health Organ. 1999;77(5):399-406.
270.Koblinsky MA, Tinker A, Daly P. Programming for safe motherhood: a guide to action. Health Policy Plan, 1994;9(3):252-66.
271.Koch-Weser D. Community-based education of the health professions in Latin America and the US. Am J Public Health, 1987;77(4):412.

272. Kolehmainen-Aitken R-L, Newbrander W. Lessons from FPMD. Decentralizing the management of Health and Familiy Planning programs. Management Sciences for Health, Newton, MA: 1997.
273. Kolehmainen-Aitken R-L. Decentralization's impact on the health workforce: Perspectives of managers, workers and national leaders. Management Sciences for Health, Boston, MA: 2004.
274. Kols MA, Sherman JL. Family planning programs: improving quality. Population Reports, Series J, Number 47. Baltimore: Johns Hopkins University School of Public Health, Population Information Program, November 1998.
275. Kwast BE. Quality of care in reproductive health programmes: monitoring and evaluation of quality improvement. Midwifery. 1998;14(4):199-206.
276. La Forgia GM, editor. World Bank Working Paper No. 57. Health System Innovations in Central America. Lessons and Impact of New Approaches. May 2005.
277. Lagarde M, Haines A, Palmer N. Conditional cash transfers for improving uptake of health interventions in low- and middle-income countries: a systematic review. JAMA. 2007;298(16):1900-10.
278. Laing R, Hogerzeil H, Ross-Degnan D. Ten recommendations to improve use of medicines in developing countries. Health Policy Plan. 2001;16(1):13-20.
279. Lambert VA, Lambert CE. Literature review of role stress/strain on nurses: an international perspective. Nurs Health Sci. 2001 Sep;3(3):161-72.
280. Lancaster T, Silagy C, Fowler G. Training health professionals in smoking cessation. Cochrane Database Syst Rev. 2000;(3):CD000214.
281. Langenbrunner J, Liu X. How To Pay. Understanding and Using Incentives. Health, Nutrition and Population (HNP) Discussion Paper. The World Bank, Washington DC: September 2004.
282. Laurant M, Reeves D, Hermens R, Braspenning J, Grol R, Sibbald B. Substitution of doctors by nurses in primary care. Cochrane Database Syst Rev. 2004;(2):CD001271.
283. le Grand A, Hogerzeil HV, Haaijer-Ruskamp FM. Intervention research in rational use of drugs: a review. Health Policy Plan. 1999;14(2):89-102.
284. Lehmann U, Friedman I, Sanders D. A joint learning initiative: human resources for health and development. Review of the utilisation and effectiveness of community-based health workers in Africa. JLI Working Paper 4-1, 2004.
285. Leighton C. Overview: health financing reforms in Africa. Health Policy Plan. 1995;10(3):213-22.
286. Lewin S, Munabi-Babigumira S, Glenton C, Daniels K, Bosch-Capblanch X, van Wyk BE, Odgaard-Jensen J, Johansen M, Aja GN, Zwarenstein M, Scheel IB. Lay health workers in primary and community health care for maternal and child health and the management of infectious diseases. Cochrane Database Syst Rev. 2010;3:CD004015.
287. Lewin SA, Babigumira SM, Bosch-Capblanch X, Aja G, van Wyk B, Glenton C, Scheel I, Zwarenstein M, Daniels K. Lay health workers in primary and community health care: A systematic review of trials. November 2006.
288. Lewis M, Petterson G. Governance in health care delivery. Raising performance. Polic Research Working Paper 5074. World Bank, October 2009, Document no. WPS5074. available at: http://www-wds.worldbank.org/servlet/WDSContentServer/WDSP/IB/2009/10/13/000158349_20091013151915/Rendered/PDF/WPS5074.pdf, accessed March 31, 2010

289. Lewis MA. Pricing and cost recovery experience in family planning programs. Washington, D.C., World Bank, 1985, 75 p. (World Bank Staff Working Papers No. 684, Population and Development Series No. 9)
290. Liebig, K. Ongoing Reforms of Latin American Health Systems: Can Market-Oriented Reforms Help to Solve the Problems? Social Justice in a Market Economy, 2001;113-133.
291. Little CD. Mandatory Continuing Education: A Survey of the Literature and a Comment on the Implications for Physical Therapy. J Contin Educ Health Prof, 1993; 13(2):159-167.
292. Liu X, Hotchkiss DR, Bose S, Bitran R, Giedion U. Contracting for primary health services: evidence on its effects and framework for evaluation. Bethesda, MD: Partners for Health Reformplus Project, Abt Associates, Inc. September 2004.
293. Loevinsohn B, Harding A. Buying results? Contracting for health service delivery in developing countries. Lancet, 2005; 366: 676–681.
294. Loevinsohn BP. Health education interventions in developing countries: a methodological review of published articles. Int J Epidemiol. 1990;19(4):788-94.
295. Lomas J. Words without action? The production, dissemination, and impact of consensus recommendations. Annu Rev Public Health. 1991;12:41-65.
296. Lönnroth K, Uplekar M, Blanc L. Hard gains through soft contracts: productive engagement of private providers in tuberculosis control. Bull World Health Organ. 2006;84(11):876-83.
297. Luck M. Safe motherhood intervention studies in Africa: a review. East Afr Med J. 2000;77(11):599-607.
298. Maceira D. Provider payment mechanisms in health care: incentives, outcomes, and organizational impact in developing countries. Major Applied Research 2, Working Paper 2, Abt Associates, Inc: Bethesda. August 1998.
299. Madden JM, Quick JD, Ross-Degnan D, Kafle KK. Undercover careseekers: simulated clients in the study of health provider behavior in developing countries. Soc Sci Med, 1997;45(10):1465-82.
300. Maher D, van Gorkom JLC, Gondrie PCFM, Raviglione M Community contribution to tuberculosis care in countries with high tuberculosis prevalence: past, present and future. Int J Tuberc Lung Dis, 1999; 3(9):762–768.
301. Makinwa B, O'Grady M, editors. FHI/UNAIDS Best Practices in HIV/AIDS Prevention Collection. Family Health International, Joint United Nations Programme on HIV/AIDS: Arlington, VA, USA. 2001. available at: http://www.fhi.org/en/HIVAIDS/pub/guide/bestpractices.htm, accessed April 1, 2010.
302. Management Sciences for Health, Euro Health Group. Managing Drug Supply : The Selection, Procurement, Distribution, and Use of Pharmaceuticals. Second Edition. Kumarian Press, Incorporated: West Hartford, Connecticut, April 1997.
303. Management Sciences for Health. The Management and Leadership Program. Final Report, 2000-2005. September 29, 2005.
304. Mancey-Jones M, Brugha RF. Using perinatal audit to promote change: a review. Health Policy and Planning, 1997;12(3):183-192.
305. Marek T, O'Farrell C, Yamamoto C, Zable I. Trends and opportunities in public-private partnerships to improve health service delivery in Africa. Africa Region Human Development Working Paper Series 33636, The World Bank, August 2005.

306. Marquez L, Kean L. Making supervision supportive and sustainable: new approaches to old problems. Supplement to Population Reports, MAQ Paper No. 4, 2002.
307. Marquez, L. Helping healthcare providers perform according to standards. QAP Operations Research Issue Paper, September 2001; 2(3). Published for USAID by the Quality Assurance Project. Bethesda, MD: Center for Human Services. available at: http://www.qaproject.org/pubs/PDFs/helphcpro3.pdf, accessed May 4, 2009
308. Marseille E, Hofmann PB, Kahn JG. HIV prevention before HAART in sub-Saharan Africa. Lancet. 2002 25;359(9320):1851-6.
309. Mathauer I, Imhoff I. Health worker motivation in Africa: the role of non-financial incentives and human resource management tools. Hum Resour Health. 2006;4:24.
310. Mathonnat J. Contracting is not an end in itself. Bulletin of the World Health Organization, 2006;84(11): 912-913.
311. Maynard A. Payment for Performance (P4P): International experience and a cautionary proposal for Estonia. Health Financing Policy Paper, Division of Country Health Systems, WHO Regional Office for Europe: Copenhagen. 2008.
312. McNamara P. Provider-specific report cards: a tool for health sector accountability in developing countries. Health Policy Plan. 2006;21(2):101-9.
313. McNamara P. Quality-based payment: six case examples. Int J Qual Health Care. 2005;17(4):357-62.
314. McPake B, Banda EE. Contracting out of health services in developing countries. Health Policy Plan. 1994;9(1):25-30.
315. McPake B. User charges for health services in developing countries: a review of the economic literature. Soc Sci Med. 1993;36(11):1397-405.
316. Merialdi M, Carroli G, Villar J, Abalos E, Gülmezoglu AM, Kulier R, de Onis M. Nutritional interventions during pregnancy for the prevention or treatment of impaired fetal growth: an overview of randomized controlled trials. J Nutr. 2003;133(5 Suppl 2):1626S-1631S.
317. Mierzecki A, et al. [Brief anti-tobacco intervention as a tool for family doctors (general practitioners)] Pneumonol Alergol Pol, 2002;70(3-4):216-22.
318. Miller S, Sloan NL, Winikoff B, Langer A, Fikree FF. Where is the "E" in MCH? The need for an evidence-based approach in safe motherhood. J Midwifery Womens Health. 2003;48(1):10-8.
319. Mills A, Broomberg J. Experiences of contracting: an overview of the literature. Macroeconomics, Health and Development Series, Number 33, World Health Organization, Geneva: 1998.
320. Mills A, Brugha R, Hanson K, McPake B. Approaches for improving service delivery in the nonstate sector: what is the evidence on what works, where and why? Prepared and presented at the "Making Services Work for the Poor" World Development Report 2003-04 Workshop, Eynsham Hall, Oxfordshire, 4-5 November 2002. available at: http://siteresources.worldbank.org/INTWDR2004/Resources/22481_millsetalWDR.pdf, accessed March 19, 2010
321. Mills A, Brugha R, Hanson K, McPake B. What can be done about the private health sector in low-income countries? Bulletin of the World Health Organization, 2002;80(4):325-30.
322. Mills A, Hongoro C, Broomberg J. Improving the efficiency of district hospitals: is contracting an option? Trop Med Int Health. 1997;2(2):116-26.

323.Mills A. To contract or not to contract? Issues for low and middle income countries. Health Policy Plan. 1998;13(1):32-40.
324.Mock C, Arreola-Risa C, Quansah R. Strengthening care for injured persons in less developed countries: A case study of Ghana and Mexico. International Journal of Injury Control and Safety Promotion, 2003;10:1, 45 - 51.
325.Mock C, Joshipura M, Goosen J, Lormand JD, Maier R. Strengthening Trauma Systems Globally: The Essential Trauma Care Project. J Trauma. 2005;59:1243–1246.
326.Mock, C. Improving Prehospital Trauma Care in Rural Areas of Low-Income Countries. J Trauma. 2003;54:1197–1198.
327.Montagu D. Franchising of health services in low-income countries. Health Policy Plan. 2002 Jun;17(2):121-30.
328.Mubbashar MH. Mental health services in rural Pakistan. In: Tansella M, Thornicroft G, eds. Common mental disorders in primary care: essays in honour of Professor Sir David Goldberg. London: Routledge, 1999:67–80.
329.Mugford M, Banfield P, O'Hanlon M. Effects of feedback of information on clinical practice: a review. BMJ. 1991;303(6799):398-402.
330.Murthy RK, Klugman B. Service accountability and community participation in the context of health sector reforms in Asia: implications for sexual and reproductive health services. Health Policy Plan. 2004;19 Suppl 1:i78-i86.
331.Musau S. Community-based health insurance: experiences and lessons learned from east Africa. Technical Report No. 34. Abt Associates, Inc: Bethesda. August 1999.
332.Mwenesi HA. Social science research in malaria prevention, management and control in the last two decades: an overview. Acta Trop. 2005;95(3):292-7.
333.Naimoli JF, Nguyen-Dinh P. Controlling malaria in Francophone Africa: taking the initiative. A series of papers on the ACSI-CCCD Malaria Initiative. Africa child survival initiative-Combatting childhood communicable diseases (ACSI-CCCD). USAID, CDC. Atlanta, GA: 1994.
334.Nanda P. Gender dimensions of user fees: implications for women's utilization of health care. Reprod Health Matters. 2002;10(20):127-34.
335.Nayar KR, Razum O. Health co-operatives: review of international experiences. Croat Med J. 2003;44(5):568-75.
336.Nichter M. Social-science lessons from diarrhea research and their application to ARI. Human Organization, 1993;52(1):53-67.
337.Nyamogoba H, Obala AA. Nosocomial infections in developing countries: cost effective control and prevention. East Afr Med J. 2002;79(8):435-41.
338.Obimbo EM. Primary health care, selective or comprehensive, which way to go? East Afr Med J. 2003;80(1):7-10.
339.O'Brien MA, Rogers S, Jamtvedt G, Oxman AD, Odgaard-Jensen J, Kristoffersen DT, Forsetlund L, Bainbridge D, Freemantle N, Davis DA, Haynes RB, Harvey EL. Educational outreach visits: effects on professional practice and health care outcomes. Cochrane Database Syst Rev. 2007;(4):CD000409. Update of: Cochrane Database Syst Rev. 1997;(4):CD000409.
340.Ofosu-Amaah V. National experience in the use of community health workers. A review of current issues and problems. WHO Offset Publ. 1983;71:1-49.
341.Oh VM. Post-release drug treatment risks: strategies to minimise harm to patients. Ann Acad Med Singapore. 2000;29(5):621-7.

342. Okeke IN, Klugman KP, Bhutta ZA, Duse AG, Jenkins P, O'Brien TF, Pablos-Mendez A, Laxminarayan R. Antimicrobial resistance in developing countries. Part II: strategies for containment. Lancet Infect Dis. 2005;5(9):568-80.
343. Oliveira-Cruz V, Hanson K, Mills A. Approaches to overcoming constraints to effective health service delivery: a review of the evidence. Journal of International Development, 2003;15:41-65.
344. Oliveira-Cruz V, Hanson K, Mills A. Approaches to overcoming health systems constraints at the peripheral level: a review of the evidence. CMH Working Paper Series, Paper No. WG5:15. WHO Commission on Macroeconomics and Health, Working Group 5 - Improving Health Outcomes of the Poor, May 2001.
345. Opiyo N, English M. In-service training for health professionals to improve care of the seriously ill newborn or child in low and middle-income countries (Review). Cochrane Database Syst Rev. 2010;4:CD007071.
346. Oren E, Shaffer ER, Guglielmo BJ. Impact of emerging technologies on medication errors and adverse drug events. Am J Health Syst Pharm. 2003;60(14):1447-58.
347. Ostini R, Hegney D, Jackson C, Williamson M, Mackson JM, Gurman K, Hall W, Tett SE. Systematic review of interventions to improve prescribing. Ann Pharmacother. 2009 Mar;43(3):502-13. Epub 2009 Mar 3.
348. Oxman AD, Thomson MA, Davis DA, Haynes RB. No magic bullets: a systematic review of 102 trials of interventions to improve professional practice. CMAJ. 1995;153(10):1423-31.
349. Paine K, Thorogood M, Wellings K. The impact of the quality of family planning services on safe and effective contraceptive use: a systematic literature review. Hum Fertil (Camb). 2000;3(3):186-193.
350. Palmer N, Mueller DH, Gilson L, Mills A, Haines A. Health financing to promote access in low income settings - How much do we know? Lancet, 2004;364(9442):1365-70.
351. Palmer N. The use of private-sector contracts for primary health care: theory, evidence and lessons for low-income and middle-income countries. Bull World Health Organ. 2000;78(6):821-9.
352. Pappaioanou M, Malison M, Wilkins K, Otto B, Goodman RA, Churchill RE, White M, Thacker SB. Strengthening capacity in developing countries for evidence-based public health: the data for decision-making project. Soc Sci Med. 2003;57(10):1925-37.
353. Parkhurst JO, Penn-Kekana L, Blaauw D, Balabanova D, Danishevski K, Rahman SA, Onama V, Ssengooba F. Health systems factors influencing maternal health services: a four-country comparison. Health Policy, 2005; 73: 127–138.
354. Parlato MB, Favin MN. Primary health care--progress and problems: An analysis of 52 AID-assisted projects. American Public Health Association, Washington, D.C.: 1982, 101 pages.
355. Paskovaty A, Pflomm JM, Myke N, Seo SK. A multidisciplinary approach to antimicrobial stewardship: evolution into the 21st century. Int J Antimicrob Agents. 2005;25(1):1-10.
356. Patouillard E, Goodman CA, Hanson KG, Mills AJ. Can working with the private for-profit sector improve utilization of quality health services by the poor? A systematic review of the literature. International Journal for Equity in Health, 2007;6:17.
357. Paul VK. Innovative programmes of medical education: I. Case studies. Indian J Pediatr, 1993;60(6):759-68.

358.Paul VK. Innovative programmes of medical education: II. Commentary and lessons for India. Indian J Pediatr. 1993;60(6):769-76.
359.Peabody JW, Taguiwalo MM, Robalino DA, Frenk J. Chapter 70. Improving the Quality of Care in Developing Countries. In: Jamison DT, Breman JG, et al, editors. Disease Control Priorities in Developing Countries. Second Edition. The World Bank and Oxford University Press: New York, 2006. p. 1293-1352. available at: http://files.dcp2.org/pdf/DCP/DCP70.pdf, accessed May 24, 2010.
360.Pegurri E, Fox-Rushby JA, Damian W. The effects and costs of expanding the coverage of immunisation services in developing countries: a systematic literature review. Vaccine, 2005;23(13):1624-35.
361.Pelletier DL, Shrimpton R. The role of information in the planning, management and evaluation of community nutrition programmes. Health Policy Plan. 1994;9(2):171-84.
362.Penny S, Murray SF. Training initiatives for essential obstetric care in developing countries: a 'state of the art' review. Health Policy Plan. 2000;15(4):386-93.
363.Perrot J. Different approaches to contracting in health systems. Bull World Health Organ. 2006;84(11):859-66.
364.Perry H, Freeman P, Gupta S, Rassekh BH. Volume 1. Summary Findings. How Effective Is Community-Based Primary Health Care in Improving the Health of Children? A Review of the Evidence. Report to the Expert Review Panel, the World Health Organization, UNICEF, and the World Bank. Community-Based Primary Health Care Working Group, International Health Section, American Public Health Association, 19 March 2008.
365.Perry H, Freeman P, Gupta S, Rassekh BH. Volume II. Description of Methodology, Individual Studies, Reviews, Cross-Cutting Themes and Programmatic Approaches. How Effective Is Community-Based Primary Health Care in Improving the Health of Children? A Review of the Evidence. Report to the Expert Review Panel, the World Health Organization, UNICEF, and the World Bank. Community-Based Primary Health Care Working Group, International Health Section, American Public Health Association, 19 March 2008.
366.Peters DH, Mirchandani GG, Hansen PM. Strategies for engaging the private sector in sexual and reproductive health: how effective are they? Health Policy Plan. 2004;19 Suppl 1:i5-i21.
367.Petersen LA, Woodard LD, Urech T, Daw C, Sookanan S. Does pay-for-performance improve the quality of health care? Ann Intern Med. 2006;145(4):265-72.
368.Piper CJ. Is there a place for traditional midwives in the provision of community-health services? Annals of Tropical Medicine and Parasitology, 1997;91(3):237-45.
369.Prata N, Montagu D, Jefferys E. Private sector, human resources and health franchising in Africa. Bull World Health Organ. 2005;83(4):274-9.
370.Preker AS, et al. Effectiveness of community health financing in meeting the cost of illness. Bulletin of the World Health Organization 2002;80:143-150.
371.Pronovost PJ, Angus DC, Dorman T, Robinson KA, Dremsizov TT, Young TL. Physician staffing patterns and clinical outcomes in critically ill patients: a systematic review. JAMA, 2002;288(17):2151-62.
372.Qingyue, M. Review of provider organization reforms in China. Final draft, October 2005.

373. Radyowijati A, Haak H. Improving antibiotic use in low-income countries: an overview of evidence on determinants. Soc Sci Med. 2003;57(4):733-44.
374. Rall M, Meyer SM. The role of the registered nurse in the marketing of primary healthcare services, as part of health promotion. Curationis. 2006;29(1):10-24.
375. Rasmuson M, Bashir N, Keith N, editors. Community-based approaches to child health: BASICS experience to date. Published for the U.S. Agency for International Development by the Basic Support for Institutionalizing Child Survival (BASICS) Project, Arlington, Va. 1998.
376. Rasmussen Z, Pio A, Enarson P. Case management of childhood pneumonia in developing countries: recent relevant research and current initiatives. Int J Tuberc Lung Dis. 2000;4(9):807-26.
377. Ratanawijitrasin S, Soumerai SB, Weerasuriya K. Do national medicinal drug policies and essential drug programs improve drug use?: a review of experiences in developing countries. Soc Sci Med. 2001;53(7):831-44.
378. Ravert P. An integrative review of computer-based simulation in the education process. Comput Inform Nurs. 2002;20(5):203-8.
379. Rawlings LB, Rubio G. Evaluating the Impact of Conditional Cash Transfer Programs: Lessons from Latin America. The World Bank, Policy Research Working Paper Series, number 3119, 31 Aug 2003. available at: http://www-wds.worldbank.org/external/default/WDSContentServer/IW3P/IB/2003/09/30/000094946_0309160409264/Rendered/PDF/multi0page.pdf, accessed January 19, 2010.
380. Ray AM, Salihu HM. The impact of maternal mortality interventions using traditional birth attendants and village midwives. J Obstet Gynaecol. 2004;24(1):5-11.
381. Reerink IH, Sauerborn R. Quality of primary health care in developing countries: recent experiences and future directions. Int J Qual Health Care. 1996;8(2):131-9.
382. Reeves S, Zwarenstein M, Goldman J, Barr H, Freeth D, Hammick M, Koppel I. Interprofessional education: effects on professional practice and health care outcomes. Cochrane Database of Systematic Reviews 2008, Issue 1. Art. No.: CD002213. DOI: 10.1002/14651858.CD002213.pub2. Update of: Zwarenstein M, Reeves S, Barr H, Hammick M, Koppel I, Atkins J. Interprofessional education: effects on professional practice and health care outcomes. Cochrane Database Syst Rev. 2001;(1):CD002213.
383. Regalía F, et al. Chapter 11: Nicaragua: Combining Demand- and Supply-Side Incentives. p. 215-235. In: Eichler R, Levine R, and the Performance-Based Incentives Working Group. Performance incentives for global health: potential and pitfalls. Versa Press: East Peoria, Illinois. 2009. available at: http://www.cgdev.org/doc/books/PBI/06_CGD_Eichler_Levine-Ch11.pdf; accessed July 7, 2009.
384. Robertson MK, Umble KE, Cervero RM. Impact studies in continuing education for health professions: update. J Contin Educ Health Prof. 2003;23(3):146-56.
385. Robson J. Scaling up health and education workers: systems for training. Literature review. DFID Health Systems Resource Centre: London, March 2005.
386. Rogers B, Goodno L. Evaluation of interventions to prevent needlestick injuries in health care occupations. Am J Prev Med. 2000;18(4 Suppl):90-8.
387. Roll Back Malaria Consultative Meeting on the Role of Medicine Sellers in the Management of Malaria: What’s worked and where do we go from here? Meeting Report, Roll Back Malaria/Malaria Case Management Working Group, 26-27 May 2004,

Accra, Ghana. 2005. London, UK and Arlington, Va., USA: the Malaria Consortium and BASICS for the United States Agency for International Development; prepared for Roll Back Malaria's Sub-group for Communication and Training and Malaria Case Management Working Group.
388. Ross-Degnan D, Laing R, Santoso B, Ofori-Adjei D, Lamoureux C, Hogerzeil H. Improving pharmaceutical use in primary care in developing counties: a critical review of experience and lack of experience. Presented at the International Conference on Improving Use of Medicines, Chiang Mai, Thailand, April 1997.
389. Rowe AK, de Savigny D, Lanata CF, Victora CG. How can we achieve and maintain high-quality performance of health workers in low-resource settings? Lancet. 2005;366(9490):1026-35.
390. Rowe AK, Rowe SY, Holloway KA, Ivanovska V, Muhe L, Lambrechts T. A systematic review of the effectiveness of shortening Integrated Management of Childhood Illness guidelines training. Final Report. October 30, 2008. World Health Organization. available at: http://whqlibdoc.who.int/publications/2008/9789241597210_eng.pdf, accessed May 24, 2010. ***NOTE: Authors are currently revising this report (revised report not yet available as of Sept 15, 2010).***
391. Rudy S, Tabbutt-Henry J, Schaefer L, McQuide P. Improving client-provider interaction. Population Reports, 2003; Fall, Series Q, Number 1. Baltimore, Johns Hopkins Bloomberg School of Public Health, the INFO Project. Available at: http://info.k4health.org/pr/q02/q02.pdf, accessed March 26, 2010.
392. Rusa L, et al. Chapter 10: Rwanda: Performance-Based Financing in the Public Sector. P. 189-214. In: Eichler R, Levine R, and the Performance-Based Incentives Working Group. Performance incentives for global health: potential and pitfalls. Versa Press: East Peoria, Illinois. 2009. available at: http://www.cgdev.org/doc/books/PBI/06_CGD_Eichler_Levine-Ch10.pdf; accessed July 7, 2009.
393. Sack RB, Rodrigues RM, Black RE. Accomplishments in child survival research and programs. Current Issues in Child Survival Series. Arlington, VA: BASICS, 1996. 12 pages.
394. Safe Injection Global Network. Report of the Global Injection Safety and Infection Control Meeting, 14 -16 November 2005, Sofitel Hotel, Hanoi, Viet Nam. World Health Organization: Geneva, 2006.
395. Samba Ndure K, Sy MN, Ntiru M, Diene SM. Best practices and lessons learned for sustainable community nutrition programming. Regional Initiative to Reinforce Capacities in Community Nutrition, ORANA, SANA, USAID, BASICS, 1999.
396. Sancho AT, Kennedy L, Revuelta CC. El voluntariado y la reorientacion de los servicios de salud. Atencion Primaria, 1998:22(7):450-456.
397. Sangani P, Rutherford G, Wilkinson D. Population-based interventions for reducing sexually transmitted infections, including HIV infection. Cochrane Database Syst Rev. 2004;(2):CD001220.
398. Sankaranarayanan R. Health care auxiliaries in the detection and prevention of oral cancer. Oral Oncol. 1997;33(3):149-54.
399. Saraceni V, Leal MC, Hartz ZMA. Avaliação de campanhas de saúde com ênfase na sífilis congênita: uma revisão sistemática. Rev Bras Saude Mater Infant [online], 2005;

5(3): 263-273. ISSN 1519-3829. doi: 10.1590/S1519-38292005000300002. available at: http://www.scielo.br/pdf/rbsmi/v5n3/a02v5n3.pdf, accessed March 24, 2010.
400. Sazawal S, Black RE. Meta-analysis of intervention trials on case-management of pneumonia in community settings. Lancet. 1992;340(8818):528-33.
401. Sazawal S, Black RE; Pneumonia Case Management Trials Group. Effect of pneumonia case management on mortality in neonates, infants, and preschool children: a meta-analysis of community-based trials. Lancet Infect Dis. 2003;3(9):547-56.
402. Schneider H, Blaauw D, Gilson L, Chabikuli N, Goudge, J. Health systems and access to antiretroviral drugs for HIV in southern Africa: Service delivery and human resource challenges. Reprod Health Matters, 2006;14(27):12-23.
403. Scholl TO, Hediger ML, Belsky DH. Prenatal care and maternal health during adolescent pregnancy: a review and meta-analysis. J Adolesc Health. 1994;15(6):444-56.
404. Schopper D, Walley J. Care for AIDS patients in developing countries: a review. AIDS Care. 1992;4(1):89-102.
405. Scrimshaw, NS (editor). Community-Based Longitudinal Nutrition and Health Studies: Classical Examples from Guatemala, Haiti, and Mexico. 1995, International Foundation for Developing Countries (INFDC). Boston, MA. ISBN Number: 0-9635522-6-0. available at: http://www.unu.edu/Unupress/food2/UIN09E/uin09e00.htm, accessed May 24, 2010.
406. Seidel RE. Notes from the field in communication for child survival. Washington, D.C., Academy for Educational Development [AED], Communication and Marketing for Child Survival [HEALTHCOM], 1993 Apr. 246 p.
407. Sengupta PG, Mondal SK, Ghosh S, Gupta DN, Sikder SN, Sircar BK. Review on development and community implementation of oral rehydration therapy. Indian J Public Health. 1994;38(2):50-7.
408. Sepúlveda J, Bustreo F, Tapia R, Rivera J, Lozano R, Oláiz G, Partida V, García-García L, Valdespino JL. Improvement of child survival in Mexico: the diagonal approach. Lancet. 2006;368(9551):2017-27.
409. Sepúlveda J, Bustreo F, Tapia R, Rivera J, Lozano R, Oláiz G, Partida V, García-García L, Valdespino JL. Improvement of child survival in Mexico: the diagonal approach. Lancet. 2006;368(9551):2017-27.
410. Sethi C, Aljunid S, Sulong SB, Zwi AB. Injury care in low- and middle-income countries: identifying potential for change . International Journal of Injury Control and Safety Promotion, 2000;7(3):153-164.
411. Setty V. Organizing work better. Population Reports, 2004; Winter, Series Q, Number 2. Baltimore, Johns Hopkins Bloomberg School of Public Health, The INFO Project. available at: http://info.k4health.org/pr/q02/q02.pdf, accessed March 26, 2010
412. Shah PM. Prevention of mental handicaps in children in primary health care. Bull World Health Organ, 1991;69(6):779-89.
413. Shahmanesh M, Patel V, Mabey D, Cowan F. Effectiveness of interventions for the prevention of HIV and other sexually transmitted infections in female sex workers in resource poor setting: a systematic review. Trop Med Int Health. 2008 May;13(5):659-79.
414. Shaw RP, Griffin C. (editors) Financing Health Care in Sub-Saharan Africa Through User Fees and Insurance. World Bank. Washington DC. 1995. ISBN 0-8213-3075-6.

415. Shea S, DuMouchel W, Bahamonde L. A meta-analysis of 16 randomized controlled trials to evaluate computer-based clinical reminder systems for preventive care in the ambulatory setting. J Am Med Inform Assoc. 1996;3(6):399-409.
416. Shefer A, Santoli J, Wortley P, Evans V, Fasano N, Kohrt A, Fontanesi J, Szilagyi P. Status of quality improvement activities to improve immunization practices and delivery: findings from the immunization quality improvement symposium, October 2003. J Public Health Manag Pract. 2006;12(1):77-89.
417. Sheikh K, Porter J, Kielmann K, Rangan S. Public-private partnerships for equity of access to care for tuberculosis and HIV/AIDS: lessons from Pune, India. Trans R Soc Trop Med Hyg. 2006;100(4):312-20.
418. Shepperd S, Iliffe S. Hospital at home versus in-patient hospital care. Cochrane Database Syst Rev. 2005;(3):CD000356. Review. Update in: Cochrane Database Syst Rev. 2009;(1):CD000356.
419. Shepperd S, McClaran J, Phillips CO, Lannin NA, Clemson LM, McCluskey A, Cameron ID, Barras SL. Discharge planning from hospital to home. Cochrane Database of Systematic Reviews 2010, Issue 1. Art. No.: CD000313. DOI: 10.1002/14651858.CD000313.pub3. Update of: Shepperd S, Parkes J, McClaren J, Phillips C. Discharge planning from hospital to home. Cochrane Database Syst Rev. 2004;(1):CD000313.
420. Shiffman RN, Liaw Y, Brandt CA, Corb GJ. Computer-based guideline implementation systems: a systematic review of functionality and effectiveness. J Am Med Inform Assoc. 1999;6(2):104-14.
421. Shortell SM, Bennett CL, Byck GR. Assessing the impact of continuous quality improvement on clinical practice: what it will take to accelerate progress. Milbank Q. 1998;76(4):593-624, 510.
422. Shortell SM, O'Brien JL, Carman JM, Foster RW, Hughes EF, Boerstler H, O'Connor EJ. Assessing the impact of continuous quality improvement/total quality management: concept versus implementation. Health Serv Res. 1995;30(2):377-401.
423. Sibley L, Sipe TA, Koblinsky M. Does traditional birth attendant training improve referral of women with obstetric complications: a review of the evidence. Social Science and Medicine, 2004;59(8):1757-68.
424. Sibley L, Sipe TA. What can a meta-analysis tell us about traditional birth attendant training and pregnancy outcomes? Midwifery, 2004;20(1):51-60.
425. Sibley LM, Sipe TA, Brown CM, Diallo MM, McNatt K, Habarta N. Traditional birth attendant training for improving health behaviours and pregnancy outcomes. Cochrane Database of Systematic Reviews 2007, Issue 3. Art. No.: CD005460. DOI: 10.1002/14651858.CD005460.pub2.
426. Sibley LM, Sipe TA, Koblinsky M. Does traditional birth attendant training increase use of antenatal care? A review of the evidence. Journal of Midwifery and Women's Health, 2004;49(4):298-305.
427. Siddiqi K, Newell J, Robinson M. Getting evidence into practice: what works in developing countries? International Journal for Quality in Health Care, 2005; 17(5): 447–453.
428. Siddiqi S, Masud TI, Sabri B. Contracting but not without caution: experience with outsourcing of health services in countries of the Eastern Mediterranean Region. Bull World Health Organ. 2006;84(11):867-75.

429. Simmons R, Brown J, Díaz M. Facilitating large-scale transitions to quality of care: an idea whose time has come. Stud Fam Plann. 2002;33(1):61-75.
430. Simmons R, Fajans P, Ghiron L, editors. Scaling up health service delivery: from pilot innovations to policies and programmes. World Health Organization, Geneva: 2007. available at: http://whqlibdoc.who.int/publications/2007/9789241563512_eng.pdf, accessed May 24, 2010.
431. Sirageldin I, Salkever D, Osborn R (editors). Evaluating population programs: international experience with cost-effectiveness analysis and cost-benefit analysis. New York, N.Y., St. Martin's Press, 1983.
432. Smith LA, Jones C, Meek S, Webster J. Review: Provider practice and user behavior interventions to improve prompt and effective treatment of malaria: do we know what works? Am J Trop Med Hyg. 2009;80(3):326-35.
433. Soderlund N, Mendoza-Arana P, Goudge J, editors. The new public/private mix in health: exploring the changing landscape. Alliance for Health Policy and Systems Research, Geneva: 2003. Available at: http://www.who.int/alliance-hpsr/resources/New_Public_Private_Mix_FULL_English.pdf, accessed April 8, 2010.
434. Steinwand RW. IMCI workshop proceedings: Reaching Communities for Child Health: Advancing PVO / NGO Technical Capacity and Leadership for Household and Community Integrated Management of Childhood Illness (HH/C IMCI), Baltimore, Maryland, January 17-19, 2001. Arlington, Virginia, Partnership for Child Health Care, Basic Support for Institutionalizing Child Survival [BASICS], 2001. 196 pages. available at: http://pdf.usaid.gov/pdf_docs/PNACQ721.pdf, accessed Dec 28, 2008
435. Stoeckel J. The intervention research approach to child survival. Asia Pac J Public Health. 1992-1993;6(1):40-5.
436. Stone EG, Morton SC, Hulscher ME, Maglione MA, Roth EA, Grimshaw JM, Mittman BS, Rubenstein LV, Rubenstein LZ, Shekelle PG. Interventions that increase use of adult immunization and cancer screening services: a meta-analysis. Ann Intern Med. 2002;136(9):641-51.
437. Task Force on Health Systems Research. Informed choices for attaining the Millennium Development Goals: towards an international cooperative agenda for health-systems research. Lancet. 2004 Sep 11-17;364(9438):997-1003.
438. Tawfik Y, Northrup R, Prysor-Jones S. Utilizing the Potential of Formal and Informal Private Practitioners in Child Survival: Situation Analysis and Summary of Promising Interventions. February 2002. Support for Analysis and Resarch in Africa (SARA) Project, Academy for Educational Development. available at: http://www.aed.org/Publications/upload/UtilizingthePotential.pdf, accessed Dec 8, 2008.
439. Thomas L, Cullum N, McColl E, Rousseau N, Soutter J, Steen N. Guidelines in professions allied to medicine. Cochrane Database Syst Rev. 2000;(2):CD000349.
440. Thomson O'Brien MA, Oxman AD, Davis DA, Haynes RB, Freemantle N, Harvey EL. Audit and feedback versus alternative strategies: effects on professional practice and health care outcomes. Cochrane Database Syst Rev. 1998;(1):CD000260. Update in: Cochrane Database Syst Rev. 2000;(2):CD000260.
441. Thota AB. Strengthening Health Services – review of evidence [Draft]. 2007
442. Tierney WM. Improving clinical decisions and outcomes with information: a review. Int J Med Inform. 2001;62(1):1-9.

443. Tomasi E, Facchini LA, Maia MDS. Health information technology in primary health care in developing countries: a literature review. Bull World Health Organ, 2004;82(11):867-74.
444. Toonen J, Canavan A, Vergeer P, Elovainio R. Learning lessons in implementing performance based financing, from a multi-country evaluation.Performance based financing. A synthesis report. Royal Tropical Institute of the Netherlands (KIT) in collaboration with Cordaid and WHO. Amsterdam, May 2009. Available at: http://www.who.int/contracting/PBF.pdf, accessed April 2, 2010.
445. Trostle J. Inappropriate distribution of medicines by professionals in developing countries. Soc Sci Med. 1996;42(8):1117-20.
446. Tully MP, Seston EM. Impact of pharmacists providing a prescription review and monitoring service in ambulatory care or community practice. Ann Pharmacother. 2000;34(11):1320-31.
447. Umble KE, Cervero RM. Impact studies in continuing education for health professionals. A critique of the research syntheses. Eval Health Prof. 1996;19(2):148-74.
448. UNAIDS Best Practices Collection Archive. Available at: http://www.unaids.org/Services/publicationsArchive.aspx?id={8C720B29-0440-44C2-8D1A-19B8CEDAD56D}&displaylang=en&page=15, accessed April 2, 2010
449. Unger JP, De Paepe P, Green A. A code of best practice for disease control programmes to avoid damaging health care services in developing countries. Int J Health Plann Manage, 2003;18 Suppl 1:S27-39.
450. Unger JP, Killingsworth JR. Selective primary health care: a critical review of methods and results. Soc Sci Med. 1986;22(10):1001-13.
451. Unger JP, Van Dormael M, Criel B, Van der Vennet J, De Munck P. A plea for an initiative to strengthen family medicine in public health care services of developing countries. Int J Health Serv. 2002;32(4):799-815.
452. University of Leeds, University of York. Hospital volume and health care outcomes, costs and patient access. Effective Health Care, 1996;2(8):1-16.
453. University of York, NHS Centre for Reviews and Dissemination. Review of the Research on the Effectiveness of Health Service Interventions to Reduce Variations in Health. Part 1. CRD Report 3. October 1995. available at: http://www.york.ac.uk/inst/crd/CRD_Reports/crdreport3.pdf, accessed May 19, 2010. (related to: Arblaster L, Lambert M, Entwistle V, Forster M, Fullerton D, Sheldon T, Watt I. A systematic review of the effectiveness of health service interventions aimed at reducing inequalities in health. J Health Serv Res Policy. 1996 Apr;1(2):93-103.)
454. University of York. Getting evidence into health care. Effective Health Care, 1999; 5(1):1-16.
455. Uplekar M, Pathania V, Raviglione M. Private practitioners and public health: weak links in tuberculosis control. Lancet. 2001;358(9285):912-6.
456. Urquhart C, Currell R, Grant MJ, Hardiker NR. Nursing record systems: effects on nursing practice and healthcare outcomes. Cochrane Database Syst Rev. 2009 Jan 21;(1):CD002099. Update of: Currell R, Urquhart C. Nursing record systems: effects on nursing practice and health care outcomes. Cochrane Database Syst Rev. 2003;(3):CD002099.
457. USAID Health Care Improvement Project. The Improvement Collaborative: An Approach to Rapidly Improve Health Care and Scale Up Quality Services. Published by

the USAID Health Care Improvement Project. Bethesda, MD: University Research Co., LLC (URC). 2008.
458. Uxa F, Bacci A, Mangiaterra V, Chiaffoni GP. Essential newborn care training activities: 8 Years of experience in Eastern European, Caucasian and Central Asian countries. Seminars In Fetal & Neonatal Medicine, 2006;11(1):58-64.
459. Van Citters AD, Bartels SJ. A systematic review of the effectiveness of community-based mental health outreach services for older adults. Psychiatr Serv. 2004;55(11):1237-49.
460. van Dam HA, van der Horst F, van den Borne B, Ryckman R, Crebolder H. Provider-patient interaction in diabetes care: effects on patient self-care and outcomes. A systematic review. Patient Educ Couns. 2003;51(1):17-28.
461. Van Wijk BL, Klungel OH, Heerdink ER, de Boer A. Effectiveness of interventions by community pharmacists to improve patient adherence to chronic medication: a systematic review. Ann Pharmacother. 2005;39(2):319-28.
462. Varkevisser CM, Mwaluko GM, Le Grand A. Research in action: the training approach of the Joint Health Systems Research Project for the Southern African Region. Health Policy Plan. 2001;16(3):281-91.
463. Vermeire E, Wens J, Van Royen P, Biot Y, Hearnshaw H, Lindenmeyer A. Interventions for improving adherence to treatment recommendations in people with type 2 diabetes mellitus. Cochrane Database Syst Rev. 2005;(2):CD003638.
464. Victora CG, Habicht JP, Bryce J. Evidence-based public health: moving beyond randomized trials. Am J Public Health, 2004;94(3):400-5.
465. Victora CG, Hanson K, Bryce J, Vaughan JP. Achieving universal coverage with health interventions. Lancet. 2004;364(9444):1541-8.
466. Victora CG, Wagstaff A, Schellenberg JA, Gwatkin D, Claeson M, Habicht JP. Applying an equity lens to child health and mortality: more of the same is not enough. Lancet. 2003;362(9379):233-41.
467. Viswanathan M, Ammerman A, Eng E, Garlehner G, Lohr KN, Griffith D, Rhodes S, Samuel-Hodge C, Maty S, Lux L, Webb L, Sutton SF, Swinson T, Jackman A, Whitener L. Community-based participatory research: assessing the evidence. Evid Rep Technol Assess (Summ). 2004;(99):1-8.
468. Vlassoff C, Fonn S. Health Workers for Change as a health systems management and development tool. Health Policy Plan, 2001;16 Suppl 1: 47-52.
469. Voigt A, Adegoroye A, Kanne J. Continuing education: the rationale for a systems approach. Africa child survival initiative-Combatting childhood communicable diseases (ACSI-CCCD). USAID, CDC. Atlanta, GA: 1993.
470. Volmink J, Garner P. Directly observed therapy for treating tuberculosis. Cochrane Database Syst Rev. 2007;(4):CD003343.
471. Waddell DL. The effects of continuing education on nursing practice: a meta-analysis. J Contin Educ Nurs. 1991;22(3):113-8.
472. Wagstaff A. Poverty and health sector inequalities. Bull World Health Organ. 2002;80(2):97-105.
473. Walker D, Fox-Rushby JA. Economic evaluation of communicable disease interventions in developing countries: a critical review of the published literature. Health Econ. 2000 Dec;9(8):681-98.
474. Walker D. Cost and cost-effectiveness of HIV/AIDS prevention strategies in developing countries: is there an evidence base? Health Policy Plan. 2003;18(1):4-17.

475.Walker DG, Jan S. How do we determine whether community health workers are cost-effective? Some core methodological issues. J Community Health, 2005;30(3):221-9.
476.Wallace, A. A review of the impact of linking health interventions to the Expanded Program on Immunization. Centers for Disease Control and Prevention, National Immunization Program, Strengthening Childhood Immunizations Group, July 2005.
477.Wallerstein N. What is the evidence on effectiveness of empowerment to improve health? Copenhagen,WHO Regional Office for Europe, 2006. (Health Evidence Network report; http://www.euro.who.int/Document/E88086.pdf, accessed September 5, 2009).
478.Walsh JA, Measham AR, Feifer CN, Gertler PJ. The impact of maternal health improvement on perinatal survival: cost-effective alternatives. Int J Health Plann Manage. 1994;9(2):131-49.
479.Walton R, Dovey S, Harvey E, Freemantle N. Computer support for determining drug dose: systematic review and meta-analysis. BMJ. 1999;318(7189):984-90.
480.Walton RT, Harvey E, Dovey S, Freemantle N. Computerised advice on drug dosage to improve prescribing practice. Cochrane Database Syst Rev. 2001;(1):CD002894.
481.Waters H, Hatt L, Peters D. Working with the private sector for child health. Health Policy Plan. 2003;18(2):127-37.
482.Waters HR, Morlock LL, Hatt L. Quality-based purchasing in health care. Int J Health Plann Mgmt 2004; 19: 365–381.
483.Weingarten S. Translating practice guidelines into patient care : guidelines at the bedside. Chest. 2000;118(2 Suppl):4S-7S.
484.Weingarten SR, Henning JM, Badamgarav E, Knight K, Hasselblad V, Gano A Jr, Ofman JJ. Interventions used in disease management programmes for patients with chronic illness-which ones work? Meta-analysis of published reports. BMJ. 2002;325(7370):925.
485.Weinstein R. Family planning training centers for paramedical personnel in the Western hemisphere. Contracept Deliv Syst, 1981;2(1):93-9.
486.Wensing M, Grol R. Single and combined strategies for implementing changes in primary care: a literature review. Int J Qual Health Care. 1994;6(2):115-32.
487.Were W, Mendis K. The Roll Back Malaria strategy for improving access to treatment through home management of malaria. World Health Organization, Geneva: 2005. Document no. WHO/HTM/MAL/2005.1101. http://www.searo.who.int/LinkFiles/Reports_RBM_Strategy.pdf, accessed April 2, 2010
488.Whiting DR, Hayes L, Unwin NC. Challenges to health care for diabetes in Africa. Journal of Cardiovascular Risk, 2003;10(2):103-10.
489.WHO Bibliography available at: www.who.int/entity/contracting/bibliography/Bibliography-Contracting.pdf , accessed May 4, 2009
490.WHO. Chapter Four: Making the Most of Existing Health Workers. In: WHO. The world health report 2006: working together for health. World Health Organization. 2006, pages 89-95.
491.WHO. Interventions and strategies to improve the use of antimicrobials in developing countries: a review. World Health Organization 2001, WHO/CDS/CSR/DRS/2001.9.
492.WHO. The World health report 2000: health systems: improving performance. World Health Organization, Geneva: 2000. available at: http://www.who.int/whr/2000/en/whr00_en.pdf, accessed May 24,. 2010.

493. WHO. WHO Global Strategy for Containment of Antimicrobial Resistance. Geneva: WHO; 2001. WHO document WHO/CDS/CSR/DRS/2001.2. available at: http://whqlibdoc.who.int/hq/2001/WHO_CDS_CSR_DRS_2001.2.pdf, accessed March 29, 2010
494. Wilmoth TA, Elder JP. An assessment of research on breastfeeding promotion strategies in developing countries. Soc Sci Med. 1995;41(4):579-94.
495. Wilton P, Smith R, Coast J, Millar M. Strategies to contain the emergence of antimicrobial resistance: a systematic review of effectiveness and cost-effectiveness. J Health Serv Res Policy, 2002;7(2):111-7.
496. Winch P, LeBan K, Kusha B and Participants at the Workshop "Reaching Communities for Child Health: Advancing PVO/NGO Technical Capacity and Leadership for Household and Community Integrated Management of Childhood Illness (HH/C IMCI)" Baltimore, Maryland, January 17-19, 2001. Reaching Communities for Child Health and Nutrition: A Framework for Household And Community IMCI. The Child Survival Technical Support (CSTS) Project. April 2001. available at: http://pdf.usaid.gov/pdf_docs/PNACL451.pdf, accessed May 25, 2010.
497. Winch PJ, Bhattacharyya K, Debay M, Sarriot EG, Bertoli SA, Morrow RH, and the CORE Monitoring and Evaluation Working Group. Improving the performance of facility- and community-based health workers. The Child Survival Technical Support (CSTS) Project, Calverton, Maryland, USA. December 2003. Available at: http://pdf.usaid.gov/pdf_docs/PNADD769.pdf, accessed April 1, 2010.
498. Winch PJ, Gilroy KE, Wolfheim C, Starbuck ES, Young MW, Walker LD, Black RE. Intervention models for the management of children with signs of pneumonia or malaria by community health workers. Health Policy Plan. 2005;20(4):199-212.
499. Wolf M. Deciding women's lives are worth saving: Expanding the role of midlevel providers in safe abortion care. Chapel Hill, NC, Ipas and IHCAR. 2002.
500. World Bank. Chapter 3: Scaling Up Service Delivery, pages 67-116. In: World Bank. Global Monitoring Report 2005: Millennium Development Goals: From Consensus to Momentum. World Bank: Washington, DC. 2005. ISBN 0-8213-6077-9. available at: http://siteresources.worldbank.org/GLOBALMONITORINGEXT/Resources/complete.pdf, accessed January 19, 2010.
501. Worrall E, Morel C, Yeung S, Borghi J, Webster J, Hill J, Wiseman V, Mills A. The economics of malaria in pregnancy--a review of the evidence and research priorities. Lancet Infect Dis. 2007 Feb;7(2):156-68.
502. Wouters AV. Essential national health research in developing countries: Health-care financing and the quality of care. International Journal of Health Planning and Management, 1991;6(4):253 - 271.
503. Wouters, A. Alternative Provider Payment Methods: Incentives for Improving Health Care Delivery. PHR Primer. 1998.
504. Yeung S, White NJ. How do patients use antimalarial drugs? A review of the evidence. Tropical Medicine and International Health, 2005;10(2):121-38.
505. Yumkella, F. Retention: health workforce issues and response actions in low-resource settings. Capacity Project Resource Paper. The Capacity Project, IntraHealth International, Chapel Hill, NC. August 2005.
506. Zeribi KA, Marquez L. Approaches to healthcare quality regulation in Latin America and the Caribbean: regional experiences and challenges. LACHSR Report No. 63. Published

for the U.S. Agency for International Development (USAID) by the Quality Assurance Project. 2005.
507. Zurn P, Dolea C, Stilwell B. Nurse retention and recruitment: developing a motivated workforce. Issue Paper 4. Department of Human Resources for Health, World Health Organization: Geneva. 2005.
508. Zurovac D, Rowe AK. Quality of treatment for febrile illness among children at outpatient facilities in sub-Saharan Africa. Annals of Tropical Medicine & Parasitology, 2006;100(4): 283–296.
509. Zwarenstein M, Goldman J, Reeves S. Interprofessional collaboration: effects of practice-based interventions on professional practice and healthcare outcomes.Cochrane Database Syst Rev. 2009;(3):CD000072. Update of: Zwarenstein M, Bryant W. Interventions to promote collaboration between nurses and doctors. Cochrane Database Syst Rev. 2000;(2):CD000072.
510. Zygmunt A, Olfson M, Boyer CA, Mechanic D. Interventions to improve medication adherence in schizophrenia. Am J Psychiatry. 2002;159(10):1653-64.

2) We searched document inventories and websites of organizations involved in implementing interventions to improve health care provider performance. Gray literature sources and search details are below:

<u>BASICS</u>

Website: http://www.basics.org/browse.asp?search=Browse
Dates searched: 1/25/2006
Search strategy: hand search all titles

<u>Capacity Project</u>

Website/contact:
http://www.capacityproject.org/index.php?option=com_content&task=view&id=30&Itemid=55; Marc Luoma
Dates searched: 3/28–6/6/2006
1) Hand search of all titles
2) Marc Luoma also provided articles.

<u>CDC</u>

Websites:
Global AIDS Program: http://www.cdc.gov/nchstp/od/gap/resources.html

President's Emergency Plan for AIDS Relief: http://www.pepfar.gov/progress/

Immunizations and Vaccines:
http://www.cdc.gov/vaccines/programs/global/default.htm
http://www.cdc.gov/vaccines/pubs/default.htm

Reproductive Health: http://www.cdc.gov/reproductivehealth/Products&Pubs/SC_Topics.htm

Division of Parasitic Diseases: http://www.cdc.gov/ncidod/dpd/recentpubs/default.htm

Division of Vector-borne Infectious Diseases: http://www.cdc.gov/ncidod/dvbid/pubs/index.htm

Division of Laboratory Systems: http://wwwn.cdc.gov/dls/dlspubs.aspx

Division of Global Public Health Capacity Development:
http://www.cdc.gov/cogh/dgphcd/publications.htm

Sustainable Management Development Program: http://www.cdc.gov/smdp/publications.htm

Date searched: 10/7–10/8/2008
Search strategy: hand search all titles

Center for Global Development

Website: http://www.cgdev.org/section/initiatives/_active/ghprn/workinggroups/performance
Dates searched: 9/7/09
Search strategy: hand search all titles

CORE group

Website: http://www.coregroup.org/document/start.cfm
Dates searched: 3/30/2006
Search strategy: Searched Child Health and Development Database (http://www.coregroup.org/imci/Search.asp?SearchType=normal) using the following sets of terms separately:
a) community health workers; adolescent health
b) community health workers; breastfeeding
c) Monitoring and evaluation; community health workers
d) traditional healers & medicine; training and evaluation
e) TBAs and midwives; training and evaluation
f) Scaling up & lessons learnt; training and evaluation
g) community mobilization
h) Program planning and management
i) Monitoring & Evaluation

DANIDA

Website: http://danida.netboghandel.dk/english/
Dates searched: 1/2006
Search strategy: hand search all titles from “Development Policy” section of Publications webpage; only hard copies were available, received from Finn Schleimann

DFID

Website: http://www.dfidhealthrc.org/publications/index.html
Dates searched: 1/27–6/6/2006
Search strategy: hand search all titles

EngenderHealth

Website: http://www.engenderhealth.org/pubs/
Dates searched: 10/8/2008
Search strategy: hand search all titles

GAVI

Websites:
http://www.gavialliance.org/media_centre/publications/index.php
http://www.gavialliance.org/performance/evaluation/index.php
Dates searched: 10/8/2008
Search strategy: hand search all titles

Global Fund to Fight AIDS, TB, and Malaria

Websites:
http://www.theglobalfund.org/en/media_center/publications/
http://www.theglobalfund.org/en/performance/
http://www.theglobalfund.org/en/links_resources/library/
Dates searched: 10/8/2008
Search strategy: hand search all titles
***Note, as of May 10, 2010:

- The Global Fund general publications website has moved to: http://www.theglobalfund.org/en/publications/
- The Global Fund Evaluation library website has moved to: http://www.theglobalfund.org/en/library/

HealthNet TPO online library
Website: http://healthnettpo.org
Dates searched: 4/8/2010
Search strategy: Search on "health financing" in the title or keywords under the "Research" section of the website

Human Resources for Health Resource Center

Website: http://www.hrhresourcecenter.org/trip_search/advanced/
Dates searched: 7/24/2006
Search strategy: Searched HRH Documents using the following terms separately:
a) staff performance

b) incentives
c) mentoring
d) motivation
e) salary
f) supervision
g) career development

Website: http://www.hrhresourcecenter.org/taxonomy/term/37
Dates searched: 6/30/2008
Search strategy: hand search all titles

<u>International Conference on Social Health Insurance in Developing Countries (Berlin, December 2005)</u>

Website: http://www.who.int/mediacentre/news/releases/2005/pr65/en/index.html
Dates searched: 1/24/2006
Search strategy: Searched all titles and abstracts
***Note, as of May 10, 2010:
A better website for the 2005 International Conference on Social Health Insurance in Developing Countries is: http://www.socialhealthprotection.org/berlin_2005_1.php

<u>ICIUM 1997, 2004 Conference proceedings</u>

Website: http://www.icium.org/index.htm
Dates searched: 7/20/2006
Search strategy: Searched all titles and abstracts

<u>IHI: Institute for Healthcare Improvement</u>

Websites:
http://www.ihi.org/IHI/Results/ImprovementStories/#Developing
http://www.ihi.org/IHI/Results/NewfromIHIPrograms/#DevelopingCountries
http://www.ihi.org/IHI/Results/WhitePapers/
http://www.ihi.org/IHI/Topics/DevelopingCountries/SouthAfrica/Literature/
Dates searched: 10/8/2008
Search strategy: hand search all titles

<u>INRUD: International network for the Rational Use of Drugs</u>

Website: www.inrud-nepal.org.np
Dates searched: 8/3/2006
Search strategy: Searched all titles in "Publications" webpage

INRUD bibliography, as of Februrary 12, 2007
Dates searched: 9/11–9/26/2008
Search strategy: (using Search function in Reference Manager, version 10)

All non-indexed terms containing “developing countries” OR
All non-indexed terms containing “low income countries” OR
All non-indexed terms containing “middle income countries” OR
All non-indexed terms containing “intervention” OR
All non-indexed terms containing “performance” OR
All non-indexed terms containing “Africa” OR
All non-indexed terms containing “Asia” OR
All non-indexed terms containing “Latin America” OR
All non-indexed terms containing “Central America” OR
All non-indexed terms containing “South America” OR
All non-indexed terms containing “Caribbean” OR
All non-indexed terms containing “West Indies” OR
All non-indexed terms containing “Middle East” OR
All non-indexed terms containing “Eastern Europe” OR
All non-indexed terms containing “Hispaniola”

JHPIEGO

Website: http://www.jhpiego.org/resources/pubs/index.asp
Dates searched: 6/6/2006
Search strategy:
1) Hand search all titles shown under “Browse catalog by category”
2) Hand search titles shown under “Browse catalog by topic” in the following topics: Maximizing Access and Quality (MAQ), Performance Improvement, and Training skills

MSH

Contacts: Linda Rawfik, Joan Cunningham, Kindra Willis (kwillis@msh.org)
Dates searched: 3/30–7/6/2006
Search strategy:
1) Four articles were found by Linda Rawfik and Joan Cunningham.
2) Kindra Willis also searched using the following sets of "MSH Institutional Memory Keywords":
="Afghanistan" / ="Bangladesh" / ="Benin" / ="Bhutan" / ="Burkina Faso" / ="Burundi" / ="Cambodia" / ="Cameroon" / ="Central Africa Rep" / ="Chad" / ="Congo" / ="Cote d Ivoire" / ="Eritrea" / ="Ethiopia" / ="Gambia" / ="Ghana" / ="Guinea" / ="Haiti" / ="India" / ="Kenya" / ="Kyrgyzstan" / ="Lao PDR" / ="Lesotho" / ="Madagascar" / ="Malawi" / ="Mali" / ="Mauritania" / ="Moldova" / ="Mozambique" / ="Nepal" / ="Nicaragua" / ="Niger" / ="Nigeria" / ="Pakistan" / ="Rwanda" / ="Sierra Leone" / ="Tajikistan" / ="Tanzania" / ="Togo" / ="Uganda" / ="Uzbekistan" / ="Vietnam" / ="Zambia" / ="Zimbabwe" / ="Albania" / ="Angola" / ="Armenia" / ="Bolivia" / ="Brazil" / ="China" / ="Colombia" / ="Dominican Republic" / ="Ecuador" / ="Egypt" / ="El Salvador" / ="Georgia" / ="Guatemala" / ="Guyana" / ="Honduras" / ="Indonesia" / ="Iraq" / ="Jamaica" / ="Kazakhstan" / ="Morocco" / ="Namibia" / ="Paraguay" / ="Peru" / ="Philippines" / ="Romania" / ="Sri Lanka" / ="Suriname" / ="Swaziland" / ="Syria" / ="Thailand" / ="Tunisia" / ="Turkmenistan" / ="Ukraine" / ="Argentina" / ="Belize" / ="Botswana" / ="Chile" / ="Costa Rica" / ="Dominica" / ="Gabon" /

="Grenada" / ="Hungary" / ="Jamaica" / ="Malaysia" / ="Mauritius" / ="Mexico" / ="Oman" / ="Panama" / ="Poland" / ="Russian Federation" / ="Seychelles" / ="South Africa" / ="St Kitts and Nevis" / ="St Lucia" / ="St Vincent and the Grenadines" / ="Turkey" / ="East Timor" / ="Congo" / ="Suriname"
AND
="training" / ="knowledge attitude practice" / ="planning" / ="job satisfaction" / ="demography" / ="incentives" / ="immunizations" / ="vaccinations" / ="vaccines" / ="strategic planning" / ="decentralization" / ="financing" / ="marketing" / ="franchising" / ="demand" / ="contracting" / ="accreditation" / ="licensing" / ="benchmarking" / ="quality assurance" / ="quality control" / ="quality of health care" / ="reengineering" / ="guidelines" / ="assessment" / ="staffing" / ="physicians" / ="nurses" / ="hospitals" / ="health services" / ="health workers" / ="hospitals" / ="physicians" / ="primary health care" / ="health care costs" / ="health education" / ="quality assurance" / ="training" / ="quality of health care"

="training" / ="knowledge attitude practice" / ="planning" / ="job satisfaction" / ="demography" / ="incentives" / ="immunizations" / ="vaccinations" / ="vaccines" / ="strategic planning" / ="decentralization" / ="financing" / ="marketing" / ="franchising" / ="demand" / ="contracting" / ="accreditation" / ="licensing" / ="benchmarking" / ="quality assurance" / ="quality control" / ="quality of health care" / ="reengineering" / ="guidelines" / ="assessment" / ="staffing" / ="physicians" / ="nurses" / ="hospitals" / ="health services" / ="health workers" / ="hospitals" / ="physicians" / ="primary health care" / ="health care costs" / ="health education" / ="quality assurance" / ="training" / ="quality of health care"
AND
="Gabon" / ="East Timor"
OR
="Africa" / ="Asia" / ="Caribbean" / ="Central Africa" / ="Central America" / ="Central Asia" / ="East Africa" / ="Eastern Asia" / ="Eastern Europe" / ="Latin America" / ="Middle East" / ="Near East" / ="Southeast Asia" / ="Southern Africa" / ="Southern Asia" / ="Southwestern Asia" / ="Sub Saharan Africa" / ="West Africa" / ="South America"
="Afghanistan" / ="Bangladesh" / ="Benin" / ="Bhutan" / ="Burkina Faso" / ="Burundi" / ="Cambodia" / ="Cameroon" / ="Central Africa Rep" / ="Chad" / ="Congo" / ="Cote d Ivoire" / ="Eritrea" / ="Ethiopia" / ="Gambia" / ="Ghana" / ="Guinea" / ="Haiti" / ="India" / ="Kenya" / ="Kyrgyzstan" / ="Lao PDR" / ="Lesotho" / ="Madagascar" / ="Malawi" / ="Mali" / ="Mauritania" / ="Moldova" / ="Mozambique" / ="Nepal" / ="Nicaragua" / ="Niger" / ="Nigeria" / ="Pakistan" / ="Rwanda" / ="Sierra Leone" / ="Tajikistan" / ="Tanzania" / ="Togo" / ="Uganda" / ="Uzbekistan" / ="Vietnam" / ="Zambia" / ="Zimbabwe" / ="Albania" / ="Angola" / ="Armenia" / ="Bolivia" / ="Brazil" / ="China" / ="Colombia" / ="Dominican Republic" / ="Ecuador" / ="Egypt" / ="El Salvador" / ="Georgia" / ="Guatemala" / ="Guyana" / ="Honduras" / ="Indonesia" / ="Iraq" / ="Jamaica" / ="Kazakhstan" / ="Morocco" / ="Namibia" / ="Paraguay" / ="Peru" / ="Philippines" / ="Romania" / ="Sri Lanka" / ="Suriname" / ="Swaziland" / ="Syria" / ="Thailand" / ="Tunisia" / ="Turkmenistan" / ="Ukraine" / ="Argentina" / ="Belize" / ="Botswana" / ="Chile" / ="Costa Rica" / ="Dominica" / ="Gabon" / ="Grenada" / ="Hungary" / ="Jamaica" / ="Malaysia" / ="Mauritius" / ="Mexico" / ="Oman" / ="Panama" / ="Poland" / ="Russian Federation" / ="Seychelles" / ="South Africa" / ="St Kitts and Nevis" / ="St Lucia" / ="St Vincent and the Grenadines" / ="Turkey" / ="East Timor" / ="Congo" / ="Suriname"/ ="Gabon"

OR
="Africa" / ="Asia" / ="Caribbean" / ="Central Africa" / ="Central America" / ="Central Asia" / ="East Africa" / ="Eastern Asia" / ="Eastern Europe" / ="Latin America" / ="Middle East" / ="Near East" / ="Southeast Asia" / ="Southern Africa" / ="Southern Asia" / ="Southwestern Asia" / ="Sub Saharan Africa" / ="West Africa" / ="South America"
AND
="occupational qualifications" / ="deconcentration" / ="partnerships" / ="midwives"

PAHO

Website: http://www.paho.org/Selection.asp?SEL=TP&LNG=ENG
Dates searched: 5/25–8/8/2006
Search strategy:
1) Searched articles in the "Topics List" (http://www.paho.org/Selection.asp?SEL=TP&LNG=ENG) under the following headings:
a) Human resources development:
- Continuing and distance education
- fellowships
- human resource management
- other
- Policies, Planning and regulation of human resources
- Professional and technical education in health sciences
- training in health communication
b) Health systems and services:
- Health care quality assurance
- Health Services Information system
- Health Services Management
- Health Systems Performance Assessment
2) Searched PAHO Library Institutional Memory Database (http://library.paho.org/uhtbin/cgisirsi.exe/Tj5kKlqSNi/300300009/60/50/X) using the following sets of search terms separately:
a) Nursing education
b) Medical education
c) Training
d) Training personnel
e) Financial Incentives
f) Health occupations
g) Physician education
h) Community health workers
i) Health workers
j) Nurse midwives
k) Nurse practitioners
l) Pharmacists
m) traditional health workers
n) traditional birth attendants
o) disease detection

p) quality improvement
q) quality of healthcare
r) career development
s) job satisfaction
t) employee relations
u) working conditions
v) work load

Partners in Health

Website: http://www.pih.org/library/index.html
Dates searched: 8/21/2006
Search strategy: searched titles in the "Books" and "Essays" categories

PHRPlus

Websites: http://www.phrplus.org/pubs_new.html; http://www.dcdata.com/abt/abt.htm
Dates searched: 2/22–3/23/2006
Search strategy:
1) Hand search of titles on http://www.phrplus.org/pubs_new.html.
2) Searched the PHRplus Resource Center Database (http://www.dcdata.com/abt/abt.htm) using the following sets of terms separately:
a) "health & education" / "health personnel"
b) "education" / "evaluation" / "health personnel" / "health worker motivation" / "incentives" / "quality of service"
c) "health personnel" / "evaluation" / "human resource development"
d) "case based payment" / "health worker motivation" / "provider payment mechanisms" / "providers" / "quality of service"
e) "clinical treatment"/"quality of service"/"quality of care"/"health worker motivation

Population Council

Website: http://www.popcouncil.org/frontiers/projects_pubs/topics/qualcare/qoc_lacRI.html (which as of May 10, 2010 is no longer active)
Dates searched: 6/27/2008
Search strategy: hand search all titles

PRIMEII Project

Website: http://www.prime2.org/prime2/section/44.html
Dates searched: 3/28–3/29/2006
Search strategy: hand search all titles

PSSMC

Websites:

http://www.malaria.org/PSSMC/publications.html
http://www.malaria.org/PSSMC/reports.html
Dates searched: 10/8–10/9/2008
Search strategy: hand search all titles

QAP

Website: http://www.qaproject.org/products.html
Dates searched: 1/23–1/24/2006
Search strategy: hand search all titles

Safe Injections Global Network (SIGN)

Website: http://www.who.int/medical_devices/collaborations/network/en/
Dates searched: 2006 (exact month and date not known)
Search strategy: hand search all titles

UNICEF

Website: http://www.unicef.org/publications/index_pubs_subject.html
Dates searched: 10/9/2008
Search strategy: hand search all titles

USAID DEC

Website: http://dec.usaid.gov/
Dates searched: 3/22-3/27/2006; 10/8/2008
Search strategy, 3/22-3/27/2006: Searched Development Experience System (DEXS) (http://dec.usaid.gov/index.cfm?p=search.sqlSearch&CFID=293189&CFTOKEN=99501636) using the following sets of terms separately:
a) On the job training, training allowances, training
b) Occupational Planning
c) Employee Relations
d) Job Satisfaction
e) Personal Development
f) Career Management
g) Mentoring
h) Working conditions
i) Work Load
j) Hours of work
k) overtime work
l) full time work
m) part time work
n) shift work
o) night work
p) leave

q) maternity leave
r) sick leave
s) wages
t) minimum wage
u) employment benefits
v) retirement benefits
w) maternity benefits
x) occupational safety
y) financial incentives
z) incentives
aa) health care costs
bb) alternative health delivery
cc) health delivery

Search strategy, 10/8/2008: Searched DEXS by using "Advanced Search" function and searched for reports with "Combatting Childhood Communicable Diseases" in title only

<u>World Bank</u>

Website: http://www.worldbank.org/reference/
Dates searched: 5/31–6/6/2006
Search strategy: Searched "Documents and Reports" database using the following sets of terms separately:
a) Health systems development and reform
b) financial incentives
c) Health professional education
d) Job satisfaction
e) career management
f) health personnel
g) hours of work
h) wage scale
i) employment benefits
j) quality of health care
k) traditional health workers
l) health worker performance
m) disease treatment
n) preventitive health care
o) health care costs
p) on the job training
q) training health workers

<u>WHO</u>

Websites:
African Programme for Onchocerciasis Control:
http://www.who.int/apoc/publications/reports/en/index.html

Alliance for Health Policy and Systems Research: http://www.who.int/alliance-hpsr/resources/en/

Blood transfusion safety: http://www.who.int/bloodsafety/education_training/en/

Buruli ulcer: http://www.who.int/buruli/information/en/

Child and adolescent health and development: http://www.who.int/child_adolescent_health/documents/atoz/en/index.html

CHOosing Interventions that are Cost Effective: http://www.who.int/choice/en/

Chronic diseases and health promotion: http://www.who.int/chp/knowledge/en/

Clean Care is Safer Care: http://www.who.int/gpsc/journals_articles/en/

Contracting: http://www.who.int/contracting/bibliography/Bibliography-Contracting.pdf

Control of Neglected Tropical Diseases: http://www.who.int/neglected_diseases/resources/en/index.html

Diagnostics and Laboratory Technology: http://www.who.int/diagnostics_laboratory/publications/en/index.html

Dracunculiasis eradication: http://www.who.int/dracunculiasis/resources/en/

Medicines Publications and Documentation: http://www.who.int/medicinedocs/en/cl/CL1.1.1/clmd,20.html

Drug resistance: http://www.who.int/drugresistance/en/

Emergency and essential surgical care: http://www.who.int/surgery/en/

Essential health technologies: http://www.who.int/eht/publications/en/

Essential medicines for children: http://www.who.int/childmedicines/publications/en/

Global Health Workforce Alliance: http://www.who.int/workforcealliance/documents/en/

Health systems: http://www.who.int/healthsystems/publications/en/

Immunization safety: http://www.who.int/immunization_safety/publications/en/

Injection safety: http://www.who.int/injection_safety/en/

IMCI Multi-country Evaluation: http://www.who.int/imci-mce/publications.htm

Knowledge management and health: http://www.who.int/kms/en/

Leishmaniasis: http://www.who.int/leishmaniasis/resources/en/

Leprosy elimination: http://www.who.int/lep/resources/en/

Lymphatic filariasis: http://www.who.int/lymphatic_filariasis/resources/en/

Making pregnancy safer: http://www.who.int/making_pregnancy_safer/documents/en/

Global Malaria Programme: http://www.who.int/malaria/whomalariapublications.htm#1998

Roll Back Malaria Partnership: http://www.rbm.who.int/multimedia/partnershippublications.html

Managers taking Action based on Knowledge and Effective use of resources to achieve Results: http://www.who.int/management/country/en/

Noncommunicable diseases and mental health: http://www.who.int/nmh/publications/en/

Nutrition: http://www.who.int/nutrition/publications/en/

Special Programme for Research and Training in Tropical Diseases: http://www.who.int/tdr/publications/default.htm

Tuberculosis: http://www.who.int/tb/publications/en/

Dates searched: 9/30–10/2/2008
Search strategy: hand search all titles

3) We collaborated with WHO/CAH to obtain unpublished reports on the Integrated Management of Childhood Illness (IMCI) strategy

4) We contacted the following colleagues and asked for references and unpublished studies: John Chalker, Robert Chen, Eveline Klinkenberg, Stephen Luby, Elizabeth Molyneux, Joe Naimoli, Dennis Ross-Degnan, Alexander Rowe, Samantha Rowe, Robert Scherpbier, Paul Shekelle, Phyllida Travis, Anita Wagner, Peter Winch. The following authors of studies included in our review also contributed papers: Tania Barham, Patricia Haggerty, Theresa Hatzell Hoke, Therese McGinn, and Paul Winters.

A.4. Screening of search results

Alex, Sam, Earl, Gabriel, Bhavya, Chunying, Shannon, and Tashana screened the search results to identify potentially relevant studies. Concordance testing was conducted against a “gold

standard" list of articles until the screeners were each able to identify at least 80% of "gold standard" articles. Sam, Bhavya, Chunying, Shannon, and Tashana obtained hard copies of potentially relevant studies. Alex, Sam, Earl, Gabriel, Bhavya, Chunying, Shannon, and Tashana reviewed the potentially relevant studies to identify those that met the inclusion criteria of the literature review. Sam double-checked the studies that were identified to assure that they should be included.

Schematic of screening process.

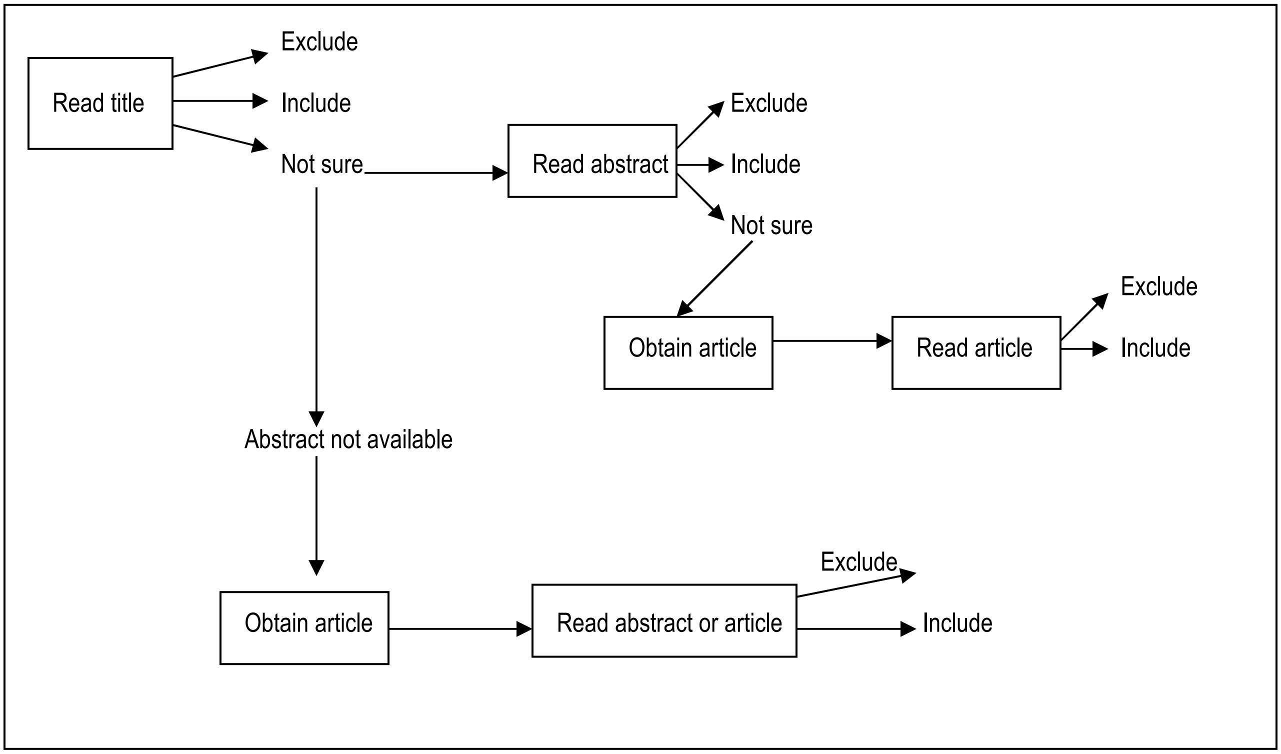

ANNEX 3. Data abstraction form

A. Final version of key documents.

- The data abstraction form: HCPP_Database(abstraction form)v22.doc (Annex 7)
- The handbook with details of how to abstract data: HCPP Database Handbook_v19.doc (Annex 8)
- The description of the data abstraction process: Data_Abstraction_Processv2.doc (Annex 9)
- The code book with names of variables in the database: HCPPDatabase_v6(Access codebook).doc (Annex 10)

B. The following summarizes some key sections of the abstraction form.

- See data abstraction form from Kathy Holloway's database
- Country, year of study, public vs. private setting, funding source of study, indicator of wealth for study area, did intervention have local "support" (e.g., did local HCP and/or community accept the intervention)
- Cost of interventions
- Quality criteria for randomized, controlled trials (Table 1, page 12 of Grimshaw, 2004)
    - Concealment of allocation
    - Protection against contamination
    - Blinded assessment of outcome
    - Reliable outcome measure
    - Outcomes measured at baseline, and no differences were found
    - Follow-up of professionals
    - Follow-up of patients
- Quality criteria for controlled before-and-after studies (Table 2, page 13 of Grimshaw, 2004)
    - Characteristics of study and control reported and similar
    - Protection against contamination
    - Blinded assessment of outcome
    - Reliable outcome measure
    - Outcomes measured at baseline, and no differences were found
    - Follow-up of professionals
    - Follow-up of patients
- Quality criteria for interrupted time series studies (Table 2, page 13 of Grimshaw, 2004)
    - The intervention is independent of other changes
    - The intervention is unlikely to affect data collection
    - Blinded assessment of outcome
    - Reliable outcome measure
    - Completeness of data set
    - Analyzed appropriately
- Quality criteria used to assess the quality of economic evaluations and cost analyses (Box 3, page 44 of Grimshaw, 2004)
    - Research question stated
    - Importance of question stated
    - Viewpoint of analysis stated and defined
    - Rationale for choosing alternative programmes or interventions compared stated

- Alternatives being compared clearly defined
- Form of economic evaluation used stated
- Choice of form of economic evaluation justified in relation to question addressed
- Source(s) of effectiveness estimates stated
- Details of design and results of effectiveness study given
- Details of methods of synthesis of estimates of health effects given
- Primary outcome measure(s) for economic evaluation clearly stated
- Methods to value health states and other benefits stated
- Details of subjects from whom valuations were obtained given
- Productivity changes (if included) reported separately
- Relevance of productivity changes to study question discussed
- Quantities of resources reported separately from their unit costs
- Methods for estimation of quantities and unit costs described
- Currency and price data recorded
- Details of currency and price adjustments for inflation or currency conversion given
- Details of any model used
- Choice of model used and key parameters on which it is based justified
- Time horizon of costs and benefits stated
- Discount rate(s) stated
- Choice of rate(s) justified
- Explanation given if costs and benefits are not discounted
- Details of statistical tests and confidence interval given for stochastic data
- Approach to sensitivity analysis given
- Choice of variable for sensitivity analysis justified
- Ranges of which variables are varied stated
- Relevant alternatives compared
- Incremental analysis reported
- Major outcomes presented in an aggregated as well as a disaggregated form
- Answer to study question given
- Conclusions follow from data reported
- Conclusions accompanied by appropriate caveats

ANNEX 4. Instrument for collecting details from study investigators on contextual factors and other factors that influenced intervention effectiveness

A. Factors that may influence HCP practices

- HCP factors: knowledge (especially of guidelines), skills, motivation and job satisfaction, remuneration, experience (outcomes of past patients), fear of a bad clinical outcome, attitudes towards the guidelines (perceived self-efficacy, or a HCP's confidence that he or she can implement the guidelines; belief that the guidelines are effective), professional values (including attitudes towards corruption), personal goals (including profit motives), perceptions of patient demands and fear that unsatisfied patients will go to another HCP, comprehension of work responsibilities, socio-demographic factors, and the HCP's personal health status
- Patient or client factors: illness severity, patient demands, and patient socio-demographic factors (e.g., age, sex, education, wealth, race, and ethnicity)
- Attributes of the "work": complexity and clarity of guidelines, health topic addressed by guidelines (acute vs. chronic care), and changes in guidelines over time
- Practice environment: practices & attitudes of co-workers, peer pressure, leadership of the director, supervision, presence of quality improvement processes, patient caseload, supplies and equipment, communication (e.g., a mobile phone), facility type (public vs. private, small clinic vs. hospital), location (urban vs. remote village), organization (patient flow, HW deployment), and HCP participation in planning and organizing work
- Professional environment: colleagues, professional associations, and certifying bodies
- Educational environment: formal and informal educational opportunities
- Administrative environment: rules governing HCP behaviors and working conditions, salary levels and regularity of payment, non-financial incentives, job security, leadership of administrative chiefs, presence of quality improvement processes, supervision of supervisors, availability of information, and decentralization (degree to which local health authorities have ownership of the planning and implementation process)
- Employment environment: employment opportunities, which may lead to absenteeism (HCPs leave a public health post to work in a private clinic)
- Commercial environment: promotion of drugs by pharmaceutical companies
- Community environment: how the HCP is perceived by the local community and media
- Socio-cultural environment: traditions and values of society-at-large
- Economic environment: economic conditions of the country and health system
- Political environment: ideologies, political structures, and corruption

B. Other information

- Country, year of study, public vs. private setting, funding source of study, indicator of wealth for study area, did intervention have local "support" (e.g., did local HCP and/or community accept the intervention)
- Cost of intervention

ANNEX 5. Authorship Guidelines: Faculty of Medicine, Harvard University

[available at: http://www.hms.harvard.edu/fa/guide_doc.html, accessed October 10, 2008]

AUTHORSHIP

1. Everyone who is listed as an author should have made a substantial, direct, intellectual contribution to the work. For example (in the case of a research report) they should have contributed to the conception, design, analysis and/or interpretation of data. Honorary or guest authorship is not acceptable. Acquisition of funding and provision of technical services, patients, or materials, while they may be essential to the work, are not in themselves sufficient contributions to justify authorship.

2. Everyone who has made substantial intellectual contributions to the work should be an author. Everyone who has made other substantial contributions should be acknowledged.

3. When research is done by teams whose members are highly specialized, individual's contributions and responsibility may be limited to specific aspects of the work.

4. All authors should participate in writing the manuscript by reviewing drafts and approving the final version.

5. One author should take primary responsibility for the work as a whole even if he or she does not have an in-depth understanding of every part of the work.

6. This primary author should assure that all authors meet basic standards for authorship and should prepare a concise, written description of their contributions to the work, which has been approved by all authors. This record should remain with the sponsoring department.

ORDER OF AUTHORSHIP

Many different ways of determining order of authorship exist across disciplines, research groups, and countries. Examples of authorship policies include descending order of contribution, placing the person who took the lead in writing the manuscript or doing the research first and the most experienced contributor last, and alphabetical or random order. While the significance of a particular order may be understood in a given setting, order of authorship has no generally agreed upon meaning.

As a result, it is not possible to interpret from order of authorship the respective contributions of individual authors. Promotion committees, granting agencies, readers, and others who seek to understand how individual authors have contributed to the work should not read into order of authorship their own meaning, which may not be shared by the authors themselves.

1. The authors should decide the order of authorship together.

2. Authors should specify in their manuscript a description of the contributions of each author and how they have assigned the order in which they are listed so that readers can interpret their roles correctly.

3. The primary author should prepare a concise, written description of how order of authorship was decided.

IMPLEMENTATION

1. Research teams should discuss authorship issues frankly early in the course of their work together.

2. Disputes over authorship are best settled at the local level by the authors themselves or the laboratory chief. If local efforts fail, the Faculty of Medicine can assist in resolving grievances through its Ombuds Office.

3. Laboratories, departments, educational programs, and other organizations sponsoring scholarly work should post, and also include in their procedure manuals, both this statement and a description of their own customary ways of deciding who should be an author and the order in which they are listed. They should include authorship policies in their orientation of new members.

4. Authorship should be a component of the research ethics course that is required for all research fellows at Harvard Medical School.

5. These policies should be reviewed periodically because both scientific investigation and authorship practices are changing.

ANNEX 6. Analysis of interrupted time series studies

These studies have two potential measures: level changes (which indicate immediate effects of interventions) and slope change (which indicate longer-term effects). See figure below.

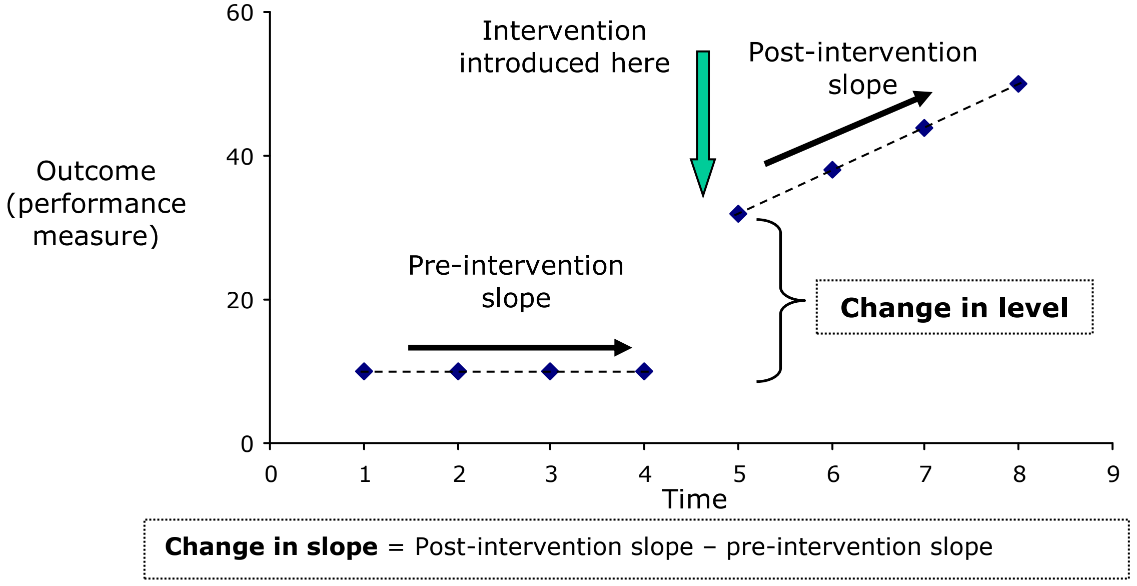

If time-point-specific outcome measures are provided, level and slope changes can be calculated with a piecewise regression model. Two data elements are required for “unadjusted” piecewise regression analysis: the mean outcome measure at each time point, and the time since the intervention began when the outcome was measured. (Time = 0 when the intervention began.)

The general mathematical model for “unadjusted” piecewise regression is below.

$Y = B_0 + B_1(G) + B_2(T) + B_3(T)(G) + e$

where
Y = outcome (performance measure)
T = time relative to when intervention began
G = 1 if $T \geq 0$, 0 if $T < 0$ (G allows intercept and slope to change at time of intervention)
B_0 = regression coefficient for baseline intercept
B_1 = regression coefficient for change in baseline intercept just after intervention began
B_2 = regression coefficient for pre-intervention slope (change in mean outcome per month during period before intervention)
B_3 = regression coefficient for change in slope after intervention began
e = residual error
$(B_0 + B_1)$ = baseline mean outcome level just after intervention began (when T = 0)
$(B_2 + B_3)$ = post-intervention slope (change in mean outcome per month during period after intervention began [when $T \geq 0$])

Regression coefficients can be estimated by using a statistical software package, such as SAS.

ANNEX 7. Data abstraction form

ANNEX 8. Data abstraction handbook

ANNEX 9. The description of the data abstraction process

ANNEX 10. Database code book
