# Supplementary material for: A systematic review of the effectiveness of strategies to improve health care provider performance in low- and middle-income countries: Methods and descriptive results
Source: PLoS One. 2019 May 31;14(5):e0217617. doi: 10.1371/journal.pone.0217617 (PMC6544255; doi:10.1371/journal.pone.0217617)
Supplement: S3 File — (PDF) [file pone.0217617.s003.pdf]

# Appendix 3. Detailed methods for assessing risk of bias and calculating effect sizes, and notes on coding outcome characteristics and strategies and analysis

## Assessing risk of bias

This method, which was designed to be an automated approach for assessing risk of bias (ROB) at the study level for use by the Health Care Provider Performance Review (HCPPR), was based on guidance from the Cochrane Effective Practice and Organisation of Care (EPOC) Group and discussions and emails with Andy Oxman and Simon Lewin in April–May 2014.

The final ROB categories are: very high ROB, high ROB, moderate ROB, and low ROB.

Note about handling ROB criteria that are unclear. For each ROB criterion that is unclear, downgrade by 0.5 ROB levels. The final number of ROB downgrades ignores fractions. Thus, it takes two unclear ROB criteria for a ROB downgrade of 1 level.

Note about studies with mixed study designs (e.g., a given study has some outcomes with an "interrupted time series (ITS) with non-randomized controls" design and some outcomes with a "pre-post with non-randomized controls" design).

- To determine the final study-level ROB category, choose the category with the highest ROB. E.g., in the example above (assuming >1 cluster per study arm), the outcomes with an "ITS with non-randomized controls" design would have a ROB category of "moderate", and the outcomes with an "pre-post with non-randomized controls" design would have a ROB category of "high". Thus, the final overall ROB category for the entire study would be "high".
- If a study with a mixed design has outcomes of different general outcome categories (e.g., process outcomes expressed as a percentage [POPs] and health impact outcomes) and one is doing an analysis of only one general outcome category (e.g., only POPs), then the above process would only apply to the POPs. E.g., in the above example (assuming >1 cluster per study arm), if all the POPs had "ITS with non-randomized controls" design, then for a POPs-only analysis, the final overall ROB category for the entire study would be "moderate".

*For randomized controlled trials (RCTs), excluding controlled interrupted time series (ITS) studies*

- Automatically give initial code of low ROB
- If 1 cluster per study arm, then automatically code as high ROB
- If 2–3 clusters per study arm, then downgrade by 1 ROB level
- If 4–5 clusters per study arm, then downgrade by 0.5 ROB level
- If incomplete dataset, then downgrade by 1 ROB level
- If completeness of dataset is unclear, then downgrade by 0.5 ROB level
- If imbalance in baseline outcome measurements, then downgrade by 1 ROB level
- If similarity in baseline outcome measurements is unclear, then downgrade by 0.5 ROB level
- If no baseline outcome measurements (i.e., post-only RCTs), then check for imbalance in baseline characteristics.
    - If there's an imbalance, then downgrade by 1 ROB level.
    - If it was unclear where there was an imbalance, then downgrade by 0.5 ROB level.
- If outcome was not reliable, then downgrade by 1 ROB level. Note that "not reliable" means something like self-reported health worker practices.

- If outcome reliability was unclear, then downgrade by 0.5 ROB level.
- For studies randomized at the individual level (note that there were only 15 of these of 491 studies), then check for adequacy of concealment of allocation. (Note: We assumed concealment was adequate if allocation was done at the district (or some similar higher level) only, health facility only, or village only.)
  - If inadequate concealment of allocation, then downgrade by 1 ROB level.
  - If adequacy of concealment of allocation was unclear, then downgrade by 0.5 ROB level.

*For non-randomized studies with controls (excluding controlled ITS studies)*
- Automatically give initial code of high ROB
- If 1 cluster per study arm, then downgrade to very high ROB
- If incomplete dataset, then downgrade 1 ROB level
- If completeness of dataset is unclear, then downgrade by 0.5 ROB level
- If intervention likely to affect data collection, then downgrade 1 ROB level
- If it is unclear whether the intervention was likely to have affected data collection, then downgrade 0.5 ROB level.
- If outcome was not reliable, then downgrade by 1 ROB level. Note that "not reliable" means something like self-reported health worker practices.
- If outcome reliability was unclear, then downgrade by 0.5 ROB level.

*For ITS without controls*
- Automatically give initial code of moderate ROB
- If intervention not independent of other changes, then downgrade by 1 ROB level
- If it was unclear whether the intervention was independent of other changes, then downgrade by 0.5 ROB level.
- If <6 data points before or <6 data points after the intervention, then downgrade by 1 ROB level
- If intervention likely to affect data collection, then downgrade 1 ROB level
- If it is unclear whether the intervention was likely to have affected data collection, then downgrade 0.5 ROB level.
- If incomplete dataset, then downgrade 1 ROB level
- If completeness of dataset is unclear, then downgrade by 0.5 ROB level
- If outcome was not reliable, then downgrade by 1 ROB level. Note that "not reliable" means something like self-reported health worker practices.
- If outcome reliability was unclear, then downgrade by 0.5 ROB level.

*For ITS with non-randomized controls*
- Automatically give initial code of moderate ROB
- If control group has only 1 cluster, then reanalyze as ITS without controls (and apply ROB algorithm shown above).
- If <6 data points before or <6 data points after the intervention, then downgrade by 1 ROB level
- If intervention likely to affect data collection, then downgrade 1 ROB level
- If it is unclear whether the intervention was likely to have affected data collection, then downgrade 0.5 ROB level.
- If incomplete dataset, then downgrade 1 ROB level
- If completeness of dataset is unclear, then downgrade by 0.5 ROB level

- If outcome was not reliable, then downgrade by 1 ROB level. Note that "not reliable" means something like self-reported health worker practices.
- If outcome reliability was unclear, then downgrade by 0.5 ROB level.

*For ITS with randomized controls*

- Automatically give initial code of low ROB
- If control group has only 1 cluster, then reanalyze as ITS without controls (and apply ROB algorithm shown above).
- If <6 data points before or < 6 data points after the intervention, then downgrade by 1 ROB level
- If intervention likely to affect data collection, then downgrade 1 ROB level
- If it is unclear whether the intervention was likely to have affected data collection, then downgrade 0.5 ROB level.
- If incomplete dataset, then downgrade 1 ROB level
- If completeness of dataset is unclear, then downgrade by 0.5 ROB level.
- If outcome was not reliable, then downgrade by 1 ROB level. Note that "not reliable" means something like self-reported health worker practices.
- If outcome reliability was unclear, then downgrade by 0.5 ROB level.

*Limitations*

1. ROB assessment for non-randomized studies might be overly conservative for studies that were well done and had many clusters.
2. ROB assessment for randomized studies might be overly generous for studies with only a few clusters per arm.

*Other EPOC criteria not used*

1. The "blinding of outcome assessment" criterion was not used because it was assumed that a lack of blinding might only introduce bias if data collectors were prejudiced
2. The "baseline characteristics similar" criterion was not used because the HCPPR assessed this for all baseline characteristics, and an imbalance would only introduce bias if the imbalance was for factors that would have been effect modifiers. At present, it wouldn't be feasible to go back to the studies to determine this.
3. The "contamination" criterion was not used because contamination would lead to an underestimation of effects.

## Calculating effect sizes

*Overview*

The primary outcome measure was the effect size. The formula for an effect size depended on whether the study design was ITS or non-ITS, and whether the outcome was dichotomous or continuous [Ross-Degnan et al., 1997]. For non-ITS studies in which the outcome was dichotomous (e.g., patient treated correctly: yes/no) or a percentage (e.g., for each patient, the percentage of needed tasks that were done), effect sizes were calculated using Equation 1. These effect sizes were based on the baseline value closest in time to the beginning of the strategy and the follow-up value furthest in time from the beginning of the strategy.

Equation 1: Effect size = $(\text{follow-up} - \text{baseline})_{\text{intervention}} - (\text{follow-up} - \text{baseline})_{\text{control}}$

If the outcome was continuous, but obviously bounded (e.g., knowledge score from 0 to 50) and summarized as a mean, it was converted to a percentage, which equaled the outcome measure minus the lowest possible outcome value divided by the difference between the maximum and minimum values and then multiplied by 100%. For example, if a performance index varied from –10 to 10, then the percentage = ([index – {–10}]/[10– {–10}]) x 100%, or ([index + 10]/20) x 100%. Then, Equation 1 was used to calculate the effect size.

For non-ITS studies in which the outcome was continuous, but not obviously bounded (e.g., number of drugs prescribed per patient) and summarized as a mean, Equation 2 was used. As with the effect sizes for dichotomous/percentage outcomes (described above), the effect sizes for continuous outcomes represent an absolute percentage-point (%-point) change, and they were based on the baseline value closest in time to the beginning of the strategy and the follow-up value furthest in time from the beginning of the strategy.

Equation 2: Effect size = $100\% \times \left( \left[ \frac{\textit{follow-up} - \textit{baseline}}{\textit{baseline}} \right]_{\text{interventi on}} - \left[ \frac{\textit{follow-up} - \textit{baseline}}{\textit{baseline}} \right]_{\text{control}} \right)$

For ITS studies, segmented linear regression modeling was performed to estimate three effect sizes: 1) the change in the level of the outcome immediately after the intervention, 2) the change in the trend of the outcome after the intervention (post-intervention slope minus pre-intervention slope), and 3) a summary effect size that incorporated both the level and trend effects. The summary effect size was the outcome level at the mid-point of the follow-up period as predicted by the regression model minus a predicted counterfactual value that equaled the outcome level based on the pre-intervention trend extended to the mid-point of the follow-up period. See below for details. The summary effect size is used in all analyses in this report because it allowed the results of ITS studies to be combined with non-ITS studies (i.e., effect sizes based on changes in level and slope are not applicable to non-ITS studies).

*Details about effect sizes*

A. Definition of effect size for dichotomous outcomes
   1. Effect size = $(\%\text{POST} - \%\text{PRE})_{\text{intervention}} - (\%\text{POST} - \%\text{PRE})_{\text{control}}$
   2. If there is no reference study group, but there is a pre- and post-intervention measurement, effect size = $(\%\text{POST} - \%\text{PRE})_{\text{intervention}}$

3. If there are no baseline measurements, but there is a reference group, effect size = $(\%POST)_{intervention} - (\%POST)_{reference}$

B. Definition of effect size for outcomes that are a percentage for each patient or subject

1. Summarize multiple observations as a mean percentage. Effect size = $(\%POST - \%PRE)_{intervention} - (\%POST - \%PRE)_{reference}$
2. If there is no reference study group, but there is a pre- and post-intervention measurement, effect size = $(\%POST - \%PRE)_{intervention}$
3. If there are no baseline measurements, but there is a reference group, effect size = $(\%POST)_{intervention} - (\%POST)_{reference}$

C. Definition of effect size for outcomes that are continuous, but <u>obviously</u> bounded (e.g., knowledge score from 0 to 50) and summarized as a mean score

1. Convert the mean score to a percentage by dividing the mean score by the maximum possible score. Effect size = $(\%POST - \%PRE)_{intervention} - (\%POST - \%PRE)_{reference}$
2. If there is no reference study group, but there is a pre- and post-intervention measurement, then convert the mean score to a percentage by dividing the mean score by the maximum possible score. Effect size = $(\%POST - \%PRE)_{intervention}$
3. If there are no baseline measurements, but there is a reference group, then convert the mean score to a percentage by dividing the mean score by the maximum possible score. Effect size = $(\%POST)_{intervention} - (\%POST)_{reference}$
4. Note 1. The maximum possible score must be the same for all subjects.
5. Note 2. If minimum possible score is not zero, then shift mean scores so minimum = zero (e.g., if score ranges from -10 to +10, then add 10 to all results, so the ranges is shifted to 0 to +20).

D. Definition of effect size for outcomes that are continuous, but not obviously bounded (e.g., mortality rate)

1. Effect size = $([POST - PRE] / PRE)_{intervention} - ([POST - PRE] / PRE)_{control}$
2. If there is no reference study group, but there is a pre- and post-intervention measurement, effect size = $([POST - PRE] / PRE)_{intervention}$
3. If there are no baseline measurements, but there is a reference group, effect size = $(POST_{intervention} - POST_{control}) / POST_{control}$
4. In a <u>non-ITS two-arm study</u>, effect size = 100% * {([POST – PRE] / PRE)intervention – ([POST – PRE] / PRE)reference}, which is a difference of baseline-to-follow-up differences relative to each arm's baseline and is on a percentage-point scale. Using the figure below,

Non-ITS effect size = 100% * {[(X – W)/W] – [(Z – Y)/Y]}

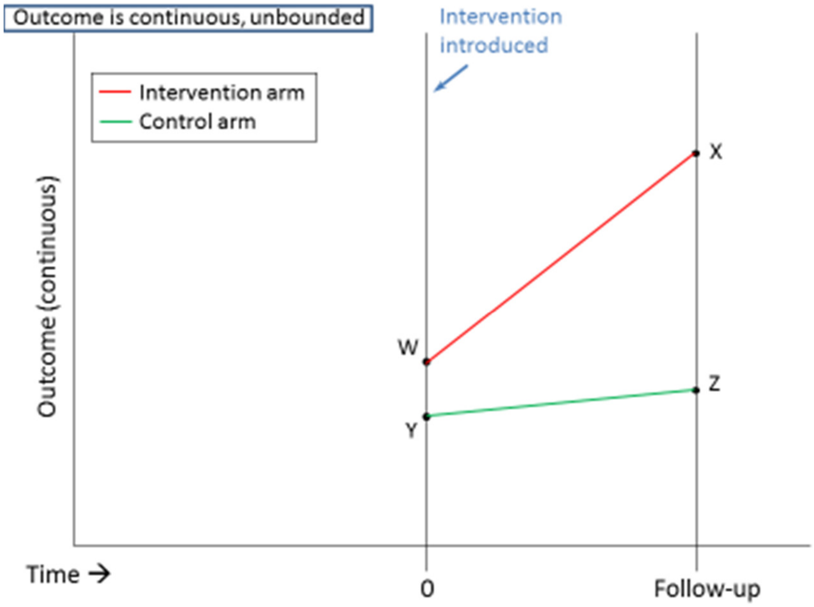


E. Interrupted time series studies had three potential measures: 1) level changes (which indicate immediate effects of interventions), 2) slope change (which indicate longer-term effects), and 3) a summary effect size that incorporated both the level and slope effects. The summary effect size was the outcome level at the mid-point of the follow-up period as predicted by the regression model (point 'A', below) minus a predicted counterfactual value that equaled the outcome level based on the pre-intervention trend extended to the mid-point of the follow-up period (point 'B' below). See figure below.

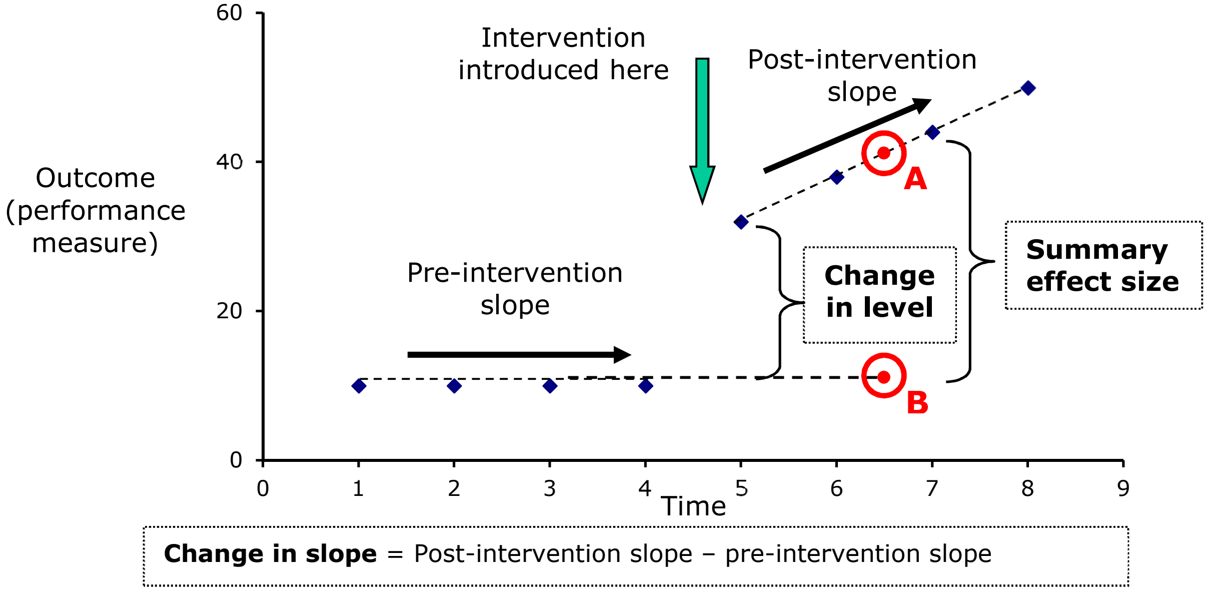


If time-point-specific outcome measures are provided, level and slope changes can be calculated with a segmented linear regression model. Two data elements are required for "unadjusted" segmented linear regression analysis: the mean outcome measure at each time point, and the time since the intervention began when the outcome was measured. (Time = 0 when the intervention began.)

The general mathematical model for "unadjusted" segmented linear regression is below.

$Y = B_0 + B_1(G) + B_2(T) + B_3(T)(G) + \varepsilon$

where
Y = outcome (performance measure)
T = time relative to when intervention began
G = 1 if $T \geq 0$, 0 if $T < 0$ (G allows intercept and slope to change at time of intervention)
$B_0$ = regression coefficient for baseline intercept
$B_1$ = regression coefficient for change in baseline intercept just after intervention began
$B_2$ = regression coefficient for pre-intervention slope (change in mean outcome per month during period before intervention)
$B_3$ = regression coefficient for change in slope after intervention began
$\varepsilon$ = residual error
$(B_0 + B_1)$ = baseline mean outcome level just after intervention began (when T = 0)
$(B_2 + B_3)$ = post-intervention slope (change in mean outcome per month during period after intervention began [when $T \geq 0$])

In all models, autoregression (AR) correlation terms were tested and retained in the final model if statistically significant at the $p < 0.05$ level. We tested AR1 terms in all models, and we also tested AR12 terms if there was a cyclical pattern to the time trend (e.g., 12-month cycles corresponding to seasonality).

<u>For ITS studies with two groups and an outcome expressed as a percentage</u>, we performed segmented regression analysis on a data series in which each time point was the difference between the two original study groups (e.g., intervention group data point minus control group data point).

<u>For ITS studies with two groups and a continuous outcome not expressed as a percentage</u>, we performed segmented regression analysis separately for each study group and calculated a summary effect size for each study group. The final effect size equaled the difference between the two summary effect sizes (e.g., intervention group summary effect size minus control group summary effect size, see figure below). We used each study group's regression model to derive for each group: 1) the estimated value just before intervention was introduced (e.g., point A for the intervention arm), 2) the estimated value based on the baseline trend of the model extended to midpoint of follow-up (e.g., point D for the intervention arm), and 3) the estimated value at midpoint of follow-up (e.g., point C for the intervention arm). Then we used these values to calculate the summary ITS effect size. Using the figure below,

ITS summary effect size = $100\% * \{[(C – A)/A – (D – A)/A] – [(E – B)/B – (F – B)/B]\}$, which simplifies to

$$100\% * [(C – D)/A – (E – F)/B]$$

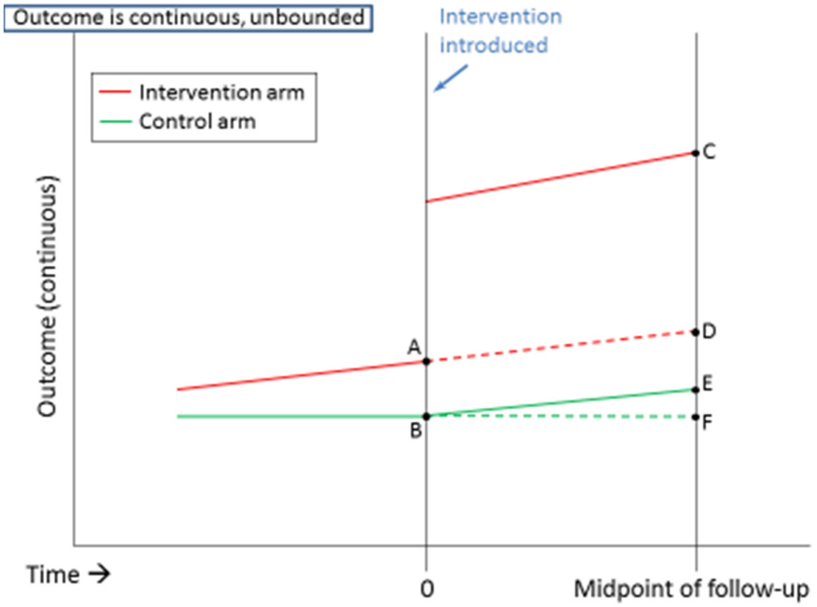


*Other notes about effect size calculations*

If outcomes expressed as a percentage had measures all <15% and could not plausibly reach 100%, we analyzed these outcomes as continuous, rather than as a percentage.

If outcomes reported as a percentage were actually ratios (numerator was not subset of denominator), we analyzed these outcome as continuous, rather than as a percentage.

**For non-ITS outcomes:**
For outcomes with very long follow-up periods (>48 months), if data were available, we calculated effect sizes using data from the shorter follow-up periods during which patterns observed seemed to be plausibly attributed to the intervention.

In pre-post studies with controls, for outcomes that were continuous, unbounded, and negative at baseline, we multiplied the relative change from baseline-to-follow-up for any arm with the negative baseline value by (-1) in order to use the absolute value of the reference value, and to ensure the relative change was calculated correctly (i.e., a positive change indicates improvement). In post-only studies with randomized controls, for outcomes that were continuous, unbounded, and negative for the referent arm, we multiplied the relative change between arms at follow-up by (-1).

> Example: idnum 62000001, post-only with randomized controls, outcome 29:
> Arm 1 follow-up Z score = -0.25
> Arm 2 follow-up Z score = -0.18
> Effect size = {-1} * {(100%)*[(-0.18 - (-0.25))/(-0.25)]} = 28 %-points

For pre-post studies with controls, if baseline measures for Arm 1 were not reported, and baseline measures for Arm 2 were reported, the effect size was calculated based only on post-intervention data.

In pre-post studies with controls, for outcomes that were continuous, unbounded, and zero at baseline for any study arm, we analyzed the absolute change from baseline-to-follow-up for both arms because

calculating the relative change would have resulted in an undefined effect size (i.e., a "divide by zero" error).

**For ITS outcomes:**
For outcomes with a very long baseline or follow-up period (>48 months) and with more than 6 measures available during that long period, we calculated effect sizes using only the 6 baseline measures closest to the time of the intervention and the 6 follow-up measures immediately after the intervention. We felt that measures beyond these 12 points would not reflect typical baseline trends or would not be plausibly attributed to the intervention.

If an outcome expressed as a percentage had predicted measures from a piecewise regression model that were below zero or >100%, the outcome was truncated at zero or 100%, respectively, for effect size calculations.

If a continuous outcome whose plausible values were >0 had predicted measures from a piecewise regression model that were below zero, the outcome was truncated at zero for effect size calculations.

One outcome had only 3 baseline measures, and those measures were at the beginning of a 12-month regular cyclical pattern that continued throughout the follow-up period (see below figure for idnum 218500001, outcome 3, % of clients who visited the HF for curative consultations per month among expected consultation per inhabitant per year). We felt that a segmented linear regression model using those 3 baseline measures would not adequately reflect the cyclical nature of the data and would lead to implausible predicted baseline and counterfactual values. Instead, we ran a linear regression model on the "residuals" when the empirical data points were compared with the predicted data points from a model with a cosine wave (i.e., residual = empirical data point minus predicted value from model with cosine wave). The residual is interpreted as the outcome after subtracting out the outcome's cyclical nature, which is based on data from an entire cycle that spans both the baseline and follow-up period.

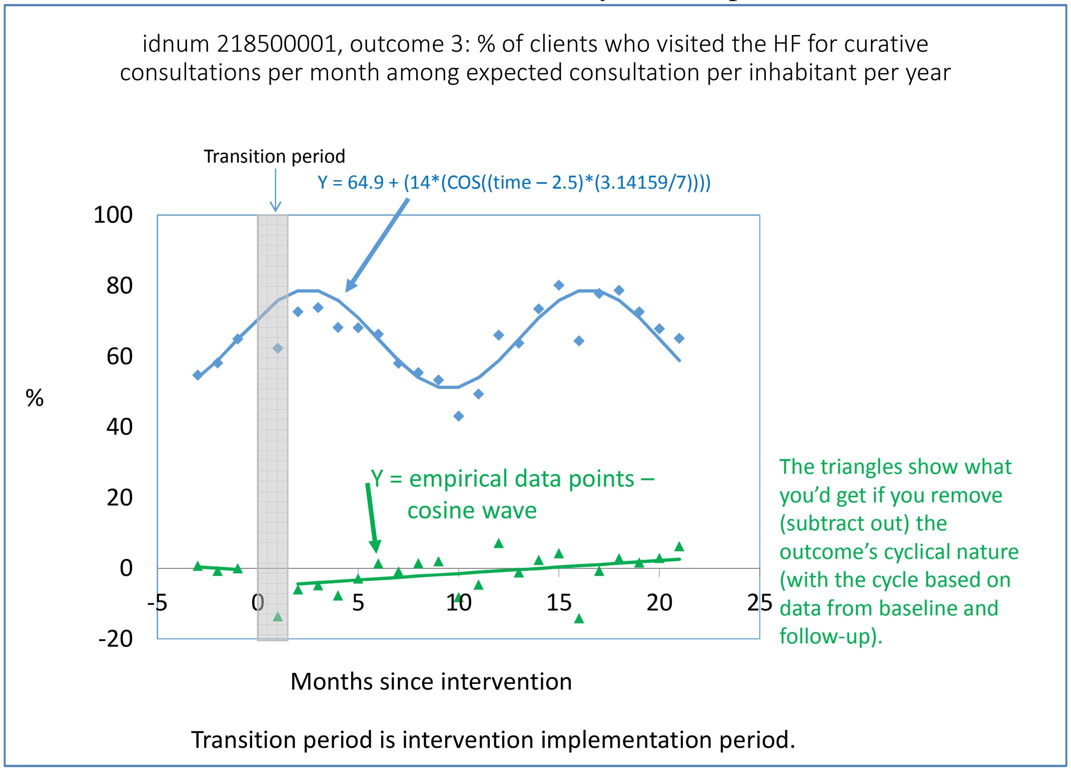

The predicted baseline was defined as the average predicted value from the cosine model during the 12 month period around 0 months since intervention. The predicted counterfactual was defined as the average predicted value from the cosine model during the 12 month period around the midpoint of the follow-up period. The predicted follow-up was defined as the average predicted value of the residual plus the predicted value from the cosine model during the 12 month period around the midpoint of the follow-up period. The single ITS effect size was defined as the average predicted follow-up minus the average predicted counterfactual. The calculations were as follows:

Average predicted baseline during 12 month period around 0 months = (sum of (cosine wave @ z month))/12 where z ranges from -6 to 5 = 65.4

Average predicted counterfactual during 12 month period around 10 months = (sum of (cosine wave @ z month))/12 where z ranges from 5 to 16 = 62.8

Average predicted follow-up during 12 month period around 10 months = (sum of (residual @ z month + cosine wave @ z month))/12 where z ranges from 5 to 16 = 61.7

Single ITS effect size
= average predicted follow-up – average predicted counterfactual
= 61.7 - 62.8 = -1.1

## Notes on coding outcome-level characteristics and strategies

### *Outcome-level characteristics*

We classified each outcome as pertaining to one health condition category. However, in reality, some outcomes relate to more than one health condition. For example, the categories of ‘Newborn’ and ‘Pregnancy’ overlap, such that some newborn outcomes might be classified under pregnancy, and vice versa. For instance, ‘average number of postpartum care visits per client’ (out_id 11800000102) indicates visits for the mother and child; we classified it as pregnancy. In another example, breastfeeding is conceptually related to ‘Malnutrition’ and ‘Newborn’ categories; we classified it as malnutrition. Also, we categorized outcomes related to delivery care but before the baby was born (e.g., out_id 20100000126: % of maximum score achieved for preparedness for resuscitation) as ‘Pregnancy’, rather than ‘Newborn’. We classified outcomes related to stillbirths as ‘Pregnancy’.

We classified each outcome as pertaining to one HCP action (usually with a short causal pathway from the quality improvement strategy), patient action, or patient health outcome category (usually with a long causal pathway from the strategy). However, in reality, some outcomes depend on both HCP actions and patient actions, primarily patient care-seeking. For example, trends in the outcomes of ‘% of child population fully immunized’ or ‘number of new cancer diagnoses per year’ might be due to a change in the process of care or a change in patient care-seeking. We classified outcomes that might depend on both HCP actions and patient care-seeking as ‘Patient care-seeking’.

### *Strategies*

We included studies that evaluated two general types of strategies to improve HCP performance: 1) strategies aimed at directly influencing HCP behaviors (e.g., training), or 2) strategies aimed at indirectly influencing HCP behaviors by changing the environment in which the HCP works. The ‘environment’ refers to the physical (i.e., health facilities or health service delivery points where HCPs work), policy (e.g., accreditation system, or new policy that allows community health workers to prescribe medicines), or financial environment (e.g., medical insurance or financial incentives). We excluded studies aimed to exclusively impact communities or patients (e.g., mass media education campaign only). However, we acknowledge that influencing community members or patients as a sole strategy could conceivably affect HCP practices (e.g., parents being more likely to accept no antibiotic treatment when their child has an upper acute respiratory infection [idnum 4500001]).

When classifying strategies, we noted the wide range of study types, with one extreme being those that are clearly testing a strategy to improve HCP performance (e.g., an RCT of training with an HCP behavior as the outcome—which is clearly eligible for the review) and the other extreme being a clinical study (e.g., an RCT of efficacy of new vaccine with morbidity as the outcome—which is clearly ineligible for the review). However, in between these extremes are studies that evaluate the effectiveness of a new programmatic approach with outcomes being a patient behavior or morbidity measure (e.g., idnum 123200001, an RCT comparing 3 versus 6 home visits to promote breastfeeding). These studies involve strategies to promote HCPs to follow a guideline, but their focus does not seem primarily to impact HCP performance; rather, the focus is on improving patient practices or morbidity; we excluded such studies from our review.

Strategy components had varying degrees of specificity. For example, on the broader end of the spectrum, training and continuous quality improvement (CQI) are both strategy components that could be designed or implemented in a variety of ways. For example, training could vary by duration, group size, and trainer attributes. CQI could receive only local support from investigators or national support from a national Quality Assurance committee [idnum 73000001]). On the narrower end of the spectrum,

there are many different strategy components for community or patient education (e.g., printed materials, home visits, group meetings, and video). One reason for variation along the spectrum is that in the review's protocol, there was variation in some strategy components such as training and community education. The strategy components with broader definitions rarely needed additional specification (because they were originally defined as broad). In contrast, strategy components with narrower definitions often required the creation of additional component definitions (because the original definitions were too specific)—for example, the original component 'Management Information System (MIS) with a paper form' required the creation of new components for variations such as 'MIS that was electronic' or 'MIS not specified as paper or electronic'.

We considered the introduction of a new type of HCP, a new responsibility for a pre-existing HCP cadre, or a new standard clinical guideline to be a strategy's contextual factor, rather than a strategy component. Furthermore, we considered interventions aimed at HCP supervisors (e.g., training or supervision of HCP supervisors, financial incentives for HCP supervisors) to be a supervision strategy's contextual factor, rather than a strategy component. For example, in a study in India (idnum 225300001), HCPs were trained to instruct community members about a new family planning guideline, which included new family planning methods and the introduction of sterilization services at a health facility. We classified the training of HCPs as a strategy component and the new family planning guidelines as a contextual factor.

Regarding the coding of training, if a training strategy involved a mix of training types, then training components reflected a combination of the training types. For example, if the training was a mix of small group and large group sessions, the group size was coded as large group. If the training was a mix of academic detailing at the HCP's worksite and off-site lectures, then the training setting was coded as both on- and off-site. We coded "academic detailing" training as having a small group size, being ongoing, and involving interactive sessions.

## Notes on analysis

*General comments*

For studies that cover multiple countries, the estimated gross domestic product (GDP) or gross national income (GNI) for the study was the median GDP or GNI across all countries in study. For example, for idnum 104600001, which involved Nigeria and Ghana, the estimated GDP was the average of the 2 countries' GDPs: (263.289 + 385.68)/2 = 324.48 US dollars.

When analyzing the duration of a training that lasted a range of days, we defined duration as the average of the upper and lower limit of the range of the number of days. If either the upper or lower limit of the range was missing, then the duration was defined as the limit that was present.

*For ITS studies*

If an ITS study had 2 arms, and the control/comparison arm had only 1 cluster, the control/comparison arm was dropped, and the study was re-analyzed as a single arm study.

If an ITS study had more than 1 arm, and all of the arms involved an active intervention (i.e., no true control arm), then the summary effect sizes that were calculated involved comparisons of one arm to another arm (Arm 1 versus Arm 2, Arm 1 versus Arm 3, Arm 2 versus Arm 3, etc.) and comparisons of one arm to its own historical control (i.e., the summary effect size based on the segmented regression analysis for each study arm alone).
