# Supplementary material for: A systematic review of the effectiveness of strategies to improve health care provider performance in low- and middle-income countries: Methods and descriptive results
Source: PLoS One. 2019 May 31;14(5):e0217617. doi: 10.1371/journal.pone.0217617 (PMC6544255; doi:10.1371/journal.pone.0217617)
Supplement: S4 File — (PDF) [file pone.0217617.s004.pdf]

Appendix 4. Detailed definitions of the 10 strategy component categories

This appendix presents detailed definitions of the 10 strategy component categories used in the Health Care Provider Performance Review. After each of the 10 strategy component categories, in square brackets, is an example dichotomous variable in the review's database that codes for the presence of the component category in a given strategy. For each of the 10 categories, there is a list of individual strategy components. After each individual strategy component, in square brackets, is an example dichotomous variable in the review's database that codes for the presence of the component in a given strategy. At the end of this appendix, a detailed definition of the control group is presented as an 11th category.

<u>Abbreviations</u>

HCP	Health care provider
HF	Health facility
STG	Standard treatment guideline

1. Patient and community support [QQA1_ConsumSupp]

- HCPs distributed drugs via mass community-wide campaign [q17i7_MassDrug]
- HCPs distributed immunizations via mass community-wide campaign [q17i7_MassImmun]
- Conditional cash transfers to households for health services or behaviors [q17L1]
- Community member received a non-conditional cash transfer [q17L9_CashTransfer]
- Community health education via group meetings (includes theater) [q17L2]
- Community health education via home visits [q17L3]
- Community health education via printed materials [q17L5]
- Community health education via radio [q17L6]
- Community health education via TV [q17L7]
- Community health education via broadcast media [q17L9_CommEduBroad]
- Community health education via children who were exposed to the health messages outside the home [q17L9_CommEduChild]
- Community health education via videos or films [q17L9_CommEduVid]
- Community health education via distribution methods other than group meetings, home visits, internet, printed materials, radio, TV, broadcast media, children, or videos [q17L9_OthCommEduc]
- Community health education via unspecified distribution methods [q17L9_unspecCommEduc]
- Emergency telephone line was set up for consultations and to clarify when to use hospitals [q17L9_EmergPhone]
- Patient health education via one-on-one meetings with HCPs (excludes health education during routine consultations) [q17L9_PtEduc1on1]
- Patient health education via home visits [q17L9_PtEducHome]
- Patient health education via group meetings [q17L9_PtEducMtg]
- Patient health education via printed materials [q17L9_PtEducPrint]
- Patient selected a support person [q17L9_PtSocSupp]
- Social marketing or promotion of health goods and services [q17o2]
- HF-based HCPs provided care in community settings (outreach) [q17c13_Outreach]
- Community members were allowed to choose their own HCPs [q17L9_CommHCPchoice]
- Communities were given health-related supplies (e.g., bednets) that were not intended to directly impact HCP performance [q17r_ComHlthSuppl]
- Community health education via internet [q17L4]
- Vouchers or in-kind subsidies to households for health services or behaviors [q17L8]
- Communities were exposed to non-health-related educational messages [q17r_nonHlthEdu]

- Strategy to strengthen relationships and cooperation among community members [q17L9_CommCoop]
- Communities were given non-health-related resources (e.g., loan for household) that were neither intended to directly impact HCP performance nor support implementation of health interventions [q17r_ComNonHlthSuppl]
- Patient health education via distribution methods other than group meetings, home visits, internet, printed materials, radio, TV, broadcast media, children, or videos [q17L9_OthPtEduc]
- Community or patient support activities (e.g., health education group meetings, home visits, printed materials) were reduced or removed [q17L9_RedComPtSupp]

2. Strengthening infrastructure [QQA1_Infrastruc]

- Printed management information system form or register [q17g2]
- Health services performance reporting [q17g3]
- Improved data collection system (without details on how data collection was improved) [q17g4_imprDataCol]
- Data were collected in new or modified electronic management information system and used to improve services [q17g4_newDataCol]
- Medical equipment inventory management or maintenance system [q17e1]
- Facilities repair or rebuilding [q17f2]
- Standard HF specifications were introduced [q17f4_StdHFSpecs]
- Provision of drugs [q17d12]
- Provision of medical equipment [q17e4]
- Provision of facility or new service provision point (e.g., in a school) [q17f3]
- Non-medical equipment or supplies to support implementation of health interventions [q17r_nonMedEquip]
- Structured stock ordering [q17d11]
- Essential drug list [q17d2]
- Drug therapeutic committee [q17d3]
- Standardized procurement systems [q17d8]
- At the district level, intermediary drug wholesalers were introduced [q17d13_DrWholesale]
- Improved medicine logistics [q17d13_imprMedLogis]
- Unnecessary HFs were removed (e.g., by demolishing, renting out, or changing use of HF) [q17f4_RemoveXSHF]
- A restricted drug list was implemented at HF [q17d13_RestrDrugList]
- Automatic stop order [q17d1]
- Kit systems [q17d6]
- Medical technology assessment systems [q17e2]
- Standardized medical technology list [q17e3]
- Facilities maintenance [q17f1]
- Computerized standardized medical records [q17g1]
- Hiring additional HCPs into pre-existing HCP cadre [q17r_HireHCP]
- HF or HCP kept records of drug purchases, sales, or stock [q17d13_MedRecKeep]

3. Financing and incentives [QQA1_Fin_Incent]

- The government exonerated importation taxes for essential medicines [q17d13_NoTax]
- Private sources of revenue [q17m3]
- Payment/incentive: Unspecified user fees were reduced or removed [q17m5_RedUserFee]
- Government funds were released for purchase of drugs [q17m6_GovDrugFund]
- Government funds were allocated to health sector [q17m6_GovHlthAlloc]
- Social health insurance [q17n4]
- Revolving drug funds [q17n5]
- A loan fund for HCPs or HFs was set up [q17n7_LoanFund]
- A fund for patients was set up [q17n7_PatientFund]
- Payment/incentive: Patients who were dispensed medicines were charged the cost of the drug packaging [q17p10_PkgFee]
- Payment/incentive: Consultation fee was introduced or increased [q17p2_IncConsFee]
- Payment/incentive: Consultation fee was reduced or removed [q17p2_RedConsFee]
- Payment/incentive: Fee per drug/health commodity item was introduced or increased [q17p3_IncDrugFee]
- Payment/incentive: Fee per drug/health commodity item was reduced or removed [q17p3_RedDrugFee]

- Payment/incentive: Prescription fee was introduced or increased [q17p8_IncPresFee]
- Payment/incentive: Fee per service was introduced or increased [q17p4_IncServFee]
- Payment/incentive: Fee per service was reduced or removed [q17p4_RedServFee]
- Payment/incentive: Performance-based financial incentives for staff [q17p6]
- Payment/incentive: Performance-based non-financial incentives for staff [q17p7]
- Payment/incentive: Non-performance-based financial incentives for HCP [q17p10_FinIncent]
- Payment/incentive: Non-performance-based non-financial incentives for HCP [q17p10_NonFinIncent]
- Payment/incentive: Salaries [q17p9]
- Payment/incentive: Performance-based financial incentives for HFs [q17p10_HFPerfFinIncen]
- Payment/incentive: Non-performance-based financial incentives for HFs [q17p10_HFNonPerfFinInc]
- Retail prices of essential medicines and consumables were set by government [q17q7_SetPrice]
- HF received recognition after meeting certain criteria [q17q7_HFaward]
- HF owners were given incentives [q17p10_HFownerIncent]
- Contracting-in services [q17j1]
- Contracting-out services [q17j2]
- Other contracting [q17j4_OthContract]
- Foreign countries/multinational org/bilateral organization (e.g., World Bank) donated funds to support strategies [q17m6_ForeignDonat]
- Funds were donated to support strategies, source unspecified [q17m6_UnspecFund]
- Different insurers were unified into a single insurer [q17n7_SingleInsurer]
- Contracting with incentives at contractor's level [q17j3]
- Donations as a source of revenue for health [q17m1]
- Insurance premiums [q17m2]
- Taxes as a source of revenue for health [q17m4]
- Community-based health insurance [q17n1]
- Enterprise-based health insurance [q17n2]
- Private/voluntary health insurance [q17n3]
- Personal health savings [q17n6]
- Capitation fee [q17p1]
- Global budgets to HFs or organizations [q17p5]
- Disciplinary action within HCP organization [q17h1]
- Personnel development practices [q17h4]
- Promotion practices [q17h5]
- Payment/incentive: Unspecified user fees were introduced or increased [q17m5_IncUserFee]
- Reimbursement (e.g., by insurance) or subsidy to HF or HCP was revised [q17p10_RevisReimbur]
- Promotion of brand name competitor drug [q17O3_BrCompPromo]
- Reduction in price of a brand name competitor drug [q17p3_BrCompRed]
- Reduction in price of a generic competitor drug [q17p3_GenCompRed]
- Reduction in price of a drug named in study outcome [q17p3_StudyDrugRed]

4. Regulation and governance [QQA1_InstituApp]

- Standard drug quality requirements were introduced [q17q7_StdDrugQual]
- Franchising or branding private HCPs [q17o1]
- Enforcement approach [q17q2]
- Accreditation [q17q3_Accreditation]
- Certification [q17q3_Certification]
- Licensing [q17q3_Licensing]
- Registration [q17q3_Registration]
- Redress mechanism [q17q4]
- Sanctions based on HCP qualifications or facility structural factor [q17q5]
- Sanctions based on services or negligence of HCP [q17q6]
- A code of ethics for HCPs or HFs was created [q17q7_CodeOfEthics]
- Public-private partnerships not involving contracting [q17i1]
- Resource control for health services given to civil society organizations [q17i4]

- Resource control for health services given to local governments [q17i5]
- Civil society organization oversight of HCPs [q17k1]
- HFs were now in control of fee revenues [q17i7_HFcontrol]
- Responsibility or authority for HCP decisions to local governments [q17i2]
- Responsibility or authority for HCP decisions to local health agencies [q17i3]
- Resource control for health services given to local public health agencies [q17i6]
- Consumer ratings of HCPs [q17k2]
- Patient bill of rights [q17k3]
- Banning drug or formulation [q17q1]
- Approval of a brand name competitor drug by committee at HF level or higher [q17d13_BrCompApprov]
- Approval of a generic competitor drug by committee at HF level or higher [q17d13_GenCompApprov]
- Approval of a drug named in study outcome by committee at HF level or higher [q17d13_StudyDrugApprov]

5. Other management techniques (i.e., not group problem solving or supervision) [QQA1_OtherMgmnt]

- Risk management [q17c10]
- HCP group process that is neither group training nor team-based problem solving [q17c13_GroupProc]
- Group meeting of HCPs and community members or patients [q17c13_GroupHCPcom]
- HCPs were given job descriptions [q17c13_HCPjobDesc]
- HCP self-assessment [q17c13_SelfAssess]
- Each HF set its own monthly performance targets [q17c13_SetTarget]
- HCPs participated in unspecified group meeting [q17c13_unspecGrpMtg]
- Reorganized management structure [q17c9]
- Hiring and selection process [q17h2]
- One or more new health services was integrated into the regular activities of HFs [q17i7_IntegServ]
- HF and HCPs were linked in a referral network [q17r_HCP_HF_link]
- Structured prescribing [q17d10]
- Change in process of care to improve accessibility of medicines [q17d13_imprMedAcces]
- Generic substitution [q17d5]
- Pre-packaging drugs [q17d7]
- Change in process of care to improve utilization of health services (including pharmaceuticals and follow-up care) [q17d13_imprMedUtil]
- Management of HCPs was decentralized to HF level (e.g., management committee was created at HF that gave staff more decision-making rights regarding management of HF) [q17c13_DecentralMgmt]
- HCPs received training on management skills (business planning, record-keeping, financial reporting, credit management, marketing) [q17c13_HCPMgmtTrain]
- Prior authorization of pharmaceuticals [q17d9]
- Operations research [q17c7]
- Regular monitoring on parameters not related to HCP clinical practice (e.g., HCP knowledge, patient outcomes) [q17c13_MonitorNonPrac]
- Reorganization of how existing HCPs are deployed (e.g., Higher-level-HF-based HCPs provided care in lower-level HFs) [q17c13_HRHReorg]
- HCPs received feedback that was not based on data collected from an audit or supervisory visit [q17c13_NonAudSupFdbk]
- Restrictions for prescribing a brand name competitor drug [q17d13_BrCompRestr]

6. Group problem solving [QQA1_GrpProbSol]

- Continuous quality improvement [q17c12]
- Improvement Collaborative [q17c13_ImprovCol]
- HCPs held meetings to discuss problems and solutions (but not formal teams) [q17c13_ProbSolv]
- Team-based problem solving [q17c3]

7. Supervision [QQA1_Supervisin]

- Benchmarking [q17c1]
- Supervision [q17c11]

- HCPs sought second opinion from peer or higher level HCP [q17c13_2ndOpinion]
- HCP received instructions from higher level HCP [q17c13_InstrHCP]
- Managers of HCPs received supervision [q17c13_MgrSup]
- Managers of HCPs received training [q17c13_MgrTrain]
- HCP received support from non-supervisory staff [q17c13_nonSupHelpHCP]
- Audit with in-person feedback [q17c4]
- Audit with written feedback [q17c5]
- Monitoring of HCP practice parameters [q17c6]
- Peer review [q17c8]
- HFs were inspected to monitor for deviations from regulations [q17q7_Inspection]
- Drug utilization review/evaluation [q17d4]
- Performance appraisal practices [q17h3]

8. High-intensity training [QQA1_TrainHiInt]

- Training with a duration greater than 5 days (or ongoing training) and at least one interactive education method (i.e., clinical practice, role play, or interactive sessions) [(q17a1 = -1) AND (QQA1_TR_DUR >5 OR q17a1bi = -1) AND (QQA1_TR_INTERACT = 1)]
- Academic detailing (i.e., one-on-one training by an opinion leader) [q17a1_AcademDet]

9. Low-intensity training [QQA1_TrainLoInt]

- Any training not categorized as high-intensity training (above) [(q17a1 = -1 OR q17a1_AcademDet = -1 OR q17r_PeerEduc = -1) AND QQA1_TrainHiInt = 0]
- Informal education of HCPs by their peers [q17r_PeerEduc]

10. Printed or electronic information (including job aids) for HCPs that is not an integral part of another component [QQA1_HCP_Inform]

- Printed pamphlet for HCP (excludes STGs) [q17b4]
- Printed patient recording form [q17b5]
- Printed STG [q17b6]
- Poster for HCP [q17b7]
- Other printed job aid for HCP (e.g., counseling cards for nurses, map of referral centers) [q17b8_OthJobaid]
- Computerized job aid [q17c2]
- Label or stamp that HCP uses to depict dosing [q17d13_DrugLabel]
- Printed newsletter for HCP [q17b3]
- Printed literature for HCP (not considered a job aid) [q17b8_Literature]
- Electronic copies of literature for HCP (not considered a job aid) [q17r_electronLit]
- Bulletin for HCP [q17b1]
- Formulary manual [q17b2]
- Educational materials for HCPs to study on their own [q17b8_SelfStudy]

11. Control group components

- No intervention [q17s]
- Placebo training [q17a1_PlaceboTr]
- Placebo printed STG [q17b8_PlaceboSTG]
- Placebo supervision [q17c13_PlaceboSup]
- Placebo community education group meetings [q17L9_PlaceboComMtg]
- Placebo community education home visit [q17L9_PlaceboComVis]
