# Supplementary material for: A systematic review of the effectiveness of strategies to improve health care provider performance in low- and middle-income countries: Methods and descriptive results
Source: PLoS One. 2019 May 31;14(5):e0217617. doi: 10.1371/journal.pone.0217617 (PMC6544255; doi:10.1371/journal.pone.0217617)
Supplement: S5 File — (PDF) [file pone.0217617.s005.pdf]

Appendix 5. Detailed flowchart of the literature search, as recommended by Preferred Reporting Items for Systematic Reviews and Meta-Analyses (PRISMA) guidelines

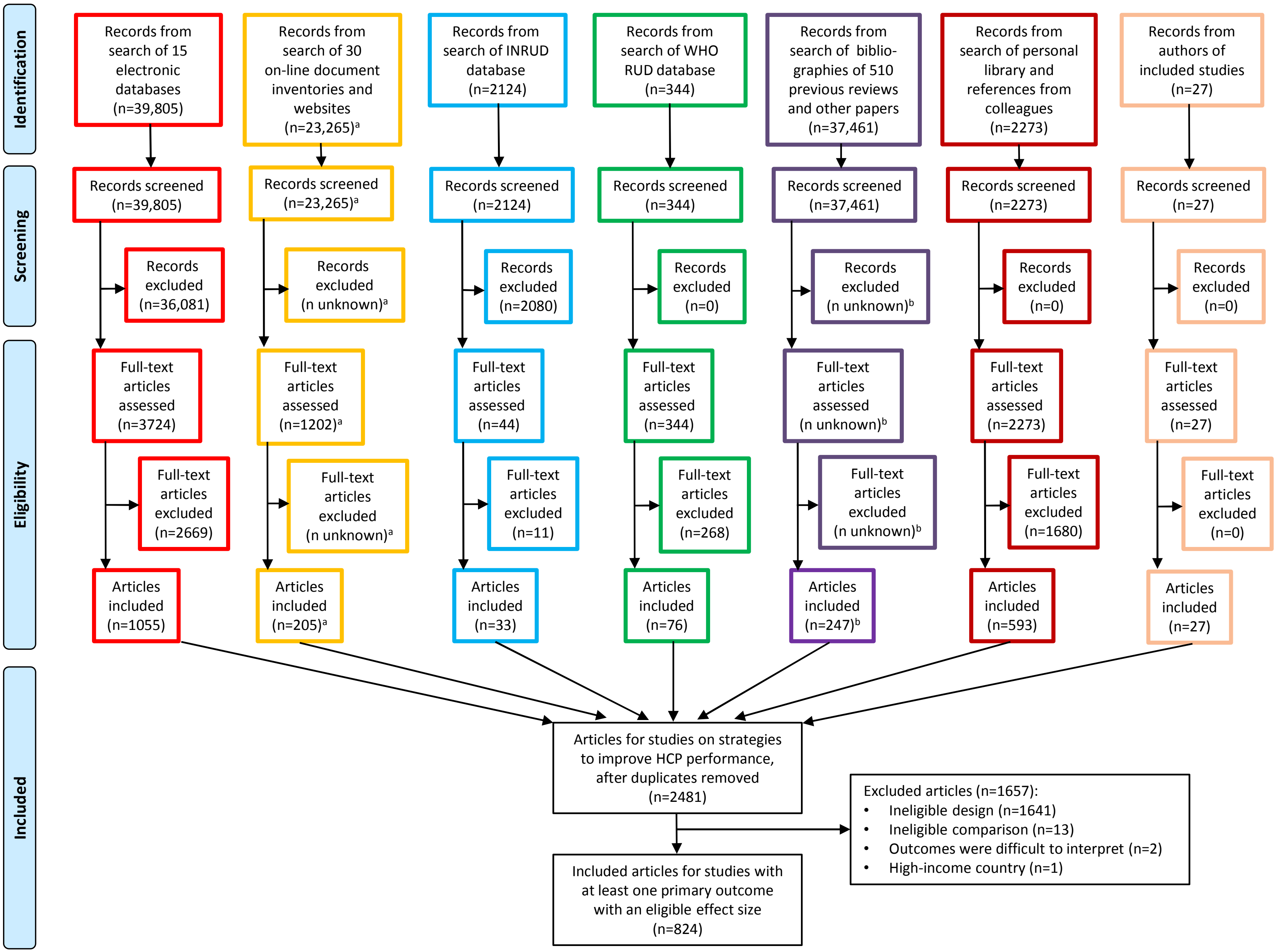

Footnotes to Appendix 5.

HCP = health care provider, INRUD = International Network for the Rational Use of Drugs, RUD = Rational Use of Drugs, WHO = World Health Organization.

[a] Early in the search of on-line document inventories and websites, detailed records were not kept of the number of citations that were screened. Thus, the number of exclusions is unknown; the exact number of records screened is unknown, but was more than 23,265 (which reflects the number once detailed records began to be kept); the exact number of full-text articles assessed is unknown, but was more than 1202 (which reflects the number once detailed records began to be kept); and the exact number of included articles is unknown, but was more than 205 (which reflects the number once detailed records began to be kept).

[b] Early in the search of the bibliographies of the 510 previous reviews and other papers, detailed records of the search were not kept. Thus, the number of exclusions and full-text assessments are unknown; and the exact number of included articles is unknown, but was more than 247 (which reflects the number once detailed records began to be kept).
