# Supplementary material for: A systematic review of the effectiveness of strategies to improve health care provider performance in low- and middle-income countries: Methods and descriptive results
Source: PLoS One. 2019 May 31;14(5):e0217617. doi: 10.1371/journal.pone.0217617 (PMC6544255; doi:10.1371/journal.pone.0217617)
Supplement: S7 File — (PDF) [file pone.0217617.s007.pdf]

Appendix 7. Additional results

Abbreviations

HCP Health care provider
ITS Interrupted time series

Table A7-1. Distribution of the number of clusters in the study arm with the fewest number of clusters, for the 377 studies that did not use an ITS design

Note: Among studies that did not use an ITSs design, this attribute is important because studies with a small number of clusters per study arm were considered to have an increased risk of bias (see Appendix 3). Among all 377 studies that did not use an ITS design, the mean number of clusters in the study arm with the fewest number of clusters was 29 (median: 4, interquartile range: 1 to 13, range: 1 to 1146).

Number of clusters in the study arm with the fewest number of clusters	No. (%) (N=377 studies)
1	105 (27.9)
2–3	64 (17.0)
4–5	36 (9.5)
6 or more	172 (45.6)

Table A7-2. Distribution of individual risk-of-bias domains for all 499 studies

Risk of bias domain[a]	All studies		
	Done No. (%)	Unclear No. (%)	Not done No. (%)
Dataset was complete (N = all 499 studies)	314 (62.9)	107 (21.4)	78 (15.6)
Reliable primary outcome (N = all 499 studies)	470 (94.2)	15 (3.0)	14 (2.8)
Balance in baseline outcome measurements (N = 133 randomized controlled trials, excluding controlled ITS)	64 (48.1)	4 (3.0)	65 (48.9)
Balance in characteristics between study arms (N = 64 post-only randomized controlled trials)	18 (28.1)	22 (34.4)	24 (37.5)
Concealment of allocation (N = 11 randomized controlled trials randomized at the individual level, excluding controlled ITS)	4 (36.4)	7 (63.6)	0 (0)
Intervention was independent of other changes (N = 101 ITS with no controls)	31 (30.7)	20 (19.8)	50 (49.5)
At least 6 measures before and at least 6 measures after intervention (N = 122 ITS studies)	68 (55.7)	0 (0)	54 (44.3)
Intervention unlikely to have affected data collection (N = 295 studies, excluding randomized controlled trials that are not ITS studies)	243 (82.4)	6 (2.0)	46 (15.6)

Footnote.

[a] Some domains only apply to a subset of all studies.

Table A7-3. Risk of bias for the 326 studies that used a randomized or ITS design

Risk of bias	No. (%)
Low	66 (20.3)
Moderate	102 (31.3)
High	92 (28.2)
Very high	66 (20.3)

Table A7-4. Distribution of the number of clusters in the study arm with the fewest number of clusters, for the 204 randomized studies that did not use an ITS design

Note: Among randomized studies that did not use an ITS design, this attribute is important because studies with a small number of clusters per study arm were considered to have an increased risk of bias (see Appendix 3). Among all 204 randomized studies that did not use an ITS design, the mean number of clusters in the study arm with the fewest number of clusters was 37 (median: 8, interquartile range: 3 to 20, range: 1 to 1110).

Number of clusters in the study arm with the fewest number of clusters (effect on risk of bias assessment)	No. (%) (N=204 studies)
1	24 (11.7)
2–3	32 (15.7)
4–5	22 (10.8)
6 or more	126 (61.8)

Table A7-5. Distribution of individual risk-of-bias domains for the 326 studies that used a randomized or interrupted time series design

Risk of bias domain[a]	All studies		
	Done No. (%)	Unclear No. (%)	Not done No. (%)
Dataset was complete (N = all 326 studies)	212 (65.0)	60 (18.4)	54 (16.6)
Reliable primary outcome (N = all 326 studies)	312 (95.7)	5 (1.5)	9 (2.8)
Balance in baseline outcome measurements (N = 133 randomized controlled trials with pre- and post-intervention measure, excluding controlled ITS studies)	64 (48.1)	4 (3.0)	65 (48.9)
Balance in characteristics between study arms (N = 64 post-only randomized controlled trials)	18 (28.1)	22 (34.4)	24 (37.5)
Concealment of allocation (N = 11 randomized controlled trials randomized at the individual level excluding controlled ITS studies)	4 (36.4)	7 (63.6)	0 (0)
Intervention was independent of other changes (N = 101 ITS studies with no controls)	31 (30.7)	20 (19.8)	50 (49.5)
At least 6 measures before and at least 6 measures after intervention (N = 122 ITS studies)	68 (55.7)	0 (0%)	54 (44.3)
Intervention unlikely to have affected data collection (N = 295 studies, excluding randomized controlled trials that are not ITS studies)	243 (82.4)	6 (2.0)	46 (15.6)

Footnote.

[a] Some domains only apply to a subset of all studies.

Table A7-6. Distribution of 161 combinations[a] of the 10 strategy component categories[b] among all active intervention arms

Combination of the 10 strategy component categories	No. (%) of study arms with the combination of strategy component categories (N = 687 arms)
................9...	82 (11.9)
..............8.....	30 (4.4)
1...............9...	30 (4.4)
....3...............	28 (4.1)
............7.......	26 (3.8)
1.2.............9...	23 (3.4)
............7...9...	21 (3.1)
1.............8.....	19 (2.8)
..........6.........	18 (2.6)
1...........7...9...	17 (2.5)
..2.............9...	14 (2.0)
..................10	13 (1.9)
....3.4.5...........	12 (1.8)
1...................	12 (1.8)
............7.8.....	11 (1.6)
1.2.........7...9...	10 (1.5)
..2.........7...9...	9 (1.3)
1...........7.8.....	9 (1.3)
1.2...4.........9...	8 (1.2)
1.2...........8.....	7 (1.0)
1.2...4.5...7...9...	7 (1.0)
........5.......9...	6 (0.9)
1.......5.......9...	6 (0.9)
1.....4.........9...	6 (0.9)
1.2.....5.......9...	6 (0.9)
1.2.....5...7...9...	6 (0.9)
............7.....10	5 (0.7)
....3.4.............	5 (0.7)
1.......5...........	5 (0.7)
1.2...4.....7...9...	5 (0.7)
1.2.3...............	5 (0.7)
..........6.....9...	4 (0.6)
....3...5...........	4 (0.6)
..2.3.......7.8.....	4 (0.6)
1.......5...7...9...	4 (0.6)
1.2.................	4 (0.6)
1.2.....5...7.8.....	4 (0.6)
1.2...4.5...7.8.....	4 (0.6)
1.2.3.......7.8.....	4 (0.6)

Combination of the 10 strategy component categories	No. (%) of study arms with the combination of strategy component categories (N = 687 arms)
1.2.3.4.....7...9...	4 (0.6)
..........6.7.....10	3 (0.4)
..........6.7.8.....	3 (0.4)
........5.........10	3 (0.4)
........5...7...9...	3 (0.4)
......4.5...7...9...	3 (0.4)
....3.......7.......	3 (0.4)
....3.......7.....10	3 (0.4)
..2.................	3 (0.4)
..2.........7.8.....	3 (0.4)
..2.....5.......9...	3 (0.4)
..2.....5...7...9...	3 (0.4)
1.................10	3 (0.4)
1.......5.........10	3 (0.4)
1...3...........9...	3 (0.4)
1...3.......7...9...	3 (0.4)
1...3...5...7.......	3 (0.4)
1.2.........7.......	3 (0.4)
1.2.3...5...7.8.....	3 (0.4)
1.2.3.4.5...7...9...	3 (0.4)
1.2.3.4.5...7.8.....	3 (0.4)
........5...........	2 (0.3)
........5.....8.....	2 (0.3)
........5...7.......	2 (0.3)
........5...7.....10	2 (0.3)
........5...7.8.....	2 (0.3)
........5.6.7.8.....	2 (0.3)
......4.............	2 (0.3)
......4...6.........	2 (0.3)
....3...........9...	2 (0.3)
....3.......7.8.....	2 (0.3)
....3...5.6...8.....	2 (0.3)
..2...........8.....	2 (0.3)
..2.........7.......	2 (0.3)
..2.......6.....9...	2 (0.3)
..2.....5...7.8.....	2 (0.3)
..2.....5.6.7.8.....	2 (0.3)
..2.3...............	2 (0.3)
..2.3.4.5...7...9...	2 (0.3)
1.......5...7.8.....	2 (0.3)
1...3.......7.8.....	2 (0.3)
1...3.4.5.6.7.8.....	2 (0.3)

Combination of the 10 strategy component categories	No. (%) of study arms with the combination of strategy component categories (N = 687 arms)
1.2...............10	2 (0.3)
1.2.........7.8.....	2 (0.3)
1.2.......6...8.....	2 (0.3)
1.2.....5.6.....9...	2 (0.3)
1.2...4.5.......9...	2 (0.3)
1.2.3...5.......9...	2 (0.3)
1.2.3...5...7...9...	2 (0.3)
1.2.3.4.....7.8.....	2 (0.3)
............7...9.10	1 (0.2)
..........6.......10	1 (0.2)
..........6...8.....	1 (0.2)
..........6.7...9...	1 (0.2)
........5.6.......10	1 (0.2)
........5.6.7.......	1 (0.2)
........5.6.7...9...	1 (0.2)
......4.....7...9...	1 (0.2)
......4.....7.8.....	1 (0.2)
......4...6.7.......	1 (0.2)
......4.5...........	1 (0.2)
....3.........8.....	1 (0.2)
....3.....6.........	1 (0.2)
....3...5.........10	1 (0.2)
....3...5...7.......	1 (0.2)
....3.4.....7.......	1 (0.2)
....3.4.5...7.......	1 (0.2)
..2.......6.7...9...	1 (0.2)
..2.....5...........	1 (0.2)
..2.....5.........10	1 (0.2)
..2.....5...7.......	1 (0.2)
..2.....5...7.....10	1 (0.2)
..2.....5.6.7.......	1 (0.2)
..2...4.............	1 (0.2)
..2...4.....7...9...	1 (0.2)
..2.3.......7.......	1 (0.2)
..2.3.......7...9...	1 (0.2)
..2.3.....6.7.8.....	1 (0.2)
..2.3...5...7.......	1 (0.2)
..2.3...5...7...9...	1 (0.2)
..2.3...5...7...9.10	1 (0.2)
..2.3.4.............	1 (0.2)
..2.3.4.....7...9...	1 (0.2)
1...........7.......	1 (0.2)

Combination of the 10 strategy component categories	No. (%) of study arms with the combination of strategy component categories (N = 687 arms)
1.........6.....9...	1 (0.2)
1.......5...7.......	1 (0.2)
1.......5...7.....10	1 (0.2)
1.....4.....7.......	1 (0.2)
1.....4.....7.8.....	1 (0.2)
1...3...............	1 (0.2)
1...3.........8.....	1 (0.2)
1...3.......7.......	1 (0.2)
1...3...5.....8.....	1 (0.2)
1...3...5...7.8.....	1 (0.2)
1...3...5.6.........	1 (0.2)
1...3...5.6.7...9...	1 (0.2)
1...3...5.6.7.8.....	1 (0.2)
1...3.4.....7...9...	1 (0.2)
1...3.4...6...8.....	1 (0.2)
1...3.4.5...........	1 (0.2)
1...3.4.5...7...9...	1 (0.2)
1...3.4.5...7.8.....	1 (0.2)
1...3.4.5.6.........	1 (0.2)
1.2.......6.........	1 (0.2)
1.2.......6.....9...	1 (0.2)
1.2.....5...........	1 (0.2)
1.2.....5.........10	1 (0.2)
1.2.....5...7.......	1 (0.2)
1.2.....5.6.7...9...	1 (0.2)
1.2...4.....7.8.....	1 (0.2)
1.2...4...6.7...9...	1 (0.2)
1.2...4.5.........10	1 (0.2)
1.2.3...........9...	1 (0.2)
1.2.3.........8.....	1 (0.2)
1.2.3.....6.7...9...	1 (0.2)
1.2.3...5...........	1 (0.2)
1.2.3...5.6.....9...	1 (0.2)
1.2.3.4.........9...	1 (0.2)
1.2.3.4.......8.....	1 (0.2)
1.2.3.4.5.........10	1 (0.2)
1.2.3.4.5.......9...	1 (0.2)
1.2.3.4.5.6.7...9...	1 (0.2)

Footnotes.

[a] For example, the combination "................9..." indicates "low-intensity training only", and "1..............9..." indicates "patient or community support + low-intensity training".

[b] The 10 strategy component categories are the following:

1) Patient or community support (e.g., community health education)
2) Strengthening infrastructure (e.g., provision of drugs)
3) Financing and incentives (e.g., changing user fees)
4) Governance or regulation (e.g., accreditation schemes)
5) Management techniques, excluding group problem solving and supervision (e.g., changing processes of care to improve utilization of health services)
6) Group problem solving (e.g., continuous quality improvement)
7) Supervision (e.g., improving routine supervision)
8) High-intensity training (i.e., duration > 5 days or ongoing training or academic detailing; and at least one interactive education method, such as clinical practice, role play, or interactive sessions)
9) Low-intensity training (i.e., any training not categorized as high-intensity training); includes informal education of HCPs by their peers
10) Printed or electronic information or job aid for HCPs that is not an integral part of another component

Table A7-7. All 3943 effect sizes from all 499 included studies stratified by outcome type, outcome expression, non-inferiority study status, continuous outcome with baseline of zero, comparison type, and predominant health worker type

Outcome type	Outcome expression	Non-inferior-ity study	Continuous outcome with zero baseline	Comparison type	Predominant health worker type	No. of studies	No. of compari -sons	No. of effect sizes
Facilitators	Continuous	No	No	New strategy vs. no new strategy	Facility-based health workers	10	10	18
Facilitators	Continuous	No	No	New strategy vs. no new strategy	Lay health workers	2	2	4
Facilitators	Continuous	No	No	New strategy A vs. New strategy B	Facility-based health workers	2	6	6
Facilitators	Continuous	No	Yes	New strategy vs. no new strategy	Facility-based health workers	1	1	3
Facilitators	Percentage	No	No	New strategy vs. no new strategy	Facility-based health workers	38	46	184
Facilitators	Percentage	No	No	New strategy vs. no new strategy	Lay health workers	3	3	66
Facilitators	Percentage	No	No	New strategy A vs. New strategy B	Facility-based health workers	20	31	134
Facilitators	Percentage	No	No	New strategy A vs. New strategy B	Lay health workers	4	4	8
Process of care	Continuous	No	No	New strategy vs. no new strategy	Facility-based health workers	56	65	105

Outcome type	Outcome expression	Non-inferior-ity study	Continuous outcome with zero baseline	Comparison type	Predominant health worker type	No. of studies	No. of compari-sons	No. of effect sizes
Process of care	Continuous	No	No	New strategy vs. no new strategy	Lay health workers	1	1	1
Process of care	Continuous	No	No	New strategy A vs. New strategy B	Facility-based health workers	20	30	46
Process of care	Continuous	No	No	New strategy A vs. New strategy B	Lay health workers	2	7	7
Process of care	Continuous	No	Yes	New strategy vs. no new strategy	Facility-based health workers	1	1	2
Process of care	Percentage	No	No	New strategy vs. no new strategy	Facility-based health workers	145	172	807
Process of care	Percentage	No	No	New strategy vs. no new strategy	Lay health workers	7	7	33
Process of care	Percentage	No	No	New strategy A vs. New strategy B	Facility-based health workers	58	82	400
Process of care	Percentage	No	No	New strategy A vs. New strategy B	Lay health workers	3	8	10
Process of care	Percentage	Yes	No	New strategy vs. no new strategy	Facility-based health workers	1	1	1
Patient careseeking	Continuous	No	No	New strategy vs. no new strategy	Facility-based health workers	55	61	108
Patient careseeking	Continuous	No	No	New strategy vs. no new strategy	Lay health workers	4	5	7

Outcome type	Outcome expression	Non-inferior-ity study	Continuous outcome with zero baseline	Comparison type	Predominant health worker type	No. of studies	No. of compari-sons	No. of effect sizes
Patient careseeking	Continuous	No	No	New strategy A vs. New strategy B	Facility-based health workers	12	27	57
Patient careseeking	Continuous	No	No	New strategy A vs. New strategy B	Lay health workers	2	2	5
Patient careseeking	Continuous	No	Yes	New strategy A vs. New strategy B	Facility-based health workers	1	1	1
Patient careseeking	Percentage	No	No	New strategy vs. no new strategy	Facility-based health workers	42	49	136
Patient careseeking	Percentage	No	No	New strategy vs. no new strategy	Lay health workers	15	16	55
Patient careseeking	Percentage	No	No	New strategy A vs. New strategy B	Facility-based health workers	17	26	113
Patient careseeking	Percentage	No	No	New strategy A vs. New strategy B	Lay health workers	3	3	13
Patient non-health, non-careseeking outcome	Continuous	No	No	New strategy vs. no new strategy	Facility-based health workers	21	28	43
Patient non-health, non-careseeking	Continuous	No	No	New strategy vs. no new strategy	Lay health workers	8	8	13
Patient non-health, non-careseeking	Continuous	No	No	New strategy A vs. New strategy B	Facility-based health workers	10	14	15

Outcome type	Outcome expression	Non-inferior-ity study	Continuous outcome with zero baseline	Comparison type	Predominant health worker type	No. of studies	No. of compari-sons	No. of effect sizes
Patient non-health, non-careseeking	Continuous	No	No	New strategy A vs. New strategy B	Lay health workers	4	6	16
Patient non-health, non-careseeking	Percentage	No	No	New strategy vs. no new strategy	Facility-based health workers	71	84	447
Patient non-health, non-careseeking	Percentage	No	No	New strategy vs. no new strategy	Lay health workers	32	34	192
Patient non-health, non-careseeking	Percentage	No	No	New strategy A vs. New strategy B	Facility-based health workers	36	60	200
Patient non-health, non-careseeking	Percentage	No	No	New strategy A vs. New strategy B	Lay health workers	8	11	95
Patient health outcome	Continuous	No	No	New strategy vs. no new strategy	Facility-based health workers	47	52	123
Patient health outcome	Continuous	No	No	New strategy vs. no new strategy	Lay health workers	20	25	68
Patient health outcome	Continuous	No	No	New strategy A vs. New strategy B	Facility-based health workers	17	26	62
Patient health outcome	Continuous	No	No	New strategy A vs. New strategy B	Lay health workers	5	5	8
Patient health outcome	Percentage	No	No	New strategy vs. no new strategy	Facility-based health workers	32	36	91
Patient health outcome	Percentage	No	No	New strategy vs. no new strategy	Lay health workers	15	17	46

Outcome type	Outcome expression	Non-inferior-ity study	Continuous outcome with zero baseline	Comparison type	Predominant health worker type	No. of studies	No. of compari-sons	No. of effect sizes
Patient health outcome	Percentage	No	No	New strategy A vs. New strategy B	Facility-based health workers	16	20	37
Patient health outcome	Percentage	No	No	New strategy A vs. New strategy B	Lay health workers	7	9	18
Patient health outcome	Percentage	Yes	No	New strategy vs. no new strategy	Facility-based health workers	2	2	2
Cost	Continuous	No	No	New strategy vs. no new strategy	Facility-based health workers	43	45	81
Cost	Continuous	No	No	New strategy vs. no new strategy	Lay health workers	2	2	2
Cost	Continuous	No	No	New strategy A vs. New strategy B	Facility-based health workers	10	18	33
Cost	Percentage	No	No	New strategy vs. no new strategy	Facility-based health workers	5	8	10
Cost	Percentage	No	No	New strategy vs. no new strategy	Lay health workers	1	2	2
Cost	Percentage	No	No	New strategy A vs. New strategy B	Facility-based health workers	4	8	8
Cost	Percentage	No	No	New strategy A vs. New strategy B	Lay health workers	1	1	1

Table A7-8. Distribution of combinations[a] of the 10 HCP type categories[b] among all comparisons

Combination of the 10 HCP type categories	No. (%) of comparisons with the combination of HCP type categories (N = 687 comparisons)
CHW predominant	117 (17.0)
A.........	70 (10.2)
A.C.......	47 (6.8)
.........J	44 (6.4)
..C.......	35 (5.1)
...D...H..	20 (2.9)
.......H..	19 (2.8)
..C.....I.	19 (2.8)
......G...	18 (2.6)
A.C.....I.	18 (2.6)
........IJ	17 (2.5)
A.C....H..	16 (2.3)
A......H..	15 (2.2)
A.C.EF....	15 (2.2)
..C....H..	13 (1.9)
A...E.....	12 (1.8)
....E.....	11 (1.6)
A........J	11 (1.6)
A.C....HI.	9 (1.3)
..C.E...I.	8 (1.2)
.......HI.	7 (1.0)
..C....HI.	7 (1.0)
A.C......J	7 (1.0)
A.CD...H..	7 (1.0)
.BC.....I.	6 (0.9)
..C......J	5 (0.7)
A...E..HI.	5 (0.7)
A.C..F.HI.	5 (0.7)
A.C.E.....	5 (0.7)
....E..HI.	4 (0.6)
A.C.....IJ	4 (0.6)
A.CD......	4 (0.6)
AB........	4 (0.6)
.....F....	3 (0.4)
..C.EF..I.	3 (0.4)

Combination of the 10 HCP type categories	No. (%) of comparisons with the combination of HCP type categories (N = 687 comparisons)
A.......IJ	3 (0.4)
A......H.J	3 (0.4)
A..D......	3 (0.4)
A.C..F....	3 (0.4)
ABC.E.....	3 (0.4)
...D...HI.	2 (0.3)
...D.F.HI.	2 (0.3)
..C..F....	2 (0.3)
..C.E.....	2 (0.3)
.BC.......	2 (0.3)
.BC..F..I.	2 (0.3)
A.......I.	2 (0.3)
A..D.....J	2 (0.3)
A.C....H.J	2 (0.3)
A.CD.F..I.	2 (0.3)
ABC.......	2 (0.3)
.......H.J	1 (0.2)
.......HIJ	1 (0.2)
....E...I.	1 (0.2)
....E..H..	1 (0.2)
...D......	1 (0.2)
..C....HIJ	1 (0.2)
..C..F..I.	1 (0.2)
..C.E...IJ	1 (0.2)
..C.E..H..	1 (0.2)
..CD.F.H..	1 (0.2)
.BC....H..	1 (0.2)
.BC..F....	1 (0.2)
.BC.E.....	1 (0.2)
.BCD.F.H..	1 (0.2)
A......HI.	1 (0.2)
A.....G...	1 (0.2)
A....F....	1 (0.2)
A...E...IJ	1 (0.2)
A...E..H..	1 (0.2)
A..D....I.	1 (0.2)
A..D....IJ	1 (0.2)

Combination of the 10 HCP type categories	No. (%) of comparisons with the combination of HCP type categories (N = 687 comparisons)
A..D...HI.	1 (0.2)
A.C...G...	1 (0.2)
A.C..F...J	1 (0.2)
A.C..FGHI.	1 (0.2)
A.C.E...I.	1 (0.2)
A.C.E..H..	1 (0.2)
A.C.E..HI.	1 (0.2)
A.C.EF.H..	1 (0.2)
A.C.EF.HI.	1 (0.2)
A.CD...HI.	1 (0.2)
A.CD.F.H..	1 (0.2)
A.CD.F.HIJ	1 (0.2)
AB.D....I.	1 (0.2)
ABC....H..	1 (0.2)
ABC.E...I.	1 (0.2)
ABC.E..H..	1 (0.2)
ABCD.....J	1 (0.2)
ABCD.F.H..	1 (0.2)
ABCD.F.HI.	1 (0.2)

Footnotes.

[a] For example, the combination "A........." indicates "Physician only", and "A.C......." indicates "Physician + nurse/midwife". "CHW predominant" indicates "CHW only or CHW with other HCP types, but CHW was main focus of strategy".

[b] The 10 HCP type categories are the following:
A) Physician
B) Clinical officer
C) Nurse/Midwife
D) Pharmacist/Laboratorian
E) Paramedic/unspecified non-MD HCP
F) Health educator/information officer
G) Student
H) Aide
I) Community health worker
J) Unspecified health professional

Table A7-9. Table of excluded comparisons, outcomes, effect sizes, and outcome measures from included studies

Reason for exclusion	Number excluded
Comparison level	
Only a patient or community strategy was tested	3 comparisons
Timing of measurements between study arms was not comparable	3 comparisons
"No intervention versus placebo" comparison	2 comparisons
"Placebo versus active strategy" comparison in a 3-arm study that already had "no intervention versus active strategy" comparison	2 comparisons
Outcome level	
Outcome trend was difficult to interpret	143 outcomes
Did not contribute to at least 1 high priority effect size	35 outcomes
Subset of, or highly correlated with, primary outcome	15 outcomes
Small sample size for all measures that were required for analysis	1 outcome
Baseline data not available or did not accurately reflect the counterfactual trend. Interrupted time series analysis was not done.	9 outcomes
Not primary study outcome	9 outcomes
For percent outcome, all baseline measures were >=95%	5 outcomes
All baseline and follow-up measures were zero	8 outcomes
Data probably were incorrect (e.g., clinically implausible)	3 outcomes
Outcome was applicable to intervention arm, but not control arm	1 outcome
Effect size level	
Effect size involved measure very far in baseline or very far in follow-up period. Effect was not plausibly attributed to strategy. (Note: Another effect size from shorter time period was available, so outcome itself was not excluded.)	90 effect sizes
Baseline measure in intervention arm was >=95%	44 effect sizes
Outcome measure level (ITS outcomes only)	
Data error	1 measure from 1 outcome
Obvious outlier that was not plausible (baseline or follow-up; usually confirmed by study author)	7 measures from 3 outcomes
Very early in the baseline period (beyond 6 data points and beyond 48 months before intervention)	72 measures from 11 outcomes
Very late in the follow-up period (beyond 6 data points and beyond 48 months after intervention)	6 measures from 2 outcomes
